# From Design to Synergy: Triazine–Triazole Hybrids as Anti-Breast Cancer Leads Targeting Survival and Invasion Pathways for Augmenting Lapatinib Efficacy

**DOI:** 10.3390/ph19101656

**Published:** 2026-10-20

**Authors:** Omnia M. Ali, Samar Hafez Abbas, Micheal G. Shehat, Maged W. Helmy, Moustafa H. Aly, Mohamed Teleb, Sherine N. Khattab, Mohamed Abdel-Aziz

**Affiliations:** 1 Department of Medicinal Chemistry, Faculty of Pharmacy, Minia University, Minia 61519, Egypt; omnia.ali810@yahoo.com or omnia.ali4492@aast.edu (O.M.A.); samar_hafez@mu.edu.eg (S.H.A.); 2 Department of Medicinal Chemistry, College of Pharmacy, Arab Academy for Science, Technology and Maritime Transport, El-Alamein 5060305, Egypt; 3 Department of Medicinal Chemistry, Faculty of Pharmacy, Minia National University, New Minia 61768, Egypt; 4 Department of Microbiology and Immunology, Faculty of Pharmacy, Alexandria University, Alexandria 21521, Egypt; michael.shehat@alexu.edu.eg; 5 Pharmacology and Toxicology Department, Faculty of Pharmacy, Damanhour University, Damanhour 22511, Egypt; maged.helmy@pharm.dmu.edu.eg; 6 Pharmacology and Toxicology Department, Faculty of Pharmacy, Damanhour National University, Damanhour 22511, Egypt; 7 College of Engineering and Technology, Arab Academy for Science, Technology and Marine Transport, El-Alamein 5060305, Egypt; mosaly@aast.edu; 8 Department of Pharmaceutical Chemistry, Faculty of Pharmacy, Alexandria University, Alexandria 21521, Egypt; mohamed.t.ismail@alexu.edu.eg; 9 Department of Medicinal Chemistry, Faculty of Pharmacy, Alamein International University (AIU), El-Alamein 5060310, Egypt; 10 Chemistry Department, Faculty of Science, Alexandria University, Alexandria 21511, Egypt

**Keywords:** AXL inhibitors, lapatinib, 1,3,5-triazines, 1,2,3-triazoles, click reactions, isocyanate synthesis, MCF-7, synergistic combination, molecular docking

## Abstract

**Background/Objectives**: Breast cancer is the most common cancer diagnosed and the leading cause of cancer-related deaths among women, ranking fourth overall in both sexes. Its treatment is difficult due to drug resistance and side effects from conventional therapies. Developing multitargeted agents and combination approaches offer a promising way to overcome these challenges. This study details the design and testing of new triazole–triazine hybrids as potential anti-breast cancer drugs, exploring their use in combination with lapatinib to enhance its efficacy in breast cancer. **Methods**: Sixteen triazine–triazole hybrid compounds with amide and ureido linkers were synthesized via click and coupling protocols and tested for their cytotoxic effects on MCF-7 cells using an MTT assay. The most effective compound was evaluated for its inhibitory effect against AXL using an ADP-Glo™ kinase assay, then examined in combination with lapatinib on cellular and molecular levels. In vitro experiments included chemoinvasion assays for metastasis, flow-cytometric analysis of apoptosis, and ELISA to evaluate the molecular mechanisms of promising hits. Molecular docking studies were conducted to simulate the potential target–hit interactions and predict the plausible structural determinants of activity, paving the way for further lead optimization. **Results**: Compound **6e** demonstrated the highest activity (IC_50_ = 24.58 μM) and good selectivity (SI = 7.87). The combination therapy showed a synergistic effect and increased tumor sensitivity to lapatinib. Biological tests indicated inhibition of the AXL enzyme, downregulation of key pathways (AKT, NF-κB, STAT3, ERK), decreased levels of VEGF, MMP9, and cyclin D1, and increased caspase-3 activity, suggesting apoptosis, which was strengthened by the flow-cytometric analysis findings. Docking results supported multitarget interactions. **Conclusions**: Compound **6e** is a promising multitarget anti-breast cancer candidate with synergistic potential in combination with lapatinib, warranting further in vivo studies.

## 1. Introduction

Second only to cardiovascular disease in terms of sickness and mortality, cancer continues to be one of the leading causes of death globally [1]. With 2.3 million new cases in both sexes, breast cancer is currently the most common disease diagnosed, surpassing lung cancer and accounting for one in eight cancer diagnoses [2]. It makes up 25% of all female cancer cases, and its prevalence is rising in many parts of the world, particularly in transitioning nations [3,4]. Globally, breast cancer claimed 670,000 lives in 2022 [5,6].

**Figure 1:**
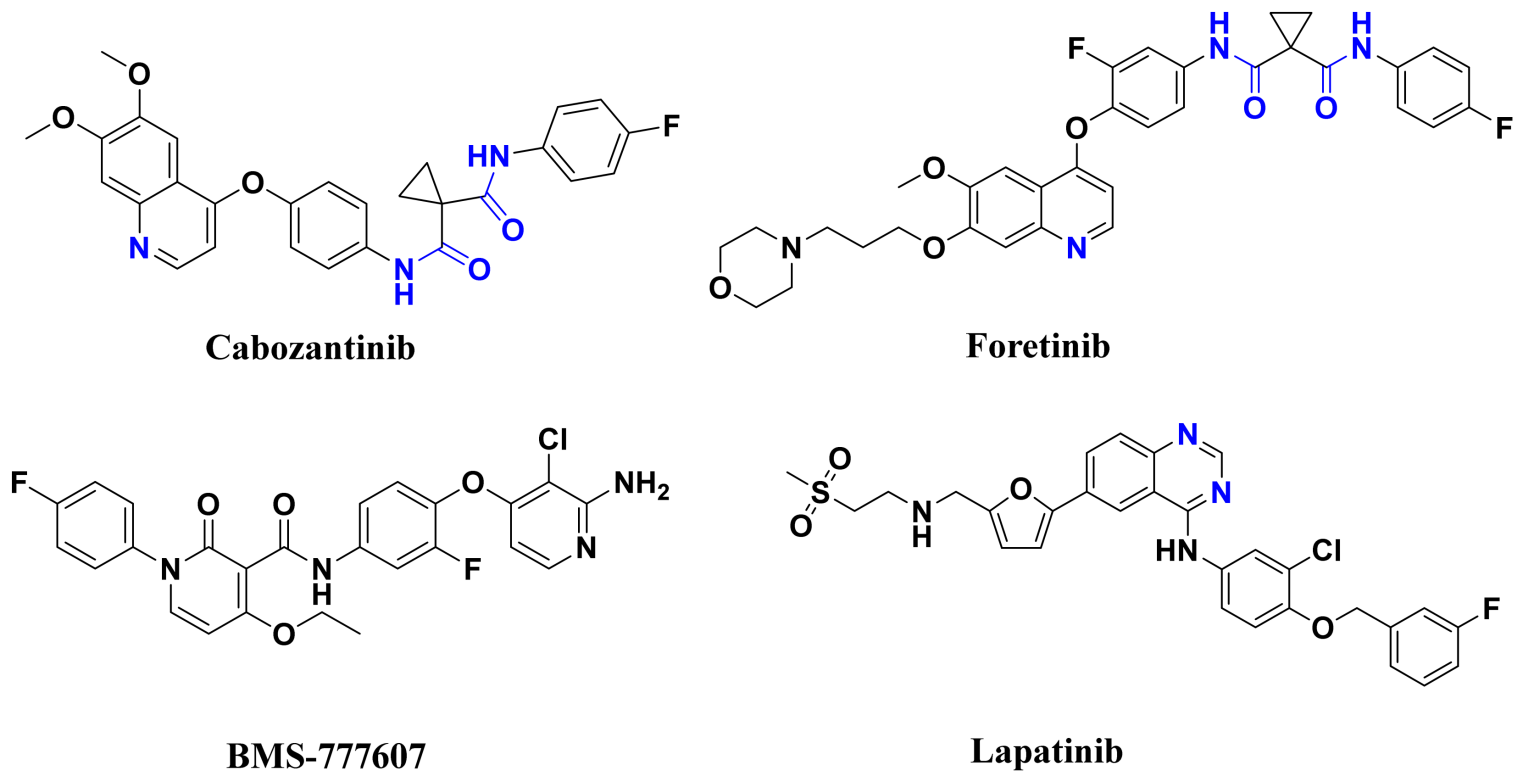
Some clinically investigated tyrosine kinase inhibitors.

Lapatinib (Figure 1) is an orally given tyrosine kinase inhibitor dually targeting epidermal growth factor receptor (EGFR) and human EGFR-2 (HER2). Both are frequently altered in tumors [7]. Its anticancer properties are obtained by binding reversibly to the ATP site of tyrosine kinase receptors within cells, blocking downstream signaling. This eventually leads to the inhibition of cell development and the activation of programmed cell death in malignant cells [8]. EGFR is a member of the ErbB family of transmembrane tyrosine kinase receptors [9]. Upon EGFR–ligand interaction, the PI3K-Akt pathway downstream signaling cascades are activated [10]. PI3K regulates cell proliferation, malignant transformation and migration. Furthermore, it is involved in modulation of MMP9 expression and activity in breast cancer [11]. HER2 is a specialized receptor tyrosine kinase found as a transmembrane glycoprotein receptor in solid tumors. It is a member of the HER family, which is known to be the oncogenic driver across several solid tumors [12]. It is linked to several potent oncogenic downstream pathways, including phosphatidil-inositol-3-kinase (PI3K) and mitogen-activated phosphokinases (MAPKs), leading to cancer cell proliferation and preventing apoptosis, cell growth, survival and differentiation [12].

AXL (a member of the TAM (TYRO3, AXL, MERTK) family of RTKs that is essential for cancer progression and resistance) also activates the PI3K pathway through p85, which induces cell proliferation [13]. When AXL binds to its main ligand, growth arrest-specific protein 6 (GAS6), it triggers signaling pathways that control cell proliferation, survival, angiogenesis, immune regulation, and migration [14,15]. The activated signaling pathways include the NF-*κ*B pathway, MAPK, extracellular signal-regulated kinase (ERK), and PI3K/AKT (protein kinase B), along with signal transducer and activator of transcription signaling (STAT) pathways [16]. Crosstalk has been reported between EGFR signaling and AXL in different cancers, including breast cancer. EGFR inhibition leads to homodimerization or heterodimerization of cell surface receptor tyrosine kinases, including AXL [17].

On the other hand, preclinical studies show that AXL causes resistance to lapatinib in breast cancer [18]. As a result, blocking AXL signaling has attracted a lot of attention as a possible breast cancer treatment since 2009 [13]. Its genetic knockdown or treatment with foretinib (AXL, MET and VEGFR inhibitor) restores lapatinib response, being anti-HER. Also, AXL inhibition leads to the disruption of tumor growth and metastasis through the dysregulation of other angiogenic factors and signaling pathways like vascular endothelial growth factor (VEGF), extracellular matrix remodeling enzymes like matrix metalloproteinase-9 (MMP9), and cell-cycle regulators like cyclin D1, highlighting the need to concurrently target multiple molecular pathways [19,20].

Structure–activity relationship (SAR) studies of reported AXL inhibitors, including cabozantinib, foretinib, and BMS-777607 (Figure 1) [21], revealed several essential pharmacophoric features required for potent kinase inhibition, as they share common structural characteristics involving heteroaromatic cores linked to substituted aromatic moieties through amide- or urea-like spacers. Most active AXL inhibitors possess nitrogen-rich heteroaromatic scaffolds capable of occupying the ATP-binding site and anchoring the inhibitor by forming key hydrogen bond interactions with hinge-region residues of the AXL kinase domain. In addition, hydrophobic aromatic systems are important for enhancing receptor occupancy and stabilizing ligand binding through hydrophobic and π–π interactions within the kinase pocket. Moreover, linker functionalities such as amide and urea groups play a critical role in providing conformational flexibility and improving ligand–receptor stabilization through hydrogen bond formation [22].

**Figure 2:**
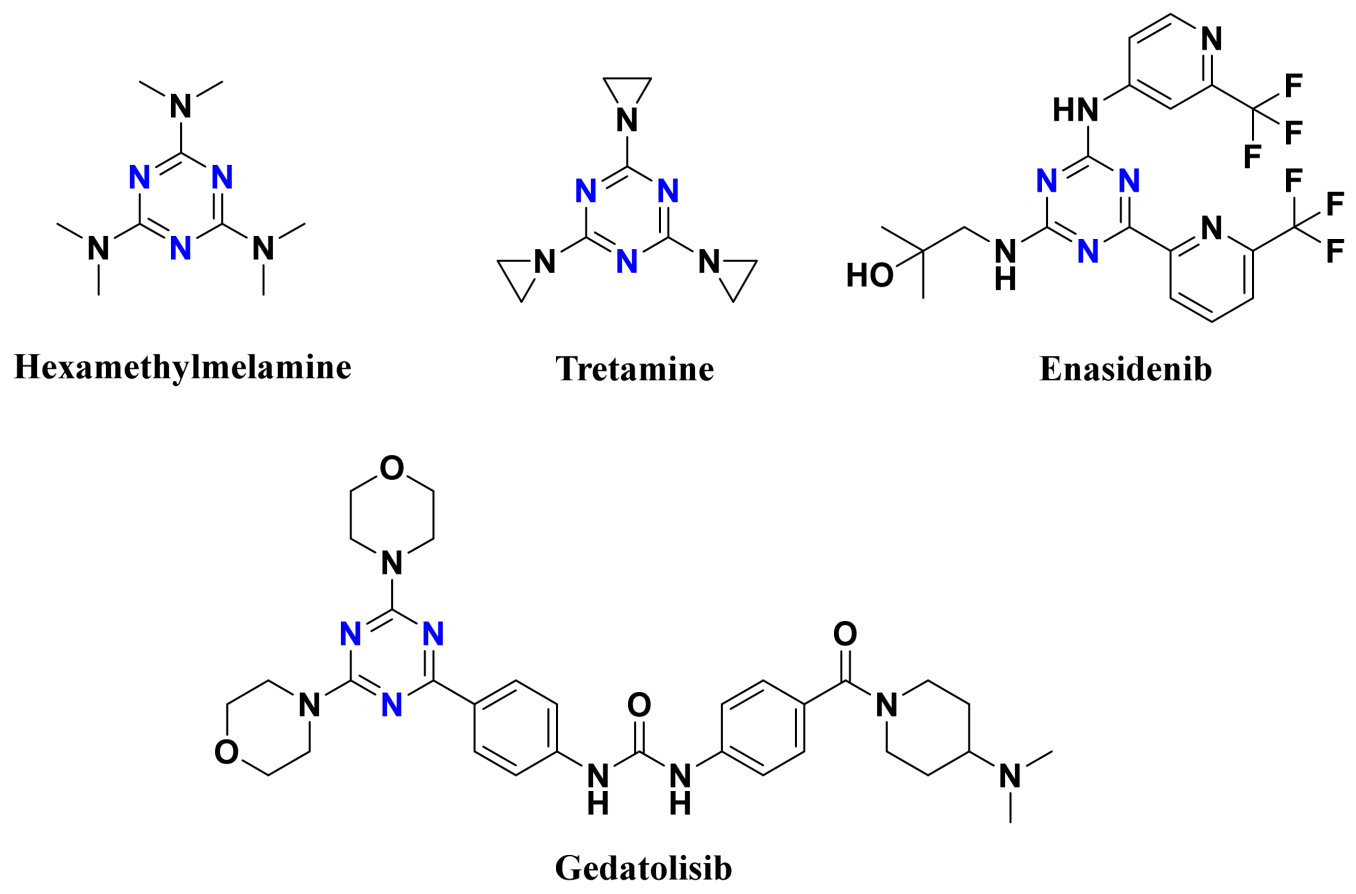
FDA-approved anticancer agents with 1,3,5-triazine scaffold.

The 1,3,5-triazine scaffold has been extensively investigated as an anticancer agent and has drawn considerable interest in drug discovery initiatives [23]. One of the first 1,3,5-triazine derivatives to receive FDA approval since 1990 as an antineoplastic drug against various cancers, such as ovarian cancer, lung cancer, and metastatic breast cancer, is hexamethylmelamine, shown in Figure 2 [24,25]. To date, other *s*-triazine derivatives, tretamine, gedatolisib, and enasidenib (Figure 2) [26], have also been authorized for use in leukemia, metastatic breast cancer, and refractory ovarian cancer [24,27]. Triazine hybrids were found to inhibit the secretion and mRNA levels of MMP2 and MMP9. Similarly, star-shaped triazine-based dendrimers have been synthesized to act as potent inhibitors of MMP9, 10, and 13 [28,29,30]. In addition, tri-substituted triazines were found to be potent dual inhibitors of both PI3K and mTOR, causing cell-cycle arrest and apoptosis [23].

**Figure 3:**
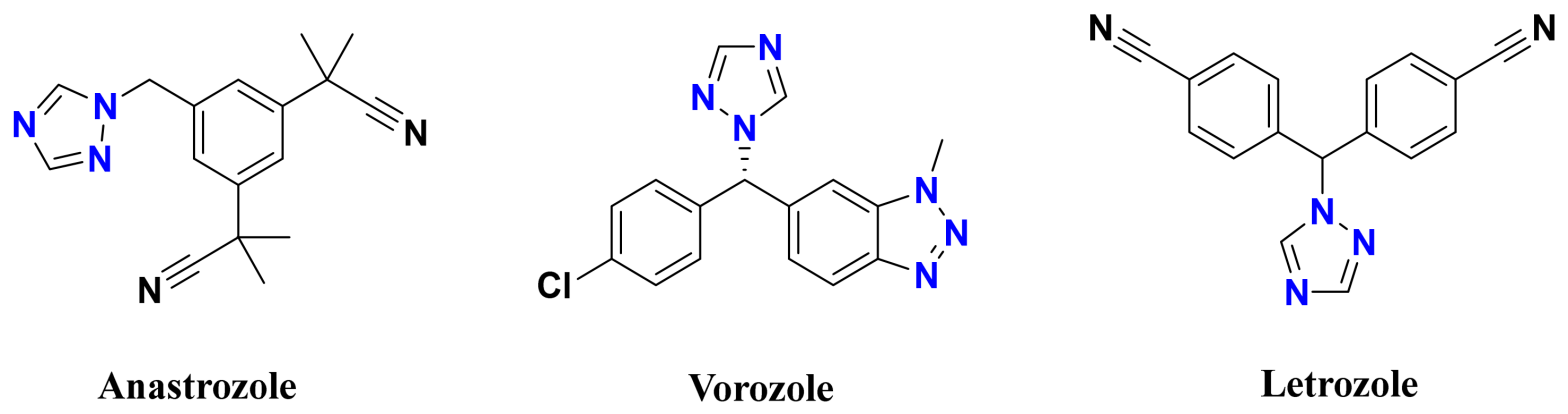
FDA-approved anticancer agents with triazole scaffold.

Further, triazoles are nitrogenous heterocycles that have three nitrogen atoms within their ring structure. They are important scaffolds in the development of pharmaceuticals because they are stable compounds that interact with biological targets through hydrogen bonding [31,32,33]. Anastrozole, vorozole, and letrozole are triazole-based FDA-approved medications that work against breast cancer (Figure 3) [26]. Triazole hybrids (such as quinoline–triazole–triazines) showed induction of cell death by causing mitochondrial damage. These compounds trigger reactive oxygen species (ROS) generation, leading to a loss of mitochondrial membranes [34]. Synthesized triazole-based hybrids, such as 1,2,3-triazoles and indolyl–triazole Schiff bases, act as targeted tyrosine kinase inhibitors via binding to the active site of the VEGFR-2 enzyme, blocking its autophosphorylation, thus leading to inhibition of angiogenesis, apoptosis and cell-cycle arrest [35].

By identifying pharmacophoric fragments in the molecular structure of two or more known bioactive derivatives, the method of rationally designing new ligands or prototypes via combining them in one derivative results in the creation of new hybrid architectures that preserve preselected traits of the original templates through the proper fusion of these subunits [36]. Also, combination therapy, in addition to anticancer natural compounds, has shown to have advantageous properties for treating malignancies in a synergistic or additive manner [37]. These combinations reduce the toxicity associated with high-dosage monotherapy, providing therapeutic benefits of the synthetic chemotherapeutic medication at lower doses, which is easily assessed using the combination index [38].

**Figure 4:**
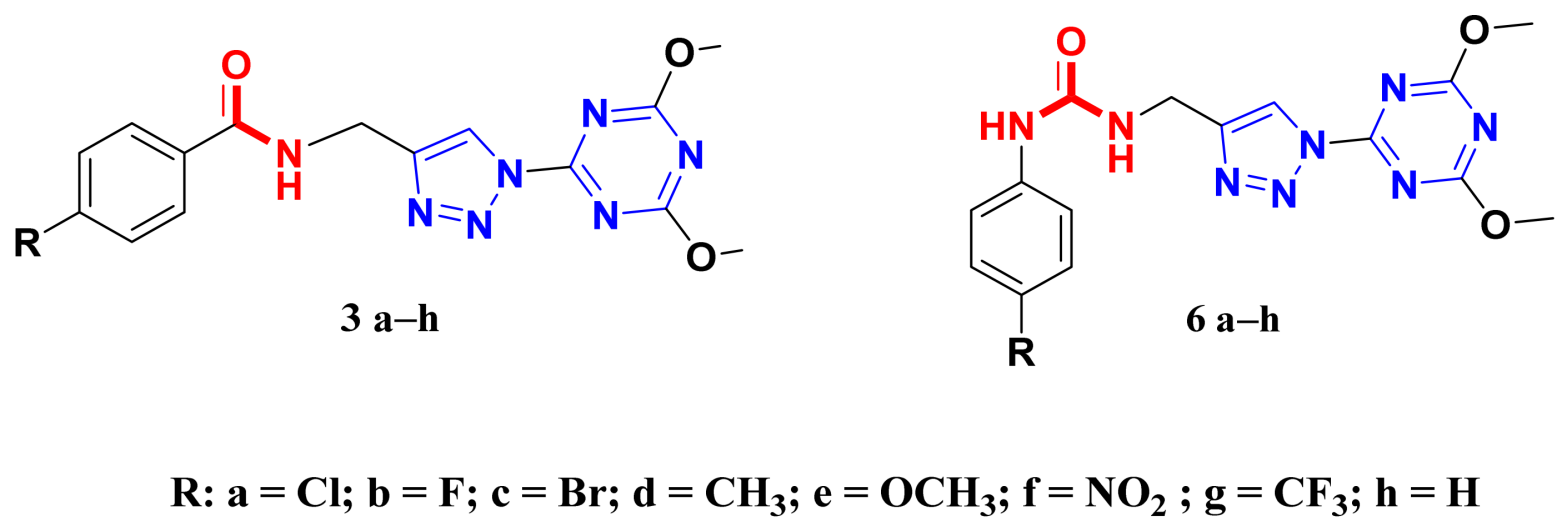
General structure of the synthesized novel triazole–triazine hybrid derivatives.

Based on these considerations, our study aims to design and synthesize a series of novel triazole–triazine hybrid derivatives linked through amide or ureido moieties (Figure 4) as AXL inhibitors as well as targeting other key oncogenic pathways—e.g., MMP9, VEGFR and caspase signaling pathways—to provide multitarget therapy for breast cancer. We adopted a molecular hybridization strategy combining two privileged anticancer pharmacophores, namely 1,2,3-triazole and 1,3,5-triazine, to design novel multitarget anti-breast cancer agents. The triazole nucleus was selected because of its well-documented kinase inhibitory properties, its function as a zinc binding group (ZBG), its hydrogen-bonding capability that enables it to bind to the active site of MMPs [29], alsoits inhibitory action against the VEGFR-2 enzyme [39], whereas the triazine scaffold represents an important pharmacophore in several anticancer kinase inhibitors that anchors the molecule within the targeted protein’s binding pocket of AXL and shows inhibitory action against PI3K, mTOR and MMPs [23,40]. In addition, there are various triazole hybrids, such as triazole–chalcones [41], triazole–pyrdines [42], triazole–quinazolinones [43] and triazole–pyrazole–pyrimidine hybrids [44].

The incorporation of amide and ureido linkers into the designed hybrids was intended to mimic the linker architecture commonly observed in reported AXL inhibitors and to enhance binding interactions within the kinase active site. Substituted aromatic rings were also introduced to improve hydrophobic interactions and optimize target affinity [22]. The most active compound was further investigated through biological studies to elucidate its effects on the key oncogenic signaling pathways involved in breast cancer progression. In addition, combination with lapatinib was investigated as a rational synergistic combination therapy approach. Although lapatinib is primarily recognized as a dual EGFR/HER2 tyrosine kinase inhibitor, accumulating evidence has demonstrated significant crosstalk between HER2 and AXL signaling pathways in breast cancer progression and therapeutic resistance [45]. AXL overexpression has been identified as an important mechanism mediating resistance to HER2-targeted therapies, including lapatinib, through persistent activation of compensatory AKT and ERK survival pathways. Consequently, simultaneous targeting of HER2-associated signaling using lapatinib together with suppression of AXL-related oncogenic pathways by compound **6e** offers a good strategy to produce synergistic anti-tumor effects and improve therapeutic efficacy against resistant breast cancer phenotypes. Molecular docking studies were also performed to gain further insight into the potential molecular interactions of the compound with relevant therapeutic targets.

## 2. Results and Discussion

### 2.1. Chemistry

**Scheme 1:**
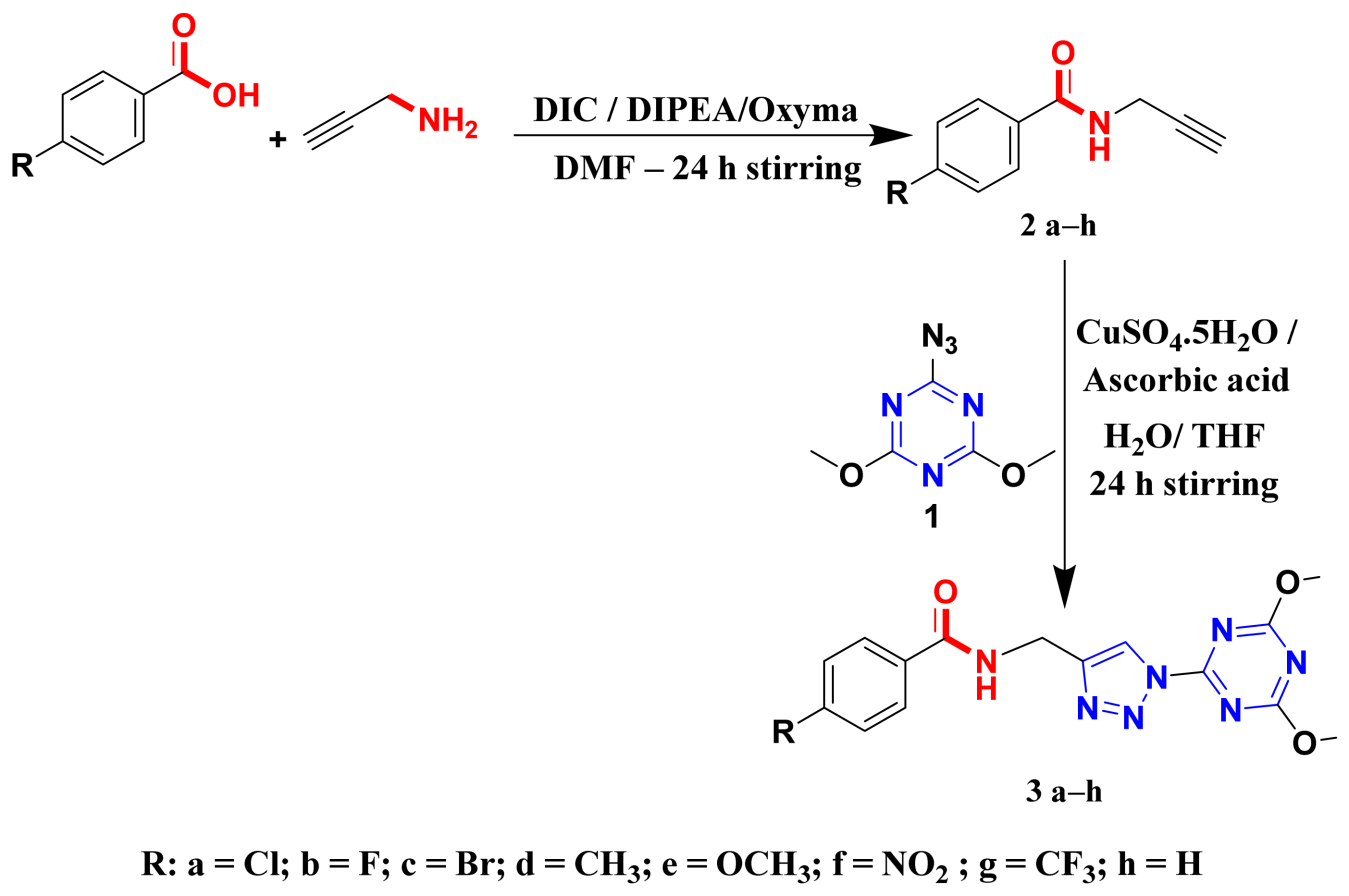
Synthesis of 4-substituted-*N*-((1-(4,6-dimethoxy-1,3,5-triazin-2-yl)-1*H*-1,2,3-triazol-4-yl) methyl) benzamide (**3a**–**h**).

The synthesis of the first series of derivatives, **3a**–**h**, was done as explained in Scheme 1. Firstly, the azido triazine 2-azido-4,6-dimethoxy-1,3,5-triazine (1) was prepared via the nucleophilic substitution of the chlorine atom of the corresponding 2-chloro-4,6-dimethoxy-1,3,5-triazine with sodium azide [46]. The mechanism of this synthetic reaction primarily involves a nucleophilic aromatic substitution (S_N_Ar) mechanism typically carried out in polar aprotic solvents like acetonitrile [47]. At the same time, the synthesis of 4-substituted-*N*-(prop-2-yn-1-yl) benzamide derivatives (**2a**–**h**) was accomplished via carbodiimide-mediated coupling of amines with carboxylic acids. In this work, the appropriate *p*-substituted benzoic acids were activated using a coupling system consisting of diisopropylcarbodiimide (DIC), *N,N’*-Diisopropylethylamine (DIPEA) and (ethyl 2-cyano-2-(hydroxyimino)acetate) oxyma [48], to enhance coupling efficiency and improve the yield of the reaction [48], and subsequently reacted with propargyl amine to yield the target amides (**2a**–**h**) (Scheme 1).

The desired final triazine–triazole hybrids **3a**–**h** were obtained through a click reaction (copper(I)-catalyzed azide–alkyne cycloaddition (CuAAC)) between 2-azido-4,6-dimethoxy-1,3,5-triazine (**1)** and the corresponding amide derivatives **2a**–**h**. Copper (I) catalyst is generated in situ from copper (II) sulfate pentahydrate and ascorbic acid as a reducing agent, giving rise to the 1,2,3-triazole ring, giving the final desired compounds **3a**–**h** (Scheme 1) [49].

The structural elucidation of compounds **3a**–**h** was confirmed through IR, ^1^H NMR, ^13^C NMR and HRMS spectral analyses. The IR spectra exhibited characteristic absorption bands corresponding to the amide N-H stretching vibrations within the range of 3500–3300 cm^−1^. Additionally, distinct bands attributed to aromatic *sp*^2^ C-H stretching were observed at 3150–3100 cm^−1^, while aliphatic *sp*^3^ C-H stretching appeared around 2950 cm^−1^. The presence of the amide functionality was further supported by the appearance of a carbonyl (C=O) stretching band in the region of 1660–1650 cm^−1^. The ^1^H NMR spectra of the synthesized compounds displayed well-defined and consistent signals, confirming their proposed structures. A characteristic singlet signal at δ: 4.00–4.02 ppm, integrated for six protons, was assigned to the two methoxy groups attached to the triazine ring. The methylene protons (-CH_2_-) linking the triazole and amide moieties appeared as singlet or doublet signals at δ: 4.54–4.62 ppm. Aromatic protons were observed as multiplets within the region of δ: 7.50–7.90 ppm, reflecting the substituted phenyl ring environment. Furthermore, the triazole C^5^–H proton resonated as a distinct singlet signal at δ: 8.65–8.74 ppm, while the amide N-H proton appeared as downfield singlet or triplet signals at δ: 8.94–9.42 ppm, which was confirmed by D_2_O exchange, indicating its labile nature. The ^13^C NMR spectra of the final compounds exhibited consistent and well-resolved peaks, supporting their proposed structures. In the aliphatic region, characteristic signals corresponding to the methylene carbons were observed at δ: 35.0–35.3 ppm, while the methoxy carbons appeared around δ 56.5 ppm. Two peaks attributed to the triazole carbons were detected around δ: 122.8 and 128.9 ppm. Additional resonances in the aromatic region were observed around δ: 129.80, 133.3, 136.8, and 146.6 ppm, consistent with the substituted phenyl ring carbons. A distinct signal at δ: 163.0 ppm was assigned to the amide carbonyl carbon, whereas peaks at *δ* 165.9 and 173.6 ppm corresponded to the triazine ring carbons [29,30]. The HRMS analysis of the synthesized compounds confirmed their molecular formula by displaying the molecular ion peak corresponding to the protonated molecules [M+H]^+^.

**Scheme 2:**
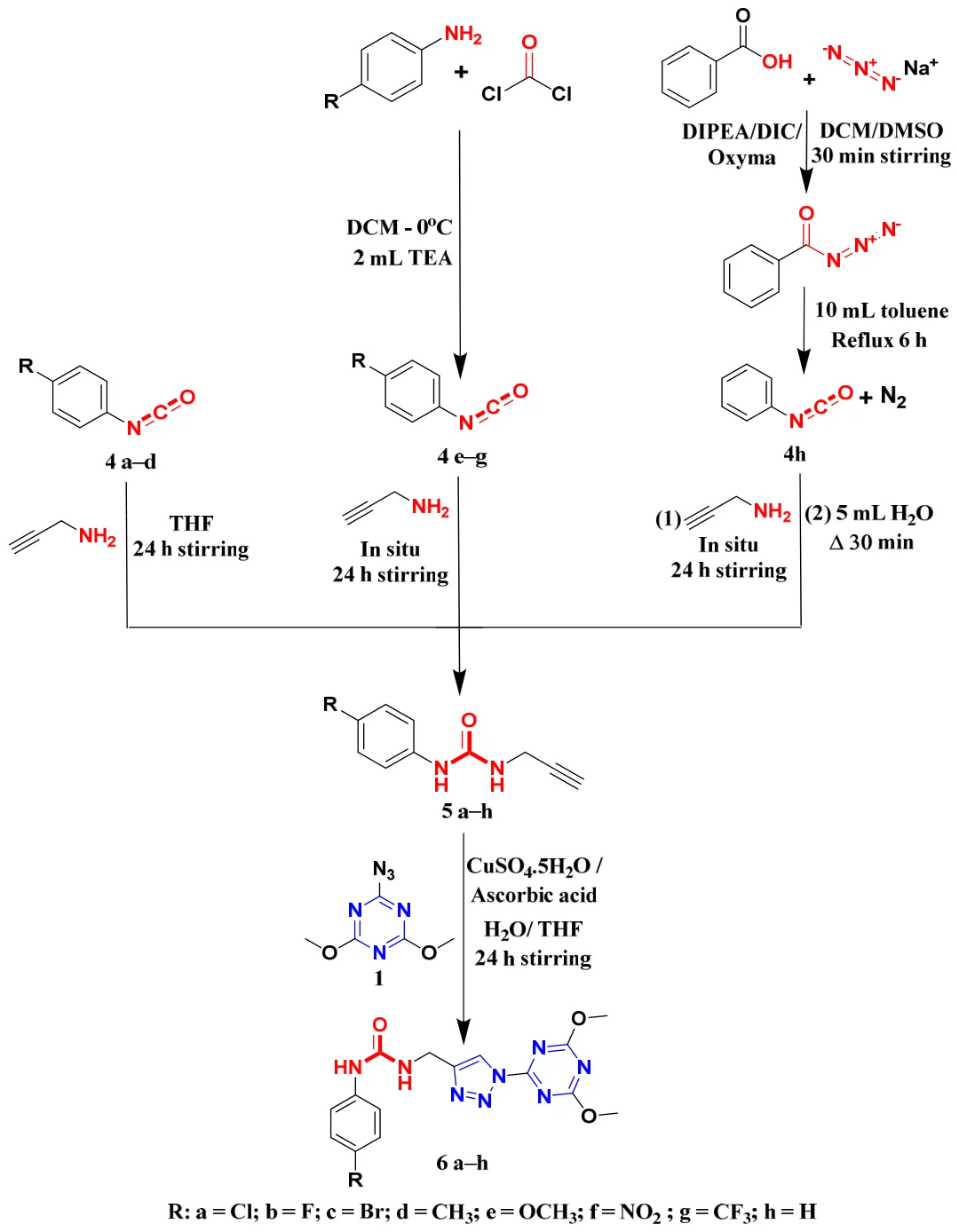
1–((1-(4,6-dimethoxy-1,3,5-triazin-2-yl)-1*H*-1,2,3-triazol-4-yl)methyl)-3-phenylurea (**6a**–**h**).

On the other hand, the approach used for synthesizing the desired compounds **6a**–**h** is sketched in Scheme 2. First, the synthesis of *p*-substituted phenyl isocyanates (**4a**–**h**) was accomplished using complementary strategies selected according to the reactivity and suitability of the corresponding derivatives. In general, conversion of the substituted anilines into the corresponding isocyanates was achieved through established methodologies, with phosgene-based protocols providing efficient access and high isolated yields, despite the need for careful handling due to its toxicity, to afford isocyanates **4e**–**g.** This transformation proceeds via initial nucleophilic attack of the aniline nitrogen on the carbonyl carbon, followed by formation of a carbamoyl chloride intermediate and subsequent dehydrohalogenation to afford the isocyanate functionality [50].

It is noteworthy that while the substituted isocyanate derivatives (**4e**–**g**) were efficiently prepared using phosgene reagent, the synthesis of the unsubstituted analog (**4h**) via this route yielded unsatisfactory purity and lower yields. Therefore, a Curtius rearrangement using NaN_3_ and oxyma was successfully employed for **4h**, offering an optimized, practical route that ensured high purity and yield while allowing a comparative evaluation of reaction efficiency and product quality. First, the coupling reaction takes place between benzoic acid in DCM and NaN_3_ in DMSO [51]. The corresponding carboxylic acid derivative is then activated using a coupling cocktail that contains DIC, oxyma and DIPEA as a base, leading to the formation of oxyma benzoyl active ester [52], followed by thermal decomposition of the resulting acyl azide to generate the isocyanate **4h** with the release of N_2_ gas [53] (Scheme 2).

The synthesis of substituted 1-(4-substituted phenyl)-3-(prop-2-yn-1-yl) urea derivatives (**5a**–**h**) was achieved using *p*-substituted phenyl isocyanates either obtained from commercial sources or generated in situ via the previously described methods [54].

In general, the corresponding urea derivatives were formed through the reaction of the appropriate isocyanates with propargyl amine in an aprotic solvent such as tetrahydrofuran (THF) [55]. For isocyanates generated in situ, propargyl amine was directly added to the reaction mixture, affording the desired urea products without isolation of the intermediate isocyanates [55] (Scheme 2). This in situ approach was used because the isolation of the formed isocyanates (**4e**–**g**) would be tedious and lead to significant material loss and reduced yields. Additionally, the use of phosgene as a reagent presents substantial safety risks; thus, a direct in situ approach was necessary to minimize handling hazards. This streamlined procedure ensured safer handling and allowed the final propargyl ureas **5e**–**g** to be obtained in adequate yields, comparable to the isolated approach used for **4a**–**d**.

The desired final compounds **6a**–**h** were obtained through a click reaction between 2-azido-4,6-dimethoxy-1,3,5-triazine (**1**) and the synthesized urea derivatives **5a**–**h** (Scheme 2). The structures of compounds **6a**–**h** were confirmed by IR, ^1^H NMR, ^13^C NMR and HRMS spectroscopy. The IR spectra showed characteristic absorption bands corresponding to N-H stretching vibrations in the range of 3400–3300 cm^−1^. In addition, the presence of aromatic and aliphatic groups was supported by the *sp*^2^ C-H stretching bands around 3100 cm^−1^ and *sp*^3^ C-H stretching bands near 2950 cm^−1^. The appearance of a distinct band at approximately 1650 cm^−1^ further confirmed the presence of the ureido group. The ^1^H NMR spectra provided additional confirmation of the proposed structures. A singlet signal at *δ* 4.02–4.03 ppm, integrated for six protons, was assigned to the two methoxy groups attached to the triazine ring. The methylene protons linking the triazole and ureido moieties appeared as singlet or doublet signals at *δ* 3.66–4.47 ppm. The aromatic protons were observed within the region of *δ* 7.20–7.50 ppm, consistent with substituted phenyl rings. The triazole C^5^-H proton signals appeared at *δ* 8.33–9.32 ppm. Moreover, the two ureido NH protons were observed as two singlet or triplet and singlet signals at *δ* 6.15–7.12 ppm and at *δ* 8.65–9.54 ppm, respectively, both disappearing upon D_2_O exchange, confirming their exchangeable nature. The ^13^C NMR spectra of the final compounds exhibited consistent and well-resolved peaks, supporting their proposed structures. In the aliphatic region, characteristic signals corresponding to the methylene carbons were observed at *δ* 35.1–35.2 ppm, while the methoxy carbons appeared around *δ* 56.5 ppm. Two peaks attributed to triazole carbons were detected around *δ* 118.5 and 122.2 ppm. Additional resonances in the aromatic region were observed around *δ* 125.5, 129.8, 138.5, and 147.4 ppm, consistent with the substituted phenyl ring carbons. A distinct peak at *δ* 155.5 ppm was assigned to the ureido carbonyl carbon, whereas peaks around *δ* 162.9 and 173.5 ppm corresponded to the triazine ring carbons. The HRMS analysis of the synthesized compounds confirmed their molecular formula by displaying the molecular ion peak corresponding to the protonated molecules [M+H]^+^.

It is also worth mentioning that the exclusive formation of the 1,4-disubstituted 1,2,3-triazole isomer via the strictly regioselective CuAAC reaction [56] was confirmed by the diagnostic ^1^H NMR singlet for the triazole proton and characteristic ^13^C NMR resonances (triazole CH around *δ* 122 ppm and quaternary carbon around *δ* 129 ppm), ensuring reliable geometry for the subsequent molecular docking analysis [57].

### 2.2. Biological Evaluation

#### 2.2.1. Cytotoxicity Screening on Human Breast Cancer (MCF-7) and Normal Human Skin Fibroblasts

Experiments were conducted to evaluate the cytotoxic activities of compounds **3a**–**h** and **6a**–**h** against the MCF-7 breast cancer cell line compared to lapatinib in terms of IC_50_, and to determine the safety profile of the studied compounds, their cytotoxic effects were evaluated on human skin fibroblast cells (HSFs), all via an MTT assay, as shown in Table 1.

Compounds **3a**, **3f**, **3g**, **3h**, and **6e** showed moderate cytotoxicity against MCF-7 (Figure S1) cells while demonstrating markedly lower potency against normal HSF (Figure S2), indicating a significant degree of selectivity. Compound **6e** demonstrated the highest antiproliferative activity manifested through its IC_50_ (24.58 μM), comparable to the activity of the reference drug lapatinib (21.06 ± 1.11 μM). In addition, compounds **3c**, **3f**, **3g**, **3h**, **6c** and **6d** displayed acceptable potency against the MCF-7 cell line, with IC_50_ values ranging from 58 to 107 μM.

Beyond potency, the selectivity index (SI) value is critical in determining the safety and appropriateness of synthetic anticancer therapeutic compounds. Compounds with SI values ≥ 3 are generally considered to have an appropriate safety profile towards cancer cells [58]. In our study, we determined the selectivity index (SI) values, as shown in Table 1, by comparing the IC_50_ ratios between normal and tumor cells. In addition to being highly potent, **6e** showed the highest selectivity index value (SI = 7.87) of all the compounds tested, suggesting a considerable preference for cytotoxicity against cancer cells over normal fibroblasts. Thus, **6e** is the most active of our compounds due to its strong antiproliferative action and exceptional selectivity. This compound’s performance demonstrates a favorable balance between efficacy and safety; hence, it was chosen for more biological and mechanistic studies.

#### 2.2.2. Biochemical and Structural Assay of AXL

##### Biochemical Kinase Assay of AXL Enzyme

**Figure 5:**
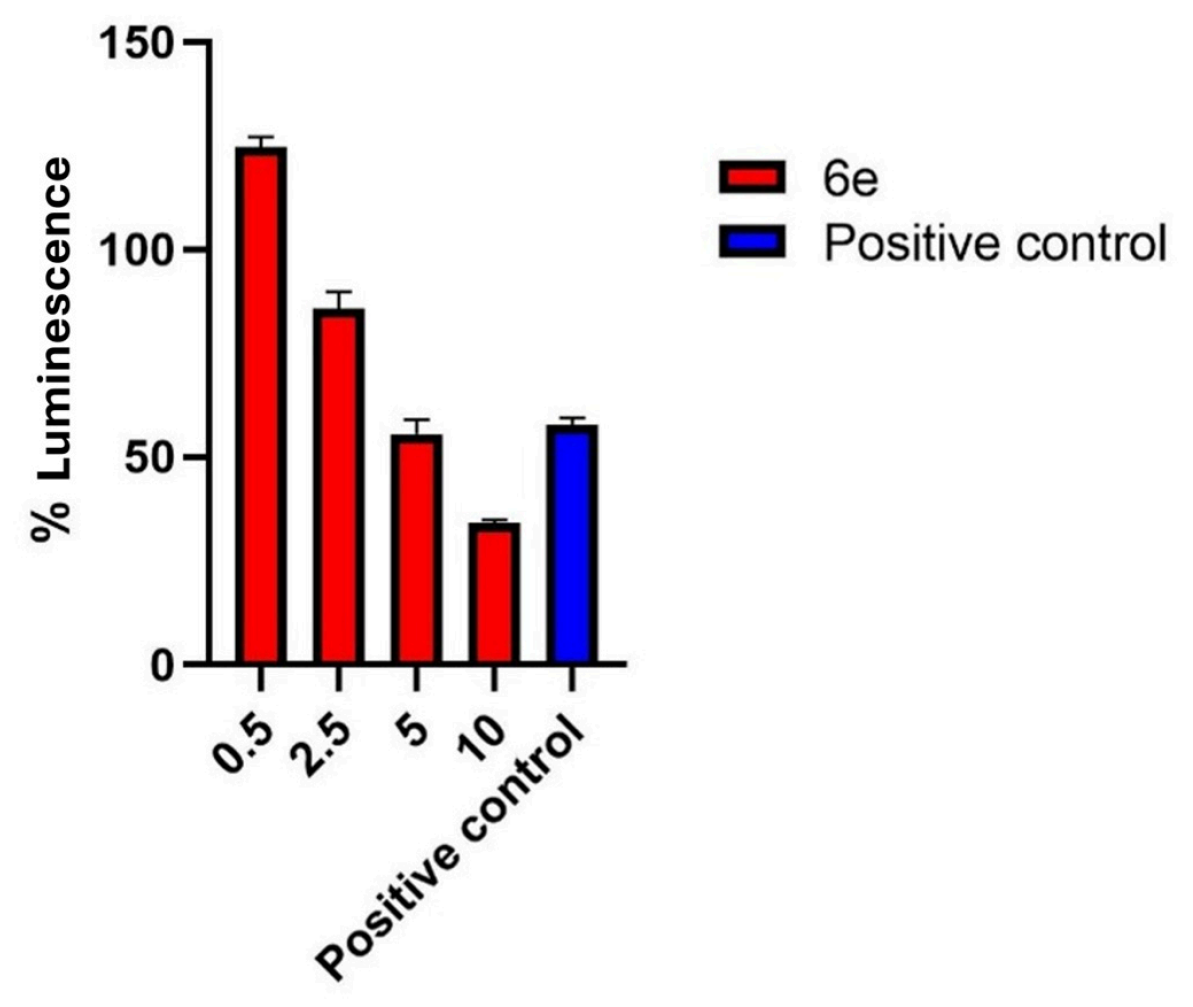
Concentration-dependent reduction of AXL kinase activity induced by compound **6e**, measured by the ADP-GloTM kinase assay. Experimental 95% confidence range of 5.0–10.0 μM.

The ADP-Glo™ kinase assay was used to biochemically confirm the synthesized **6e** compound’s ability to directly inhibit AXL kinase activity. The assay results in luminescence intensity that is directly proportional to enzymatic activity through quantification of ADP produced during the kinase reaction (Figure 5). Consequently, the observed concentration-dependent reduction in luminescence following treatment with **6e** indicates the effective suppression of AXL catalytic activity (Table 2) [59].

The results showed that **6e** can directly block the enzymatic activity of the receptor tyrosine kinase, AXL. Additionally, when the concentration of **6e** increased, the luminescence intensity gradually decreased, which was closely correlated with kinase enzymatic activity and showed that the kinase reaction effectively suppressed the generation of ADP. Compound **6e** exhibited a slightly higher luminescence intensity at 0.5 μM compared to the DMSO negative control, resulting in negative inhibition values. This behavior could be explained by the compound’s sub-inhibitory concentration, leading to normal enzyme activity, resulting in luminescence levels above baseline controls. As the concentration of **6e** increases, luminescence intensity decreases as evidence of a marked increase in the inhibition effect, with 14.26% inhibition at 2.5 μM, 44.49% inhibition at 5 μM and reaching a high inhibition percentage of 65.79% at 10 μM, surpassing the positive control (Bosutinib), highlighting its strong biochemical potency, with a calculated IC_50_ = 6.3 μM.

##### Molecular Docking Analysis of **6e**

To gain insight into the binding mode of compound **6e** within the catalytic domain of AXL kinase and to identify its most plausible bioactive conformation, a molecular docking study was conducted using the crystal structure of AXL kinase (PDB ID: 5TC0). The docking calculations were performed using Flare™ version 12.0.0 (Cresset, Litlington, UK), employing the Lead Finder docking engine for pose generation and scoring. The crystal structure was prepared using the default Protein Preparation Wizard protocol, which included the addition of missing hydrogen atoms, removal of non-essential crystallographic components, assignment of formal and partial charges, optimization of amino acid side-chain orientations, and subsequent energy minimization to eliminate unfavorable steric contacts.

**Figure 6:**
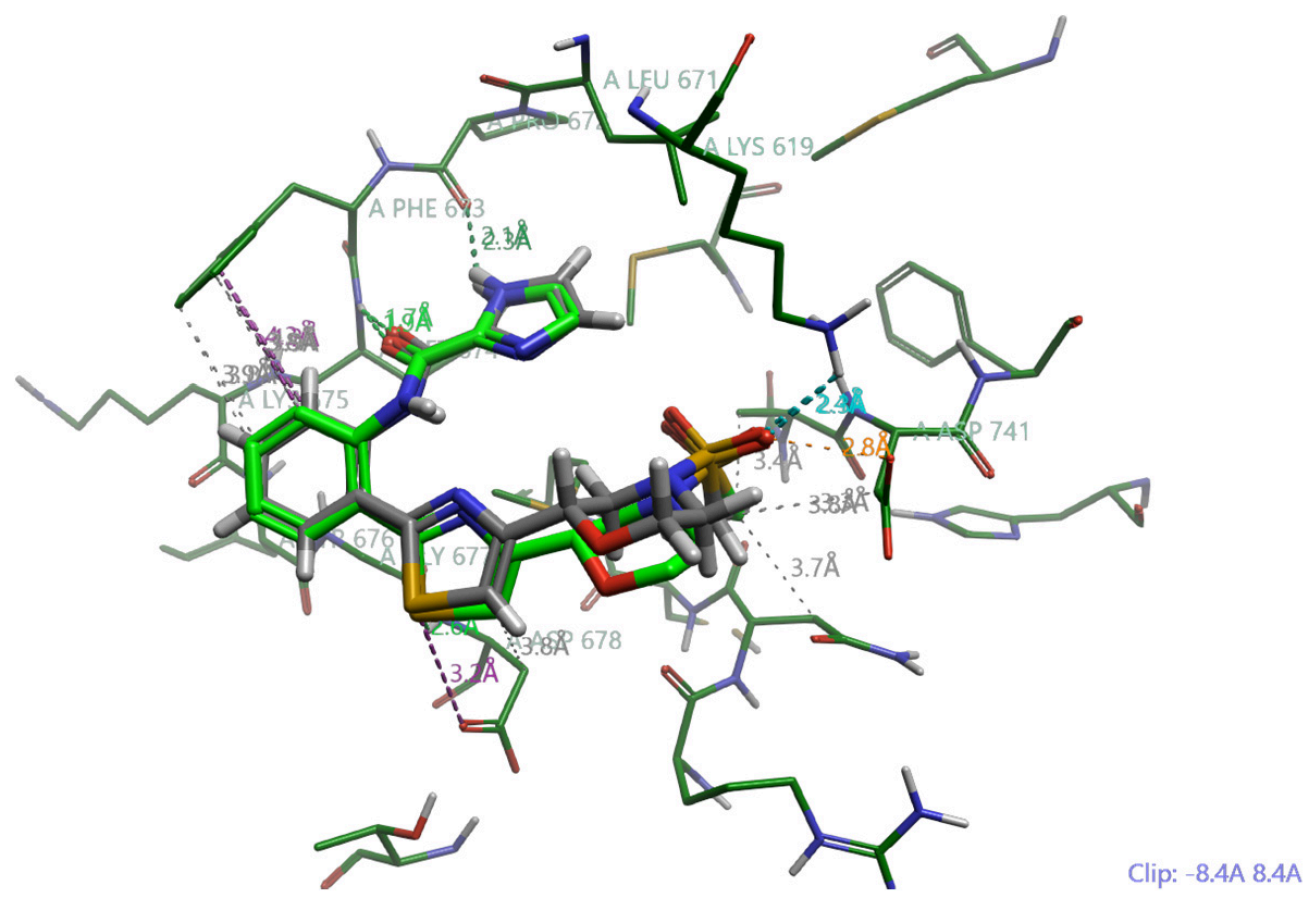
3D representation of the overlay of the co-crystallized ligand (gray) and its predicted docking pose (light green) within the catalytic site (dark green) of AXL (PDB: 5TC0). Aromatic π–π interaction is shown as violet dashed lines, hydrophobic interaction as gray dashed lines and hydrogen bonds as green dashed lines.

**Figure 7:**
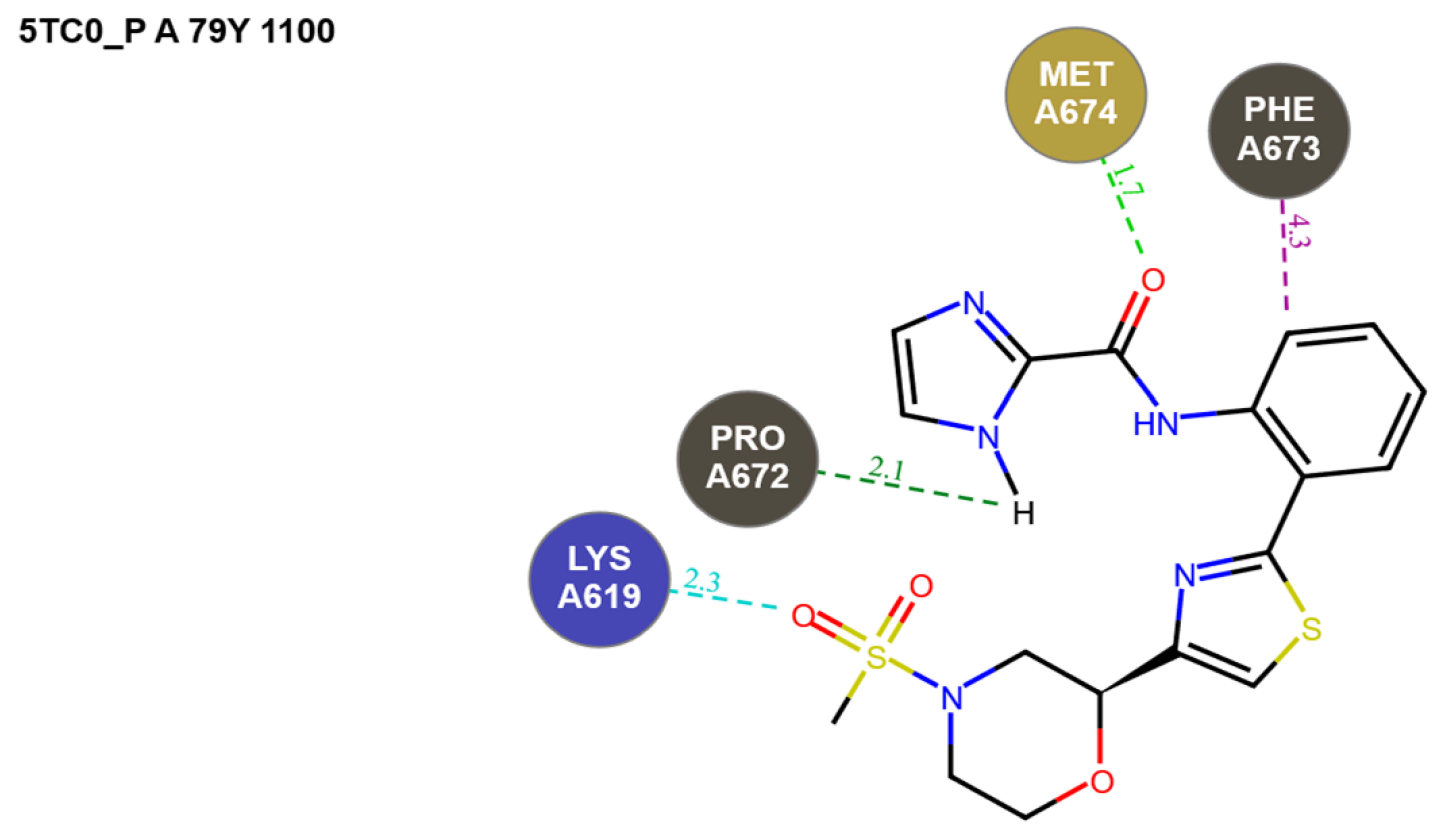
2D interaction diagram for both the co-crystallized ligand and its predicted docking pose within the AXL (PDB: 5TC0) catalytic site. Aromatic π–π interaction is shown as violet dashed lines, hydrophobic interaction as gray dashed lines and hydrogen bonds as green and cyan dashed lines.

To validate the reliability of the docking protocol, the co-crystallized ligand was extracted from the AXL structure and subsequently re-docked into the ATP-binding site. The adopted protocol successfully reproduced the experimentally observed binding mode of the native ligand, yielding a docking score of −9.819 kcal/mol and an RMSD value of 0.813 Å between the predicted and crystallographic poses. These results indicate excellent agreement with the experimental structure and demonstrate the capability of the docking methodology to accurately reproduce the native ligand orientation within the active site (Figure 6 and Figure 7). Furthermore, the re-docked pose retained the essential interactions observed in the crystal structure, confirming the suitability of the employed protocol for subsequent docking studies [60].

Following validation, compound **6e** was docked into the ATP-binding pocket of AXL kinase. Several binding conformations were generated and carefully evaluated based on docking scores, interaction profiles, and the absence of unfavorable steric clashes. The selected binding pose exhibited a docking score of −9.011 kcal/mol, which is comparable to that of the co-crystallized ligand, suggesting a favorable binding affinity toward the target protein [61].

**Figure 8:**
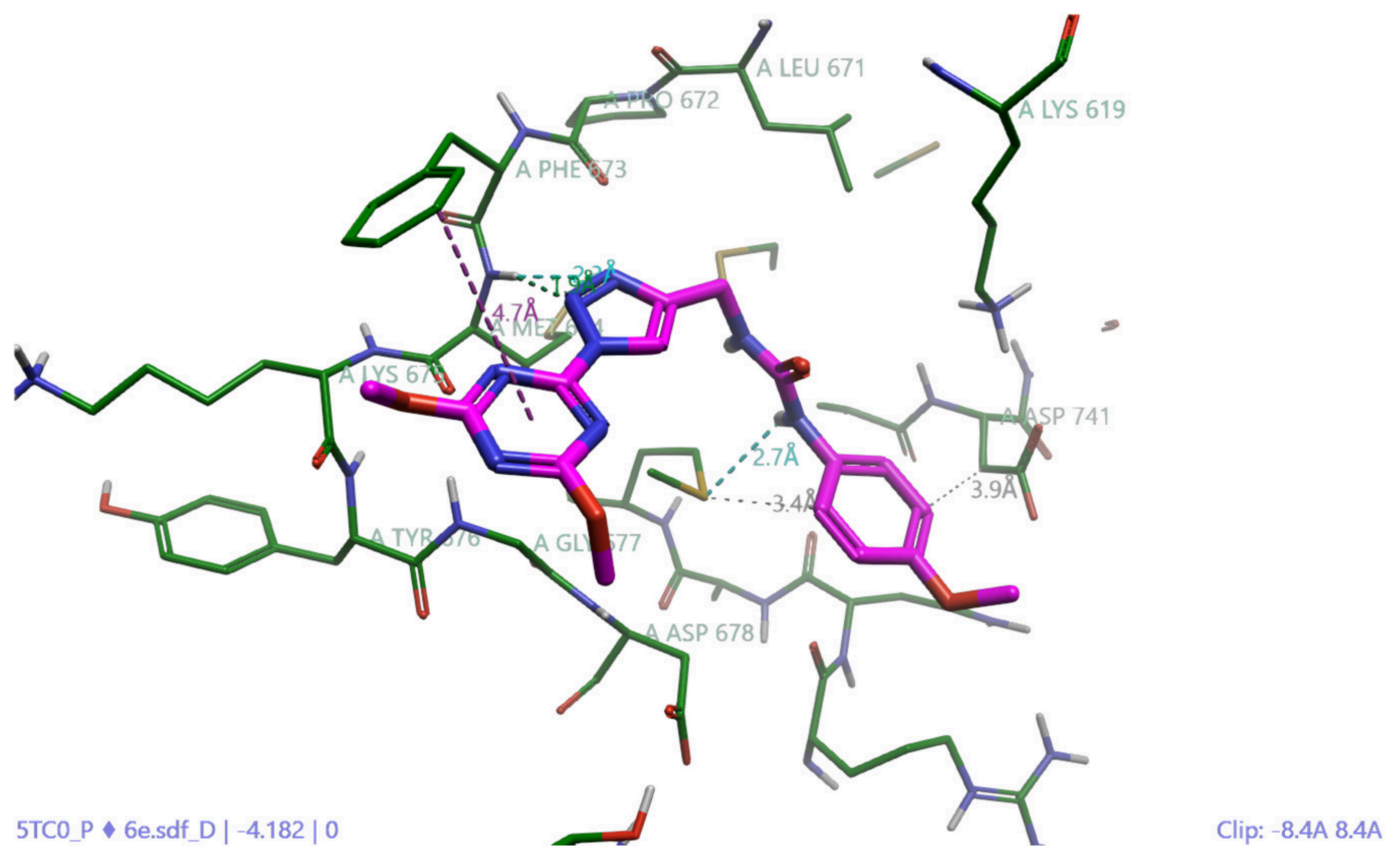
Docking pose of compound **6e** in the crystal structure of AXL (crystal structure PDB: 5TC0). The protein backbone is represented as dark green and the ligand as pink sticks. Aromatic π–π interaction is shown as violet dashed lines, hydrophobic interaction as gray dashed lines and hydrogen bonds as cyan and green dashed lines.

**Figure 9:**
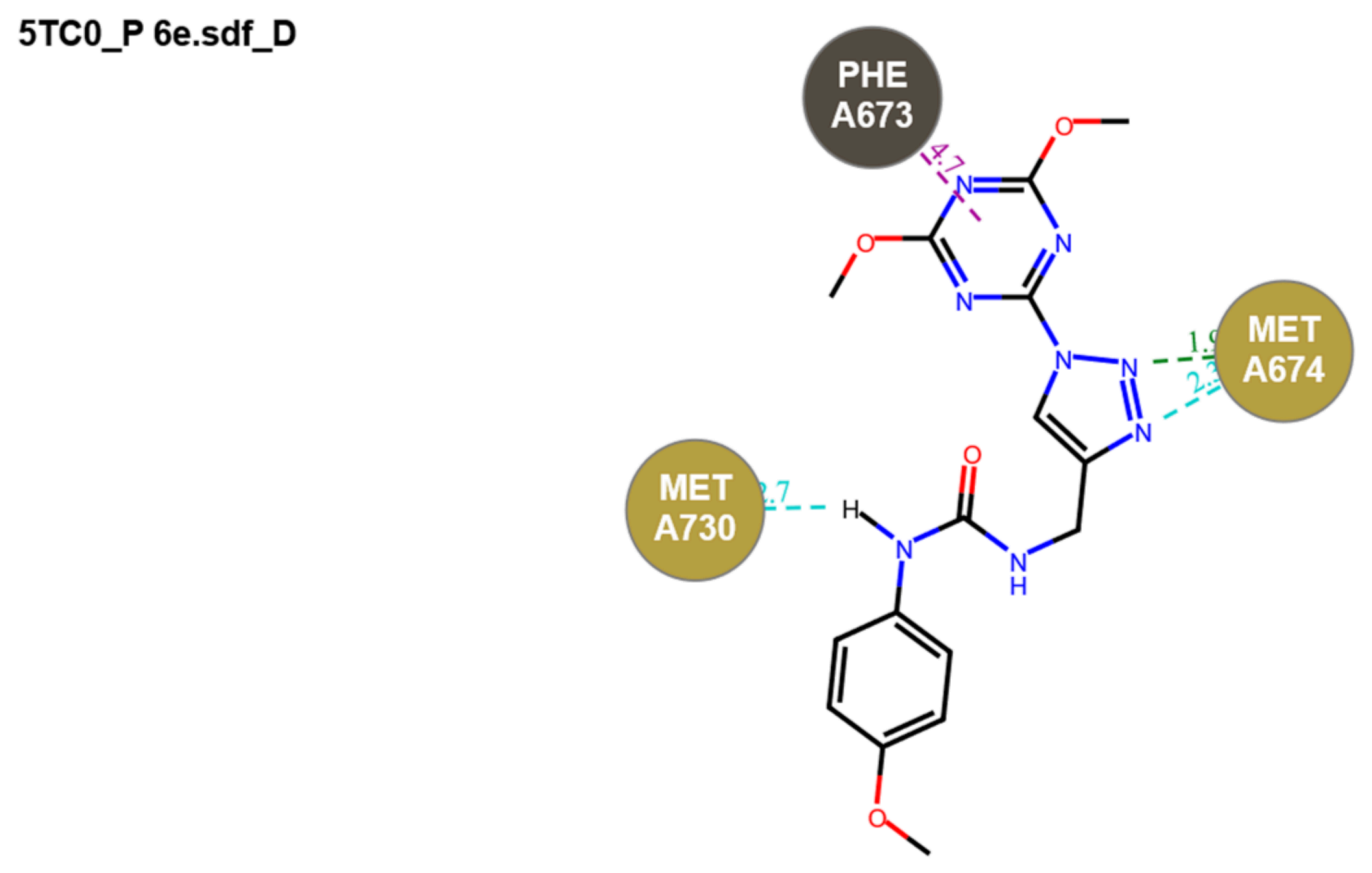
2D interaction diagram for predicted docking pose of **6e** within AXL (PDB: 5TC0) catalytic site. Aromatic π–π interaction is shown as violet dashed lines, hydrophobic interaction as gray dashed lines and hydrogen bonds as green and cyan dashed lines.

Analysis of the predicted binding mode revealed that compound **6e** preserves key interactions required for effective kinase inhibition. The nitrogen atoms of the 1,2,3-triazole ring established a crucial hydrogen-bonding interaction with the amide backbone of Met674 within the hinge region, an interaction commonly observed among ATP-competitive kinase inhibitors and considered essential for high-affinity binding. In addition, the triazine scaffold engaged in a π–π stacking interaction with the aromatic side chain of Phe673, corresponding to the interaction pattern observed for the co-crystallized ligand. The urea linker further contributed to ligand stabilization through an additional hydrogen-bonding interaction with the Met730 side chain, thereby enhancing anchoring of the ligand within the catalytic cleft. Collectively, these interactions suggest that compound **6e** adopts a binding mode highly consistent with known AXL inhibitors, and provide a structural rationale for its observed biological activity (Figure 8 and Figure 9).

In conclusion, the preservation of the characteristic hinge-binding interactions, combined with favorable aromatic stacking and additional stabilizing hydrogen bonds, supports the proposed mechanism of AXL inhibition by compound **6e**. Moreover, the comparable docking score relative to the co-crystallized ligand further reinforces the potential of **6e** as a promising AXL-targeted inhibitor.

Also, the high activity of the methoxy-substituted **6e** over the halogenated (Cl, F, Br) and electron-withdrawing NO_2_ and CF_3_ analogs could be attributed to the mild electron-donating resonance effect and compact steric profile of the OCH_3_ group, which help preserve the optimal bioactive conformation and electronic environment required for the key hinge-region interactions (Met674, Phe673, and Met730) described above in our docking study [62].

#### 2.2.3. Combination Studies with Lapatinib

##### MTT Assay and Determination of Combination and Dose-Reducing Indices

The MTT assay demonstrated that **6e** exhibited comparable anti-breast cancer potential to lapatinib, as indicated by their low IC_50_ values (24.58 μM and 21.06 μM, respectively). To investigate the antiproliferative activity of **6e** as an adjuvant to lapatinib against MCF-7 cells, it was combined with lapatinib at different fixed ratios (1:1, 1:2, 2:1) and incubated with the cells; then, MTT experiments were carried out as described [63]. As shown in Table 3, the combinations showed marked reductions in IC_50_ values compared to either agent alone.

Although the 1:2 and 2:1 ratios of **6e**–lapatinib showed increased potency, with IC_50_ = 18.05 μM and 12.05 μM, respectively, the 1:1 ratio demonstrated the greatest improvement in cytotoxicity, resulting in an IC_50_ of 10.35 μM. This represents a more than two-fold improvement relative to each agent alone. The interaction between **6e** and lapatinib was quantitively evaluated using the combination index (CI) value [64]. As summarized in Table 4, all CI values for the various **6e**–lapatinib combination ratios were below 1, indicating synergistic cytotoxic effects against MCF-7 cells. The results also showed a high dose-reduction index (DRI) within the combinations. The strongest synergy was observed at a 1:1 ratio, with a CI value of 0.456, followed by the 2:1 ratio (CI = 0.518). The 1:2 ratio demonstrated moderate synergy (CI = 0.816). Instead of simply additive cytotoxicity, this ratio-dependent synergy suggests cooperative mechanisms of action, with the highest interaction seen at the 1:1 ratio.

**Figure 10:**
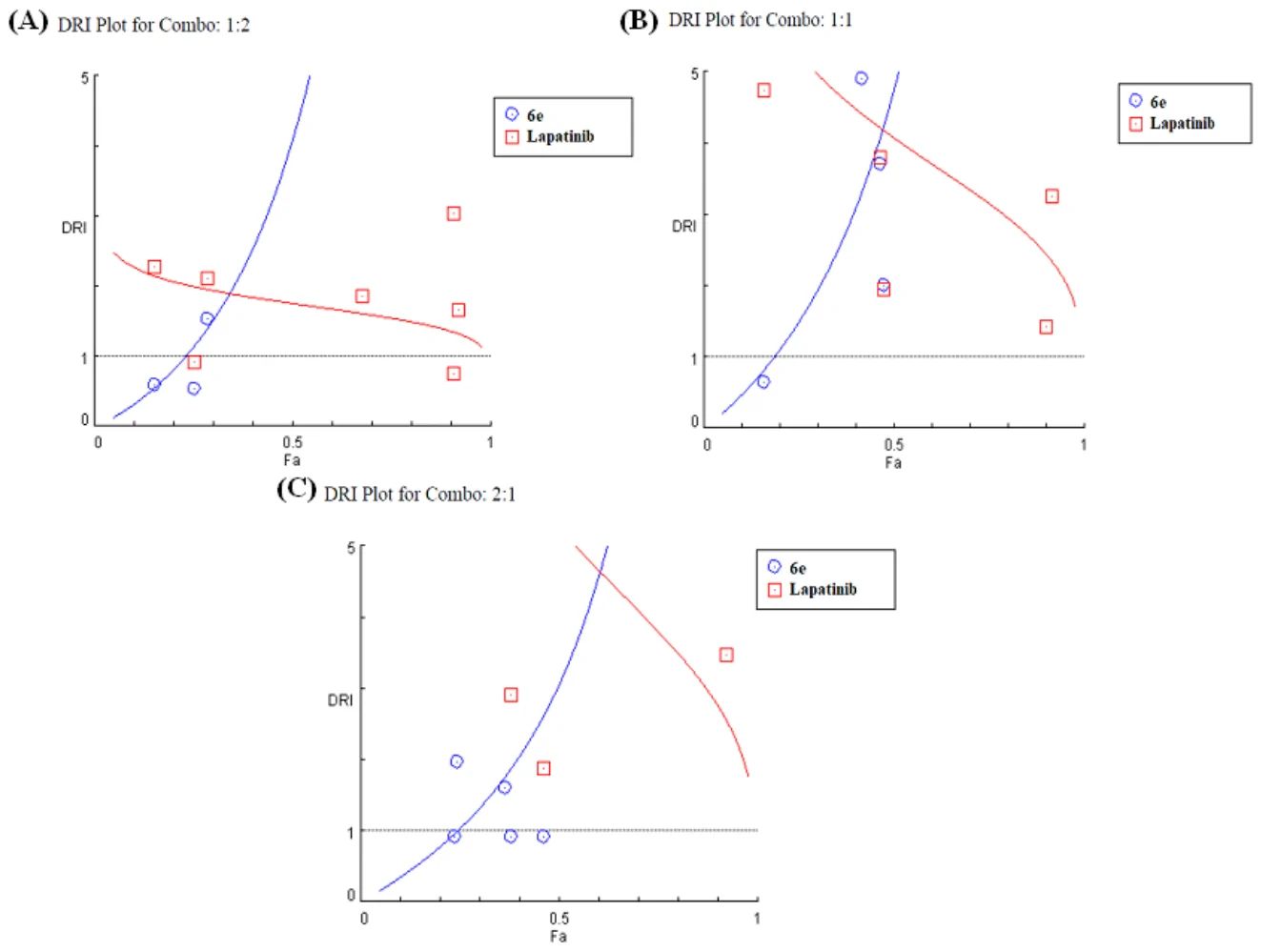
Dose-reduction indices of **6e** and lapatinib, respectively, across various ratios: (**A**) 1:2; (**B**) 1:1; (**C**) 2:1 on treated MCF-7 cells. The X-axis (Fa) indicates the percentage of cancer cell growth inhibition caused by the concentrations of the drug or tested compound.

On the other hand, the dose-reduction index (DRI) values indicate that substantially lower concentrations of **6e** and lapatinib were able to achieve equivalent antiproliferative effects when used in combination compared to single-agent treatment, minimizing dose-related side effects [65]. At the 1:1 combination ratio, the effective concentration of **6e** was reduced from 24.58 µM to 5.18 µM, while the concentration of lapatinib was reduced from 21.06 µM to 5.18 µM. Similar dose reductions were observed at the 1:2 and 2:1 ratios, indicating that lower doses of both agents were sufficient to achieve comparable antiproliferative effects when administered in combination. These synergistic anticancer effects were confirmed by a plot of DRI values at various fractions affected (Fa; MCF-7 growth inhibition % impacted by the employed quantities of lapatinib or the researched chemical **6e**) (Figure 10).

##### Chemoinvasion Assay

**Figure 11:**
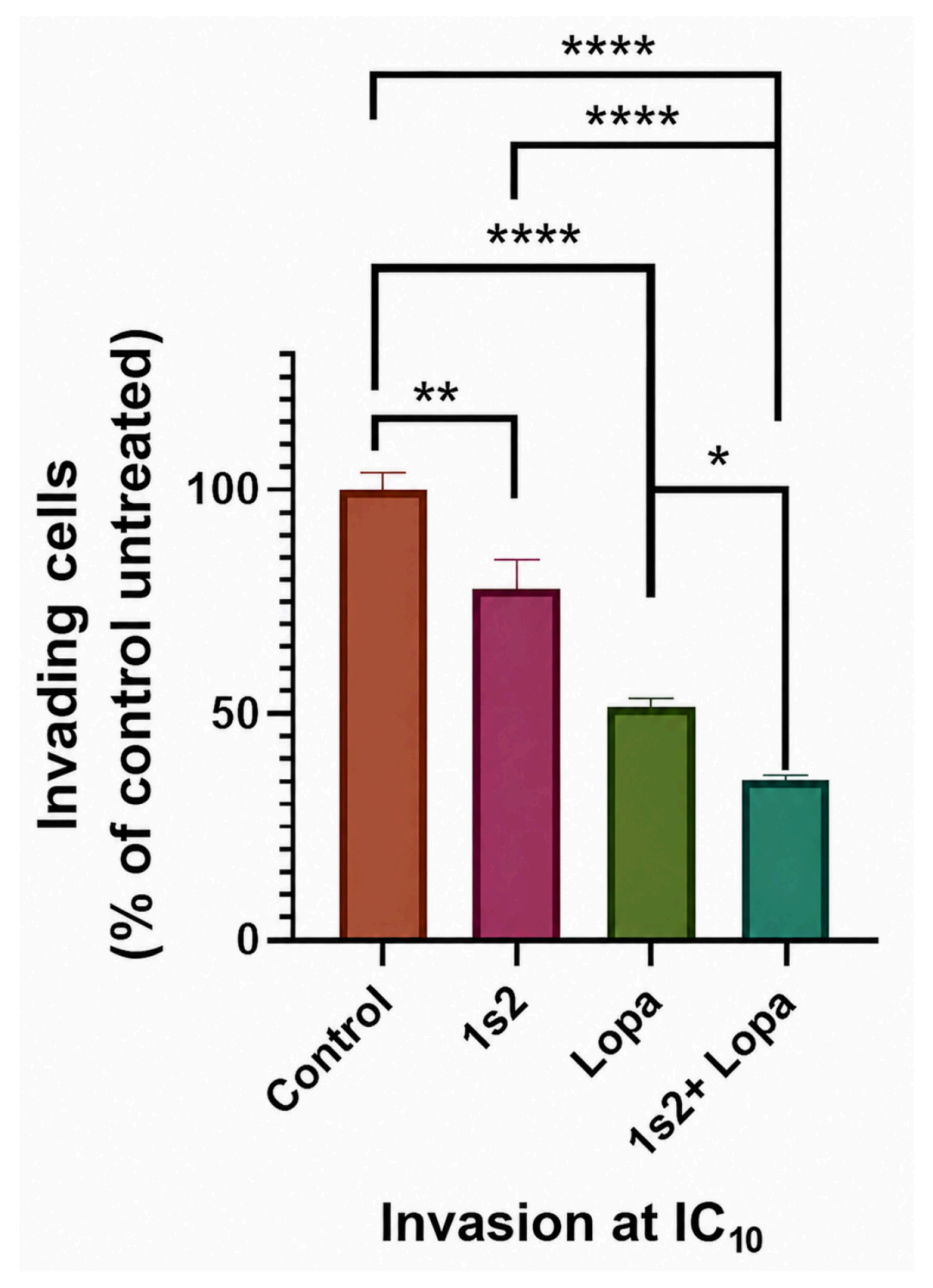
Quantification of MCF-7 invading cells. Invading cells were normalized to control untreated cells, and the percentage of invasion relative to the control was calculated for each treatment. One-way ANOVA with Tukey’s post hoc multiple comparison test was used for statistical analysis (*, *p* < 0.05; **, *p* < 0.01; ****, *p* < 0.0001). Data expressed as mean. Error bars represent SEM (*n* = 3).

A key factor influencing tumor growth and metastatic dissemination is cancer cell invasion. Consequently, a significant therapeutic benefit is the suppression of invasive activity at non-cytotoxic dosages [66]. The effects of **6e**, lapatinib, and their combination on the invasive behavior of MCF-7 cells were assessed using a chemoinvasion assay at IC_10_ concentrations to avoid cytotoxic interference [67,68]. As illustrated in Figure 11, lapatinib monotherapy had a greater inhibitory effect on cell invasion, while treatment with compound 6e alone led to a significant decrease in the number of invading cells, as evidenced by the significantly lower number of cells that invaded the basal side of the transwell and degraded collagen when compared to the untreated control. Moreover, the greatest reduction in invading cells per field was achieved when **6e** and lapatinib were administered combined. When compared to the control group and the individual treatments, this decrease was statistically significant, suggesting that the combination more successfully inhibited the invasive capability of MCF-7 cells than either agent alone. Compound **6e** dramatically reduced the number of invading cells to 77.91% of the control, representing a 22.09% drop in invasive ability. MCF-7 cells treated with lapatinib showed a more dramatic inhibition of invasion, with invasion decreased to 51.72% of control levels (48.28% reduction). The co-treatment with **6e** and lapatinib significantly inhibited MCF-7 cell invasion, with the highest identified anti-invasive impact, reducing cell invasion to 35.49% (64.51% reduction).

As previously discussed, when given separately at IC_10_ doses, **6e** and lapatinib both markedly decreased the invasive potential of MCF-7 cells. This finding suggests that the anti-invasive effects are a direct modification of invasion-related cellular processes rather than being solely a result of decreased cell viability [67]. Furthermore, when **6e** and lapatinib were administered in combination, cell invasion was significantly suppressed as opposed to when either drug was used alone. The idea that the two agents act in a complementary manner rather than through simple additive effects is supported by this enhanced anti-invasive activity, which is consistent with the synergistic antiproliferative interaction seen in the combination cytotoxicity studies. This suggests that this novel scaffold may influence additional molecular targets relevant to invasion control [69].

##### Annexin V/PI Flow-Cytometric Analysis

**Figure 12:**
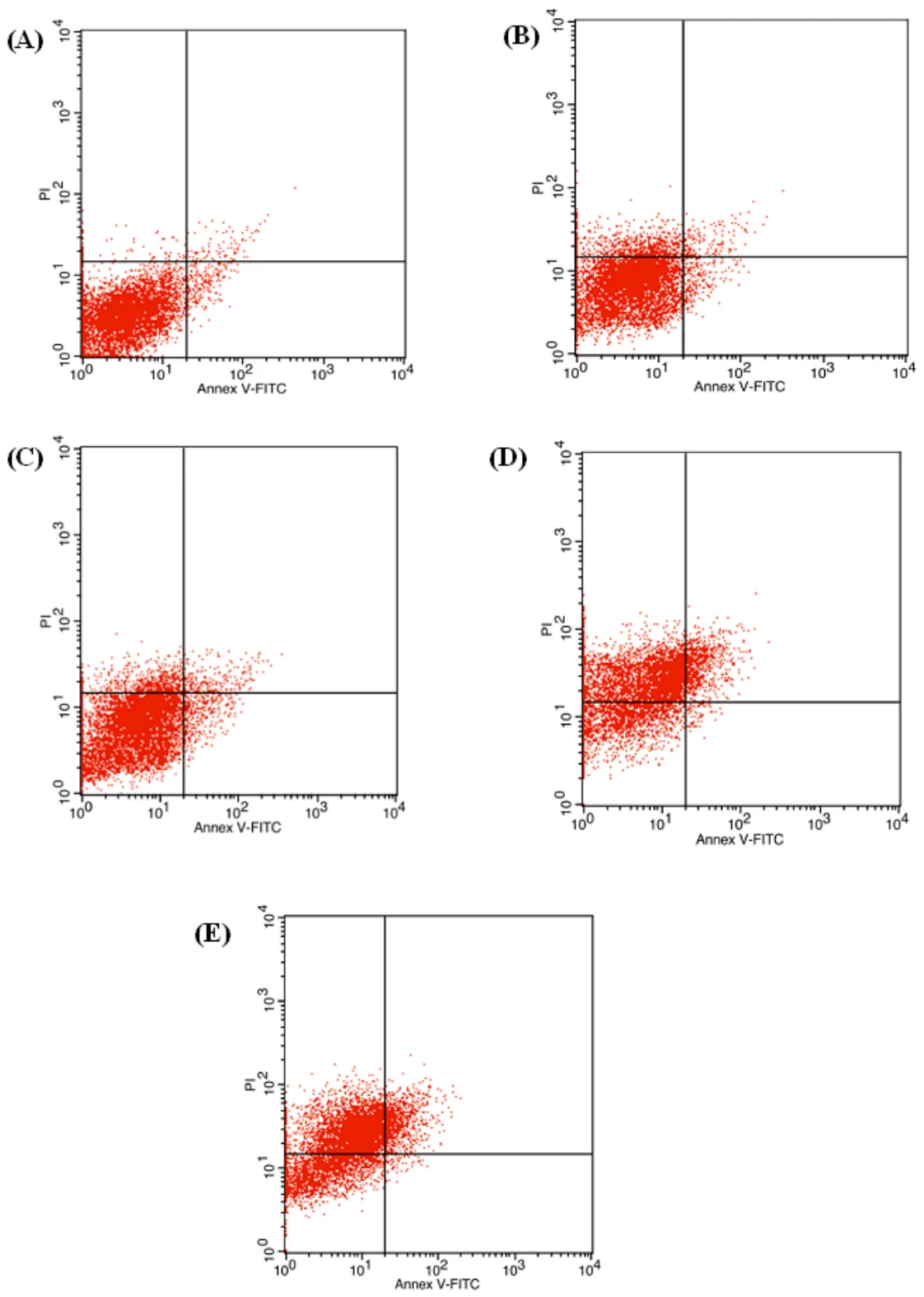
Annexin-V results for (**A**) MCF-7 control cells, (**B**) MCF-7 exposed to IC_50_ concentration of lapatinib, (**C**) MCF-7 exposed to IC_50_ concentration of **6e**, (**D**) MCF-7 exposed to IC_50_ concentration of the combination of lapatinib and **6e**, and (**E**) MCF-7 exposed to the reduced-concentration combination of lapatinib and **6e** (*n* = 2).

**Figure 13:**
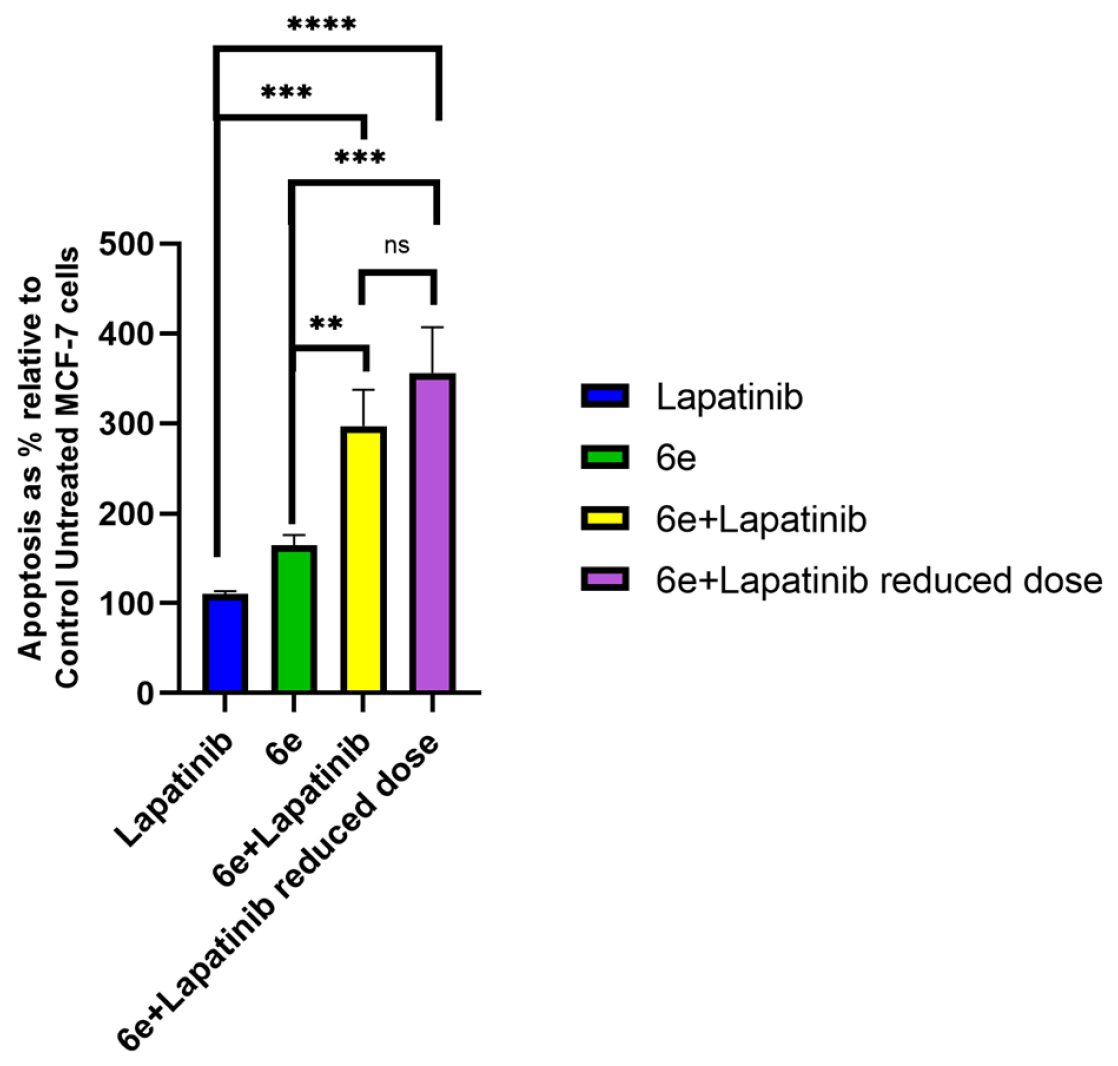
Effect of compound **6e**, lapatinib, and their combinations at either their IC_50_ levels or their reduced dose on apoptosis induction in MCF-7 cells after 24 h incubation. Apoptosis was measured using Annexin V-FITC/PI flow cytometry and reported as a percentage compared to untreated control cells. Statistical significance was determined by one-way analysis of variance followed by Tukey’s post hoc test, where significance levels were denoted as ** *p* < 0.01, *** *p* < 0.001 and **** *p* < 0.0001. Results are reported as means ± standard error of the mean (*n* = 2).

Flow-cytometric analysis using annexin V-FITC/PI staining was performed to evaluate the apoptotic effects of **6e**, lapatinib and their 1:1 combination on MCF-7 breast cancer cells. Cell populations were quantified as % of gated cells, which are classified into viable cells (Lower Left), early apoptotic (Lower Right), late apoptotic (Upper Right) and necrotic fractions (Upper Left). These results are shown in Figure 12 and Figure 13 [70]. Untreated control cells exhibited a predominantly viable population, accounting for 95.13% ± 0.247 of the total cell population, with minimal apoptosis (2.79% early apoptotic and 1.08% late apoptotic cells; total apoptosis 3.87% ± 0.106) and negligible necrosis (1.01% ± 0.141). Meanwhile, the treatment with lapatinib alone reduced the viable cell population to 84.52% ± 1.937 and slightly increased total apoptosis to 4.26% ± 0.021 (early apoptosis 2.59%, late apoptosis 1.67%) A noticeable increase in necrotic cells was observed (11.24% ± 1.237), indicating limited induction of programmed cell death under these conditions. However, treatment with **6e** alone resulted in a clearer apoptotic response. The viable cell fraction decreased to 85.80% ± 0.827, while total apoptosis increased to 6.38% ± 0.608, between 3.61% early apoptotic and 2.77% late apoptotic cells. Necrosis remained low (7.82% ± 0.226), suggesting that apoptotic pathways contributed significantly to cell death.

A pronounced shift in cell fate was remarkably observed upon combined treatment with **6e** and lapatinib at full dose (at their IC_50_ concentrations, 24.58 and 21.06 μM, respectively). The viable cell population dropped to 31.92% ± 0.014, accompanied by a substantial increase in late apoptotic cells (10.68%) and a total apoptotic fraction of 11.46% ± 1.237. This treatment also produced a high necrotic fraction (56.63%), reflecting extensive loss of cell viability. Simultaneously, the reduced-dose combination (5.18 μM of each compound) maintained strong apoptotic induction while slightly altering the distribution of cell death. Viable cells accounted for 34.17% ± 1.164, while total apoptosis reached 13.74% ± 1.583, consisting of 1.29% early apoptotic and 12.45% late apoptotic cells. Necrosis remained high (52.09%), but the apoptotic fraction was higher than that observed with the full-dose combination (Figure 8).

When comparing the previous results, combining **6e** and lapatinib resulted in a significant shift in cell fate, as seen by a significant drop in viable cells and a notable rise in apoptotic populations, particularly late apoptotic cells. This trend suggests irreversible apoptotic death rather than transient or early apoptotic signaling. Furthermore, the reduced-dose combination maintained a significant apoptotic response comparable to the full-dose combination, indicating that effective apoptosis induction can be obtained at lower drug concentrations when the two agents are administered together. As a result, it is acceptable to conclude that apoptosis contributes significantly to the anticancer activity of **6e** and lapatinib when used together.

##### Biomarker Analysis Using ELISA Technique

**Figure 14:**
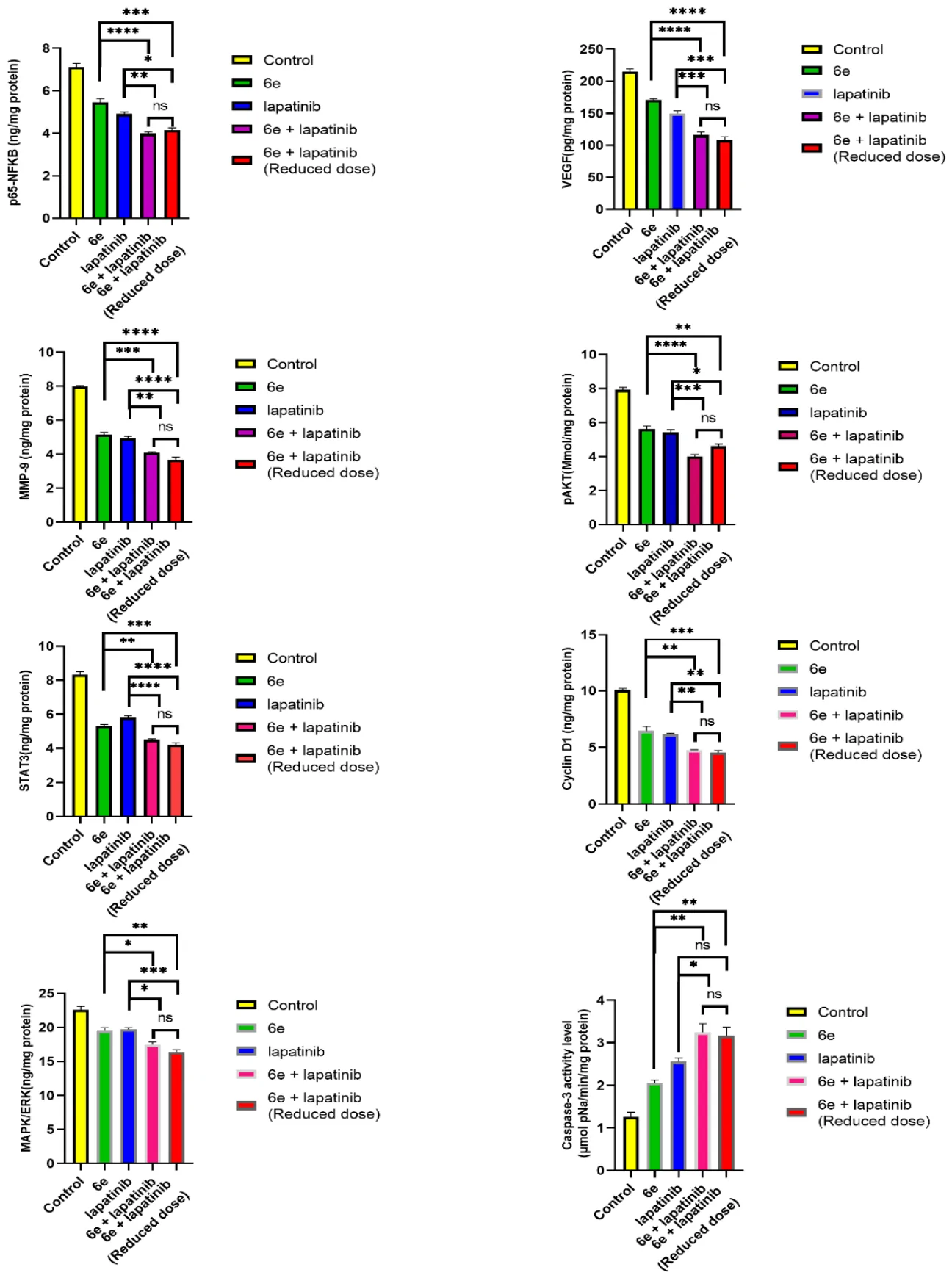
ELISA-based analysis of survival-, proliferation-, invasion-, and apoptosis-related biomarkers following treatment with **6e**, lapatinib, their combination and the reduced dose in MCF-7 cells. Statistical significance was determined by one-way analysis of variance followed by Tukey’s post hoc test, where significance levels are denoted as * *p* < 0.05, ** *p* < 0.01, *** *p* < 0.001, **** *p* < 0.0001 and ns is non-significant. Results are reported as means ± standard error of the mean (*n* = 3).

The use of ELISA-based profiling of key signaling biomarkers provided valuable insight into the cellular mechanisms underlying the anticancer activity of **6e**, both as a single agent and in combination with lapatinib. The effects of **6e**, lapatinib, and their combinations at either their IC_50_ levels (24.58 μM and 21.06 μM, respectively) or their reduced dose (5.18 μM of each compound) were evaluated on key cancer-related signaling biomarkers in MCF-7 cell lysates using ELISA. The measured biomarkers included NF-κB p65, VEGF, MMP9, phosphorylated AKT, STAT3, cyclin D1, p38 MAPK/ERK, and caspase-3 activity. All values were normalized to total protein content and expressed as mean ± SEM (Figure 14).

In untreated control cells, the measured biomarkers were NF-κB p65: 7.12 ± 0.17 ng/mg; VEGF: 214.87 ± 4.23 pg/mg; MMP9: 7.99 ± 0.05 ng/mg; p-AKT: 7.93 ± 0.14 mmol/mg protein; and p38 MAPK/ERK: 22.59 ± 0.51. Treatment with lapatinib alone led to a moderate decrease in survival- and invasion-related markers. NF-κB p65 dropped to 4.91 ± 0.09 ng/mg, VEGF to 149.80 ± 2.44 pg/mg, MMP9 to 4.93 ± 0.12 ng/mg, p-AKT to 5.43 ± 0.15 mmol/mg protein, and p38 MAPK/ERK to 19.71 ± 0.26 ng/mg. Treatment with **6e** alone resulted in noticeable downregulation of several oncogenic markers. NF-κB p65 levels dropped to 5.46 ± 0.17 ng/mg, VEGF to 170.72 ± 1.72 pg/mg, MMP9 to 5.16 ± 0.13 ng/mg, p-AKT to 5.63 ± 0.17 mmol/mg protein, and p38 MAPK/ERK to 19.50 ± 0.46 ng/mg. At the same time, STAT3 and cyclin D1 levels decreased from 8.33 ± 0.17 and 10.05 ± 0.14 ng/mg in control cells to 5.31 ± 0.09 and 6.46 ± 0.40 ng/mg in cells treated with **6e**, respectively.

It is interesting to note that all evaluated survival-, proliferation-, and invasion-related indicators were more significantly suppressed when **6e** and lapatinib were administered combined (at IC_50_ doses of 24.58 and 21.06 μM, respectively). NF-κB p65 dropped to 3.99 ± 0.08 ng/mg, VEGF to 115.75 ± 4.69 pg/mg, MMP9 to 4.09 ± 0.05 ng/mg, p-AKT to 4.01 ± 0.12 mmol/mg protein, and p38 MAPK/ERK to 17.51 ± 0.33 ng/mg. The levels of cyclin D1 and STAT3 were further reduced to 4.77 ± 0.04 and 4.50 ± 0.06 ng/mg, respectively. In addition, significant biomarker modulation was maintained by the reduced-dose combination (5.17 μM of each compound). NF-κB p65 (4.15 ± 0.11 ng/mg), VEGF (108.17 ± 5.43 pg/mg), MMP9 (3.69 ± 0.13 ng/mg), p-AKT (4.61 ± 0.13 mmol/mg protein), p38 MAPK/ERK (16.41 ± 0.32 ng/mg), STAT3 (4.20 ± 0.12 ng/mg), and cyclin D1 (4.57 ± 0.16 ng/mg) were all significantly decreased compared to the control cells.

After drug treatment, caspase-3 activity increased, in contrast to the downregulation of survival-associated markers. Lapatinib and **6e** individually raised caspase-3 levels to 2.56 ± 0.08 and 2.06 ± 0.06 μmol pNA/min/mg protein, respectively, while control cells had modest caspase-3 activity (1.26 ± 0.11 μmol pNA/min/mg protein). The maximum caspase-3 activation was produced by combination therapy (3.25 ± 0.20 for the full IC_50_ dose and 3.17 ± 0.20 μmol pNA/min/mg protein for the decreased dose), suggesting increased activation of apoptotic execution pathways.

All these results suggest that at the molecular level, treatment with **6e** resulted in coordinated suppression of several key signaling pathways that are essential to tumor cell survival, proliferation, invasion, and resistance. In particular, the decreased concentrations of phosphorylated AKT, NF-κB p65, and STAT3 indicate effective inhibition of interconnected survival and inflammatory signaling networks.

Beyond survival signaling, **6e** markedly reduced the concentration of cyclin D1, a critical regulator of G1/S cell-cycle progression that integrates mitogenic cues from ERK-, STAT3-, and NF-κB-dependent signaling. This reduction provides mechanistic insight into the antiproliferative activity observed in the cytotoxicity assays and suggests that **6e** interferes with cell-cycle control in addition to promoting cell death [71,72]. The ELISA results further demonstrated significant suppression of VEGF and MMP9, two key mediators of angiogenesis and extracellular matrix remodeling, respectively. These molecular changes translated into functional consequences, as evidenced by the chemoinvasion assay, where **6e** significantly reduced the invasive capacity of MCF-7 cells at sub-cytotoxic concentrations.

While induction of apoptosis was further confirmed by Annexin V/PI flow-cytometric analysis, the apoptotic response was accompanied by a significant increase in caspase-3 activity, as demonstrated by ELISA. This confirms the engagement of the apoptotic execution phase. Together, these findings establish apoptosis as a major contributor to the observed reduction in cell viability [73]. Overall, considering the baseline expression level of AXL in MCF-7 [74], **6e** is therefore viewed as a multitarget agent, rather than a sole in vitro AXL inhibitor, that deserves further evaluation on both the protein and cellular levels to identify the upstream effector(s) of the altered oncogenic pathways.

## 3. Materials and Methods

### 3.1. Chemistry

#### 3.1.1. Materials and Instrumentation

Solvents, reagents and instrumentation are detailed in the Supplementary Materials.

#### 3.1.2. Experimental Procedures

##### Preparation of 2-Azido-4,6-Dimethoxy-1,3,5-Triazine (**1**)

2-Azido-4,6-dimethoxy-1,3,5-triazine (ADT) was prepared by nucleophilic substitution of 2-chloro-4,6-dimethoxy-1,3,5-triazine (0.76 g, 4.3 mmol) with (0.31 g, 4.7 mmol) sodium azide, following the procedure reported in the literature. Full experimental details are provided in the Supplementary Materials [75].

##### General Procedure for the Preparation of 4-Substituted-*N*-(Prop-2-yn-1-yl) Benzamide (**2a**–**h**)

Amides **2a**–**h** were synthesized via the coupling reaction of the appropriate *p*-substituted benzoic acid (1.5 mmol) with propargylamine (130 μL, 2 mmol) using oxyma (0.284 g, 2 mmol), DIC (0.31 mL, 2 mmol), and DIPEA (0.35 mL, 2 mmol) in DMF (3 mL) at room temperature overnight. Full experimental details, isolated yields, melting points, and characterization data for compounds **2a**–**h** are provided in the Supplementary Materials [76,77,78,79,80,81,82].

##### General Procedure for the Preparation of 4-Substituted-N-((1-(4,6-Dimethoxy-1,3,5-triazin-2-yl)-1H-1,2,3-triazol-4-yl) methyl) Benzamide (**3a**–**h**)

2-azido-4,6-dimethoxy-1,3,5-triazine (0.153 g, 8.4 mmol) and 4-substituted-*N*-(prop-2-yn-1-yl) benzamide (7 mmol) were dissolved in 5 mL THF. Copper sulfate pentahydrate (0.038 g, 0.15 mmol) was dissolved in 1 mL water (following a stepwise Husigen cycloaddition reaction) and added to the reaction mixture, followed by an aqueous solution of ascorbic acid (0.073 g, 0.41 mmol) in 1 mL of water. The reaction mixture was stirred for 24 h at room temperature and monitored using TLC. THF was allowed to evaporate under reduced pressure, and precipitation occurred [83]. The formed precipitate was filtered, washed several times with distilled water and ether and allowed to dry. The products **3a**–**h** were obtained in good yield as fine amorphous powder.

- Compound **3a**: White solid; (0.25 g, 86.5% yield); m.p: 130 °C; IR (KBr) ṽ: 3318 (NH), 3138, 3102 (sp^2^ C–H), 2954 (sp^3^ C–H), 1656 (C=O), cm^−1^; ^1^H NMR (500 MHz, DMSO-*d*_6_) δ (ppm): 4.02 (s, 6H, 2OCH_3_), 4.58 (s, 2H, CH_2_), 7.45–7.55 (m, 2H, Ar-H), 7.80–7.95 (m, 2H, Ar-H), 8.70 (s, 1H, triazole *C*^5^-H), 9.17 (br. s, 1 NH, D_2_O exchangeable); ^13^C NMR (125 MHz, DMSO-*d*_6_) δ (ppm): 35.2, 56.45, 122.8, 129.0, 129.8, 133.2, 136.8, 146.5, 163.0, 165.8, 173.6; HRMS (ESI) Calcd. for C_15_H_14_ClN_7_O_3_ [M]^+^: 375.08467, [M+H]^+^: 376.09195, [M+K]^+^: 414.04783; Found: 376.09244 [M+H]^+^, 414.04870 [M+K]^+^.
- Compound **3b**: White solid; (0.23 g, 82.1% yield); m.p: 110 °C; IR (KBr) ṽ: 3322 (NH), 3142, 3105 (sp^2^ C–H), 2954 (sp^3^ C–H), 1655 (C=O), cm^−1^; ^1^H NMR (500 MHz, DMSO-*d*_6_) δ (ppm): 4.00 (s, 6H, 2OCH_3_), 4.56 (s, 2H, CH_2_), 7.24–7.25 (m, 2H, Ar-H), 7.80–7.95 (m, 2H, Ar-H), 8.67 (s, 1H, triazole *C*^5^-H), 9.13 (s, 1H, NH, D_2_O exchangeable); ^13^C NMR (125 MHz, DMSO-*d*_6_) δ (ppm): 35.1 56.5, (115.8 (d, C-C-F, ^2^*J* = 21.6 Hz)), 122.7, 130.5, 130.5, 146.5, 162.9, (164.5 (d, C-F, ^1^*J* = 247.5 Hz)), 166.1, 173.5; HRMS (ESI) Calcd. for C_15_H_14_FN_7_O_3_ [M]^+^: 359.11422, [M+H]^+^: 360.12150, [M+K]^+^: 398.07738; Found: 360.12218 [M+H]^+^, 398.07834 [M+K]^+^.
- Compound **3c**: White solid; (0.26 g,79.3% yield); m.p: 140 °C; IR (KBr) ṽ: 3311 (NH), 3137 (Ar-H), 3100 (sp^2^ C–H), 2953 (sp^3^ C–H), 1655 (C=O), cm^−1^; ^1^H NMR (500 MHz, DMSO-*d*_6_) δ (ppm): 4.01 (s, 6H, 2OCH_3_), 4.56 (d, 2H, ^3^*J* = 5.6, CH_2_), 7.63 (d, 2H, ^3^*J* = 8.1 Hz, Ar-H), 7.77 (d, 2H, ^3^*J* = 8.1 Hz, Ar-H), 8.69 (s, 1H, triazole *C*^5^-H), 9.17 (t, 1H, ^3^*J* = 5.6 Hz, NH, D_2_O exchangeable); ^13^C NMR (125 MHz, DMSO-*d*_6_) δ (ppm): 35.1, 56.5, 122.8, 125.7, 130.0, 131.9, 132.0, 133.6, 146.5, 163.0, 166.0, 173.6; HRMS (ESI) Calcd. for C_15_H_14_BrN_7_O_3_ [M]^+^: 419.03415, [M+H]^+^: 420.04143, [^81^Br, M+K]^+^: 459.99731; Found: 420.04197 [M+H]^+^, 459.99646 [^81^Br, M+K]^+^.
- Compound **3d**: White solid; (0.19 g, 69.4% yield);, m.p: 115 °C; IR (KBr) ṽ: 3326 (NH), 3139 (Ar-H), 3101 (sp^2^ C–H), 2955 (sp^3^ C–H), 1650 (C=O), cm^−1^; ^1^H NMR (500 MHz, DMSO-*d*_6_) δ (ppm): 2.29 (s, 3H, CH_3_), 4.01 (s, 6H, 2OCH_3_), 4.55 (s, 2H, CH_2_), 7.21 (d, 2H, ^3^*J* = 7.8 Hz, Ar-H), 7.73 (d, 2H, ^3^*J* = 7.8 Hz, Ar-H), 8.66 (s, 1H, triazole C^5^-H), 8.99 (br. s, 1H, NH, D_2_O exchangeable); ^13^C NMR (125 MHz, DMSO-*d*_6_) δ (ppm): 21.5, 35.0, 56.5, 122.7, 127.8, 129.5, 131.5, 146.7, 162.9, 173.5; HRMS (ESI) Calcd. for C_16_H_17_N_7_O_3_ [M]^+^: 355.13929, [M+H]^+^: 356.14657, [M+K]^+^: 394.10245; Found: 356.14735 [M+H]^+^, 394.10350 [M+K]^+^.
- Compound **3e**: White solid; (0.24 g, 83.9% yield), m.p: 180 °C; IR (KBr) ṽ: 3325 (NH), 3100 (Ar-H), 3013 (sp^2^ C–H), 2954, 2842 (sp^3^ C–H), 1650 (C=O), cm^−1^; ^1^H NMR (500 MHz, DMSO-*d*_6_) δ (ppm): 3.75 (s, 3H, OCH_3_), 4.01 (s, 6H, 2OCH_3_), 4.54 (d, 2H, ^3^*J* = 5.6 Hz, CH_2_), 6.94 (d, 2H, ^3^*J* = 8.8 Hz, Ar-H), 7.81 (d, 2H, ^3^*J* = 8.8 Hz, Ar-H), 8.65 (s, 1H, triazole *C*^5^-H), 8.94 (t, 1H, ^3^*J* = 5.6 Hz, NH, D_2_O exchangeable); ^13^C NMR (125 MHz, DMSO-*d*_6_) δ (ppm): 35.0, 55.9, 56.5, 114.1, 122.7, 126.6, 129.7, 146.9, 162.2, 163.0, 166.4, 173.7; HRMS (ESI) Calcd. for C_16_H_17_N_7_O_4_ [M]^+^: 371.13420, [M+K]^+^: 410.09736, [M+H]^+^: 372.14148; Found: 410.09803 [M+K]^+^, 372.14169 [M+H]^+^.
- Compound **3f**: White solid; (0.22 g, 73.9% yield), m.p: 200 °C; IR (KBr) ṽ: 3298 (NH), 3151 (Ar-H), 3109 (sp^2^ C–H), 2954 (sp^3^ C–H), 1667 (C=O), 1602, 1536 (NO_2_), cm^−1^; ^1^H NMR (500 MHz, DMSO-*d*_6_) δ (ppm): 4.02 (s, 6H, 2OCH_3_), 4.62 (d, 2H, ^3^*J* = 5.6 Hz, CH_2_), 8.05 (d, 2H, ^3^*J* = 8.7 Hz, Ar-H), 8.27 (d, 2H, ^3^*J* = 8.7 Hz, Ar-H), 8.74 (s, 1H, triazole *C*^5^-H), 9.42 (t, 1H, ^3^*J* = 5.6 Hz, NH, D_2_O exchangeable); ^13^C NMR (125 MHz, DMSO-*d*_6_) δ (ppm): 35.3, 56.5, 122.9, 124.1, 129.4, 140.1, 146.2, 149.6, 163.0, 165.3, 173.6; HRMS (ESI) Calcd. for C_15_H_14_N_8_O_5_ [M]^+^: 386.10872, [M+H]^+^: 387.11600, [M+K]^+^: 425.07188; Found: 387.11614 [M+H]^+^, 425.07255 [M+K]^+^.
- Compound **3g**: White solid; (0.27 g, 87.2% yield), m.p: 175 °C; IR (KBr) ṽ: 3316 (NH), 3131 (Ar-H), 3099 (sp^2^ C–H), 2985 (sp^3^ C–H), 1655 (C=O), cm^−1^; ^1^H NMR (500 MHz, DMSO-*d*_6_) δ (ppm): 4.02 (s, 6H, 2OCH_3_), 4.66 (s, 2H, CH_2_), 7.82–7.84 (m, 2H, Ar-H), 8.04–8.06 (m, 2H, Ar-H), 8.73 (s, 1H, triazole C^5^-H), 9.32 (s, 1H, NH, D_2_O exchangeable); ^13^C NMR (125 MHz, DMSO-*d*_6_) δ (ppm): 35.2, 56.5, 122.8, (123.7, 125.5, 125.9, 128.8 (q, C-F, ^1^*J* = 270 Hz)), 131.7, 132.0, 138.2 146.3, 163.0, 165.8, 173.6; HRMS (ESI) Calcd. for C_16_H_14_F_3_N_7_O_3_ [M]^+^: 410.11102, [M+H]^+^: 411.11830, [M+K]^+^: 449.07418; Found: 411.11472 [M+H]^+^, 449.06978 [M+K]^+^.
- Compound **3h**: White solid; (0.19 g, 69.4% yield), m.p: 85 °C; IR (KBr) ṽ: 3316 (NH), 3134 (Ar-H), 3096 (sp^2^ C–H), 2953 (sp^3^ C–H), 1650 (C=O), cm^−1^; ^1^H NMR (500 MHz, DMSO-*d*_6_) δ (ppm): 4.02 (s, 6H, 2OCH_3_), 4.58 (d, 2H, ^3^*J* = 5.6 Hz, CH_2_), 7.42–7.45 (m, 2H, Ar-H), 7.49–7.51 (m, 1H, Ar-H), 7.84 (d, 2H, ^3^*J =* 7.7 Hz, Ar-H), 8.69 (s, 1H, triazole C^5^-H), 9.10 (s, 1H, NH, D_2_O exchangeable); ^13^C NMR (125 MHz, DMSO-*d*_6_) δ (ppm): 35.1, 56.5, 122.7, 127.8, 129.0, 132.1, 134.3, 146.6, 162.9, 167.1, 173.6; HRMS (ESI) Calcd. for C_15_H_15_N_7_O_3_ [M]^+^: 341.12364, [M+H]^+^: 342.13092, [M+K]^+^: 380.08680; Found: 342.13165 [M+H]^+^, 380.08785 [M+K]^+^.

##### General Procedure for Preparation of p-Substituted Phenyl Isocyanates (**4e**–**h**)

Method A:

[CAUTION: PHOSGENE IS VOLATILE AND HIGHLY TOXIC—USE N95 MASK—USE HOOD]

A solution of TEA (0.5 mL, 3.6 mmol) in DCM (1 mL) was added to an ice-cooled solution of liquified phosgene in toluene (solution concentration 20% *v*/*v* − ρ = 0.94g/mL) (8.5 mL, 1.6 mmol) in a round-bottom flask equipped with a magnetic bar. A solution of p-substituted aniline (0.8 mmol) in DCM was added dropwise over 15 min. The reaction was allowed to stir for 1 h at 0 °C to obtain the corresponding *p*-substituted phenyl isocyanates **4e**–**g** (yield~70%).

Method B:

Amounts of 3 mmol of oxyma (0.426 g), 3 mmol DIC (0.464 mL), and 3 mmol DIPEA (0.523 mL) were added at room temperature to a solution of benzoic acid (2.0 mmol) in dry CH_2_Cl_2_ (5.0 mL), followed by the addition of 3 mmol NaN_3_ (0.39 g) in 2 mL DMSO. The reaction mixture was stirred for 30 min at room temperature to obtain the corresponding benzoyl azide. An amount of 10 mL of toluene was added to the reaction mixture, which was then refluxed for 6 h, leading to the formation of phenyl isocyanate **4h** (yield~62%) [51].

##### General Procedure for the Preparation of Substituted 1-(4-Substituted phenyl)-3-(prop-2-yn-1-yl) Urea (**5a**–**h**)

Method A: (using purchased p-substituted phenyl isocyanates)

4-Substituted phenyl isocyanate (2 mmol) was dissolved in 3 mL THF. An amount of 2.5 mmol of propargyl amine (0.16 mL) was added to the flask and left stirring for 2 h at room temperature. A white precipitate was formed. THF was allowed to evaporate, and the precipitate was filtered, washed several times with water and then hexane, and allowed to dry [84,85]. The products **5a**–**d** were obtained as fine amorphous powder.

Method B: (in situ after preparation of isocyanates from the corresponding amines;, Method A)

An amount of 3.2 mmol of propargyl amine (0.21 mL) was added to the flask with the reaction mixture containing the formed p-substituted isocyanate, and left stirring for 24 h at room temperature. The precipitated by-product salt was filtered off. DCM and toluene were allowed to evaporate, and the remaining residue was washed using 1N HCl to get rid of any excess amine, followed by washing with water and then hexane, and allowed to dry. The products **5e**–**g** were obtained as fine amorphous powder.

Method C: (in situ after preparation of isocyanate from the corresponding acids; Method B)

An amount of 4 mmol of propargyl amine (0.26 mL) was added to the flask with the reaction mixture containing the formed phenyl isocyanate, and left stirring for 24 h at room temperature. After total evaporation of the organic solvents, 5 mL of H_2_O was added to the reaction mixture and allowed to reflux for 30 min to remove any excess azide. The reaction mixture was allowed to cool down. The precipitated product was then filtered, washed with 1N HCl, water, and hexane and allowed to dry. The product **5h** was obtained as fine amorphous powder.

Full experimental procedures, isolated yields, melting points, and characterization data for compounds 5a–h are provided in the Supplementary Materials [84,85,86,87,88,89].

##### General Procedure for the Preparation of 1-(4-Substituted phenyl)-3-((1-(4,6-dimethoxy-1,3,5-triazin-2-yl)-1H-1,2,3-triazol-4-yl) methyl) Urea (**6a**–**h**)

2-Azido-4,6-dimethoxy-1,3,5-triazine (0.2185 g) and 1-(4-substituted phenyl)-3-(prop-2-yn-1-yl) urea (1 mmol) were dissolved in 5 mL THF. Copper sulfate pentahydrate (0.05 g) in water (1 mL) and ascorbic acid (0.1 g) in water (1 mL) were added (following a stepwise Husigen cycloaddition reaction). The reaction mixture was stirred for 24 h at room temperature and observed using TLC. THF was allowed to evaporate, and precipitation occurred [83]. The precipitate was filtered, washed several times with distilled water and then ether and allowed to dry. The products **6a**–**h** were obtained as fine amorphous powder.

- Compound **6a**: White solid (0.32 gm, 82% yield), m.p. 185 °C; IR (KBr) ṽ: 3345 (NH), 3146, 3106 (sp^2^ C–H), 2953 (sp^3^ C–H), 1689 (C=O), cm^−1^; ^1^H NMR (500 MHz, DMSO-*d*_6_) δ (ppm): 4.02 (s, 6H, 2OCH_3_), 4.40 (d, 2H, ^3^*J* = 5.7 Hz, CH_2_), 6.73 (distorted t, 1H, ^3^*J* = 5.7 Hz, NH, D_2_O exchangeable), 7.21 (d, 2H, ^3^*J* = 8.3 Hz, Ar-H), 7.38 (d, 2H, ^3^*J* = 8.3 Hz, Ar-H), 8.66 (s, 1H, triazole *C*^5^-H), 8.76 (s, 1H, NH, D_2_O exchangeable); ^13^C NMR (125 MHz, DMSO-*d*_6_) δ (ppm): 35.1, 56.5, 119.7, 122.3, 125.2, 129.0, 139.8, 147.3, 155.5, 163.0, 173.6; HRMS (ESI) Calcd. for C_15_H_15_ClN_8_O_3_ [M]^+^: 390.103385, [^35^Cl, M+H]^+^: 391.11066, [^37^Cl, M+H]^+^: 393.11066; Found: 391.10251 [^35^Cl, M+H]^+^, 393.10251 [^37^Cl, M+H]^+^.
- Compound **6b**: White solid (0.32 gm, 87.5% yield), m.p. 190 °C; IR (KBr) ṽ: 3349, 3298 (NH), 3151, 3070 (sp^2^ C–H), 2953 (sp^3^ C–H), 1683 (C=O) cm^−1^; ^1^H NMR (500 MHz, DMSO-*d*_6_) δ (ppm): 4.02 (s, 6H, 2OCH_3_), 4.39 (d, 2H, ^3^*J* = 5.6, CH_2_), 6.71 (br. s, 1H, NH, D_2_O exchangeable), 6.99 (t, 2H, *J* = 8.7 Hz, Ar-H), 7.34–7.37 (m, 2H, Ar-H), 8.65 (s, 1H, NH, D_2_O exchangeable), 8.67 (s, 1H, triazole C5-H); ^13^C NMR (125 MHz, DMSO-*d*_6_) δ (ppm): 35.1, 56.5, 118.4, 122.3, 129.6, 130.6, 138.2, 147.5, 155.7, 163.0, 173.6; HRMS (ESI) Calcd. for C_15_H_15_FN_8_O_3_ [M+H]^+^: 375.12511; Found: 375.13205 [M+H]^+^.
- Compound **6c**: White solid (0.39 gm, 89.8% yield), m.p. 215 °C; IR (KBr) ṽ: 3386, 3326 (NH), 3154, 3111 (sp^2^ C–H), 2955 (sp^3^ C–H), 1671 (C=O), cm^−1^; ^1^H NMR (500 MHz, DMSO-*d*_6_) δ (ppm): 4.03 (s, 6H, 2OCH_3_), 4.41 (s, 2H, CH_2_), 6.75 (s, 1H, NH, D_2_O exchangeable), 7.32–7.36 (m, 4H, Ar-H), 8.66 (s, 1H, triazole *C*^5^-H), 8.77 (s, 1H, NH, D_2_O exchangeable); ^13^C NMR (125 MHz, DMSO-*d*_6_) δ (ppm): 35.1, 56.5, 113.1, 120.2, 122.3, 131.9, 140.2, 147.3, 155.5, 163.0, 173.6; HRMS (ESI) Calcd. for C_15_H_15_BrN_8_O_3_ [M]^+^: 434.04505, [^79^Br, M+H]^+^: 435.05233, [^81^Br, M+H]^+^: 437.05233; Found: 435.05070 [^79^Br, M+H]^+^, 437.05070 [^81^Br, M+H]^+^.
- Compound **6d**: White solid (0.31 gm, 81% yield), m.p. 140 °C; IR (KBr) ṽ: 3316 (NH), 3134, 3024 (sp^2^ C–H), 2956 (sp^3^ C–H), 1605 (C=O), cm^−1^; ^1^H NMR (500 MHz, DMSO-*d*_6_) δ (ppm): 2.16 (s, 3H, CH_3_), 4.02 (s, 6H, 2OCH_3_), 4.39 (d, 2H, ^3^*J* = 5.8 Hz, CH_2_), 6.62 (dist. t, 1H, ^3^*J* = 5.8 Hz, NH, D_2_O exchangeable), 6.97 (d, 2H, ^3^*J* = 8.1 Hz, Ar-H), 7.22 (d, 2H, ^3^*J* = 8.1 Hz, Ar-H), 8.48 (s, 1H, triazole *C*^5^-H), 8.65 (s, 1H, NH, D_2_O exchangeable); ^13^C NMR (125 MHz, DMSO-*d*_6_) δ (ppm): 35.1, 56.5, 118.4, 122.3, 129.6, 130.5, 138.2, 147.5, 155.7, 163.0, 173.6; HRMS (ESI) Calcd. for C_15_H_15_N_9_O_5_ [M]^+^: 370.15019, [M+H]^+^: 371.15747; Found: 371.15748 [M+H]^+^.
- Compound **6e**: Buff solid (0.29 gm, 79% yield), m.p. 210 °C; IR (KBr) ṽ: 3314 (NH), 3192 (sp^2^ C–H), 2959 (sp^3^ C–H), 1644 (C=O), cm^−1^; ^1^H NMR (500 MHz, DMSO-*d*_6_) δ (ppm): 3.66 (s, 2H, CH_2_), 3.76 (s, 9H, 3 OCH_3_), 6.80 (d, 2H, ^3^*J* = 8.6 Hz, Ar-H), 7.28 (d, 2H, ^3^*J* = 8.6 Hz, Ar-H), 7.37 (br. s, 2H, NH, D_2_O exchangeable), 8.33 (s, 1H, triazole *C*^5^-H); ^13^C NMR (125 MHz, DMSO-*d*_6_) δ (ppm): 31.2, 54.6, 55.7, 114.5, 120.4, 133.4, 153.5, 154.8, 169.7, 172.6; Calcd. for C_16_H_18_N_8_O_4_ [M]^+^: 386.14510, [M+H]^+^: 387.15238; Found: 387.07165 [M+H]^+^. The purity of **6e** (~96.5%) was determined by HPLC analysis using a C_18_ reversed phase column (Eclipse XDB-C_18_ 4.6 mm × 150 mm, pore size 5 micro-analytical), with a linear gradient of 30–100% of ACN (0.1% TFA)/70–0% of H_2_O (0.1% TFA) over 15 min, flow rate: 1.0 mL.min^−1^, detection at 250 nm. The t_R_ value for **6e** was 5.4 min. The chromatogram and spectrum are provided in the Supplementary Materials.
- Compound **6f**: Yellow solid, (0.33 gm, 84% yield), m.p: 240 °C; IR (KBr) ṽ: 3305 (NH), 3154, 3054 (sp^2^ C–H), 2955 (sp^3^ C–H), 1706 (C=O), 1555, 1367 (NO_2_) cm^−1^; ^1^H NMR (500 MHz, DMSO-*d*_6_) δ (ppm): 4.02 (s, 6H, 2 OCH_3_), 4.43 (d, 2H, ^3^*J* = 5.7 Hz, CH_2_), 7.12 (t, 1H, ^3^*J* = 5.7 Hz, NH, D_2_O exchangeable), 7.58 (d, 2H, ^3^*J* = 8.9 Hz, Ar-H), 8.09 (d, 2H, ^3^*J* = 8.9 Hz, Ar-H), 8.68 (s, 1H, triazole *C*^5^-H), 9.54 (s, 1H, NH, D_2_O exchangeable); ^13^C NMR (125 MHz, DMSO-*d*_6_) δ (ppm): 35.2, 56.5, 117.5, 122.4, 125.7, 141.1, 146.9, 147.5, 155.0, 163.0, 173.6; HRMS (ESI) Calcd. for C_15_H_15_N_9_O_5_ [M]^+^: 401.11961, [M+H]^+^: 402.12689; Found: 402.12750 [M+H]^+^.
- Compound **6g**: Buff solid, (0.37 gm, 89% yield), m.p. 210 °C; IR (KBr) ṽ: 3347 (NH), 3174, 3061 (sp^2^ C–H), 2959 (sp^3^ C–H), 1697 (C=O), cm^−1^; ^1^H NMR (500 MHz, DMSO-*d*_6_) δ (ppm): 4.03 (s, 6H, 3OCH_3_), 4.42 (d, 2H, *J* = 5.6 Hz, CH_2_), 6.90 (t, 1H, ^3^*J* = 5.7 Hz, NH, D_2_O exchangeable), 7.52 (d, 2H, ^3^*J* = 8.8 Hz, Ar-H), 7.56 (d, 2H, ^3^*J* = 8.8 Hz, Ar-H), 8.68 (s, 1H, NH, D_2_O exchangeable), 9.19 (s, 1H, triazole *C*^5^-H); ^13^C NMR (125 MHz, DMSO-*d*_6_) δ (ppm): 35.1, 56.5, (117.6, 121.5, 122.4, 126.5, (q, C-F, ^1^*J* = 281 Hz)), 144.6, 147.2, 155.4, 163.0, 173.6; HRMS (ESI) Calcd. for C_16_H_15_ F_3_N_8_O_3_ [M]^+^: 424.12192, [M+K]^+^: 463.08508, [M+H]^+^: 425.12920; Found: 463.08571 [M+K]^+^, 425.12987 [M+H]^+^.
- Compound **6h**: Brown solid, (0.245 gm, 69% yield), m.p. 250 °C; IR (KBr) ṽ: 3395 (NH), 2958 (sp^3^ C–H), 1681 (C=O), cm^−1^; ^1^H NMR (500 MHz, DMSO-d_6_) δ (ppm): 4.02 (s, 6H, 3OCH_3_), 4.47 (d, 2H, ^3^*J* = 5.9 Hz, CH_2_), 6.14–6.16 (m, 1H, NH, D_2_O exchangeable), 6.93–7.18 (m, 4H, Ar-H), 8.08–8.10 (m, 1H, Ar-H), 8.68 (s, 1H, NH, D_2_O exchangeable), 9.32 (s, 1H, triazole C^5^-H); attempts to acquire the ^13^C-NMR spectrum were unsuccessful due to the compound’s poor solubility in common deuterated solvents, resulting in undetectable signals; HRMS (ESI) Calcd. for C_15_H_16_ N_8_O_3_ [M]^+^: 357.13454, [M+H]^+^: 358.14182; Found: 358.17055 [M+H]^+^.

### 3.2. Biological Evaluation

#### 3.2.1. Cytotoxicity Screening on Human Breast Cancer (MCF-7) and Normal Human Skin Fibroblasts

The anticancer efficacy of the compounds was evaluated using the MCF-7 human breast cancer cell line. Additionally, their cytotoxic effects on normal cells were tested on human skin fibroblast HSF cells. Cells were cultured, seeded, and incubated following the protocols detailed in the Supplementary Materials.

#### 3.2.2. Biochemical and Structural Assay of AXL

##### Biochemical Kinase Assay

To assess the ability of the synthesized active drug **6e** to interrupt the catalysis of AXL kinase, the ADP-Glo^TM^ Kinase Assay kit (Promega, Madison, WI, USA, CAT # V9101) was used, following the manufacturer’s instructions. AXL (Promega, CAT # V3961) kinase enzyme systems were procured from Promega (Madison, WI, USA). The ADP-Glo™ kinase assay is detailed in the Supplementary Materials.

##### Molecular Docking Analysis of **6e**

The crystal structure of AXL kinase (PDB ID: 5TC0) was retrieved from the Protein Data Bank and utilized for molecular docking studies. Protein preparation was carried out using the Protein Preparation Wizard implemented in Flare™ version 12.0.0 (Cresset, Litlington, Cambridgeshire, UK). The preparation procedure involved the addition of hydrogen atoms, removal of non-structural water molecules, assignment of protonation states for ionizable amino acid residues and ligand functional groups, optimization of hydrogen-bonding networks, and correction of protein structural inconsistencies. Subsequently, energy minimization was performed using the Flare energy minimization protocol employing Accurate XED (eXtended Electron Distribution) force field calculations.

The docking protocol was validated through re-docking of the co-crystallized ligand into the ATP-binding site of AXL kinase. The accuracy of the docking procedure was assessed by comparing the predicted binding pose with the experimentally determined crystallographic conformation using root-mean-square deviation (RMSD) analysis.

Molecular docking calculations were performed using the Lead Finder algorithm implemented in Flare™ 12.0.0 under the “Very Accurate but Slow” docking mode. The docking grid box was centered on the centroid of the co-crystallized ligand with a radius of 6.0 Å. Generated poses were ranked according to the Lead Finder scoring function, and the top-ranked binding poses were visually inspected and analyzed based on binding interactions, docking scores, and geometric compatibility within the active site. All molecular modeling calculations, analyses, and graphical representations were generated using Flare™ version 12.0.0 (Cresset^®^, Litlington, UK).

#### 3.2.3. Combination Studies of 6e with Lapatinib

A panel of in vitro assays was employed to characterize the activity of compound **6e** against MCF-7 cells, both as a single treatment and as an adjuvant to lapatinib therapy: an MTT assay for viability, analysis of combination and dose-reduction indices, and a chemoinvasion assay [68,90]. Images were analyzed using ImageJ software (version 1.54g) [29], annexin V/PI flow cytometry, and biomarker evaluation via ELISA [91].

## 4. Conclusions

To address the ongoing need for new therapeutic options in the breast cancer vicious cycle, particularly to augment targeted chemotherapy, innovative multitarget medicines have emerged as an effective approach to improving treatment outcomes. As a result, a variety of triazole–triazine hybrids with amide and ureido linkers were successfully developed and synthesized in this study, and their biological potential was thoroughly assessed. Compound **6e** emerged as the most active candidate among the produced derivatives, exhibiting considerable cytotoxic activity against the MCF-7 breast cancer cell line while maintaining reasonable selectivity for normal cells. Moreover, when combined with lapatinib, it resulted in an impressive synergistic increase in anticancer activity, indicating that this drug has potential in combination-based therapy strategies through minimizing the resistance mechanism against lapatinib. An in vitro enzymatic evaluation assay showed that compound **6e** inhibited AXL. Docking simulations presumed its possible binding mode within the enzymatic active site. On the other hand, cellular data revealed that the levels of VEGF, MMP9, cyclin D1, p-AKT, NF-κB, STAT3, and ERK were significantly decreased after treatment with **6e**, indicating its capacity to halt angiogenesis, invasion, and cell proliferation. The activation of apoptotic cell death was concurrently validated by a marked rise in caspase-3 activity, which was further validated by flow cytometry analysis and decreased chemo-invasive capacity.

These results demonstrate compound **6e**’s potential as a lead structure with an advanced mode of action against breast cancer and synergistic interaction with lapatinib. It is worth mentioning that based on the current study, our findings are limited to in vitro evaluation of the AXL inhibitory potential of **6e**. Its capacity to alter the aforementioned oncogenic pathways should not be interpreted herein as a downstream response to enzymatic inhibition. Accordingly, the mode of action of the hit compound **6e** should be further investigated on the cellular level in other cell lines overexpressing AXL and HER2. Furthermore, pharmacokinetic profiles and in vivo efficacy will be crucial to moving this scaffold closer to potentially beneficial medical applications.

## Figures and Tables

**Table 1:** Cytotoxicity screening of the synthesized triazine–triazole derivatives on HSF and MCF-7 expressed as IC_50_ values (µM) utilizing an MTT assay and their selectivity index values (SI).

Compd Name	IC_50_ on HSF (μM)	IC_50_ on MCF-7 (μM)	SI
**3a**	402.70 ± 1.209	195.90 ± 1.062	2.05
**3b**	560.60 ± 1.706	242.80 ± 1.292	2.30
**3c**	62.21 ± 1.056	58.35 ± 1.102	1.06
**3d**	223.30 ± 1.079	169.10 ± 1.159	1.32
**3e**	260.30 ± 1.211	119.20 ± 1.075	2.18
**3f**	278.20 ± 1.542	79.34 ± 1.092	3.50
**3g**	135.70 ± 1.040	44.57 ± 1.162	3.04
**3h**	354.40 ± 1.180	91.62 ± 1.087	3.86
**6a**	83.31 ± 1.051	125.70 ± 1.084	0.66
**6b**	180.20 ± 1.054	189.90 ± 1.142	0.94
**6c**	90.50 ± 1.075	83.02 ± 1.100	1.09
**6d**	144.80 ± 1.055	107.20 ± 1.021	1.35
**6e**	193.50 ± 1.251	24.58 ± 1.173	7.87
**6f**	956.10 ± 2.797	266.80 ± 1.999	3.58
**6g**	121.90 ± 1.088	189.50 ± 1.089	0.64
**6h**	N.D	>300	N.D

All data are presented as mean values with corresponding standard error of the mean (±SEM) (*n* = 3). IC_50_ refers to the concentration of the compound required to inhibit 50% of the target activity. The selectivity index (SI) is calculated as the ratio of the EC_50_ value in normal cells to the IC_50_ value in cancer cells. N.D indicates that the measurement was not determined.

**Table 2:** Biochemical inhibition of AXL kinase by 6e as determined be ADP-GloTM kinase assay expressed as % luminescence and % inhibition.

Drug	6e Dose (μM)	% Luminescence	% Inhibition
**6e**	0.5	124.66 + 2.58	−24.66
**6e**	2.5	85.74 + 4.07	14.26
**6e**	5	55.51 + 3.42	44.49
**6e**	10	34.21 + 0.77	65.79
Positive control (Bosutinib)	0.5	57.7 + 1.72	42.3

All values are presented as the mean ± standard error of the mean. (*n* = 2).

**Table 3:** The IC_50_ (µM) value of compound **6e** was measured both when used as a standalone anticancer agent and in combination with lapatinib against MCF-7 human breast cancer cells at various ratios following a 72 h incubation period (*n* = 2).

Drug/Combo	IC_50_
**6e**	24.59 ± 1.23
Lapatinib	21.06 ± 1.11
**6e** + Lapatinib (1:1)	10.35 ± 1.16
**6e** + Lapatinib (1:2)	18.05 ± 1.12
**6e** + Lapatinib (2:1)	12.05 ± 1.22

**Table 4:** Combination indices (CIs) and dose-reduction indices (DRIs) for **6e** in conjunction with lapatinib were evaluated against human breast cancer (MCF-7) cells across various ratios (*n* = 2).

Drug/Combo	CI Value	6e Dose (μM)	Lapatinib Dose (μM)	DRI of 6e	DRI of Lapatinib
**6e**	-	24.5850	-	-	-
Lapatinib	-	-	21.0647	-	-
**6e** + Lapatinib (1:1)	0.45638	5.17737	5.17737	4.74855	4.06861
**6e** + Lapatinib (1:2)	0.81588	6.01586	12.0317	4.08669	1.75076
**6e** + Lapatinib (2:1)	0.51764	8.03650	4.01825	3.05916	5.24225

## Data Availability

The original contributions presented in this study are included in the article/Supplementary Materials. Further inquiries can be directed to the corresponding authors.

## Supplementary Materials

The following supporting information can be downloaded at https://www.mdpi.com/article/10.3390/ph19101656/s1: Table S1: Complete dose-response inhibition data and replicate measurements for compound **6e** against AXL kinase (*n* = 2); Figure S1: Dose-Response curve of the synthesized triazine-triazole derivatives on MCF-7 cell line; Figure S2: Dose-Response curve of the synthesized triazine-triazole derivatives on HSF cell line; Figure S3: Combination Index (CI) plot showing the relationship between the fraction affected (*Fa*) and the combination index (*CI*) across fixed combination ratios of 1:1 (blue circles), 1:2 (red squares), and 2:1 (green triangles). Points falling below the reference line (*CI* = 1) indicate synergism; Figure S4: Logarithmic Combination Index plot displaying log(*CI*) versus the fraction affected (*Fa*) for the same fixed ratios (1:1, 1:2, and 2:1), generated using CompuSyn software; Figure S5: Effect of 6e on MCF-7 cells invasion as single agent and in combination with Lapatinib compared to untreated control. The invaded MCF-7 cells observed under inverted microscope using 10X objective lens; Figure S6: HPLC chromatogram of compound 6e measured at a detection wavelength of 250 nm; Figure S7: HPLC Area Percent Report for compound **6e** showing peak retention times, peak widths, peak areas, peak heights, and area percentages (indicating a purity of 96.25% for the main peak at retention time 5.444 min); Figure S8: 3D UV-absorbance spectrum/PDA three-dimensional chromatogram profile of compound **6e**, illustrating chromatographic separation across retention time and wavelength dimensions; Figure S9: UV spectrum of compound **6e** recorded via PDA detector, showing absorption peaks primarily in the UV region (250 nm); Figure S10: IR (KBr) spectrum of 4-chloro-*N*-((1-(4,6-dimethoxy-1,3,5-triazin-2-yl)-1*H*-1,2,3-triazol-4-yl) methyl) benzamide (3a); Figure S11: ^1^H-NMR (500 MHz, DMSO-*d_6_*) spectrum of 4-chloro-*N*-((1-(4,6-dimethoxy-1,3,5-triazin-2-yl)-1*H*-1,2,3-triazol-4-yl) methyl) benzamide (3a); Figure S12: ^1^H-NMR (500 MHz, DMSO-*d_6_* D_2_O) spectrum of 4-chloro-*N*-((1-(4,6-dimethoxy-1,3,5-triazin-2-yl)-1*H*-1,2,3-triazol-4-yl) methyl) benzamide (3a); Figure S13: ^13^C-NMR (DMSO-*d_6_*) spectrum of 4-chloro-*N*-((1-(4,6-dimethoxy-1,3,5-triazin-2-yl)-1*H*-1,2,3-triazol-4-yl) methyl) benzamide (3a); Figure S14: HRMS of 4-chloro-*N*-((1-(4,6-dimethoxy-1,3,5-triazin-2-yl)-1*H*-1,2,3-triazol-4-yl) methyl) benzamide (3a); Figure S15: IR (KBr) spectrum of *N*-((1-(4,6-dimethoxy-1,3,5-triazin-2-yl)-1*H*-1,2,3-triazol-4-yl) methyl)-4-fluorobenzamide (3b); Figure S16: ^1^H-NMR (500 MHz, DMSO-*d_6_*) spectrum of *N*-((1-(4,6-dimethoxy-1,3,5-triazin-2-yl)-1*H*-1,2,3-triazol-4-yl) methyl)-4-fluorobenzamide (3b); Figure S17: ^1^H-NMR (500 MHz, DMSO-*d_6_* D_2_O) spectrum of *N*-((1-(4,6-dimethoxy-1,3,5-triazin-2-yl)-1*H*-1,2,3-triazol-4-yl) methyl)-4-fluorobenzamide (3b); Figure S18: ^13^C-NMR (DMSO-*d_6_*) spectrum of *N*-((1-(4,6-dimethoxy-1,3,5-triazin-2-yl)-1*H*-1,2,3-triazol-4-yl) methyl)-4-fluorobenzamide (3b); Figure S19: HRMS of *N*-((1-(4,6-dimethoxy-1,3,5-triazin-2-yl)-1*H*-1,2,3-triazol-4-yl) methyl)-4-fluorobenzamide (3b); Figure S20: IR (KBr) spectrum of 4-bromo-*N*-((1-(4,6-dimethoxy-1,3,5-triazin-2-yl)-1*H*-1,2,3-triazol-4-yl) methyl) benzamide (3c); Figure S21: ^1^H-NMR (500 MHz, DMSO-*d_6_*) spectrum of 4-bromo-*N*-((1-(4,6-dimethoxy-1,3,5-triazin-2-yl)-1*H*-1,2,3-triazol-4-yl) methyl) benzamide (3c); Figure S22: ^1^H-NMR (500 MHz, DMSO-*d_6_*) spectrum of 4-bromo-*N*-((1-(4,6-dimethoxy-1,3,5-triazin-2-yl)-1*H*-1,2,3-triazol-4-yl) methyl) benzamide (3c); Figure S23: ^1^H-NMR (500 MHz, DMSO-*d_6_*) spectrum of 4-bromo-*N*-((1-(4,6-dimethoxy-1,3,5-triazin-2-yl)-1*H*-1,2,3-triazol-4-yl) methyl) benzamide (3c); Figure S24: ^1^H-NMR (500 MHz, DMSO-*d_6_*, D_2_O) spectrum of 4-bromo-*N*-((1-(4,6-dimethoxy-1,3,5-triazin-2-yl)-1*H*-1,2,3-triazol-4-yl) methyl) benzamide (3c); Figure S25: ^13^C-NMR (DMSO-*d_6_*) spectrum of 4-bromo-*N*-((1-(4,6-dimethoxy-1,3,5-triazin-2-yl)-1*H*-1,2,3-triazol-4-yl) methyl) benzamide (3c); Figure S26: HRMS of 4-bromo-*N*-((1-(4,6-dimethoxy-1,3,5-triazin-2-yl)-1*H*-1,2,3-triazol-4-yl) methyl) benzamide (3c); Figure S27: IR (KBr) spectrum of *N*-((1-(4,6-dimethoxy-1,3,5-triazin-2-yl)-1*H*-1,2,3-triazol-4-yl) methyl)-4-methylbenzamide (3d); Figure S28: ^1^H-NMR (500 MHz, DMSO-*d_6_*) spectrum of *N*-((1-(4,6-dimethoxy-1,3,5-triazin-2-yl)-1*H*-1,2,3-triazol-4-yl) methyl)-4-methylbenzamide (3d); Figure S29: ^1^H-NMR (500 MHz, DMSO-*d_6_*) spectrum of *N*-((1-(4,6-dimethoxy-1,3,5-triazin-2-yl)-1*H*-1,2,3-triazol-4-yl) methyl)-4-methylbenzamide (3d); Figure S30: ^1^H-NMR (500 MHz, DMSO-*d_6_*, D_2_O) spectrum of *N*-((1-(4,6-dimethoxy-1,3,5-triazin-2-yl)-1*H*-1,2,3-triazol-4-yl) methyl)-4-methylbenzamide (3d); Figure S31: ^13^C-NMR (DMSO-*d_6_*) spectrum of *N*-((1-(4,6-dimethoxy-1,3,5-triazin-2-yl)-1*H*-1,2,3-triazol-4-yl) methyl)-4-methylbenzamide (3d); Figure S32: HRMS of *N*-((1-(4,6-dimethoxy-1,3,5-triazin-2-yl)-1*H*-1,2,3-triazol-4-yl) methyl)-4-methylbenzamide (3d); Figure S33: IR (KBr) spectrum of *N*-((1-(4,6-dimethoxy-1,3,5-triazin-2-yl)-1*H*-1,2,3-triazol-4-yl) methyl)-4-methoxybenzamide (3e); Figure S34: ^1^H-NMR (500 MHz, DMSO-*d_6_*) spectrum of *N*-((1-(4,6-dimethoxy-1,3,5-triazin-2-yl)-1*H*-1,2,3-triazol-4-yl) methyl)-4-methoxybenzamide (3e); Figure S35: ^1^H-NMR (500 MHz, DMSO-*d_6_*) spectrum of *N*-((1-(4,6-dimethoxy-1,3,5-triazin-2-yl)-1*H*-1,2,3-triazol-4-yl) methyl)-4-methoxybenzamide (3e); Figure S36: ^1^H-NMR (500 MHz, DMSO-*d_6_*) spectrum of *N*-((1-(4,6-dimethoxy-1,3,5-triazin-2-yl)-1*H*-1,2,3-triazol-4-yl) methyl)-4-methoxybenzamide (3e); Figure S37: ^1^H-NMR (500 MHz, DMSO-*d_6_*, D_2_O) spectrum of *N*-((1-(4,6-dimethoxy-1,3,5-triazin-2-yl)-1*H*-1,2,3-triazol-4-yl) methyl)-4-methoxybenzamide (3e); Figure S38: ^13^C-NMR (DMSO-*d_6_*) spectrum of *N*-((1-(4,6-dimethoxy-1,3,5-triazin-2-yl)-1*H*-1,2,3-triazol-4-yl) methyl)-4-methoxybenzamide (3e); Figure S39: HRMS of *N*-((1-(4,6-dimethoxy-1,3,5-triazin-2-yl)-1*H*-1,2,3-triazol-4-yl) methyl)-4-methoxybenzamide (3e); Figure S40: IR (KBr) spectrum of *N*-((1-(4,6-dimethoxy-1,3,5-triazin-2-yl)-1*H*-1,2,3-triazol-4-yl) methyl)-4-nitrobenzamide (3f); Figure S41: ^1^H-NMR (500 MHz, DMSO-*d_6_*) spectrum of *N*-((1-(4,6-dimethoxy-1,3,5-triazin-2-yl)-1*H*-1,2,3-triazol-4-yl) methyl)-4-nitrobenzamide (3f); Figure S42: ^1^H-NMR (500 MHz, DMSO-*d_6_*) spectrum of *N*-((1-(4,6-dimethoxy-1,3,5-triazin-2-yl)-1*H*-1,2,3-triazol-4-yl) methyl)-4-nitrobenzamide (3f); Figure S43: ^1^H-NMR (500 MHz, DMSO-*d_6_*) spectrum of *N*-((1-(4,6-dimethoxy-1,3,5-triazin-2-yl)-1*H*-1,2,3-triazol-4-yl) methyl)-4-nitrobenzamide (3f); Figure S44: ^1^H-NMR (500 MHz, DMSO-*d_6_*) spectrum of *N*-((1-(4,6-dimethoxy-1,3,5-triazin-2-yl)-1*H*-1,2,3-triazol-4-yl) methyl)-4-nitrobenzamide (3f); Figure S45: ^1^H-NMR (500 MHz, DMSO-*d_6_*, D_2_O) spectrum of *N*-((1-(4,6-dimethoxy-1,3,5-triazin-2-yl)-1*H*-1,2,3-triazol-4-yl) methyl)-4-nitrobenzamide (3f); Figure S46: ^13^C-NMR (DMSO-*d_6_*) spectrum of *N*-((1-(4,6-dimethoxy-1,3,5-triazin-2-yl)-1*H*-1,2,3-triazol-4-yl) methyl)-4-nitrobenzamide (3f); Figure S47: HRMS of *N*-((1-(4,6-dimethoxy-1,3,5-triazin-2-yl)-1*H*-1,2,3-triazol-4-yl) methyl)-4-nitrobenzamide (3f); Figure S48: IR (KBr) spectrum of *N*-((1-(4,6-dimethoxy-1,3,5-triazin-2-yl)-1*H*-1,2,3-triazol-4-yl) methyl)-4-(trifluoromethyl) benzamide (3g); Figure S49: ^1^H-NMR (500 MHz, DMSO-*d_6_*) spectrum of *N*-((1-(4,6-dimethoxy-1,3,5-triazin-2-yl)-1*H*-1,2,3-triazol-4-yl) methyl)-4-(trifluoromethyl) benzamide (3g); Figure S50: ^1^H-NMR (500 MHz, DMSO-*d_6_*, D_2_O) spectrum of *N*-((1-(4,6-dimethoxy-1,3,5-triazin-2-yl)-1*H*-1,2,3-triazol-4-yl) methyl)-4-(trifluoromethyl) benzamide (3g); Figure S51: ^13^C-NMR (DMSO-*d_6_*) spectrum of *N*-((1-(4,6-dimethoxy-1,3,5-triazin-2-yl)-1*H*-1,2,3-triazol-4-yl) methyl)-4-(trifluoromethyl) benzamide (3g); Figure S52: HRMS of *N*-((1-(4,6-dimethoxy-1,3,5-triazin-2-yl)-1*H*-1,2,3-triazol-4-yl) methyl)-4-(trifluoromethyl) benzamide (3g); Figure S53: IR (KBr) spectrum of *N*-((1-(4,6-dimethoxy-1,3,5-triazin-2-yl)-1*H*-1,2,3-triazol-4-yl) methyl) benzamide (3h); Figure S54: ^1^H-NMR (500 MHz, DMSO-*d_6_*) spectrum of *N*-((1-(4,6-dimethoxy-1,3,5-triazin-2-yl)-1*H*-1,2,3-triazol-4-yl) methyl) benzamide (3h); Figure S55: ^1^H-NMR (500 MHz, DMSO-*d_6_*) spectrum of *N*-((1-(4,6-dimethoxy-1,3,5-triazin-2-yl)-1*H*-1,2,3-triazol-4-yl) methyl) benzamide (3h); Figure S56: ^1^H-NMR (500 MHz, DMSO-*d_6_*) spectrum of *N*-((1-(4,6-dimethoxy-1,3,5-triazin-2-yl)-1*H*-1,2,3-triazol-4-yl) methyl) benzamide (3h); Figure S57: ^13^C-NMR (DMSO-*d_6_*) spectrum of *N*-((1-(4,6-dimethoxy-1,3,5-triazin-2-yl)-1*H*-1,2,3-triazol-4-yl) methyl) benzamide (3h); Figure S58: HRMS of *N*-((1-(4,6-dimethoxy-1,3,5-triazin-2-yl)-1*H*-1,2,3-triazol-4-yl) methyl) benzamide (3h); Figure S59: IR (KBr) spectrum of 1-(4-chlorophenyl)-3-((1-(4,6-dimethoxy-1,3,5-triazin-2-yl)-1*H*-1,2,3-triazol-4-yl) methyl) urea (6a); Figure S60: ^1^H-NMR (500 MHz, DMSO-*d_6_*) spectrum of 1-(4-chlorophenyl)-3-((1-(4,6-dimethoxy-1,3,5-triazin-2-yl)-1*H*-1,2,3-triazol-4-yl) methyl) urea (6a); Figure S61: ^1^H-NMR (500 MHz, DMSO-*d_6_*) spectrum of 1-(4-chlorophenyl)-3-((1-(4,6-dimethoxy-1,3,5-triazin-2-yl)-1*H*-1,2,3-triazol-4-yl) methyl) urea (6a); Figure S62: ^1^H-NMR (500 MHz, DMSO-*d_6_*) spectrum of 1-(4-chlorophenyl)-3-((1-(4,6-dimethoxy-1,3,5-triazin-2-yl)-1*H*-1,2,3-triazol-4-yl) methyl) urea (6a); Figure S63: ^1^H-NMR (500 MHz, DMSO-*d_6_*, D_2_O) spectrum of 1-(4-chlorophenyl)-3-((1-(4,6-dimethoxy-1,3,5-triazin-2-yl)-1*H*-1,2,3-triazol-4-yl) methyl) urea (6a); Figure S64: ^13^C-NMR (DMSO-*d_6_*) spectrum of 1-(4-chlorophenyl)-3-((1-(4,6-dimethoxy-1,3,5-triazin-2-yl)-1*H*-1,2,3-triazol-4-yl) methyl) urea (6a); Figure S65: HRMS of 1-(4-chlorophenyl)-3-((1-(4,6-dimethoxy-1,3,5-triazin-2-yl)-1*H*-1,2,3-triazol-4-yl) methyl) urea (6a); Figure S66: IR (KBr) spectrum of 1-((1-(4,6-dimethoxy-1,3,5-triazin-2-yl)-1*H*-1,2,3-triazol-4-yl) methyl)-3-(4-fluorophenyl) urea (6b); Figure S67: ^1^H-NMR (500 MHz, DMSO-*d_6_*) spectrum of 1-((1-(4,6-dimethoxy-1,3,5-triazin-2-yl)-1*H*-1,2,3-triazol-4-yl) methyl)-3-(4-fluorophenyl) urea (6b); Figure S68: ^1^H-NMR (500 MHz, DMSO-*d_6_*) spectrum of 1-((1-(4,6-dimethoxy-1,3,5-triazin-2-yl)-1*H*-1,2,3-triazol-4-yl) methyl)-3-(4-fluorophenyl) urea (6b); Figure S69: ^1^H-NMR (500 MHz, DMSO-*d_6_*) spectrum of 1-((1-(4,6-dimethoxy-1,3,5-triazin-2-yl)-1*H*-1,2,3-triazol-4-yl) methyl)-3-(4-fluorophenyl) urea (6b); Figure S70: ^1^H-NMR (500 MHz, DMSO-*d_6_*, D_2_O) spectrum of 1-((1-(4,6-dimethoxy-1,3,5-triazin-2-yl)-1*H*-1,2,3-triazol-4-yl) methyl)-3-(4-fluorophenyl) urea (6b); Figure S71: ^13^C-NMR (DMSO-*d_6_*) spectrum of 1-((1-(4,6-dimethoxy-1,3,5-triazin-2-yl)-1*H*-1,2,3-triazol-4-yl) methyl)-3-(4-fluorophenyl) urea (6b); Figure S72: HRMS of 1-((1-(4,6-dimethoxy-1,3,5-triazin-2-yl)-1*H*-1,2,3-triazol-4-yl) methyl)-3-(4-fluorophenyl) urea (6b); Figure S73: IR (KBr) spectrum of 1-(4-bromophenyl)-3-((1-(4,6-dimethoxy-1,3,5-triazin-2-yl)-1*H*-1,2,3-triazol-4-yl) methyl) urea (6c); Figure S74: ^1^H-NMR (500 MHz, DMSO-*d_6_*) spectrum of 1-(4-bromophenyl)-3-((1-(4,6-dimethoxy-1,3,5-triazin-2-yl)-1*H*-1,2,3-triazol-4-yl) methyl) urea (6c); Figure S75: ^1^H-NMR (500 MHz, DMSO-*d_6_*, D_2_O) spectrum of 1-(4-bromophenyl)-3-((1-(4,6-dimethoxy-1,3,5-triazin-2-yl)-1*H*-1,2,3-triazol-4-yl) methyl) urea (6c); Figure S76: ^13^C-NMR (DMSO-*d_6_*) spectrum of 1-(4-bromophenyl)-3-((1-(4,6-dimethoxy-1,3,5-triazin-2-yl)-1*H*-1,2,3-triazol-4-yl) methyl) urea (6c); Figure S77: HRMS of 1-(4-bromophenyl)-3-((1-(4,6-dimethoxy-1,3,5-triazin-2-yl)-1*H*-1,2,3-triazol-4-yl) methyl) urea (6c); Figure S78: IR (KBr) spectrum of 1-((1-(4,6-dimethoxy-1,3,5-triazin-2-yl)-1*H*-1,2,3-triazol-4-yl) methyl)-3-(p-tolyl) urea (6d); Figure S79: ^1^H-NMR (500 MHz, DMSO-*d_6_*) spectrum of 1-((1-(4,6-dimethoxy-1,3,5-triazin-2-yl)-1*H*-1,2,3-triazol-4-yl) methyl)-3-(p-tolyl) urea (6d); Figure S80: ^1^H-NMR (500 MHz, DMSO-*d_6_*) spectrum of 1-((1-(4,6-dimethoxy-1,3,5-triazin-2-yl)-1*H*-1,2,3-triazol-4-yl) methyl)-3-(p-tolyl) urea (6d); Figure S81: ^1^H-NMR (500 MHz, DMSO-*d_6_*) spectrum of 1-((1-(4,6-dimethoxy-1,3,5-triazin-2-yl)-1*H*-1,2,3-triazol-4-yl) methyl)-3-(p-tolyl) urea (6d); Figure S82: ^1^H-NMR (500 MHz, DMSO-*d_6_*, D_2_O) spectrum of 1-((1-(4,6-dimethoxy-1,3,5-triazin-2-yl)-1*H*-1,2,3-triazol-4-yl) methyl)-3-(p-tolyl) urea (6d); Figure S83: ^13^C-NMR (DMSO-*d_6_*) spectrum of 1-((1-(4,6-dimethoxy-1,3,5-triazin-2-yl)-1*H*-1,2,3-triazol-4-yl) methyl)-3-(p-tolyl) urea (6d); Figure S84: HRMS of 1-((1-(4,6-dimethoxy-1,3,5-triazin-2-yl)-1*H*-1,2,3-triazol-4-yl) methyl)-3-(p-tolyl) urea (6d); Figure S85: IR (KBr) spectrum of 1-((1-(4,6-dimethoxy-1,3,5-triazin-2-yl)-1*H*-1,2,3-triazol-4-yl) methyl)-3-(4-methoxyphenyl) urea (6e); Figure S86: ^1^H-NMR (500 MHz, DMSO-*d_6_*) spectrum of 1-((1-(4,6-dimethoxy-1,3,5-triazin-2-yl)-1*H*-1,2,3-triazol-4-yl) methyl)-3-(4-methoxyphenyl) urea (6e); Figure S87: ^1^H-NMR (500 MHz, DMSO-*d_6_*) spectrum of 1-((1-(4,6-dimethoxy-1,3,5-triazin-2-yl)-1*H*-1,2,3-triazol-4-yl) methyl)-3-(4-methoxyphenyl) urea (6e); Figure S88: ^1^H-NMR (500 MHz, DMSO-*d_6_*, D_2_O) spectrum of 1-((1-(4,6-dimethoxy-1,3,5-triazin-2-yl)-1*H*-1,2,3-triazol-4-yl) methyl)-3-(4-methoxyphenyl) urea (6e); Figure S89: ^13^C-NMR (DMSO-*d_6_*) spectrum of 1-((1-(4,6-dimethoxy-1,3,5-triazin-2-yl)-1*H*-1,2,3-triazol-4-yl) methyl)-3-(4-methoxyphenyl) urea (6e); Figure S90: HRMS of 1-((1-(4,6-dimethoxy-1,3,5-triazin-2-yl)-1*H*-1,2,3-triazol-4-yl) methyl)-3-(4-methoxyphenyl) urea (6e); Figure S91: IR (KBr) spectrum of 1-((1-(4,6-dimethoxy-1,3,5-triazin-2-yl)-1*H*-1,2,3-triazol-4-yl) methyl)-3-(4-nitrophenyl) urea (6f); Figure S92: ^1^H-NMR (500 MHz, DMSO-*d_6_*) spectrum of 1-((1-(4,6-dimethoxy-1,3,5-triazin-2-yl)-1*H*-1,2,3-triazol-4-yl) methyl)-3-(4-nitrophenyl) urea (6f); Figure S93: ^1^H-NMR (500 MHz, DMSO-*d_6_*) spectrum of 1-((1-(4,6-dimethoxy-1,3,5-triazin-2-yl)-1*H*-1,2,3-triazol-4-yl) methyl)-3-(4-nitrophenyl) urea (6f); Figure S94: ^1^H-NMR (500 MHz, DMSO-*d_6_*) spectrum of 1-((1-(4,6-dimethoxy-1,3,5-triazin-2-yl)-1*H*-1,2,3-triazol-4-yl) methyl)-3-(4-nitrophenyl) urea (6f); Figure S95: ^1^H-NMR (500 MHz, DMSO-*d_6_*, D_2_O) spectrum of 1-((1-(4,6-dimethoxy-1,3,5-triazin-2-yl)-1*H*-1,2,3-triazol-4-yl) methyl)-3-(4-nitrophenyl) urea (6f); Figure S96: ^13^C-NMR (DMSO-*d_6_*) spectrum of 1-((1-(4,6-dimethoxy-1,3,5-triazin-2-yl)-1*H*-1,2,3-triazol-4-yl) methyl)-3-(4-nitrophenyl) urea (6f); Figure S97: HRMS of 1-((1-(4,6-dimethoxy-1,3,5-triazin-2-yl)-1*H*-1,2,3-triazol-4-yl) methyl)-3-(4-nitrophenyl) urea (6f); Figure S98: IR (KBr) spectrum of 1-((1-(4,6-dimethoxy-1,3,5-triazin-2-yl)-1*H*-1,2,3-triazol-4-yl) methyl)-3-(4-(trifluoromethyl) phenyl) urea (6g); Figure S99: ^1^H-NMR (500 MHz, DMSO-*d_6_*) spectrum of 1-((1-(4,6-dimethoxy-1,3,5-triazin-2-yl)-1*H*-1,2,3-triazol-4-yl) methyl)-3-(4-(trifluoromethyl) phenyl) urea (6g); Figure S100: ^1^H-NMR (500 MHz, DMSO-*d_6_*) spectrum of 1-((1-(4,6-dimethoxy-1,3,5-triazin-2-yl)-1*H*-1,2,3-triazol-4-yl) methyl)-3-(4-(trifluoromethyl) phenyl) urea (6g); Figure S101: ^1^H-NMR (500 MHz, DMSO-*d_6_*) spectrum of 1-((1-(4,6-dimethoxy-1,3,5-triazin-2-yl)-1*H*-1,2,3-triazol-4-yl) methyl)-3-(4-(trifluoromethyl) phenyl) urea (6g); Figure S102: ^13^C-NMR (DMSO-*d_6_*) spectrum of 1-((1-(4,6-dimethoxy-1,3,5-triazin-2-yl)-1*H*-1,2,3-triazol-4-yl) methyl)-3-(4-(trifluoromethyl) phenyl) urea (6g); Figure S103: HRMS of 1-((1-(4,6-dimethoxy-1,3,5-triazin-2-yl)-1*H*-1,2,3-triazol-4-yl) methyl)-3-(4-(trifluoromethyl) phenyl) urea (6g); Figure S104: IR (KBr) spectrum of 1-((1-(4,6-dimethoxy-1,3,5-triazin-2-yl)-1*H*-1,2,3-triazol-4-yl) methyl)-3-phenyl urea (6h); Figure S105: ^1^H-NMR (500 MHz, DMSO-*d_6_*) spectrum of 1-((1-(4,6-dimethoxy-1,3,5-triazin-2-yl)-1*H*-1,2,3-triazol-4-yl) methyl)-3-phenyl urea (6h); Figure S106: ^1^H-NMR (500 MHz, DMSO-*d_6_*) spectrum of 1-((1-(4,6-dimethoxy-1,3,5-triazin-2-yl)-1*H*-1,2,3-triazol-4-yl) methyl)-3-phenyl urea (6h); Figure S107: ^1^H-NMR (500 MHz, DMSO-*d_6_*) spectrum of 1-((1-(4,6-dimethoxy-1,3,5-triazin-2-yl)-1*H*-1,2,3-triazol-4-yl) methyl)-3-phenyl urea (6h); Figure S108: ^1^H-NMR (500 MHz, DMSO-*d_6_*) spectrum of 1-((1-(4,6-dimethoxy-1,3,5-triazin-2-yl)-1*H*-1,2,3-triazol-4-yl) methyl)-3-phenyl urea (6h); Figure S109: ^1^H-NMR (500 MHz, DMSO-*d_6_*) spectrum of 1-((1-(4,6-dimethoxy-1,3,5-triazin-2-yl)-1*H*-1,2,3-triazol-4-yl) methyl)-3-phenyl urea (6h).

## Abbreviations

The following abbreviations are used in this manuscript:

AKT	AKR Strain Transforming/Protein Kinase B
DIPEA	*N*,*N*′-Diisopropylethylamine
DCM	Dichloromethane
DMSO	Dimethyl Sulfoxide
DIC	*N*,*N*′-Diisopropylcarbodiimide
ERK	Extracellular signal-Regulated Kinase
ELISA	Enzyme-Linked ImmunoSorbent Assay
FDA	Food and Drug Administration
HER	Human Epidermal growth factor Receptor
MERTK	Myeloid Epithelial Reproductive Tyrosine Kinase
MAPK	Mitogen-Activated Protein Kinase
MCF-7	Michigan Cancer Foundation–7/breast cancer cell line
MH	Molecular Hybridization
MMP9	Matrix Metalloproteinase-9
MTT	3-(4,5-dimethylthiazol-2-yl)-2,5-diphenyltetrazolium bromide assay
NF-κB	Nuclear Factor kappa-light-chain-enhancer of activated B cells
PI3K	Phosphoinositide 3-Kinase
PDB	Protein Data Bank
RTK	Receptor Tyrosine Kinase
TYRO3	Tyrosine-protein Kinase 3
THF	Tetrahydrofuran
VEGF	Vascular Endothelial Growth Factor
V-FTIC/PI	Annexin V-Fluorescein Isothiocyanate (FITC)/Propidium Iodide (PI) assay
