# Supplementary material for: From Design to Synergy: Triazine–Triazole Hybrids as Anti-Breast Cancer Leads Targeting Survival and Invasion Pathways for Augmenting Lapatinib Efficacy
Source: Pharmaceuticals (Basel). 2026 Oct 20;19(10):1656. doi: 10.3390/ph19101656 (PMC13708434; doi:10.3390/ph19101656)
Supplement: Supplementary file 1 [file pharmaceuticals-19-01656-s001.zip › pharmaceuticals-4435080-supplementary.pdf]

From Design to Synergy: Triazine–Triazole Hybrids as Anti-Breast Cancer Leads Targeting Survival and Invasion Pathways for Augmenting Lapatinib Efficacy

Omnia M. Ali [1,2], Samar Hafez Abbas [1,3], Micheal G. Shehat [4], Maged W. Helmy [5,6], Moustafa H. Aly [7], Mohamed Teleb [8,9], Sherine N. Khattab [10,*] and Mohamed Abdel-Aziz [1,3,*]

[1] Department of Medicinal Chemistry, Faculty of Pharmacy, Minia University, Minia, Egypt

[2] Department of Medicinal Chemistry, College of Pharmacy, Arab Academy for Science, Technology and Maritime Transport, El-Alamein 1029, Egypt

[3] Medicinal Chemistry Department, Faculty of Pharmacy, Minia National University, New Minia, Egypt

[4] Department of Microbiology and Immunology, Faculty of Pharmacy, Alexandria University, Alexandria, Egypt

[5] Pharmacology and Toxicology Department, Faculty of Pharmacy, Damanhour University, Egypt

[6] Pharmacology and Toxicology Department, Faculty of Pharmacy, Damanhour National University, Egypt

[7] College of Engineering and Technology, Arab Academy for Science, Technology and Marine Transport, El-Alamein 1029, Egypt

[8] Department of Pharmaceutical Chemistry, Faculty of Pharmacy, Alexandria University, Alexandria 21521, Egypt

[9] Department of Medicinal Chemistry, Faculty of Pharmacy, Alamein International University (AIU), Alamein City, Alamein City 5060310, Egypt.

[10] Chemistry Department, Faculty of Science, Alexandria University, Alexandria 21321, Egypt.

* **Correspondence:** M. Abdel-Aziz: abulnil@hotmail.com, abulnil@mu.edu.eg

Sh. N. Khattab: sherinekhattab@alexu.edu.eg,

Results and Discussion

- ***Biological Evaluation:***
- ***Cytotoxicity screening on human breast cancer (MCF-7) and normal human skin fibroblasts:***

Experiments were conducted to evaluate compounds 3a-h and 6a-h cytotoxic activities against MCF-7 breast cancer cell line compared to lapatinib in terms of IC_{50} and to determine the safety profile of the studied compounds, their cytotoxic effects were evaluated on the human skin fibroblast cells (HSF) all via MTT assay (Figure S1, S2)

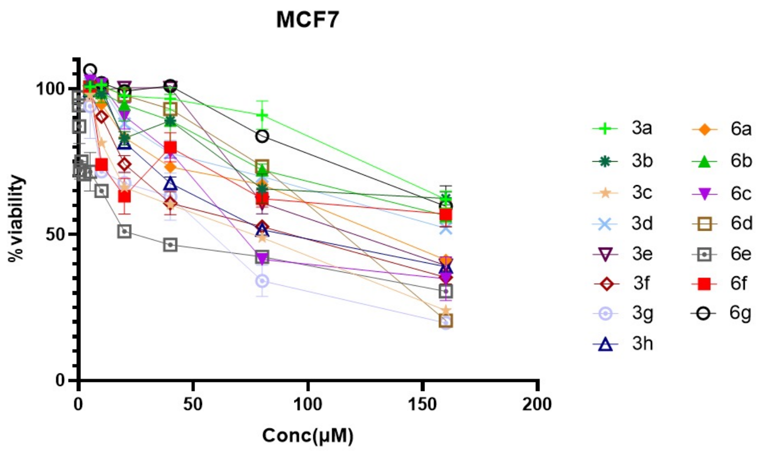

Figure S1: Dose-Response curve of the synthesized triazine-triazole derivatives on MCF-7 cell line.

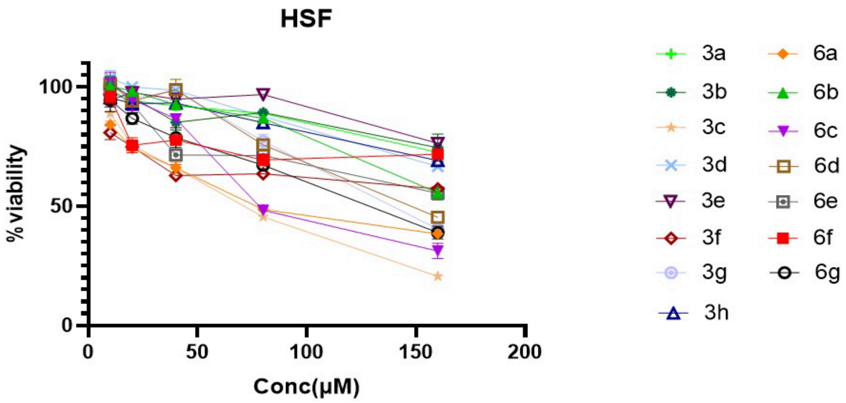

Figure S2: Dose-Response curve of the synthesized triazine-triazole derivatives on HSF cell line.

- **Biochemical kinase assay:**

Table S1. Complete dose-response inhibition data and replicate measurements for compound **6e** against AXL kinase ($n = 2$).

Dose (μM)	Exp. 1 (%)	Exp. 2 (%)	Mean Inhibition (%)	Standard Deviation (SD)	Standard Error of the Mean (SEM)
0.5	-24.66	-19.52	-22.09	3.63	2.57
2.5	14.26	22.41	18.34	5.76	4.08
5	44.49	37.65	41.07	4.84	3.42
10	65.79	67.34	66.57	1.09	0.77

- **Combination studies with Lapatinib:**

- **MTT assay and determination of combination and dose reducing indices:**

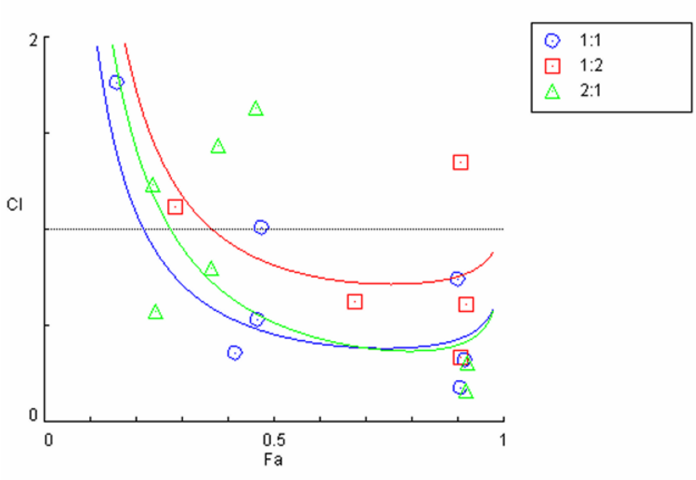

Figure S3: Combination Index (CI) plot showing the relationship between the fraction affected (Fa) and the combination index (CI) across fixed combination ratios of 1:1 (blue circles), 1:2 (red squares), and 2:1 (green triangles). Points falling below the reference line ($CI = 1$) indicate synergism.

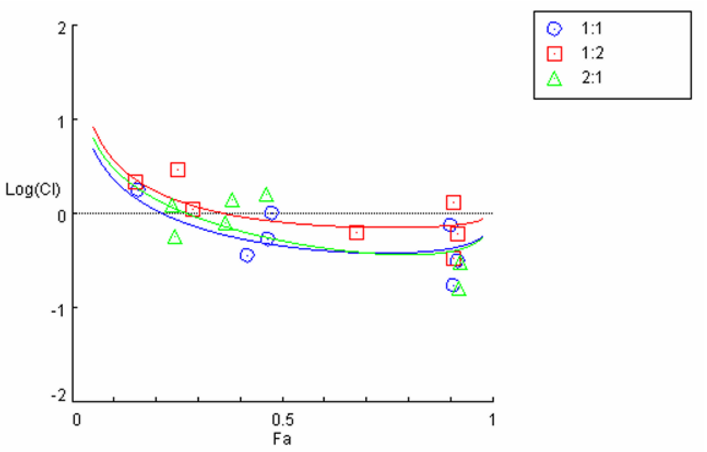

Figure S4: Logarithmic Combination Index plot displaying $\log(CI)$ versus the fraction affected (Fa) for the same fixed ratios (1:1, 1:2, and 2:1), generated using CompuSyn software.

- **Chemo-invasion assay:**

The effect of **6e**, lapatinib, and their combination on the invasive behavior of MCF-7 cells was assessed using a chemoinvasion assay at IC_{10} concentrations to avoid cytotoxic interference (Figure S5) [64, 65].

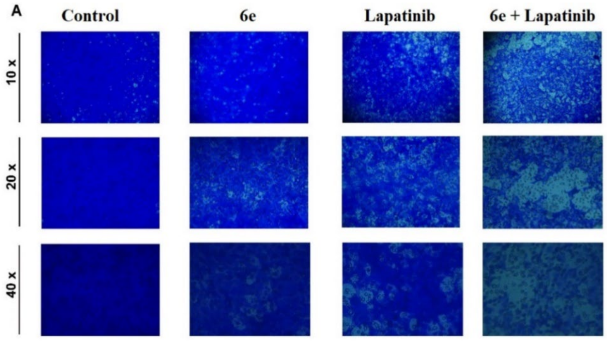

Figure S5: Effect of 6e on MCF-7 cells invasion as single agent and in combination with Lapatinib compared to untreated control. The invaded MCF-7 cells observed under inverted microscope using 10X objective lens.

Materials and Methods

- ***Chemistry:***

- ***Material and Instrumentation:***

Solvents and all reagents were afforded by Sigma-Aldrich. Unless otherwise stated, normal work-up from organic solvents involved drying over Na_2SO_4 and rotary evaporation. TLC was performed using aluminum-backed Merck Silica Gel 60 F-254 plates using suitable solvent systems with spots being visualized by a Spectroline UV Lamp, USA (254 or 365 nm) or I_2 vapor. Melting points were obtained in open capillary tubes by using a MEL-Temp II, USA melting point apparatus and are uncorrected.

Infrared spectra (IR) were recorded on a Brucker Tensor 37 FT-IR Fourier transform instrument, Germany, either neat (as film between NaCl plates) or as KBr pellets. The absorption bands ($\bar{\nu}_{max}$) are given in wave numbers (cm^{-1}). Nuclear magnetic resonance (NMR) spectra (^1H NMR and ^{13}C NMR) were recorded on a JEOL 500 MHz spectrophotometer, Japan, at an ambient temperature. Tetramethyl silane (TMS) was used as an internal reference for all ^1H-NMR spectra with chemical shifts reported as ppm relative to TMS. The data is reported as follows: chemical shifts, multiplicity (s = singlet, d = doublet, t = triplet, q = quadruplet, quint = quintuplet, m = multiplet, br. = broad, dd = doublet of doublet, td = triplet of doublet, etc.), integration, and interpretation. The HRMS were recorded on LC/Q-TOF, 6530 (Agilent Technologies, Santa Clara, CA, USA) at Faculty of pharmacy, Fayoum University.

- **Experimental Procedures:**
- ***Preparation of 2-azido-4,6-dimethoxy-1,3,5-triazine (1):***

A solution of 2-chloro-4,6-dimethoxy-1,3,5-triazine (0.76 g, 4.3 mmol) was prepared by dissolving the compound in 5 mL of acetonitrile within a round-bottom flask fitted with a magnetic stirrer. An aqueous solution of sodium azide (0.306 g (4.7 mmol) NaN_3 in 5 mL of H_2O) was added dropwise at room temperature with stirring. Then, the reaction mixture was stirred at 50 °C for 2.5 h. After cooling to ambient temperature, the organic solvent (acetonitrile) was removed under reduced pressure. Then, the mixture was extracted with DCM (3 times) and the combined organic extracts were dried over anhydrous Na_2SO_4, filtered, and concentrated under reduced pressure to afford ADT **1 (yield~88%)** as a white crystalline solid [75].

- ***General procedure for the preparation of 4-substituted-N-(prop-2-yn-1-yl) benzamide (2a-h):***

A solution of the appropriate *p*-substituted benzoic acid (1.5 mmol) in 3 mL DMF was introduced to a round-bottom flask. 2 mmol of oxyma (0.284 g), 2 mmol of DIC (0.31 mL) and 2 mmol of DIPEA (0.35 mL) were added to the flask and left stirring for 10 minutes for preactivation. 2 mmol of propargyl amine (130 µL, ρ. 0.86 g/mL) was added to the reaction mixture and left stirring overnight at room temperature. After 24 h, iced water was introduced to the reaction mixture leading to precipitation of the formed amide. The formed precipitate was filtered, washed several times with distilled water and diethyl ether and allowed to dry. The products **2a-h** were obtained as fine amorphous powder.

- ***4-Chloro-N-(prop-2-yn-1-yl) benzamide (2a)***

White solid; (0.261 g, 90.1% yield); m.p: 147 °C [145–146 °C] [76]

- ***4-Fluoro-N-(prop-2-yn-1-yl)benzamide (2b)***

White solid; (0.245 g, 92.5% yield); m.p: 130 °C [133 °C] [77].

- ***4-Bromo-N-(prop-2-yn-1-yl)benzamide (2c)***

White solid; (0.318 g, 89.7% yield); m.p: 161 °C [157 – 159 °C] [78].

- ***4-Methyl-N-(prop-2-yn-1-yl)benzamide (2d)***

White solid; (0.23 g, 88.7% yield); m.p: 112 °C [115 – 116 °C] [79].

- ***4-Methoxy-N-(prop-2-yn-1-yl)benzamide (2e)***

White solid; (0.259 g, 91.4% yield); m.p: 130 °C [130 – 131 °C] [80].

- ***4-Nitro-N-(prop-2-yn-1-yl)benzamide (2f)***

White solid; (0.269 g, 89.2% yield); m.p: 192 °C [189 °C] [80].

- ***N-(prop-2-yn-1-yl)-4-(trifluoromethyl)benzamide: (2g)***

White solid; (0.312 g, 93.2% yield); m.p: 145°C [146.5 – 147 °C] [81].

- ***N-(prop-2-yn-1-yl)benzamide (2h)***

White solid; (0.189 g, 79.4% yield); m.p: 106 °C [97 – 104 °C] [82].

- ***General procedure for the preparation of substituted 1-(4-substituted phenyl)-3-(prop-2-yn-1-yl) urea (5a-h)***

Method A: (using purchased p-substituted phenyl isocyanates)

4-Substituted phenyl isocyanate (2 mmol) was dissolved in 3 mL THF. 2.5 mmol of propargyl amine (0.16 mL) were added to the flask and left stirring for 2 hours at room temperature. White precipitate was formed. THF was allowed to evaporate, and the precipitate was filtered, washed several times with water, then hexane and allowed to dry. The products **5a-d** are obtained as fine amorphous powder [84, 85].

Method B: (in-situ after preparation of isocyanates from the corresponding amines, Section 4.1.2.4, Method A)

To the reaction mixture containing the formed p-substituted isocyanate, 3.2 mmol of propargyl amine (0.21 mL) were added to the flask and left stirring for 24 hours at room temperature. The precipitated by-product salt was filtered off. DCM and toluene were allowed to evaporate, and the remaining residue was washed using 1N HCl to get rid of any excess amine, followed by washing with water, then hexane and allowed to dry. The products **5e-g** were obtained as fine amorphous powder.

Method C: (in-situ after preparation of isocyanate from the corresponding acids, Section 4.1.2.4, Method B)

To the reaction mixture containing the formed phenyl isocyanate, 4 mmol of propargyl amine (0.26 mL) were added to the flask and left stirring for 24 hours at room temperature. After total evaporation of the organic solvents, 5 mL of H_2O was added to the reaction mixture and allowed to reflux for 30 minutes to remove any excess azide. The reaction was allowed to cool down. The precipitated product was then filtered, washed with 1N HCl, water, hexane and allowed to dry. The products **5h** is obtained as fine amorphous powder.

- ***1-(4-chlorophenyl)-3-(prop-2-yn-1-yl)urea (5a)***

  White solid; (0.38 g,92.4% yield); m.p. 230°C [220 – 222°C] [84].

- ***1-(4-fluorophenyl)-3-(prop-2-yn-1-yl)urea (5b)***

  White solid; (0.36 g, 93.6% yield); m.p. 165°C [168 – 170°C] [85].

- ***1-(4-bromophenyl)-3-(prop-2-yn-1-yl)urea (5c)***

  White solid; (0.46 g, 91.8% yield); m.p. 160°C [162 – 164°C] [86].

- ***1-(prop-2-yn-1-yl)-3-(p-tolyl)urea (5d)***

  White solid; (0.34 g, 89.5% yield); m.p. 170°C [174 – 176°C] [87].

- ***1-(4-methoxyphenyl)-3-(prop-2-yn-1-yl)urea (5e)***

Buff solid; (0.49 g, 82.4% yield); m.p. 185°C [177 – 179°C] [84].

- ***1-(4-nitrophenyl)-3-(prop-2-yn-1-yl)urea (5f)***

  Buff solid; (0.57 g, 81.7% yield); m.p. 110°C [107 – 108.5°C] [88].

- ***1-(prop-2-yn-1-yl)-3-(4-(trifluoromethyl)phenyl)urea (5g)***

  Buff solid; (0.64 g, 83.6% yield); m.p. 200°C [193 – 195°C] [89].

- ***1-phenyl-3-(prop-2-yn-1-yl)urea: (5h)***

  Brown solid; (0.27 g, 78.7% yield); m.p. 120°C [122 – 124°C] [83].

○ ***HPLC chromatographic purity data of 6e:***

Figures S6, S7, S8 and S9 provide HPLC chromatogram, area and purity percent report, 3D UV-absorbance spectrum and UV spectrum of **6e** respectively, confirming its high purity for biological evaluations.

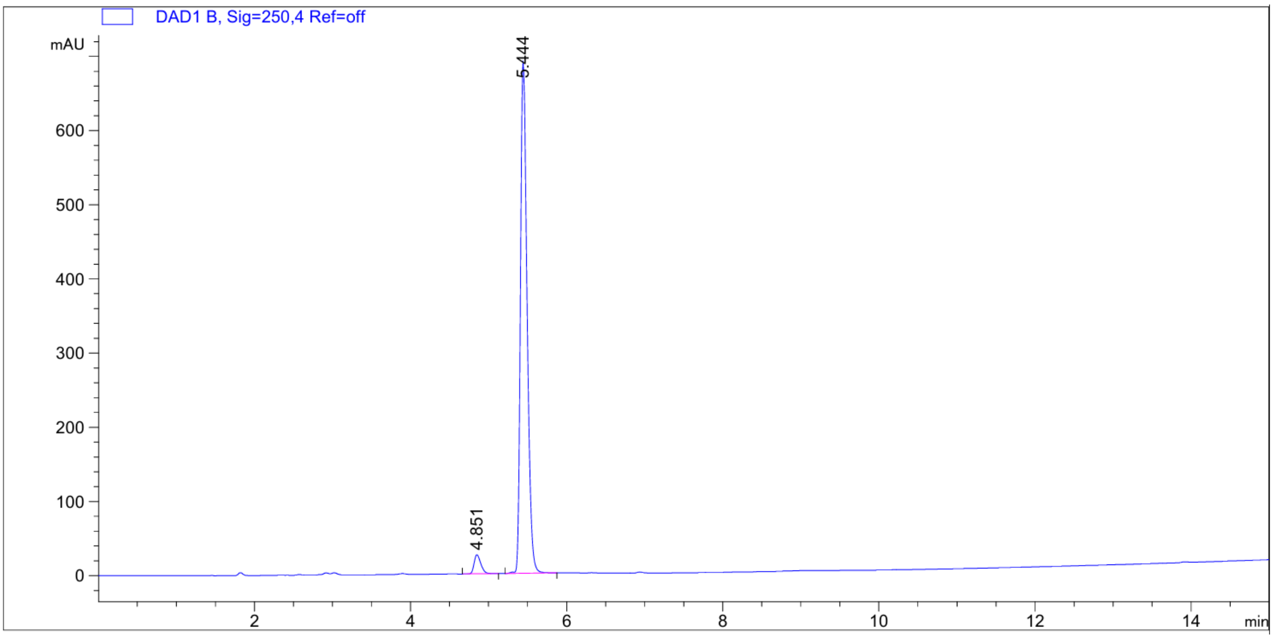

Figure S6: HPLC chromatogram of compound 6e measured at a detection wavelength of 250 nm.

```
=====================================================================
                         Area Percent Report
=====================================================================

Sorted By             :      Signal
Multiplier            :      1.0000
Dilution              :      1.0000
Sample Amount:               :     100.00000  [ng/ul]   (not used in calc.)
Do not use Multiplier & Dilution Factor with ISTDs


Signal 1: DAD1 B, Sig=250,4 Ref=off

Peak RetTime Type  Width     Area      Height     Area
  #   [min]        [min]   [mAU*s]     [mAU]        %
----|-------|----|-------|----------|----------|--------|
   1   4.851 BB    0.0922  154.40819   25.65186   3.7491
   2   5.444 VB R  0.0870 3964.16309  690.45581  96.2509

Totals :                  4118.57127  716.10767
```

Figure S7: HPLC Area Percent Report for compound **6e** showing peak retention times, peak widths, peak areas, peak heights, and area percentages (indicating a purity of 96.25% for the main peak at retention time 5.444 min)

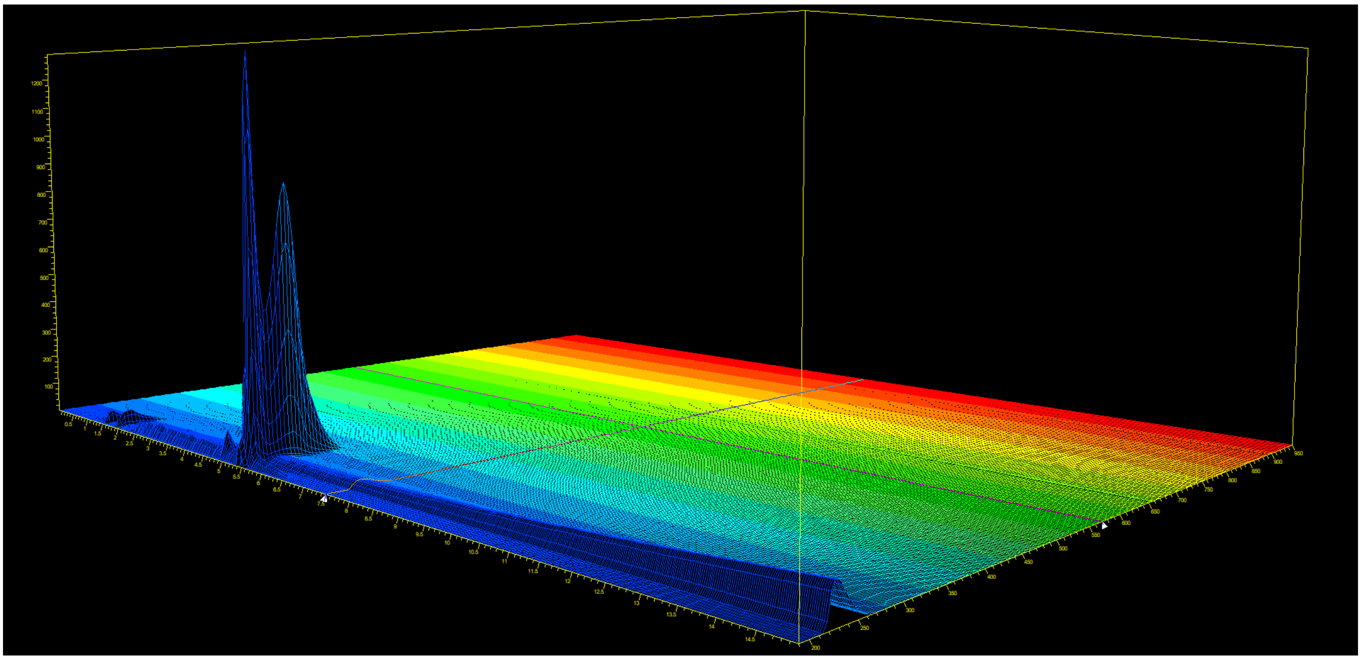

Figure S8: 3D UV-absorbance spectrum / PDA three-dimensional chromatogram profile of compound **6e**, illustrating chromatographic separation across retention time and wavelength dimensions.

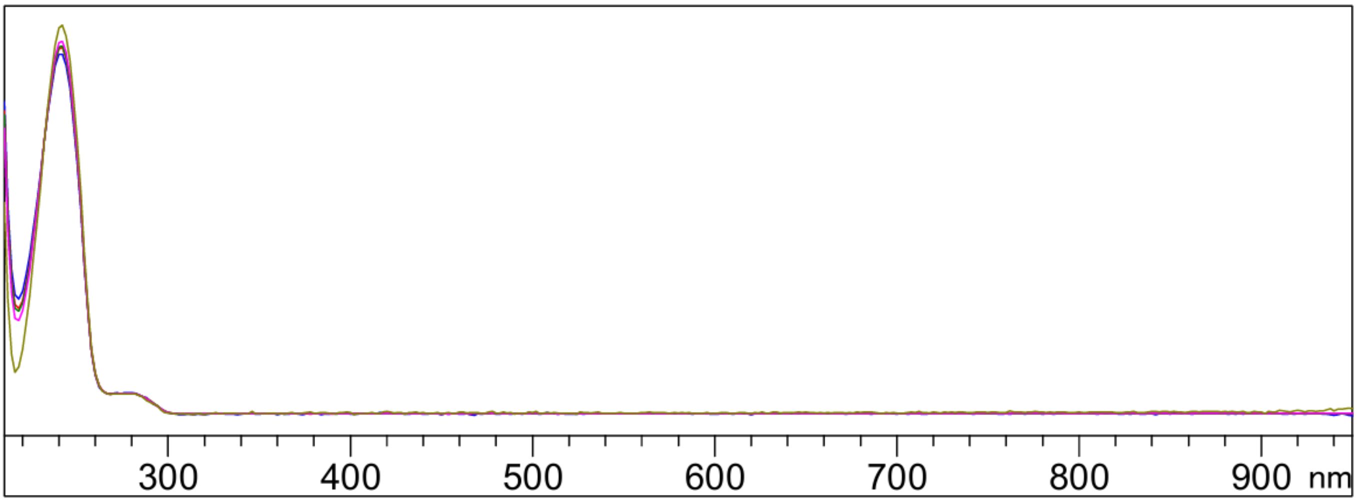

Figure S9: UV spectrum of compound **6e** recorded via PDA detector, showing absorption peaks primarily in the UV region (250 nm).

- ***Biological Evaluation:***

- ***Cytotoxicity screening on human breast cancer (MCF-7) and normal human skin fibroblasts:***

The anticancer activities of the studied compounds were assayed using human breast cancer (MCF-7), while the cytotoxicity of the studied compounds on normal cells was assessed using human skin fibroblast HSF cell line. Any of these cell lines were cultured in DMEM medium containing 10% fetal bovine serum (FBS), seeded at 5x10^3 cells per well in a 96-well cell culture plate, and incubated at 37 °C in a 5% CO_2 incubator. After 24 h for cell attachment, serial concentrations of the synthetic compounds and standard chemotherapy (Lapatinib) were incubated with HSF cells for 72 h. Cell viability was assayed by MTT method. Twenty microliters of 5 mg/ml MTT (Sigma, USA) were added to each well and the plate was incubated at 37ºC for 3 h. Then MTT solution was removed, 100 µL DMSO was added, and the absorbance of each well was measured with a microplate reader (BMG LabTech, Germany) at 570 nm. The dose IC_{50} values of the tested compounds was estimated by the GraphpadInstat software.

- ***Biochemical kinase assay:***

To assess the ability of synthesized active drug 6e to interrupt the catalysis of AXL kinase, the ADP-Glo™ Kinase Assay kit (Promega, CAT # V9101) was used, following the manufacturer's instructions. AXL (Promega, CAT # V3961) kinase enzyme systems were procured from Promega (Madison, WI, USA). The ADP-Glo™ kinase assay is a versatile, high-throughput screening tool that uses luminescence to measure kinase activity. It works by detecting and quantifying ADP produced during kinase reactions, offering a universal and homogeneous method for assessing enzyme function across various applications. Briefly, 25 µL/well kinase reactions were set up in 96-well plates as follows: 5µL of the compound of interest as an inhibitor or 5% DMSO as a negative control was added to the

wells followed by 10 μL of 75 ng mL^{-1} AXL kinase in kinase buffer, and finally 10 μL of a mixture containing 0.2 μg/μL peptide substrate and 50 μM ATP to initiate the kinase reaction. The plate was then briefly mixed for 30 seconds and incubated for 1 h at room temperature. Following that, an equal volume (25 μL/well) of ADP-Glo™ Reagent was added to terminate the kinase reaction and deplete the unconsumed ATP, leaving only ADP with a very low background of ATP. Incubation at room temperature was resumed for 40 minutes. The Kinase Detection Reagent (50 μL/well) was then added to concurrently convert ADP to ATP and introduce luciferase and luciferin to enable the newly synthesized ATP to be quantified using a luciferase/luciferin reaction. The incubation continued at room temperature for an additional 30 minutes and luminescence was measured using a PheraStar FSX plate-reading luminometer with an integration time of 1 second per well. Dose-response fitting in GraphPad Prism software was finally implemented to calculate IC_{50} values for the inhibitors.

The outcome is displayed as a graph showing percentages of luminescence and inhibition of kinase relative to positive and negative controls.

- ***Combination studies with Lapatinib:***

- ***MTT assay and determination of combination and dose reducing indices:***

The cytotoxic potential of 6e was assayed alone and as lapatinib adjuvants against breast cancer cell line (MCF-7). MCF-7 cells ($4x10^3$ cells/well) were seeded in sterile 96-well plates using DMEM medium (Lonza, USA) with 10% FBS (GIBCO, USA). After 24 h, serial concentrations of the tested compounds alone and in combination with lapatinib were incubated with these cell lines for 72 h at 37ºC in 5% CO_2 incubator. MTT method was done as described above. The half maximal inhibitory concentration (IC_{50}) values were calculated using the GraphpadInstat software. Combination index (CI) and dose reducing index (DRI) values and plots were computed by The CompuSyn software. IC_{50} values were calculated using the GraphpadInstat software. Furthermore, cellular morphological changes before and after treatment were investigated using phase contrast inverted microscope with a digital camera (Olympus, Japan).

- ***Chemo-invasion assay:***

The effects of 6e (single agent and combined with lapatinib) on MCF-7 invasion were tested using 24 well 8 μm Transwell chambers (Greiner Bio-One, Austria) as described previously [66,88] with the following modifications. Briefly, the Transwell chambers were coated with Collagen R (Serva, Germany) with a concentration of 5 $\mu g/cm^2$ for 3 h at 37°C. After coating, the chambers were washed with PBS and allowed to dry in the laminar flow cabinet. MCF-7 cells in a serum-free medium were seeded (4×10^4 cells) onto the upper surfaces and left either untreated or with the different indicated

treatments (compounds/combinations) at their corresponding IC_{10} as determined from the MTT assay. The lower wells were then filled with 600 µL DMEM supplemented with 10 % fetal bovine serum to serve as chemoattractant. After 16 h of incubation, the cells that invaded the lower surface of the filter were fixed with 4 % formaldehyde for 2 min, permeabilized with 100 % methanol for 20 min, then stained with 0.5 % crystal violet for 20 min. The cells on the apical side (non-invaded cells) were gently removed using clean cotton swabs. The invaded cells were imaged using Olympus E-330 digital DSLR camera (Olympus Corporation, Tokyo, Japan) mounted on an Optika XDS-2 inverted microscope (Bergamo, Italy) using 10X, 20X and 40X objective lenses and counted in 5 randomly selected fields. Invaded cells were normalized to control untreated cells and the percentage of invasion relative to the control was calculated for each treatment. Images were analyzed using ImageJ software [29, 88].

- ***Annexin V/PI flow cytometric analysis:***

The FITC-Annexin V Apoptosis Detection Kit (BD Bioscience, Heidelberg, Germany) was used according to the manufacturer´s protocol. In brief, floating and harvested cells were mixed, washed twice with cold phosphate buffered saline (PBS) and resuspended in binding buffer at a final density of 106 cells/mL. FITC-annexin (5 µL) and PI (5 µL) were added to 100 µL of the cell suspension containing 10^5 cells. The cell suspension was mixed by gently vortexing and then incubated for 15 min at room temperature in the dark. Subsequently, 400 µL of binding buffer were added and cells were analyzed by flow cytometry using FACS Calibur (BD Bioscience, Heidelberg, Germany) and the Cellquest Pro software (BD Bioscience). All tests were performed in duplicates and repeated twice.

- ***Biomarker analysis using ELISA technique:***
    - ***Preparation of cell lysates***

Cell lysates were prepared using RIPA lysis and extraction buffer, purchased from Thermo Scientific, USA (Catalog Number: 89900). As directed by the manufacturer, 1 mL of cold RIPA buffer was added to 40 mg of wet cell pellets, which were then kept on ice and gently shaken for 15 min and then centrifuged at 14,000×*g* for 15 min to pellet the cell debris. After that, the supernatants were transferred to new tubes and stored at 20°C for subsequent analysis [29].

- ***Biomarker analysis using ELISA technique:***

P65-NFKB (ng/mg protein), VEGF (pg/mg protein), MMP-9 (ng/mg protein), pAKT (Mmol/mg protein), STAT3 (ng/mg protein), cyclin D1 (ng/mg protein) and p38 MAPK/ERK (ng/mg protein) were

evaluated in the cell lysates from different treatment groups using the ELISA technique using Human NFKB-p65 ELISA Kit (E-EL-H1388), Human VEGF ELISA kit (Cusabio Biotech Co., LTD, China, code: CSB-E11718h), Human MMP-9 (Matrix Metalloproteinase 9) ELISA Kit (E-EL-H6075), Phospho-AKT1 (S473) Quantitative ELISA kit (ab279731), STAT3 elisa kit, Human STAT3 ELISA Kit (MBS2509698), Human Cyclin-D1 ELISA kit (Eiaab Science Inc, Wuhan, China, code: E0585h), and extracellular signal-regulated kinase, ERK ELISA Kit (MBS164291). The manufacturer's protocol was followed in all measurements. Each parameter was assayed in triplicate and was expressed relative to the total protein content in the same sample [91].

- ***Statistical Analysis:***

The data were expressed as the mean ± standard error of the mean (SEM). Multiple comparisons were analyzed using a one-way analysis of variance (ANOVA) followed by a post hoc Tukey’s multiple comparison test, and the differences were considered significant at $p < 0.05$. GraphPad Prism software package version 8.0.2 (GraphPad Software Inc., CA, USA) was used for all statistical analyses and data presentation.

Table of Contents

	Content	Page No.
S 10	IR (KBr) spectrum of 4-chloro-*N*-((1-(4,6-dimethoxy-1,3,5-triazin-2-yl)-1*H*-1,2,3-triazol-4-yl) methyl) benzamide (3a)	19
S 11	^{1}H-NMR (500 MHz, DMSO-d_6) spectrum of 4-chloro-*N*-((1-(4,6-dimethoxy-1,3,5-triazin-2-yl)-1*H*-1,2,3-triazol-4-yl) methyl) benzamide (3a)	20
S 12	^{1}H-NMR (500 MHz, DMSO-d_6 D_2O) spectrum of 4-chloro-*N*-((1-(4,6-dimethoxy-1,3,5-triazin-2-yl)-1*H*-1,2,3-triazol-4-yl) methyl) benzamide (3a)	21
S 13	^{13}C-NMR (DMSO-d_6) spectrum of 4-chloro-*N*-((1-(4,6-dimethoxy-1,3,5-triazin-2-yl)-1*H*-1,2,3-triazol-4-yl) methyl) benzamide (3a)	22
S 14	HRMS of 4-chloro-*N*-((1-(4,6-dimethoxy-1,3,5-triazin-2-yl)-1*H*-1,2,3-triazol-4-yl) methyl) benzamide (3a)	23
S 15	IR (KBr) spectrum of *N*-((1-(4,6-dimethoxy-1,3,5-triazin-2-yl)-1*H*-1,2,3-triazol-4-yl) methyl)-4-fluorobenzamide (3b)	24
S 16	^{1}H-NMR (500 MHz, DMSO-d_6) spectrum of *N*-((1-(4,6-dimethoxy-1,3,5-triazin-2-yl)-1*H*-1,2,3-triazol-4-yl) methyl)-4-fluorobenzamide (3b)	25
S 17	^{1}H-NMR (500 MHz, DMSO-d_6 D_2O) spectrum of *N*-((1-(4,6-dimethoxy-1,3,5-triazin-2-yl)-1*H*-1,2,3-triazol-4-yl) methyl)-4-fluorobenzamide (3b)	26
S 18	^{13}C-NMR (DMSO-d_6) spectrum of *N*-((1-(4,6-dimethoxy-1,3,5-triazin-2-yl)-1*H*-1,2,3-triazol-4-yl) methyl)-4-fluorobenzamide (3b)	27
S 19	HRMS of *N*-((1-(4,6-dimethoxy-1,3,5-triazin-2-yl)-1*H*-1,2,3-triazol-4-yl) methyl)-4-fluorobenzamide (3b)	28
S 20	IR (KBr) spectrum of 4-bromo-*N*-((1-(4,6-dimethoxy-1,3,5-triazin-2-yl)-1*H*-1,2,3-triazol-4-yl) methyl) benzamide (3c)	29
S 21	^{1}H-NMR (500 MHz, DMSO-d_6) spectrum of 4-bromo-*N*-((1-(4,6-dimethoxy-1,3,5-triazin-2-yl)-1*H*-1,2,3-triazol-4-yl) methyl) benzamide (3c)	30
S 22	^{1}H-NMR (500 MHz, DMSO-d_6) spectrum of 4-bromo-*N*-((1-(4,6-dimethoxy-1,3,5-triazin-2-yl)-1*H*-1,2,3-triazol-4-yl) methyl) benzamide (3c)	31
S 23	^{1}H-NMR (500 MHz, DMSO-d_6) spectrum of 4-bromo-*N*-((1-(4,6-dimethoxy-1,3,5-triazin-2-yl)-1*H*-1,2,3-triazol-4-yl) methyl) benzamide (3c)	32
S 24	^{1}H-NMR (500 MHz, DMSO-d_6, D_2O) spectrum of 4-bromo-*N*-((1-(4,6-dimethoxy-1,3,5-triazin-2-yl)-1*H*-1,2,3-triazol-4-yl) methyl) benzamide (3c)	33
S 25	^{13}C-NMR (DMSO-d_6) spectrum of 4-bromo-*N*-((1-(4,6-dimethoxy-1,3,5-triazin-2-yl)-1*H*-1,2,3-triazol-4-yl) methyl) benzamide (3c)	34
S 26	HRMS of 4-bromo-*N*-((1-(4,6-dimethoxy-1,3,5-triazin-2-yl)-1*H*-1,2,3-triazol-4-yl) methyl) benzamide (3c)	35
S 27	IR (KBr) spectrum of *N*-((1-(4,6-dimethoxy-1,3,5-triazin-2-yl)-1*H*-1,2,3-triazol-4-yl) methyl)-4-methylbenzamide (3d)	36

S 28	^{1}H-NMR (500 MHz, DMSO-d_6) spectrum of *N*-((1-(4,6-dimethoxy-1,3,5-triazin-2-yl)-1*H*-1,2,3-triazol-4-yl) methyl)-4-methylbenzamide (3d)	37
S 29	^{1}H-NMR (500 MHz, DMSO-d_6) spectrum of *N*-((1-(4,6-dimethoxy-1,3,5-triazin-2-yl)-1*H*-1,2,3-triazol-4-yl) methyl)-4-methylbenzamide (3d)	38
S 30	^{1}H-NMR (500 MHz, DMSO-d_6, D_2O) spectrum of *N*-((1-(4,6-dimethoxy-1,3,5-triazin-2-yl)-1*H*-1,2,3-triazol-4-yl) methyl)-4-methylbenzamide (3d)	39
S 31	^{13}C-NMR (DMSO-d_6) spectrum of *N*-((1-(4,6-dimethoxy-1,3,5-triazin-2-yl)-1*H*-1,2,3-triazol-4-yl) methyl)-4-methylbenzamide (3d)	40
S 32	HRMS of *N*-((1-(4,6-dimethoxy-1,3,5-triazin-2-yl)-1*H*-1,2,3-triazol-4-yl) methyl)-4-methylbenzamide (3d)	41
S 33	IR (KBr) spectrum of *N*-((1-(4,6-dimethoxy-1,3,5-triazin-2-yl)-1*H*-1,2,3-triazol-4-yl) methyl)-4-methoxybenzamide (3e)	42
S 34	^{1}H-NMR (500 MHz, DMSO-d_6) spectrum of *N*-((1-(4,6-dimethoxy-1,3,5-triazin-2-yl)-1*H*-1,2,3-triazol-4-yl) methyl)-4-methoxybenzamide (3e)	43
S 35	^{1}H-NMR (500 MHz, DMSO-d_6) spectrum of *N*-((1-(4,6-dimethoxy-1,3,5-triazin-2-yl)-1*H*-1,2,3-triazol-4-yl) methyl)-4-methoxybenzamide (3e)	44
S 36	^{1}H-NMR (500 MHz, DMSO-d_6) spectrum of *N*-((1-(4,6-dimethoxy-1,3,5-triazin-2-yl)-1*H*-1,2,3-triazol-4-yl) methyl)-4-methoxybenzamide (3e)	45
S 37	^{1}H-NMR (500 MHz, DMSO-d_6, D_2O) spectrum of *N*-((1-(4,6-dimethoxy-1,3,5-triazin-2-yl)-1*H*-1,2,3-triazol-4-yl) methyl)-4-methoxybenzamide (3e)	46
S 38	^{13}C-NMR (DMSO-d_6) spectrum of *N*-((1-(4,6-dimethoxy-1,3,5-triazin-2-yl)-1*H*-1,2,3-triazol-4-yl) methyl)-4-methoxybenzamide (3e)	47
S 39	HRMS of *N*-((1-(4,6-dimethoxy-1,3,5-triazin-2-yl)-1*H*-1,2,3-triazol-4-yl) methyl)-4-methoxybenzamide (3e)	48
S 40	IR (KBr) spectrum of *N*-((1-(4,6-dimethoxy-1,3,5-triazin-2-yl)-1*H*-1,2,3-triazol-4-yl) methyl)-4-nitrobenzamide (3f)	49
S 41	^{1}H-NMR (500 MHz, DMSO-d_6) spectrum of *N*-((1-(4,6-dimethoxy-1,3,5-triazin-2-yl)-1*H*-1,2,3-triazol-4-yl) methyl)-4-nitrobenzamide (3f)	50
S 42	^{1}H-NMR (500 MHz, DMSO-d_6) spectrum of *N*-((1-(4,6-dimethoxy-1,3,5-triazin-2-yl)-1*H*-1,2,3-triazol-4-yl) methyl)-4-nitrobenzamide (3f)	51
S 43	^{1}H-NMR (500 MHz, DMSO-d_6) spectrum of *N*-((1-(4,6-dimethoxy-1,3,5-triazin-2-yl)-1*H*-1,2,3-triazol-4-yl) methyl)-4-nitrobenzamide (3f)	52
S 44	^{1}H-NMR (500 MHz, DMSO-d_6) spectrum of *N*-((1-(4,6-dimethoxy-1,3,5-triazin-2-yl)-1*H*-1,2,3-triazol-4-yl) methyl)-4-nitrobenzamide (3f)	53
S 45	^{1}H-NMR (500 MHz, DMSO-d_6, D_2O) spectrum of *N*-((1-(4,6-dimethoxy-1,3,5-triazin-2-yl)-1*H*-1,2,3-triazol-4-yl) methyl)-4-nitrobenzamide (3f)	54
S 46	^{13}C-NMR (DMSO-d_6) spectrum of *N*-((1-(4,6-dimethoxy-1,3,5-triazin-2-yl)-1*H*-1,2,3-triazol-4-yl) methyl)-4-nitrobenzamide (3f)	55
S 47	HRMS of *N*-((1-(4,6-dimethoxy-1,3,5-triazin-2-yl)-1*H*-1,2,3-triazol-4-yl) methyl)-4-nitrobenzamide (3f)	56

S 48	IR (KBr) spectrum of *N*-((1-(4,6-dimethoxy-1,3,5-triazin-2-yl)-1*H*-1,2,3-triazol-4-yl) methyl)-4-(trifluoromethyl) benzamide (3g)	57
S 49	^{1}H-NMR (500 MHz, DMSO-d_6) spectrum of *N*-((1-(4,6-dimethoxy-1,3,5-triazin-2-yl)-1*H*-1,2,3-triazol-4-yl) methyl)-4-(trifluoromethyl) benzamide (3g)	58
S 50	^{1}H-NMR (500 MHz, DMSO-d_6, D_2O) spectrum of *N*-((1-(4,6-dimethoxy-1,3,5-triazin-2-yl)-1*H*-1,2,3-triazol-4-yl) methyl)-4-(trifluoromethyl) benzamide (3g)	59
S 51	^{13}C-NMR (DMSO-d_6) spectrum of *N*-((1-(4,6-dimethoxy-1,3,5-triazin-2-yl)-1*H*-1,2,3-triazol-4-yl) methyl)-4-(trifluoromethyl) benzamide (3g)	60
S 52	HRMS of *N*-((1-(4,6-dimethoxy-1,3,5-triazin-2-yl)-1*H*-1,2,3-triazol-4-yl) methyl)-4-(trifluoromethyl) benzamide (3g)	61
S 53	IR (KBr) spectrum of *N*-((1-(4,6-dimethoxy-1,3,5-triazin-2-yl)-1*H*-1,2,3-triazol-4-yl) methyl) benzamide (3h)	62
S 54	^{1}H-NMR (500 MHz, DMSO-d_6) spectrum of *N*-((1-(4,6-dimethoxy-1,3,5-triazin-2-yl)-1*H*-1,2,3-triazol-4-yl) methyl) benzamide (3h)	63
S 55	^{1}H-NMR (500 MHz, DMSO-d_6) spectrum of *N*-((1-(4,6-dimethoxy-1,3,5-triazin-2-yl)-1*H*-1,2,3-triazol-4-yl) methyl) benzamide (3h)	64
S 56	^{1}H-NMR (500 MHz, DMSO-d_6) spectrum of *N*-((1-(4,6-dimethoxy-1,3,5-triazin-2-yl)-1*H*-1,2,3-triazol-4-yl) methyl) benzamide (3h)	65
S 57	^{13}C-NMR (DMSO-d_6) spectrum of *N*-((1-(4,6-dimethoxy-1,3,5-triazin-2-yl)-1*H*-1,2,3-triazol-4-yl) methyl) benzamide (3h)	66
S 58	HRMS of *N*-((1-(4,6-dimethoxy-1,3,5-triazin-2-yl)-1*H*-1,2,3-triazol-4-yl) methyl) benzamide (3h)	67
S 59	IR (KBr) spectrum of 1-(4-chlorophenyl)-3-((1-(4,6-dimethoxy-1,3,5-triazin-2-yl)-1*H*-1,2,3-triazol-4-yl) methyl) urea (6a)	68
S 60	^{1}H-NMR (500 MHz, DMSO-d_6) spectrum of 1-(4-chlorophenyl)-3-((1-(4,6-dimethoxy-1,3,5-triazin-2-yl)-1*H*-1,2,3-triazol-4-yl) methyl) urea (6a)	69
S 61	^{1}H-NMR (500 MHz, DMSO-d_6) spectrum of 1-(4-chlorophenyl)-3-((1-(4,6-dimethoxy-1,3,5-triazin-2-yl)-1*H*-1,2,3-triazol-4-yl) methyl) urea (6a)	70
S 62	^{1}H-NMR (500 MHz, DMSO-d_6) spectrum of 1-(4-chlorophenyl)-3-((1-(4,6-dimethoxy-1,3,5-triazin-2-yl)-1*H*-1,2,3-triazol-4-yl) methyl) urea (6a)	71
S 63	^{1}H-NMR (500 MHz, DMSO-d_6, D_2O) spectrum of 1-(4-chlorophenyl)-3-((1-(4,6-dimethoxy-1,3,5-triazin-2-yl)-1*H*-1,2,3-triazol-4-yl) methyl) urea (6a)	72
S 64	^{13}C-NMR (DMSO-d_6) spectrum of 1-(4-chlorophenyl)-3-((1-(4,6-dimethoxy-1,3,5-triazin-2-yl)-1*H*-1,2,3-triazol-4-yl) methyl) urea (6a)	73
S 65	HRMS of 1-(4-chlorophenyl)-3-((1-(4,6-dimethoxy-1,3,5-triazin-2-yl)-1*H*-1,2,3-triazol-4-yl) methyl) urea (6a)	74
S 66	IR (KBr) spectrum of 1-((1-(4,6-dimethoxy-1,3,5-triazin-2-yl)-1*H*-1,2,3-triazol-4-yl) methyl)-3-(4-fluorophenyl) urea (6b)	75
S 67	^{1}H-NMR (500 MHz, DMSO-d_6) spectrum of 1-((1-(4,6-dimethoxy-1,3,5-triazin-2-yl)-1*H*-1,2,3-triazol-4-yl) methyl)-3-(4-fluorophenyl) urea (6b)	76

S 68	^{1}H-NMR (500 MHz, DMSO-d_6) spectrum of 1-((1-(4,6-dimethoxy-1,3,5-triazin-2-yl)-1*H*-1,2,3-triazol-4-yl) methyl)-3-(4-fluorophenyl) urea (6b)	77
S 69	^{1}H-NMR (500 MHz, DMSO-d_6) spectrum of 1-((1-(4,6-dimethoxy-1,3,5-triazin-2-yl)-1*H*-1,2,3-triazol-4-yl) methyl)-3-(4-fluorophenyl) urea (6b)	78
S 70	^{1}H-NMR (500 MHz, DMSO-d_6, D_2O) spectrum of 1-((1-(4,6-dimethoxy-1,3,5-triazin-2-yl)-1*H*-1,2,3-triazol-4-yl) methyl)-3-(4-fluorophenyl) urea (6b)	79
S 71	^{13}C-NMR (DMSO-d_6) spectrum of 1-((1-(4,6-dimethoxy-1,3,5-triazin-2-yl)-1*H*-1,2,3-triazol-4-yl) methyl)-3-(4-fluorophenyl) urea (6b)	80
S 72	HRMS of 1-((1-(4,6-dimethoxy-1,3,5-triazin-2-yl)-1*H*-1,2,3-triazol-4-yl) methyl)-3-(4-fluorophenyl) urea (6b)	81
S 73	IR (KBr) spectrum of 1-(4-bromophenyl)-3-((1-(4,6-dimethoxy-1,3,5-triazin-2-yl)-1*H*-1,2,3-triazol-4-yl) methyl) urea (6c)	82
S 74	^{1}H-NMR (500 MHz, DMSO-d_6) spectrum of 1-(4-bromophenyl)-3-((1-(4,6-dimethoxy-1,3,5-triazin-2-yl)-1*H*-1,2,3-triazol-4-yl) methyl) urea (6c)	83
S 75	^{1}H-NMR (500 MHz, DMSO-d_6, D_2O) spectrum of 1-(4-bromophenyl)-3-((1-(4,6-dimethoxy-1,3,5-triazin-2-yl)-1*H*-1,2,3-triazol-4-yl) methyl) urea (6c)	84
S 76	^{13}C-NMR (DMSO-d_6) spectrum of 1-(4-bromophenyl)-3-((1-(4,6-dimethoxy-1,3,5-triazin-2-yl)-1*H*-1,2,3-triazol-4-yl) methyl) urea (6c)	85
S 77	HRMS of 1-(4-bromophenyl)-3-((1-(4,6-dimethoxy-1,3,5-triazin-2-yl)-1*H*-1,2,3-triazol-4-yl) methyl) urea (6c)	86
S 78	IR (KBr) spectrum of 1-((1-(4,6-dimethoxy-1,3,5-triazin-2-yl)-1*H*-1,2,3-triazol-4-yl) methyl)-3-(p-tolyl) urea (6d)	87
S 79	^{1}H-NMR (500 MHz, DMSO-d_6) spectrum of 1-((1-(4,6-dimethoxy-1,3,5-triazin-2-yl)-1*H*-1,2,3-triazol-4-yl) methyl)-3-(p-tolyl) urea (6d)	88
S 80	^{1}H-NMR (500 MHz, DMSO-d_6) spectrum of 1-((1-(4,6-dimethoxy-1,3,5-triazin-2-yl)-1*H*-1,2,3-triazol-4-yl) methyl)-3-(p-tolyl) urea (6d)	89
S 81	^{1}H-NMR (500 MHz, DMSO-d_6) spectrum of 1-((1-(4,6-dimethoxy-1,3,5-triazin-2-yl)-1*H*-1,2,3-triazol-4-yl) methyl)-3-(p-tolyl) urea (6d)	90
S 82	^{1}H-NMR (500 MHz, DMSO-d_6, D_2O) spectrum of 1-((1-(4,6-dimethoxy-1,3,5-triazin-2-yl)-1*H*-1,2,3-triazol-4-yl) methyl)-3-(p-tolyl) urea (6d)	91
S 83	^{13}C-NMR (DMSO-d_6) spectrum of 1-((1-(4,6-dimethoxy-1,3,5-triazin-2-yl)-1*H*-1,2,3-triazol-4-yl) methyl)-3-(p-tolyl) urea (6d)	92
S 84	HRMS of 1-((1-(4,6-dimethoxy-1,3,5-triazin-2-yl)-1*H*-1,2,3-triazol-4-yl) methyl)-3-(p-tolyl) urea (6d)	93
S 85	IR (KBr) spectrum of 1-((1-(4,6-dimethoxy-1,3,5-triazin-2-yl)-1*H*-1,2,3-triazol-4-yl) methyl)-3-(4-methoxyphenyl) urea (6e)	94
S 86	^{1}H-NMR (500 MHz, DMSO-d_6) spectrum of 1-((1-(4,6-dimethoxy-1,3,5-triazin-2-yl)-1*H*-1,2,3-triazol-4-yl) methyl)-3-(4-methoxyphenyl) urea (6e)	95
S 87	^{1}H-NMR (500 MHz, DMSO-d_6) spectrum of 1-((1-(4,6-dimethoxy-1,3,5-triazin-2-yl)-1*H*-1,2,3-triazol-4-yl) methyl)-3-(4-methoxyphenyl) urea (6e)	96

S 88	^{1}H-NMR (500 MHz, DMSO-d_6, D_2O) spectrum of 1-((1-(4,6-dimethoxy-1,3,5-triazin-2-yl)-1*H*-1,2,3-triazol-4-yl) methyl)-3-(4-methoxyphenyl) urea (6e)	97
S 89	^{13}C-NMR (DMSO-d_6) spectrum of 1-((1-(4,6-dimethoxy-1,3,5-triazin-2-yl)-1*H*-1,2,3-triazol-4-yl) methyl)-3-(4-methoxyphenyl) urea (6e)	98
S 90	HRMS of 1-((1-(4,6-dimethoxy-1,3,5-triazin-2-yl)-1*H*-1,2,3-triazol-4-yl) methyl)-3-(4-methoxyphenyl) urea (6e)	99
S 91	IR (KBr) spectrum of 1-((1-(4,6-dimethoxy-1,3,5-triazin-2-yl)-1*H*-1,2,3-triazol-4-yl) methyl)-3-(4-nitrophenyl) urea (6f)	100
S 92	^{1}H-NMR (500 MHz, DMSO-d_6) spectrum of 1-((1-(4,6-dimethoxy-1,3,5-triazin-2-yl)-1*H*-1,2,3-triazol-4-yl) methyl)-3-(4-nitrophenyl) urea (6f)	101
S 93	^{1}H-NMR (500 MHz, DMSO-d_6) spectrum of 1-((1-(4,6-dimethoxy-1,3,5-triazin-2-yl)-1*H*-1,2,3-triazol-4-yl) methyl)-3-(4-nitrophenyl) urea (6f)	102
S 94	^{1}H-NMR (500 MHz, DMSO-d_6) spectrum of 1-((1-(4,6-dimethoxy-1,3,5-triazin-2-yl)-1*H*-1,2,3-triazol-4-yl) methyl)-3-(4-nitrophenyl) urea (6f)	103
S 95	^{1}H-NMR (500 MHz, DMSO-d_6, D_2O) spectrum of 1-((1-(4,6-dimethoxy-1,3,5-triazin-2-yl)-1*H*-1,2,3-triazol-4-yl) methyl)-3-(4-nitrophenyl) urea (6f)	104
S 96	^{13}C-NMR (DMSO-d_6) spectrum of 1-((1-(4,6-dimethoxy-1,3,5-triazin-2-yl)-1*H*-1,2,3-triazol-4-yl) methyl)-3-(4-nitrophenyl) urea (6f)	105
S 97	HRMS of 1-((1-(4,6-dimethoxy-1,3,5-triazin-2-yl)-1*H*-1,2,3-triazol-4-yl) methyl)-3-(4-nitrophenyl) urea (6f)	106
S 98	IR (KBr) spectrum of 1-((1-(4,6-dimethoxy-1,3,5-triazin-2-yl)-1*H*-1,2,3-triazol-4-yl) methyl)-3-(4-(trifluoromethyl) phenyl) urea (6g)	107
S 99	^{1}H-NMR (500 MHz, DMSO-d_6) spectrum of 1-((1-(4,6-dimethoxy-1,3,5-triazin-2-yl)-1*H*-1,2,3-triazol-4-yl) methyl)-3-(4-(trifluoromethyl) phenyl) urea (6g)	108
S 100	^{1}H-NMR (500 MHz, DMSO-d_6) spectrum of 1-((1-(4,6-dimethoxy-1,3,5-triazin-2-yl)-1*H*-1,2,3-triazol-4-yl) methyl)-3-(4-(trifluoromethyl) phenyl) urea (6g)	109
S 101	^{1}H-NMR (500 MHz, DMSO-d_6) spectrum of 1-((1-(4,6-dimethoxy-1,3,5-triazin-2-yl)-1*H*-1,2,3-triazol-4-yl) methyl)-3-(4-(trifluoromethyl) phenyl) urea (6g)	110
S 102	^{13}C-NMR (DMSO-d_6) spectrum of 1-((1-(4,6-dimethoxy-1,3,5-triazin-2-yl)-1*H*-1,2,3-triazol-4-yl) methyl)-3-(4-(trifluoromethyl) phenyl) urea (6g)	111
S 103	HRMS of 1-((1-(4,6-dimethoxy-1,3,5-triazin-2-yl)-1*H*-1,2,3-triazol-4-yl) methyl)-3-(4-(trifluoromethyl) phenyl) urea (6g)	112
S 104	IR (KBr) spectrum of 1-((1-(4,6-dimethoxy-1,3,5-triazin-2-yl)-1*H*-1,2,3-triazol-4-yl) methyl)-3-phenyl urea (6h)	113
S 105	^{1}H-NMR (500 MHz, DMSO-d_6) spectrum of 1-((1-(4,6-dimethoxy-1,3,5-triazin-2-yl)-1*H*-1,2,3-triazol-4-yl) methyl)-3-phenyl urea (6h)	114
S 106	^{1}H-NMR (500 MHz, DMSO-d_6) spectrum of 1-((1-(4,6-dimethoxy-1,3,5-triazin-2-yl)-1*H*-1,2,3-triazol-4-yl) methyl)-3-phenyl urea (6h)	115
S 107	^{1}H-NMR (500 MHz, DMSO-d_6) spectrum of 1-((1-(4,6-dimethoxy-1,3,5-triazin-2-yl)-1*H*-1,2,3-triazol-4-yl) methyl)-3-phenyl urea (6h)	116

S 108	^{1}H-NMR (500 MHz, DMSO-d_6) spectrum of 1-((1-(4,6-dimethoxy-1,3,5-triazin-2-yl)-1*H*-1,2,3-triazol-4-yl) methyl)-3-phenyl urea (6h)	117
S 109	HRMS of 1-((1-(4,6-dimethoxy-1,3,5-triazin-2-yl)-1*H*-1,2,3-triazol-4-yl) methyl)-3-phenyl urea (6h)	118

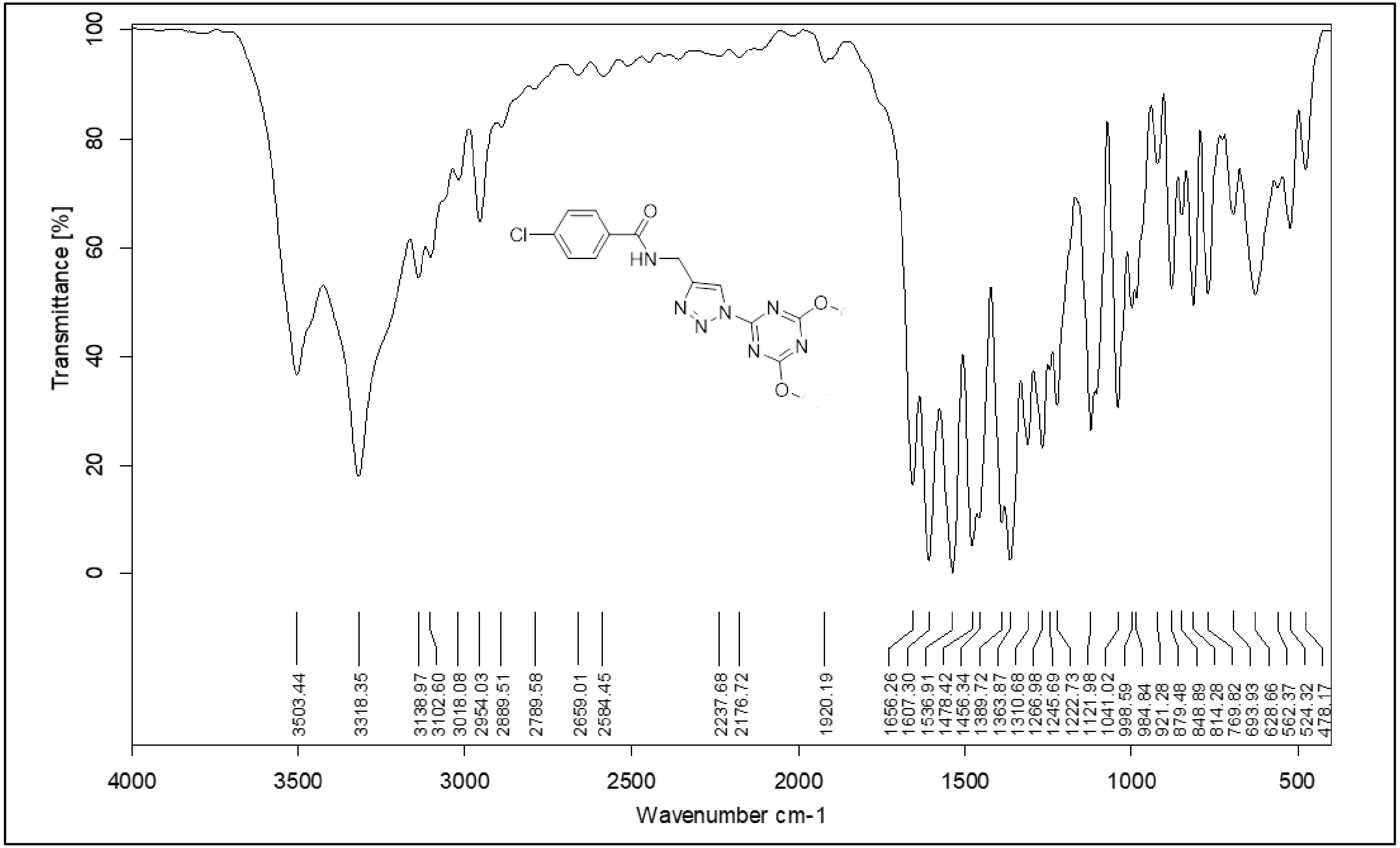

Figure S10: IR (KBr) spectrum of 4-chloro-*N*-((1-(4,6-dimethoxy-1,3,5-triazin-2-yl)-1*H*-1,2,3-triazol-4-yl) methyl) benzamide **(3a)**

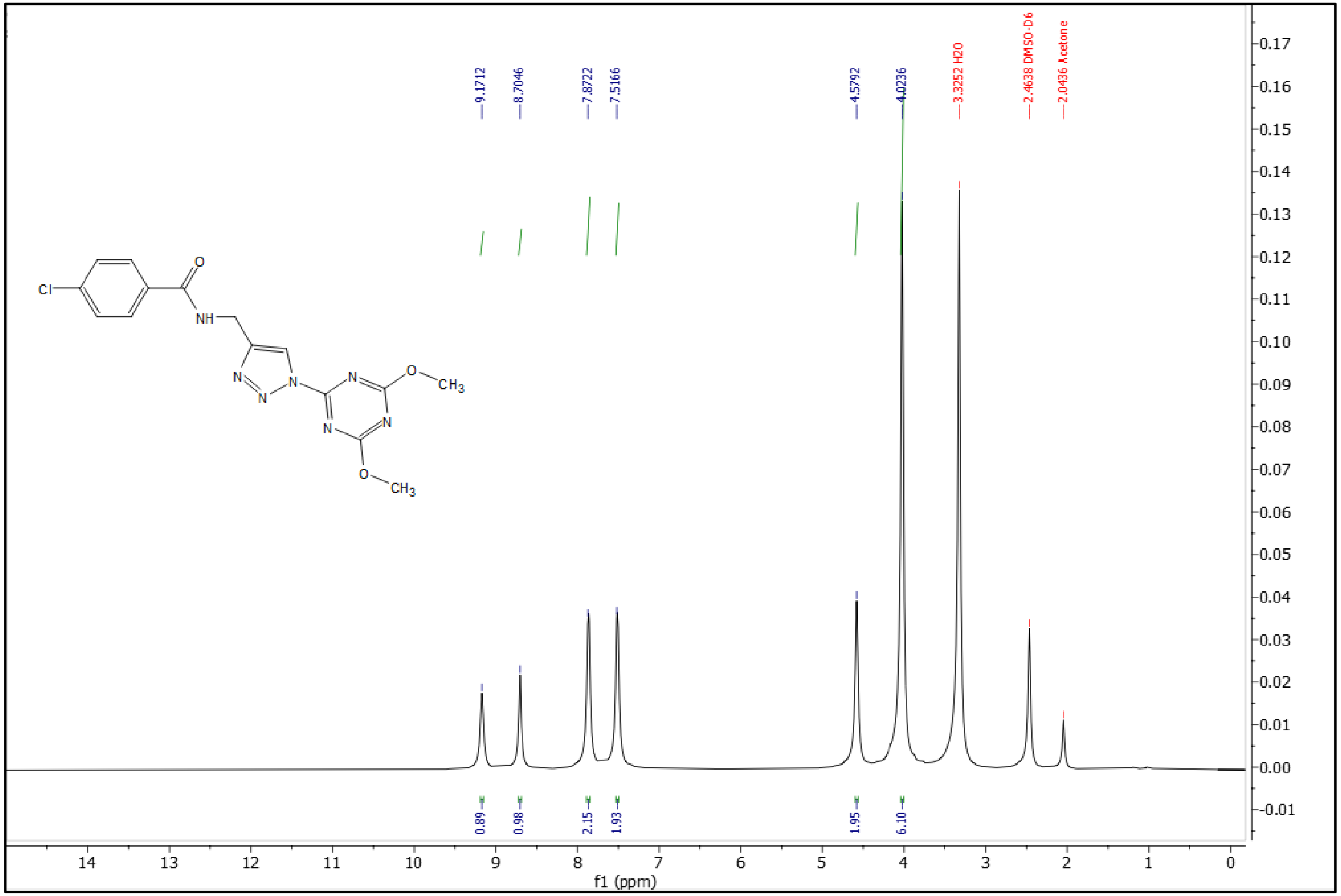

Figure S11: ^{1}H-NMR (500 MHz, DMSO-d_6) spectrum of 4-chloro-*N*-((1-(4,6-dimethoxy-1,3,5-triazin-2-yl)-1*H*-1,2,3-triazol-4-yl) methyl) benzamide **(3a)**

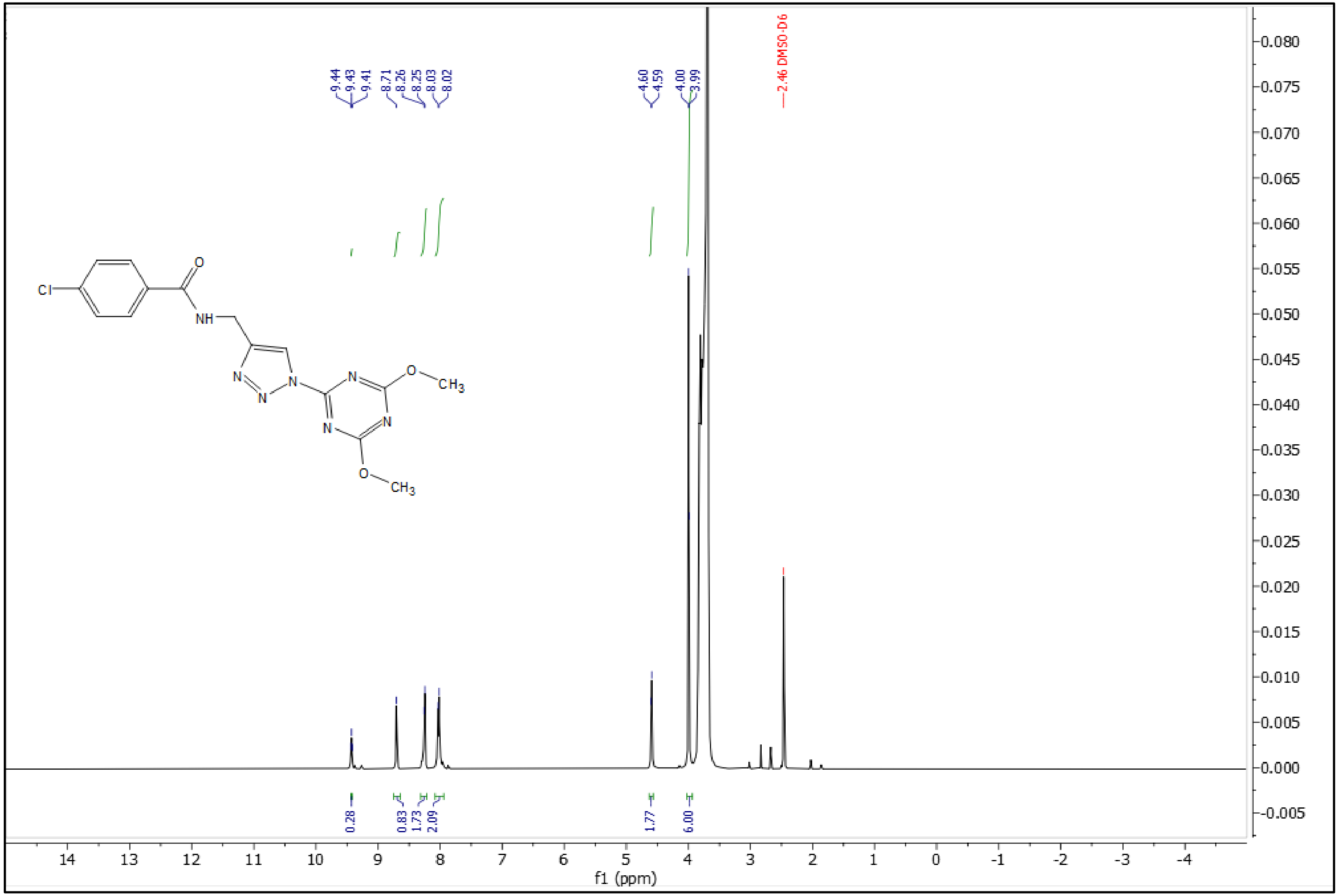

Figure S12: ^{1}H-NMR (500 MHz, DMSO-d_6, D_2O) spectrum of 4-chloro-*N*-((1-(4,6-dimethoxy-1,3,5-triazin-2-yl)-1*H*-1,2,3-triazol-4-yl) methyl) benzamide **(3a)**

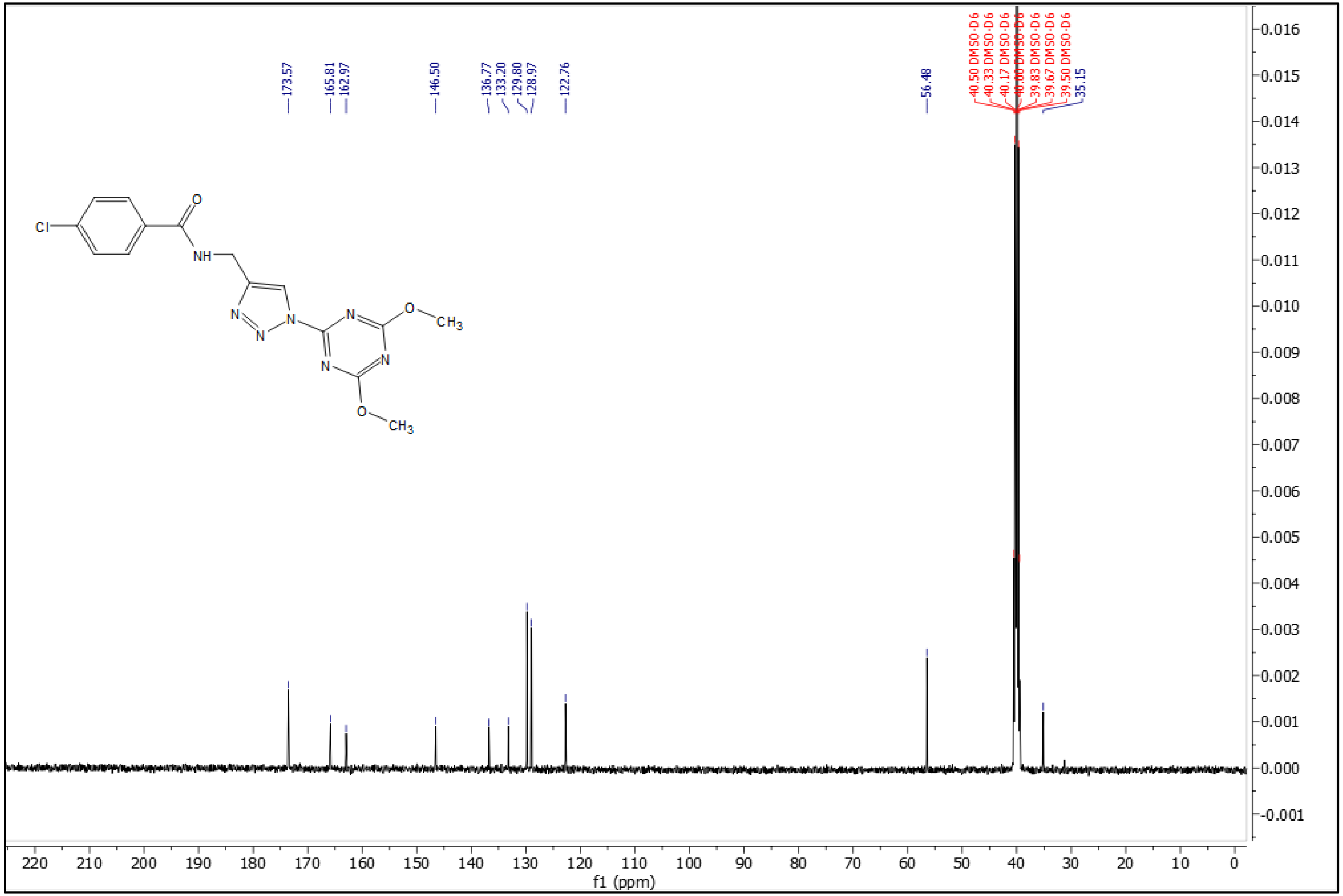

Figure S13: ^{13}C-NMR (DMSO-d_6) spectrum of 4-chloro-*N*-((1-(4,6-dimethoxy-1,3,5-triazin-2-yl)-1*H*-1,2,3-triazol-4-yl) methyl) benzamide **(3a)**

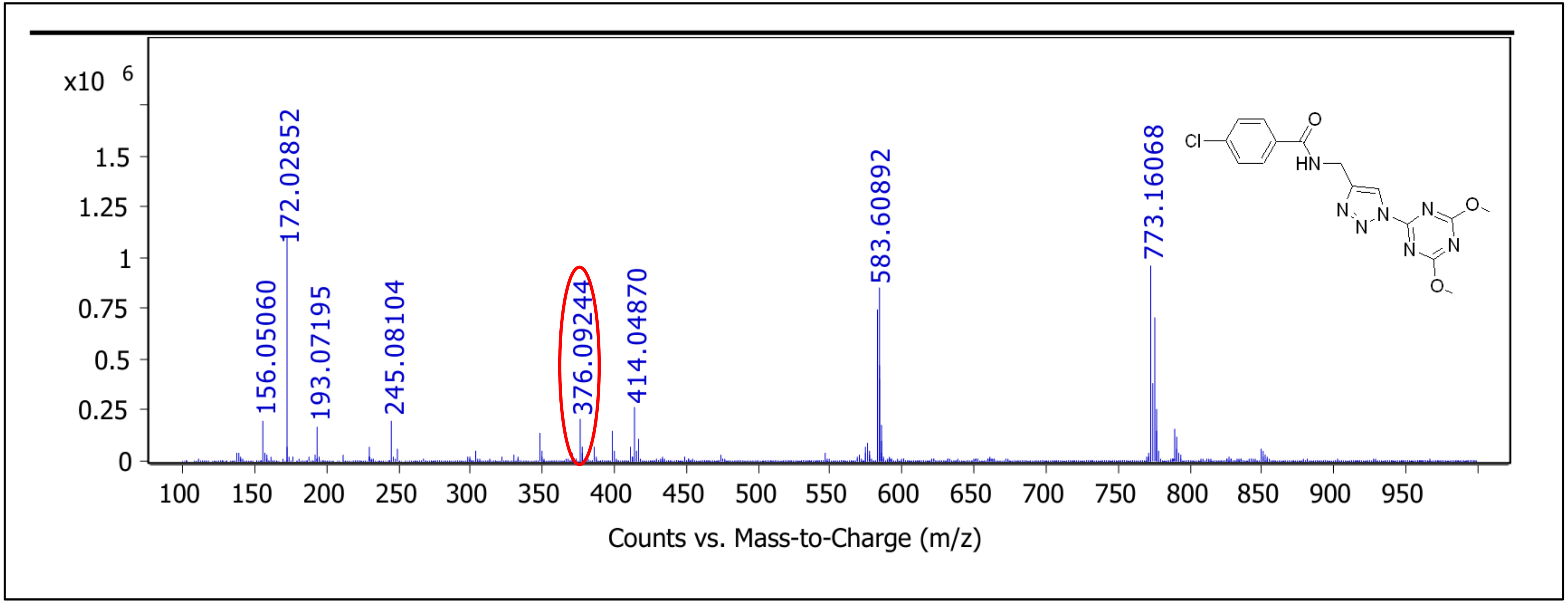

Figure S14: HRMS of 4-chloro-*N*-((1-(4,6-dimethoxy-1,3,5-triazin-2-yl)-1*H*-1,2,3-triazol-4-yl) methyl) benzamide **(3a)**

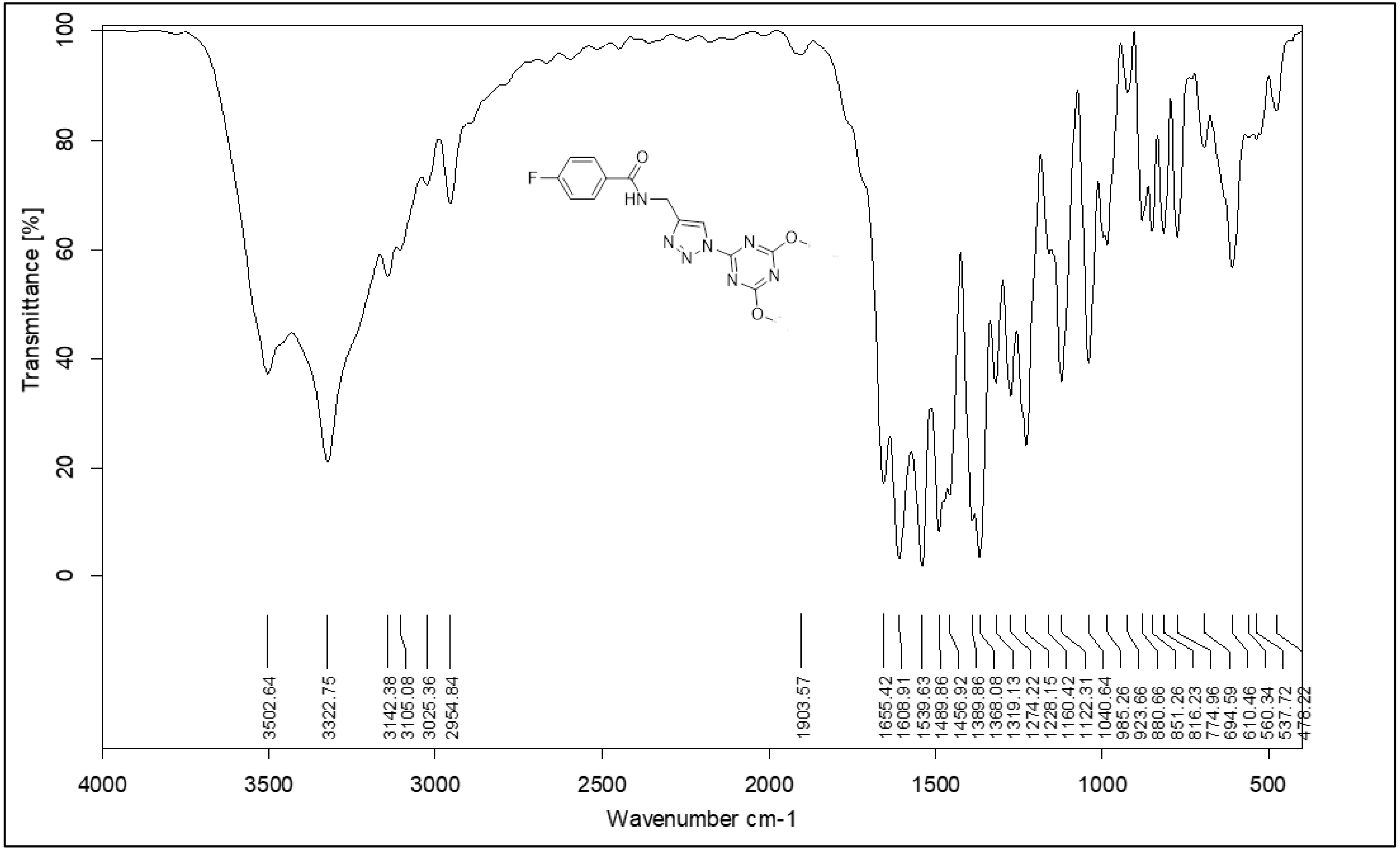

Figure S15: IR (KBr) spectrum of *N*-((1-(4,6-dimethoxy-1,3,5-triazin-2-yl)-1*H*-1,2,3-triazol-4-yl) methyl)-4-fluorobenzamide **(3b)**

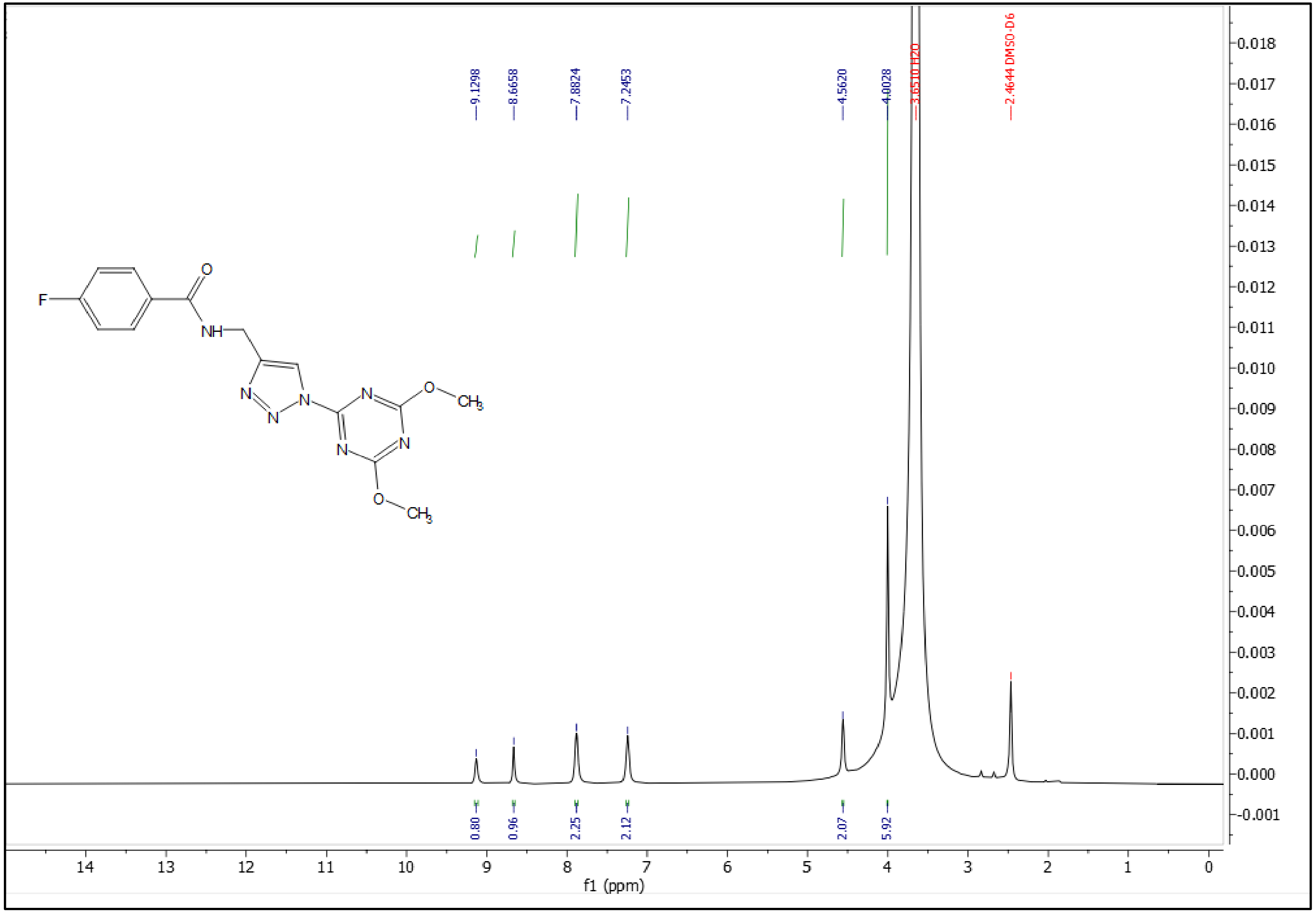

Figure S16: ^{1}H-NMR (500 MHz, DMSO-d_6) spectrum of *N*-((1-(4,6-dimethoxy-1,3,5-triazin-2-yl)-1*H*-1,2,3-triazol-4-yl) methyl)-4-fluorobenzamide **(3b)**

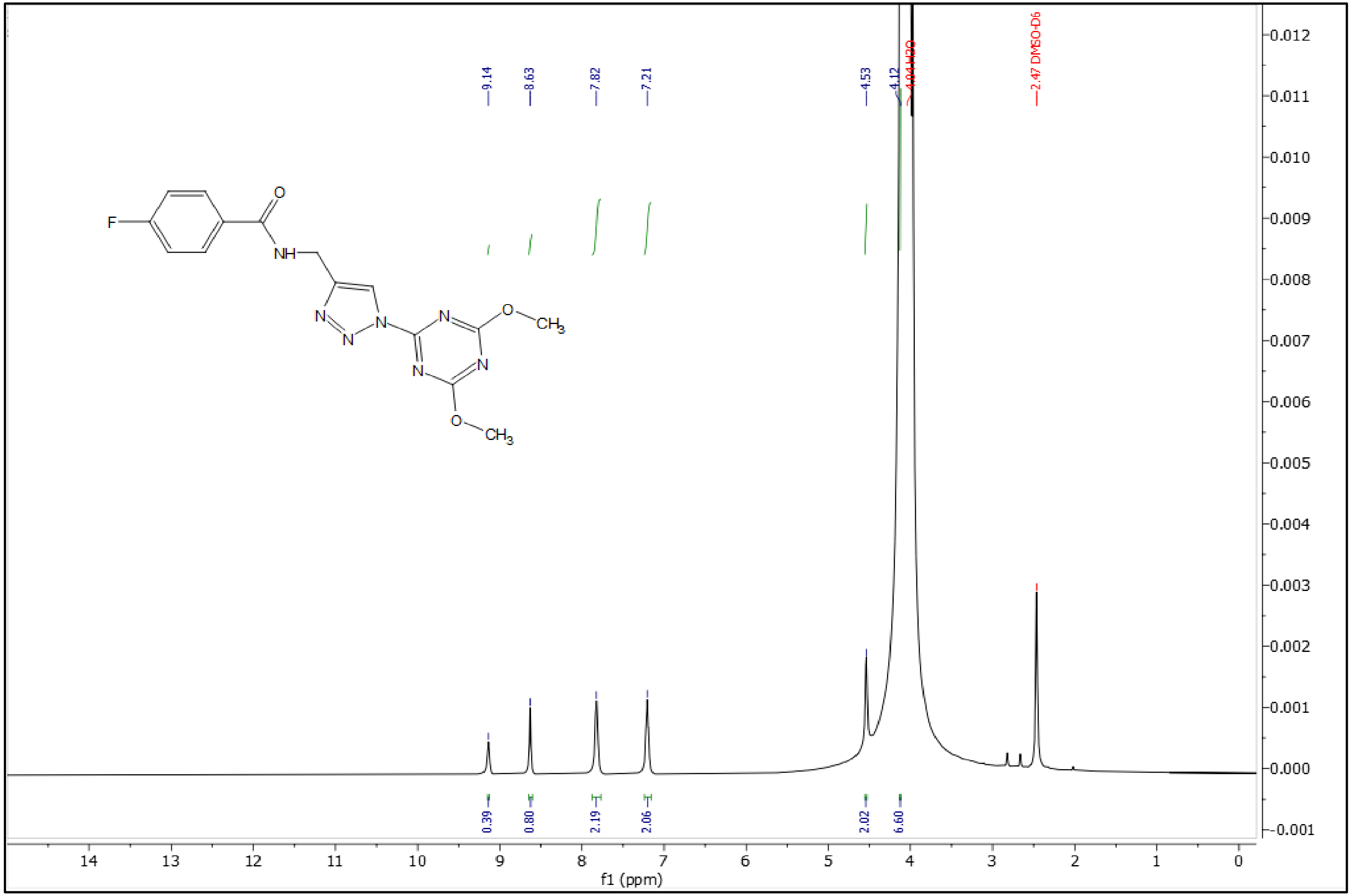

Figure S17: ^{1}H-NMR (500 MHz, DMSO-d_6, D_2O) spectrum of *N*-((1-(4,6-dimethoxy-1,3,5-triazin-2-yl)-1*H*-1,2,3-triazol-4-yl) methyl)-4-fluorobenzamide **(3b)**

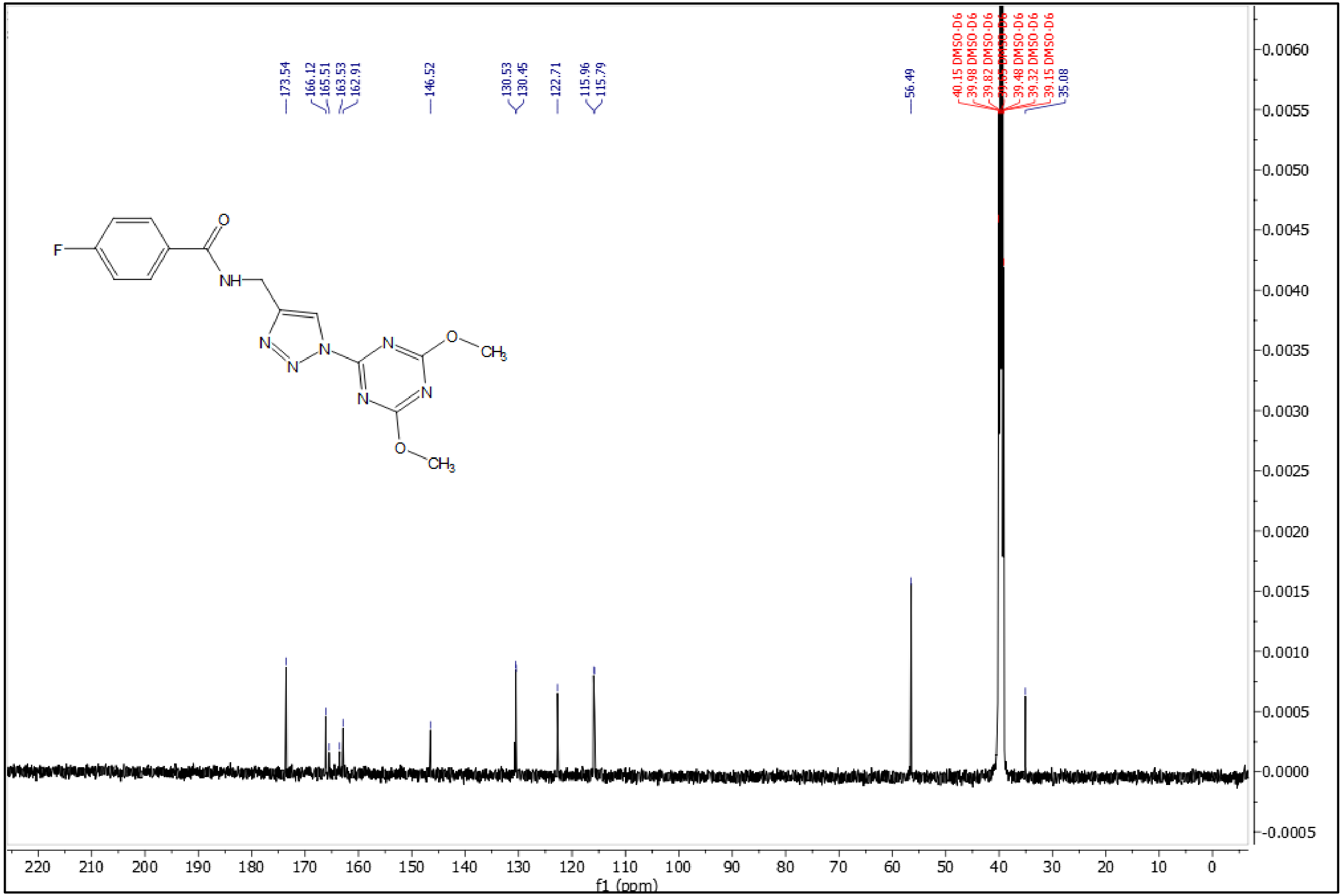

Figure S18: ^{13}C-NMR (DMSO-d_6) spectrum of *N*-((1-(4,6-dimethoxy-1,3,5-triazin-2-yl)-1*H*-1,2,3-triazol-4-yl) methyl)-4-fluorobenzamide **(3b)**

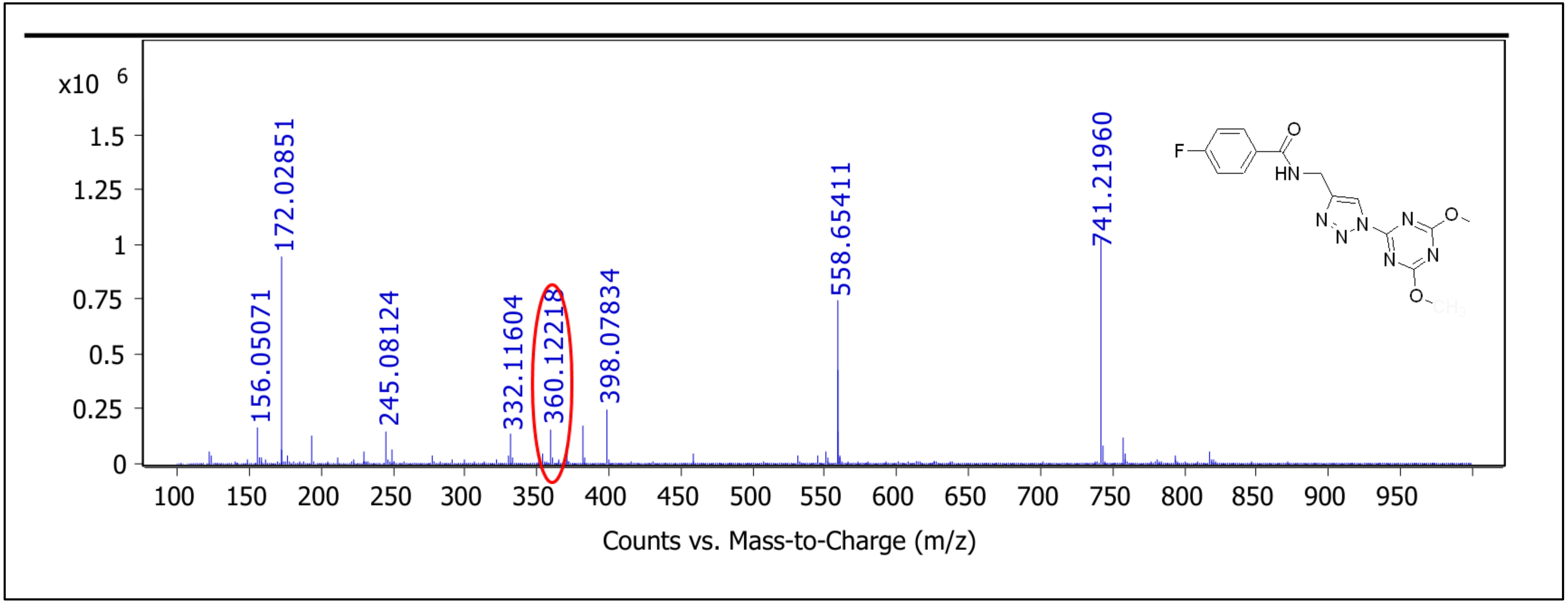

Figure S19: HRMS of *N*-((1-(4,6-dimethoxy-1,3,5-triazin-2-yl)-1*H*-1,2,3-triazol-4-yl) methyl)-4-fluorobenzamide **(3b)**

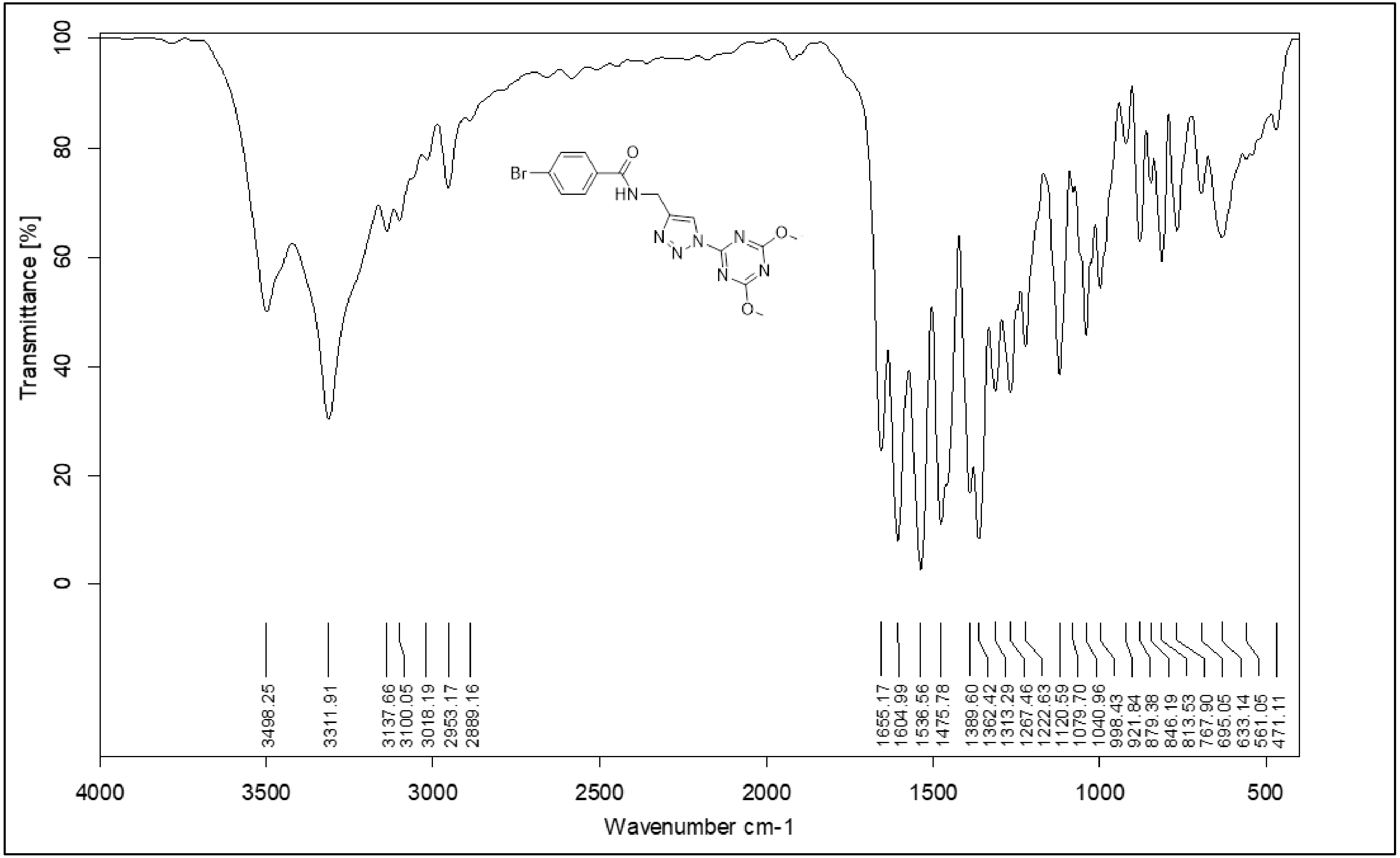

Figure S20: IR (KBr) spectrum of 4-bromo-*N*-((1-(4,6-dimethoxy-1,3,5-triazin-2-yl)-1*H*-1,2,3-triazol-4-yl) methyl) benzamide **(3c)**

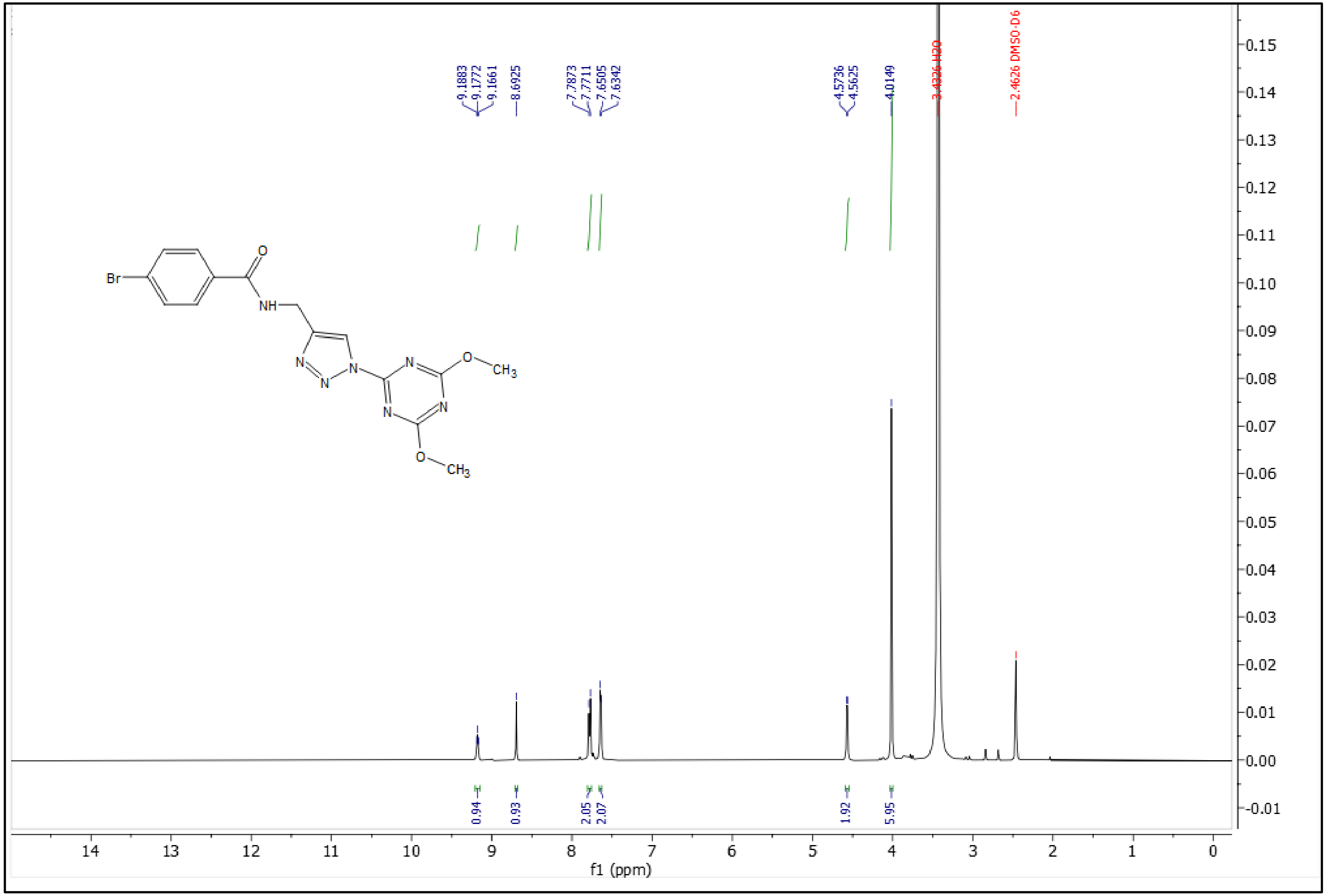

Figure S21: ^{1}H-NMR (500 MHz, DMSO-d_6) spectrum of 4-bromo-*N*-((1-(4,6-dimethoxy-1,3,5-triazin-2-yl)-1*H*-1,2,3-triazol-4-yl) methyl) benzamide **(3c)**

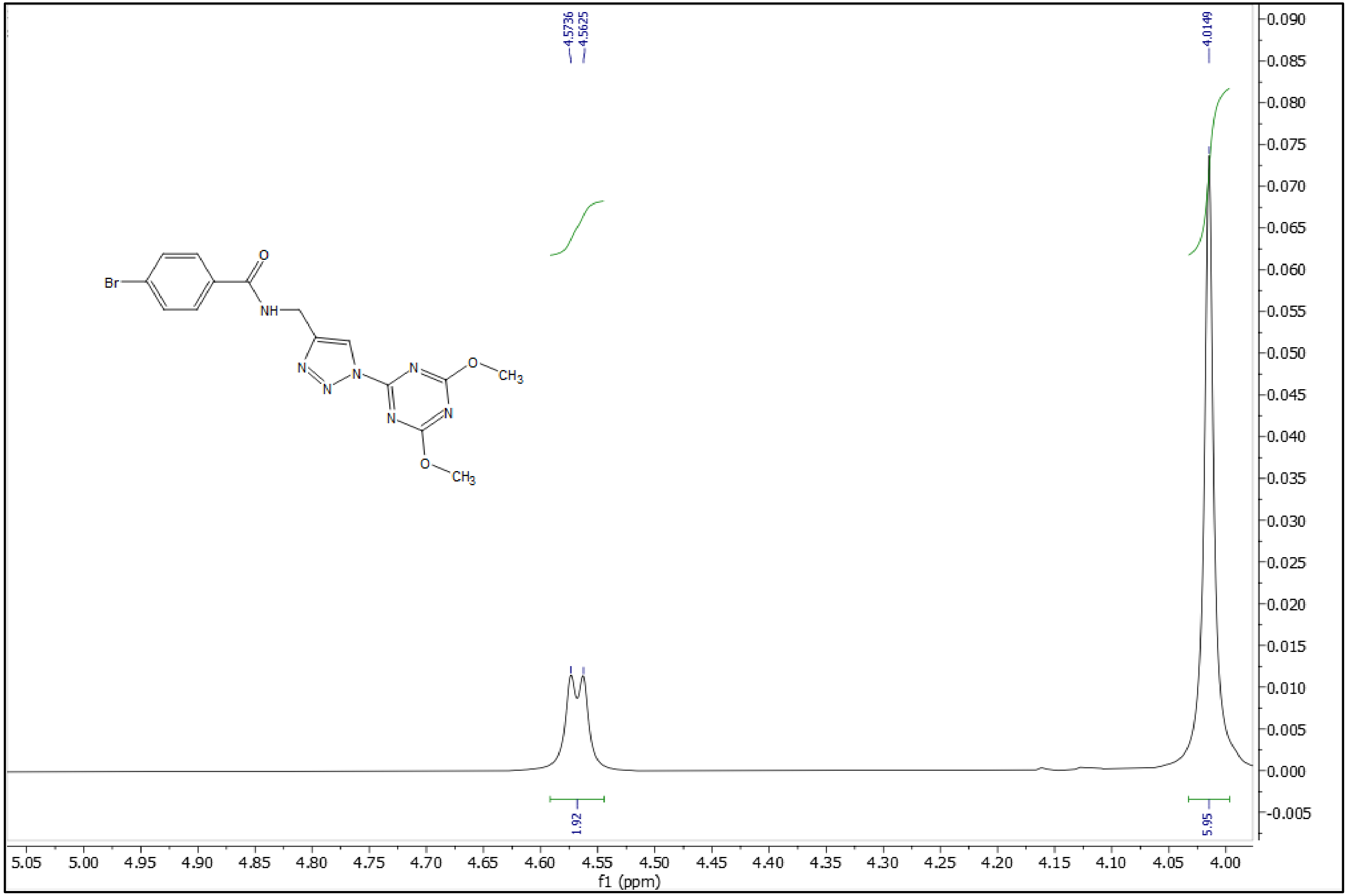

Figure S22: ^{1}H-NMR (500 MHz, DMSO-*d₆*) spectrum of 4-bromo-*N*-((1-(4,6-dimethoxy-1,3,5-triazin-2-yl)-1*H*-1,2,3-triazol-4-yl) methyl) benzamide

(3c)

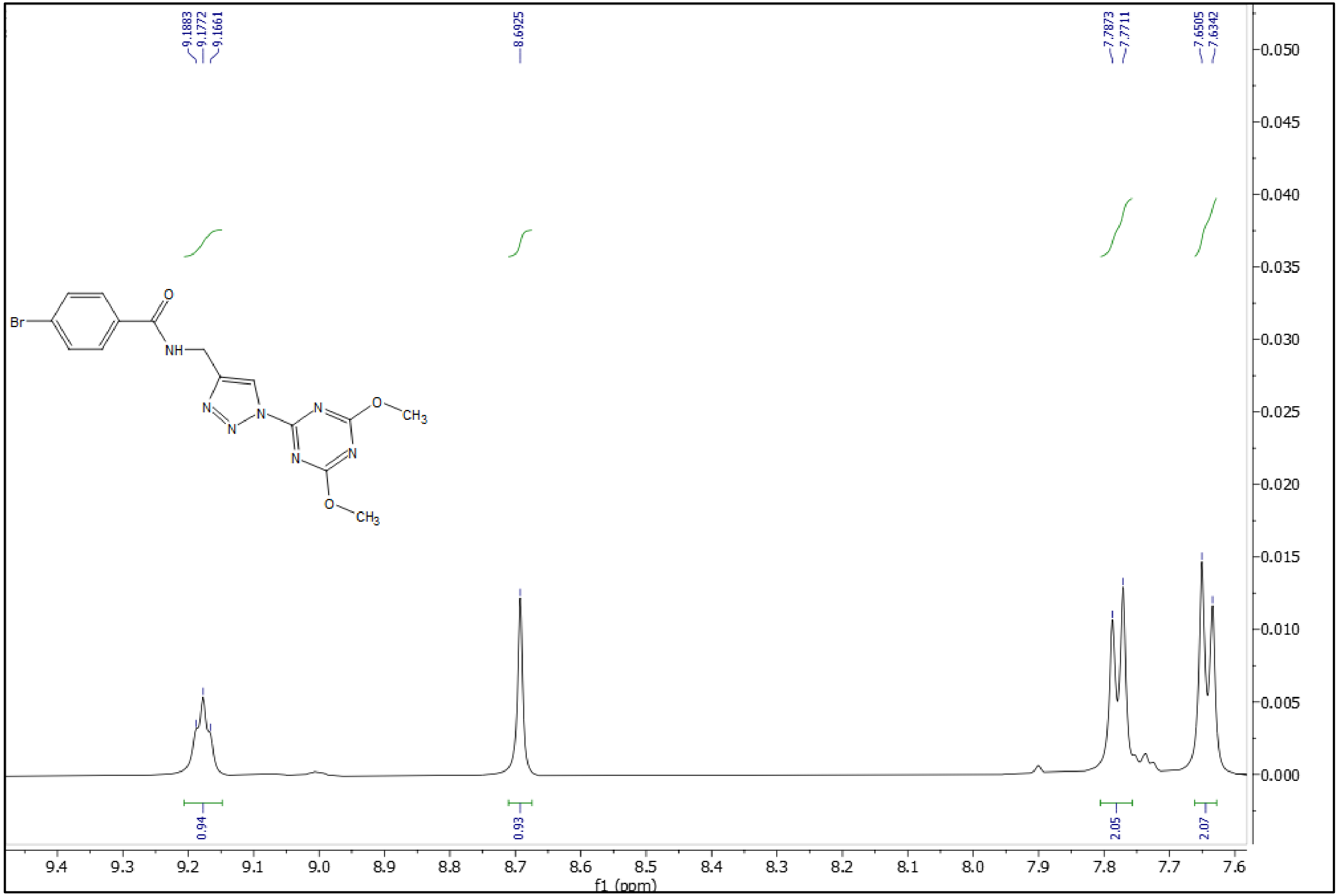

Figure S23: ^{1}H-NMR (500 MHz, DMSO-d_6) spectrum of 4-bromo-*N*-((1-(4,6-dimethoxy-1,3,5-triazin-2-yl)-1*H*-1,2,3-triazol-4-yl) methyl) benzamide **(3c)**

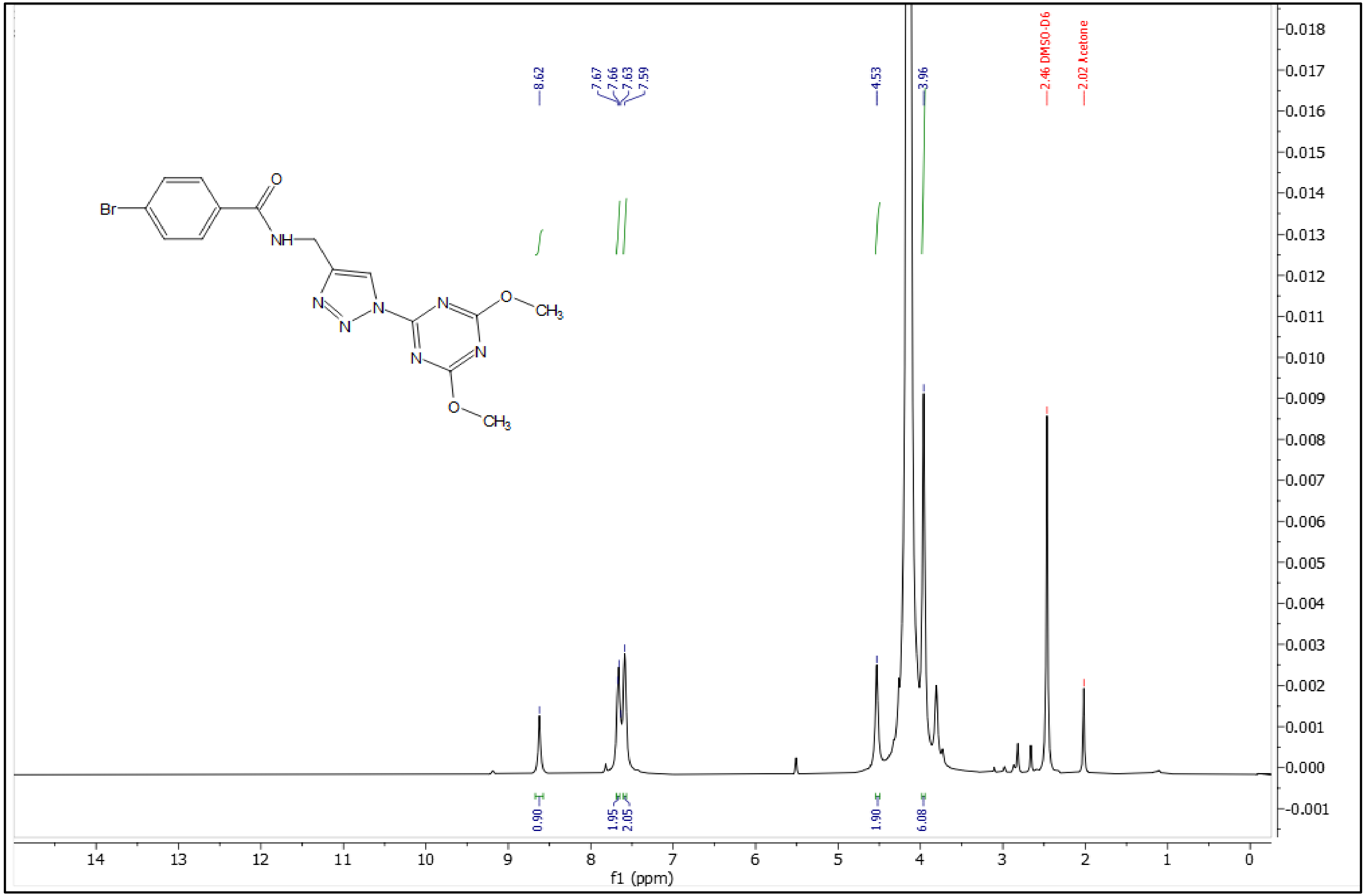

Figure S24: ^{1}H-NMR (500 MHz, DMSO-d_6, D_2O) spectrum of 4-bromo-*N*-((1-(4,6-dimethoxy-1,3,5-triazin-2-yl)-1*H*-1,2,3-triazol-4-yl) methyl) benzamide **(3c)**

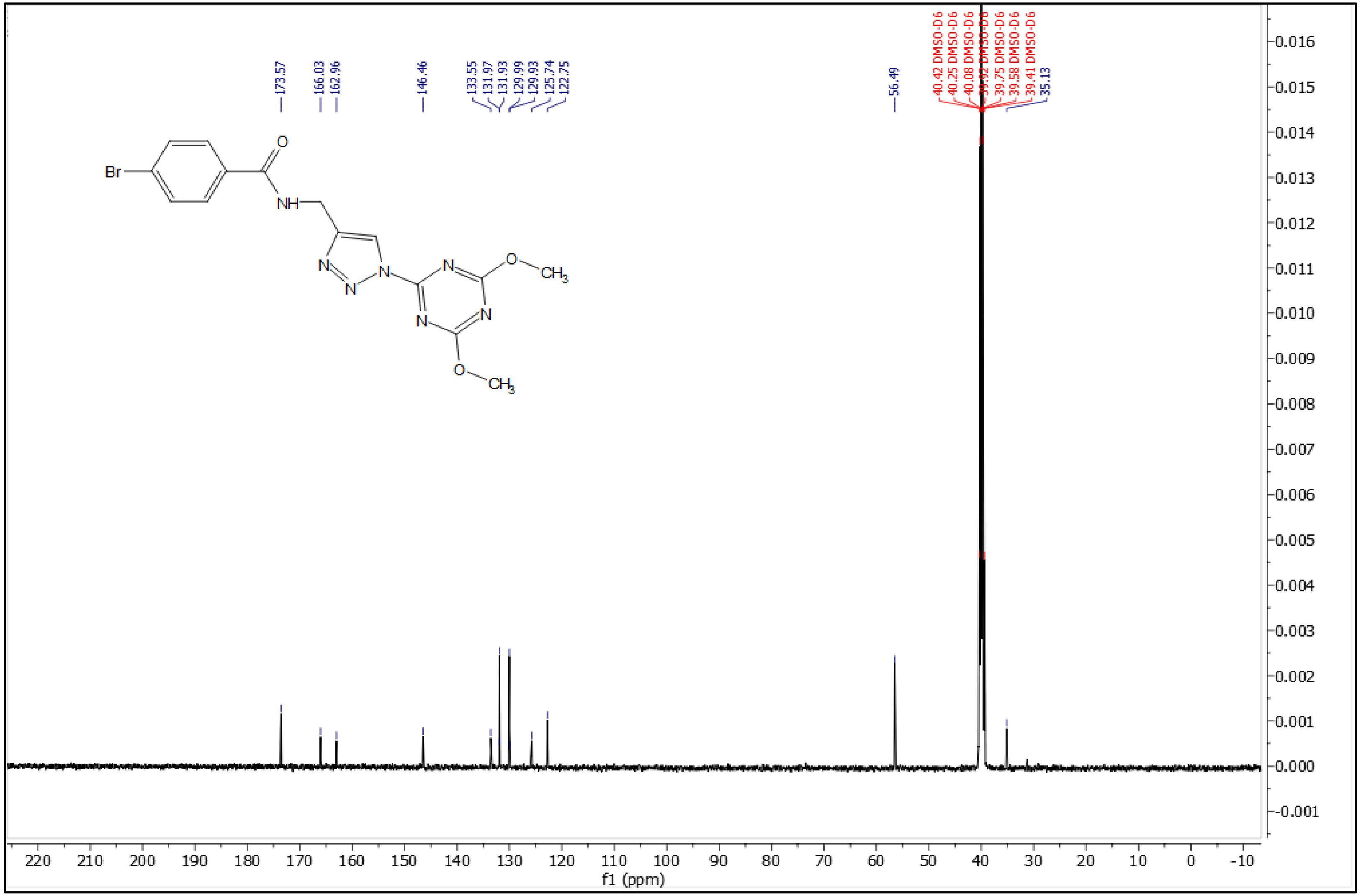

Figure S25: ^{13}C-NMR (DMSO-d_6) spectrum of 4-bromo-*N*-((1-(4,6-dimethoxy-1,3,5-triazin-2-yl)-1*H*-1,2,3-triazol-4-yl) methyl) benzamide **(3c)**

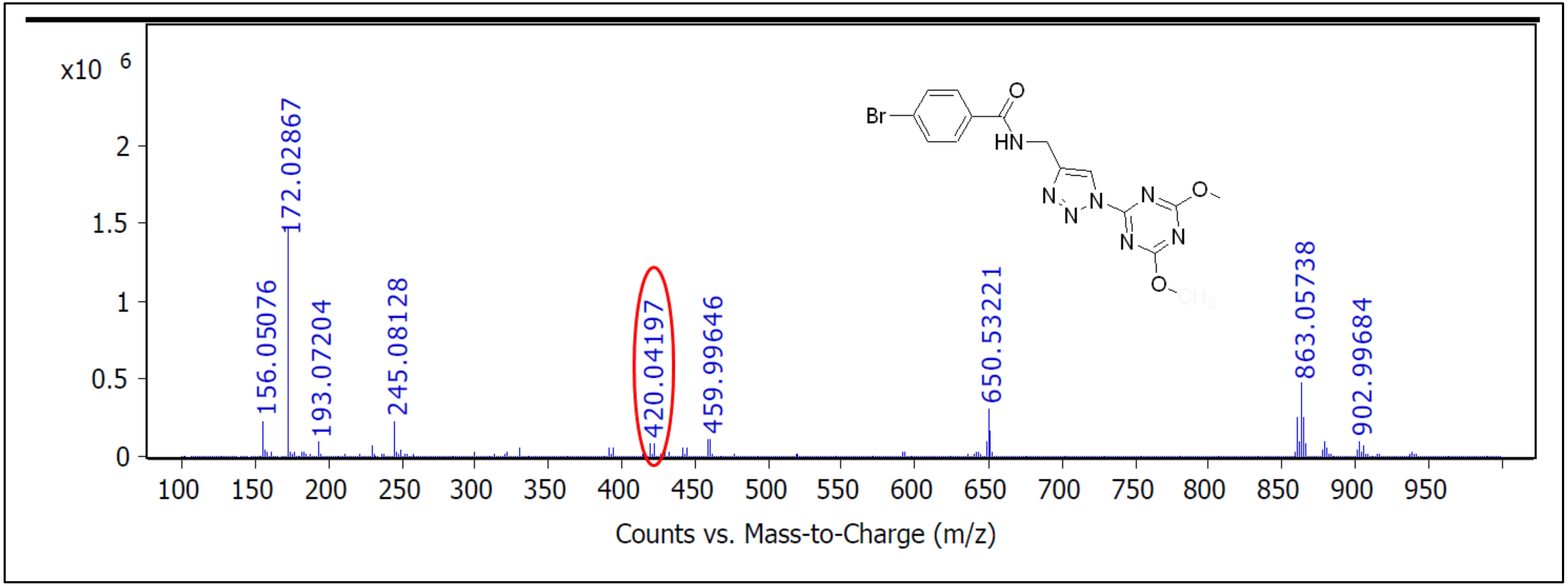

Figure S26: HRMS of 4-bromo-*N*-((1-(4,6-dimethoxy-1,3,5-triazin-2-yl)-1*H*-1,2,3-triazol-4-yl) methyl) benzamide **(3c)**

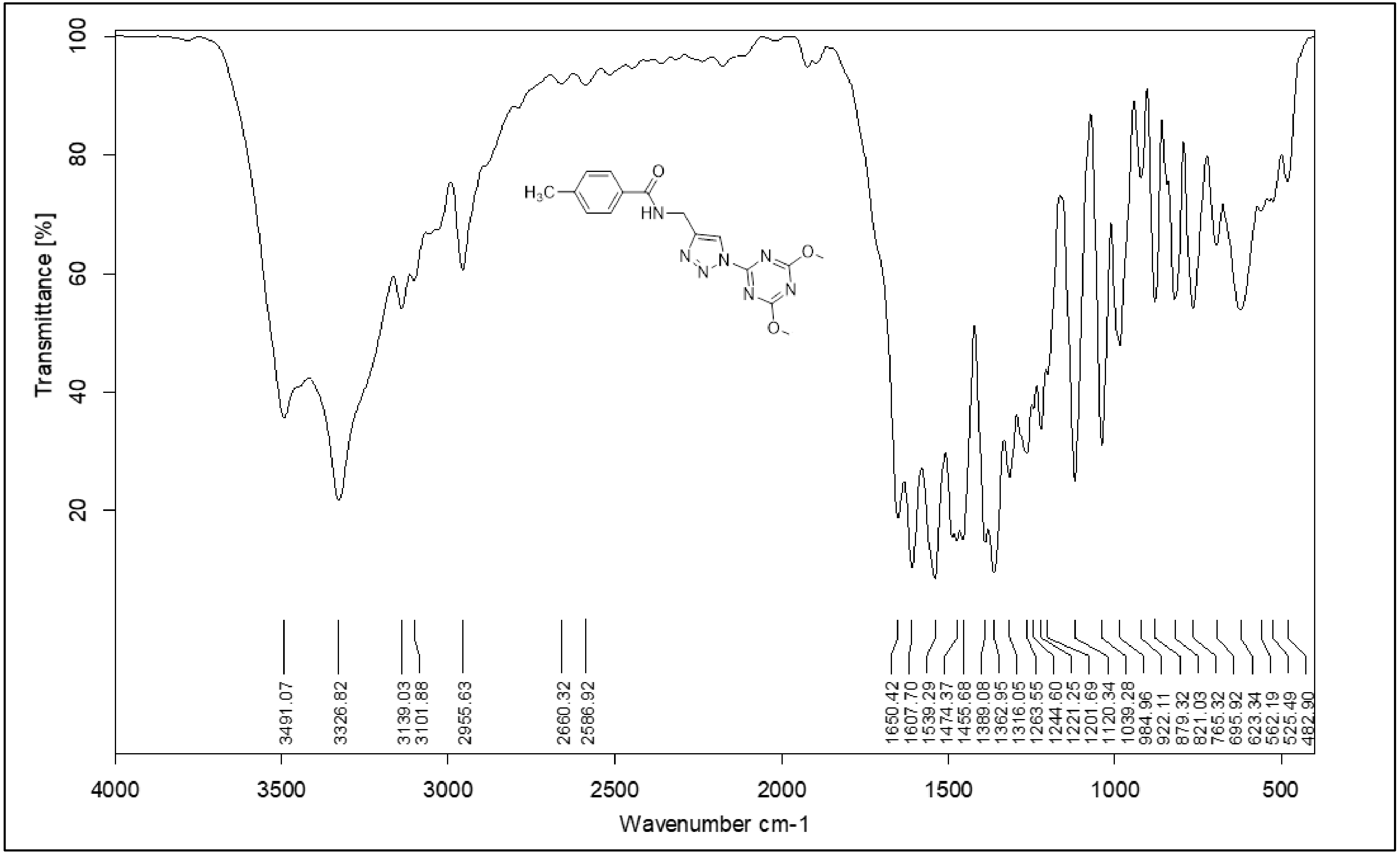

Figure S27: IR (KBr) spectrum of *N*-((1-(4,6-dimethoxy-1,3,5-triazin-2-yl)-1*H*-1,2,3-triazol-4-yl) methyl)-4-methylbenzamide **(3d)**

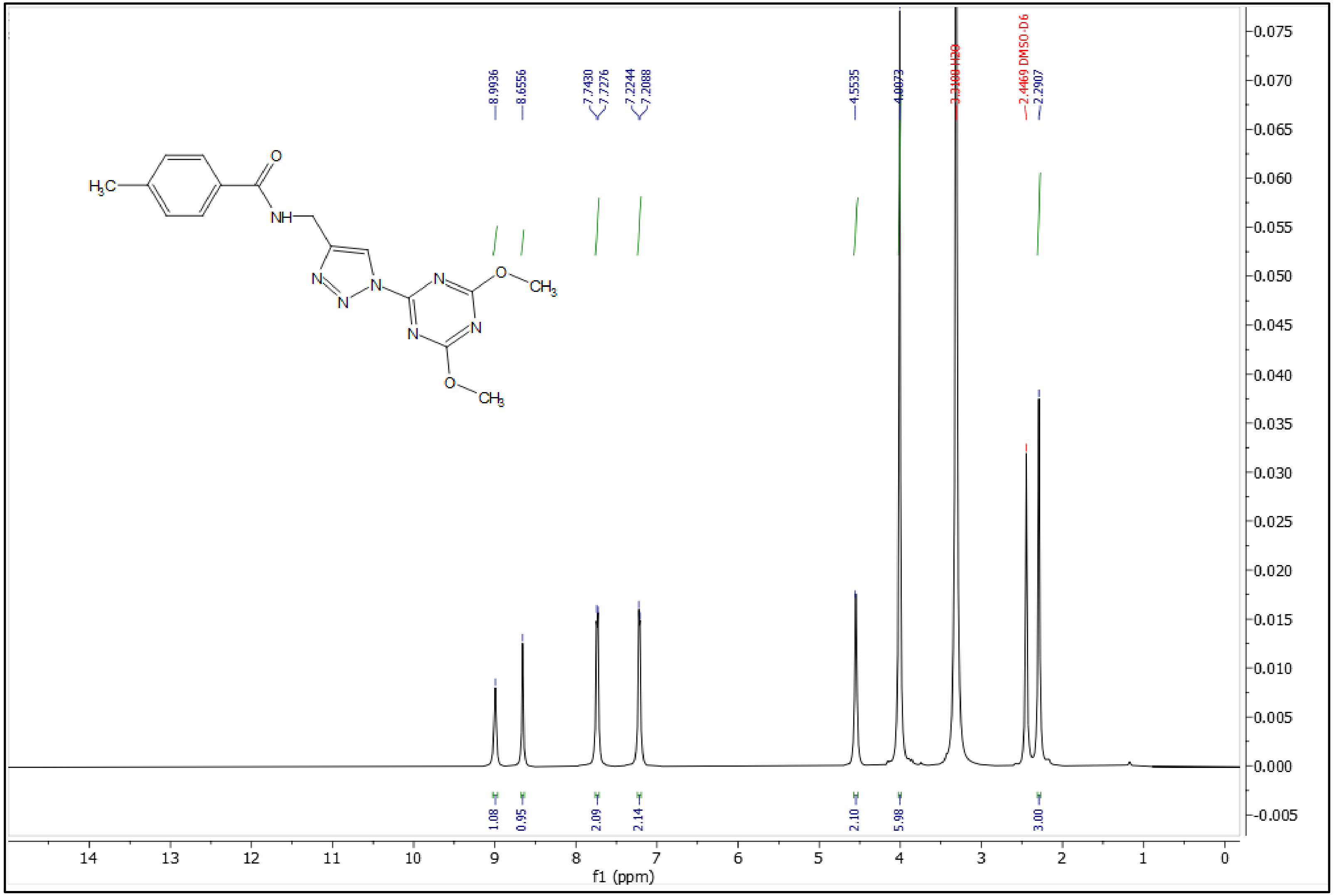

Figure S28: ^{1}H-NMR (500 MHz, DMSO-*d*$_6$) spectrum of *N*-((1-(4,6-dimethoxy-1,3,5-triazin-2-yl)-1*H*-1,2,3-triazol-4-yl) methyl)-4-methylbenzamide **(3d)**

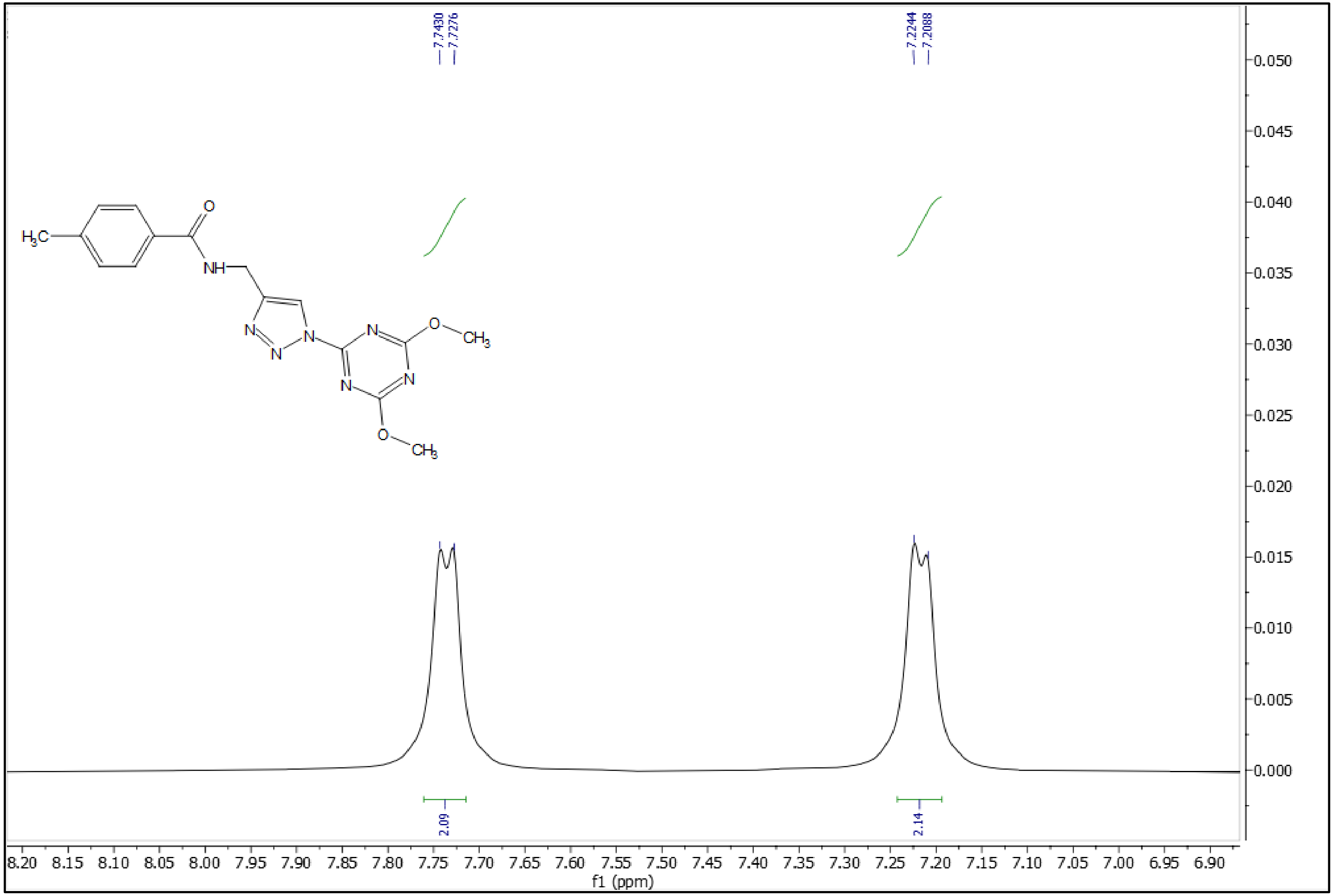

Figure S29: ^{1}H-NMR (500 MHz, DMSO-d_6) spectrum of *N*-((1-(4,6-dimethoxy-1,3,5-triazin-2-yl)-1*H*-1,2,3-triazol-4-yl) methyl)-4-methylbenzamide **(3d)**

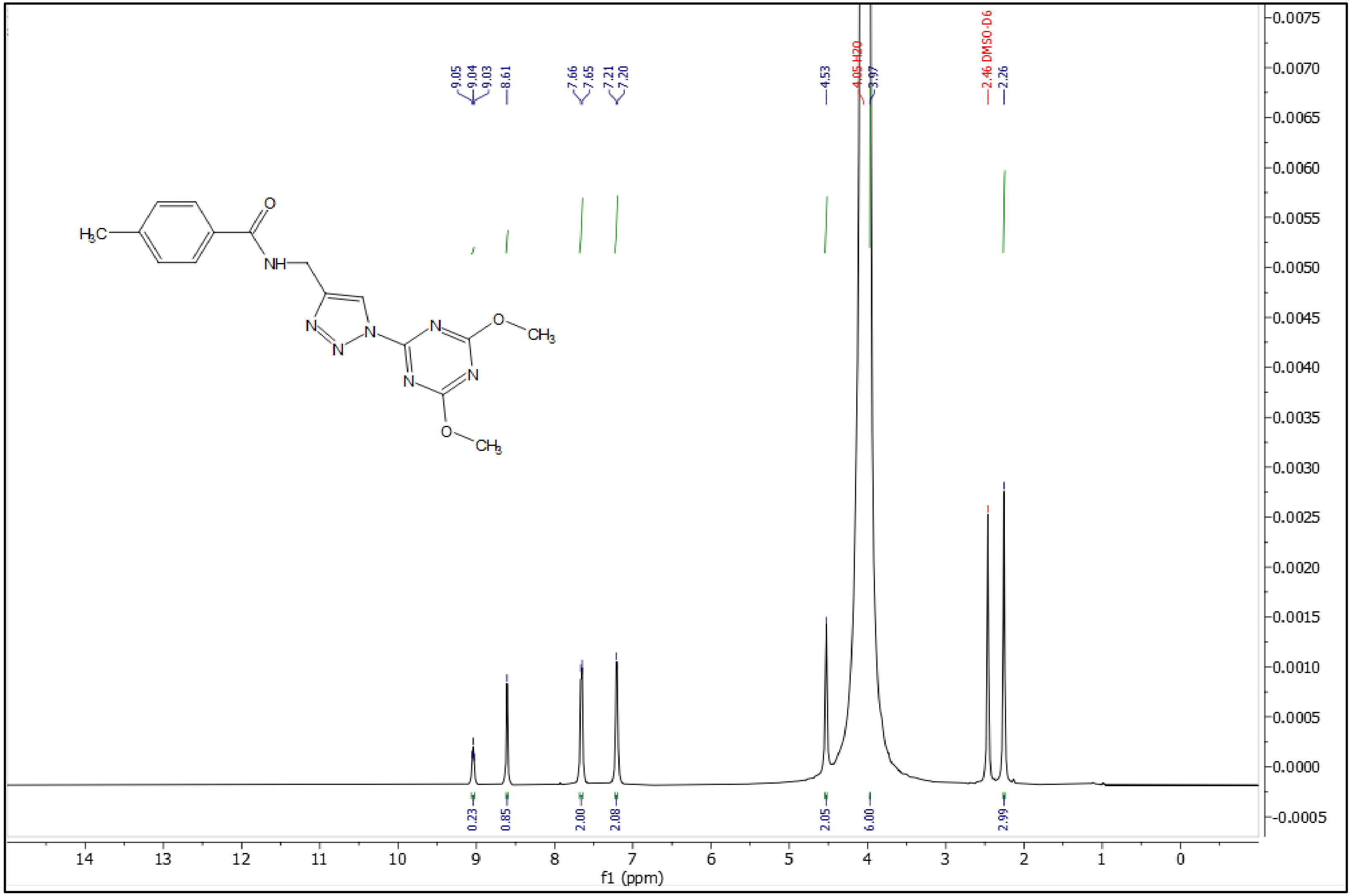

Figure S30: ^{1}H-NMR (500 MHz, DMSO-d_6, D_2O) spectrum of *N*-((1-(4,6-dimethoxy-1,3,5-triazin-2-yl)-1*H*-1,2,3-triazol-4-yl) methyl)-4-methylbenzamide **(3d)**

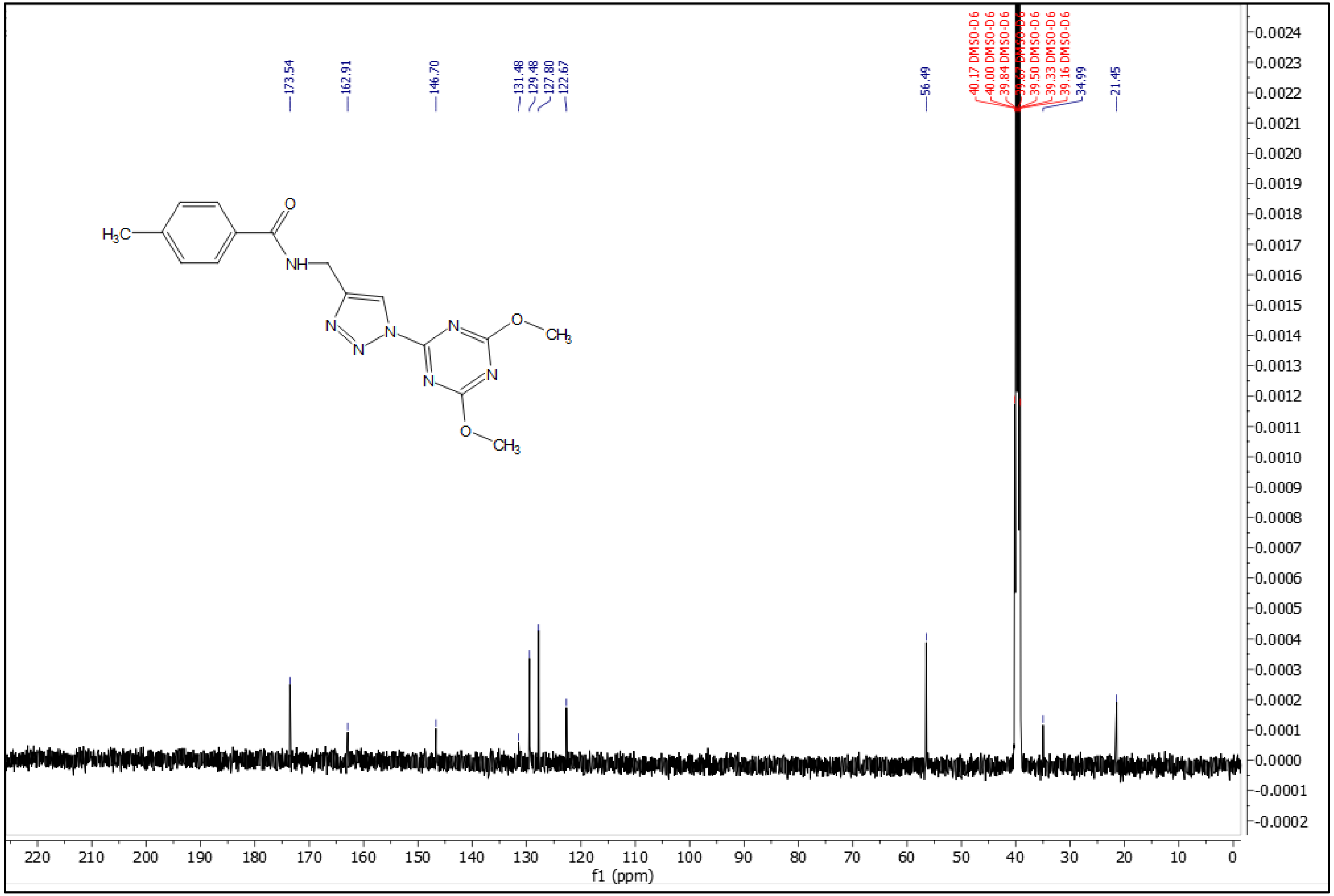

Figure S31: ^{13}C-NMR (DMSO-d_6) spectrum of *N*-((1-(4,6-dimethoxy-1,3,5-triazin-2-yl)-1*H*-1,2,3-triazol-4-yl) methyl)-4-methylbenzamide **(3d)**

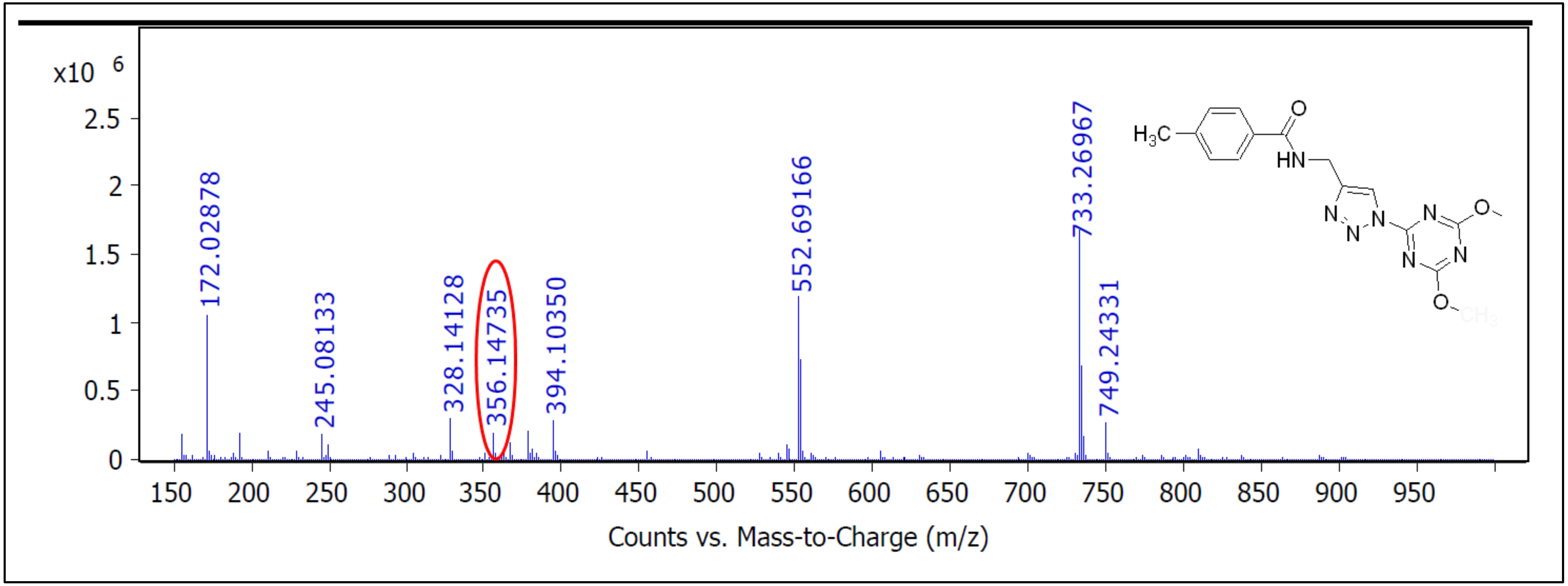

Figure S32: HRMS of *N*-((1-(4,6-dimethoxy-1,3,5-triazin-2-yl)-1*H*-1,2,3-triazol-4-yl) methyl)-4-methylbenzamide **(3d)**

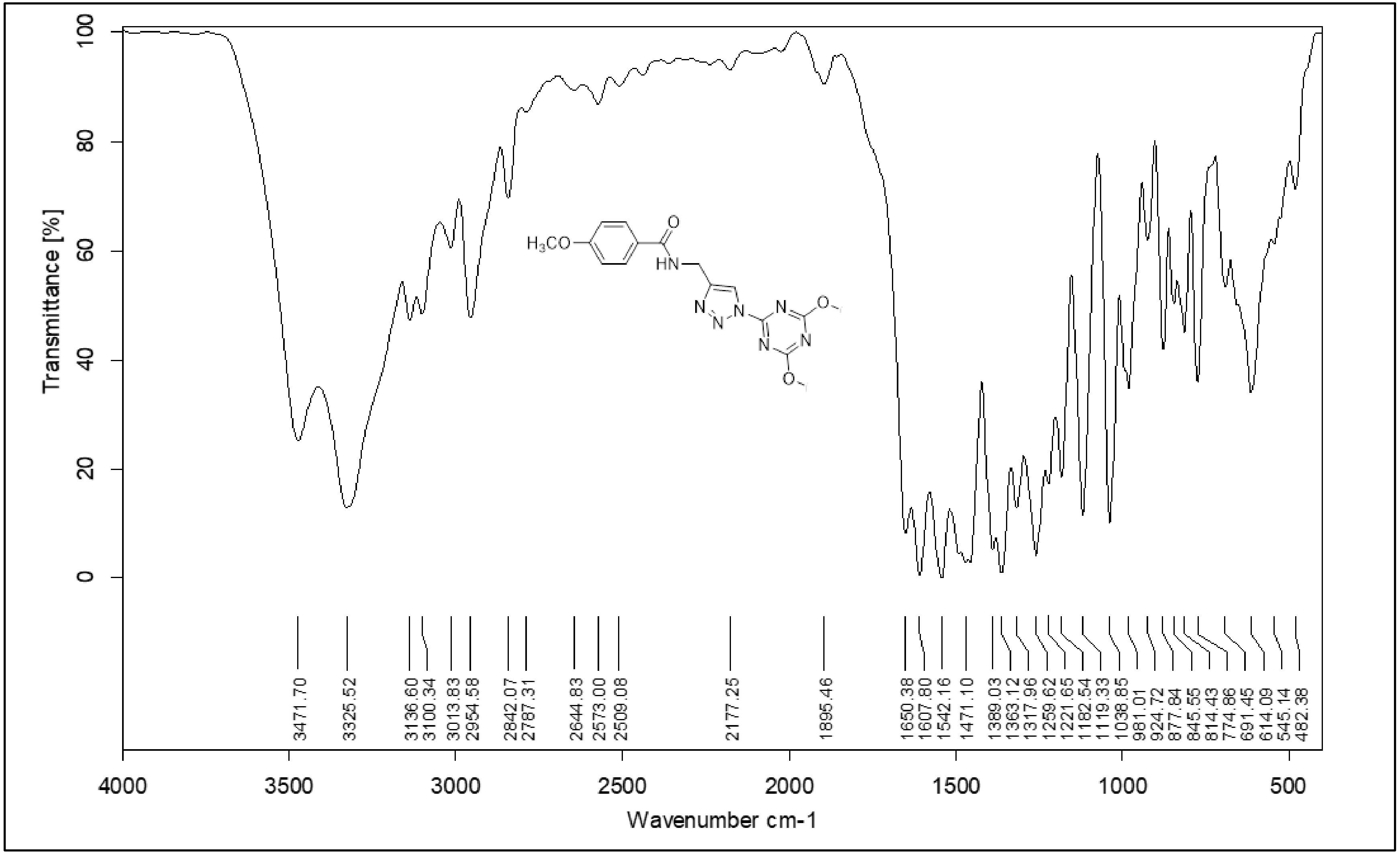

Figure S33: IR (KBr) spectrum of *N*-((1-(4,6-dimethoxy-1,3,5-triazin-2-yl)-1*H*-1,2,3-triazol-4-yl) methyl)-4-methoxybenzamide **(3e)**

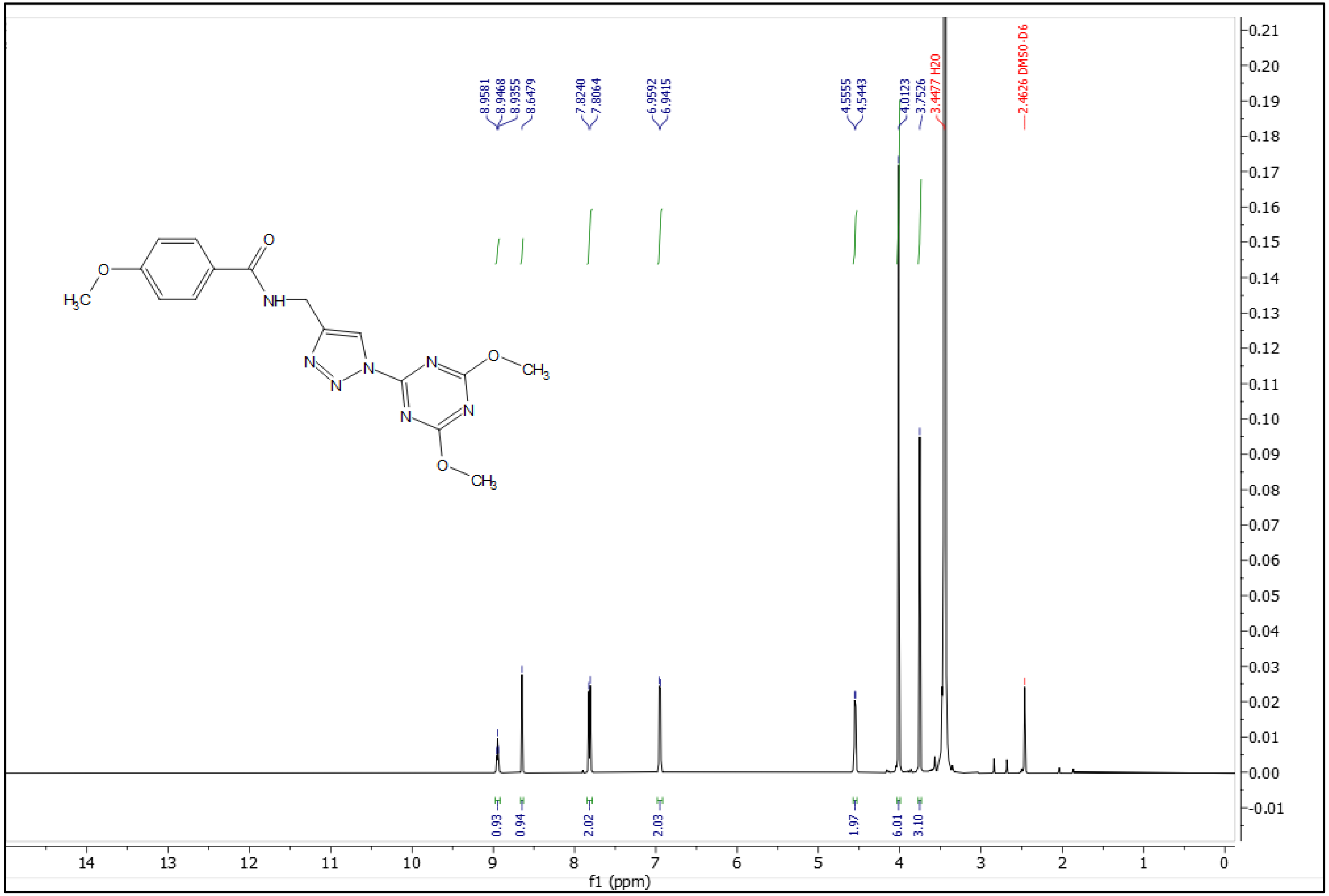

Figure S34: ^{1}H-NMR (500 MHz, DMSO-d_6) spectrum of *N*-((1-(4,6-dimethoxy-1,3,5-triazin-2-yl)-1*H*-1,2,3-triazol-4-yl) methyl)-4-methoxybenzamide **(3e)**

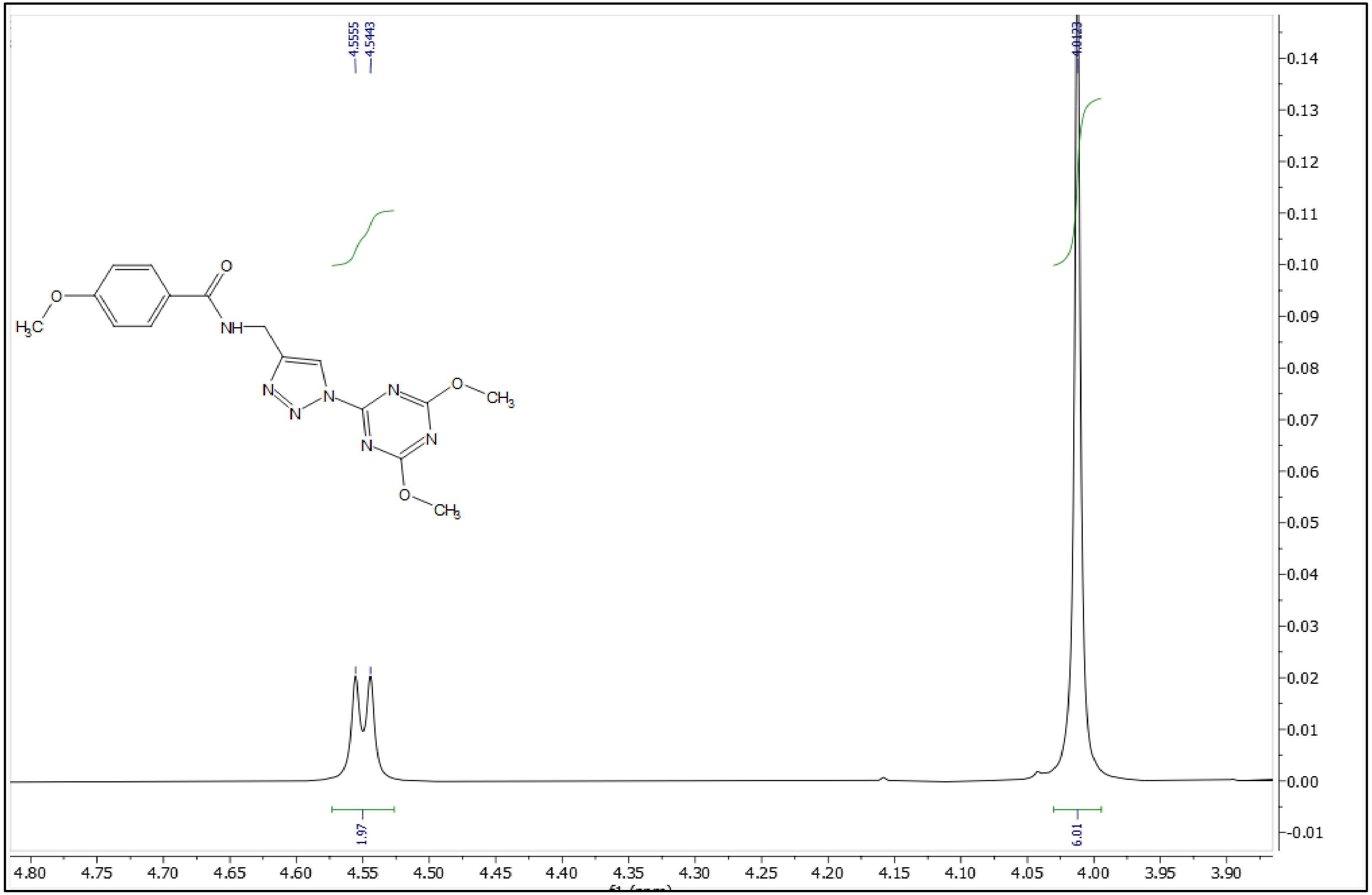

Figure S35: ^{1}H-NMR (500 MHz, DMSO-d_6) spectrum of *N*-((1-(4,6-dimethoxy-1,3,5-triazin-2-yl)-1*H*-1,2,3-triazol-4-yl) methyl)-4-methoxybenzamide **(3e)**

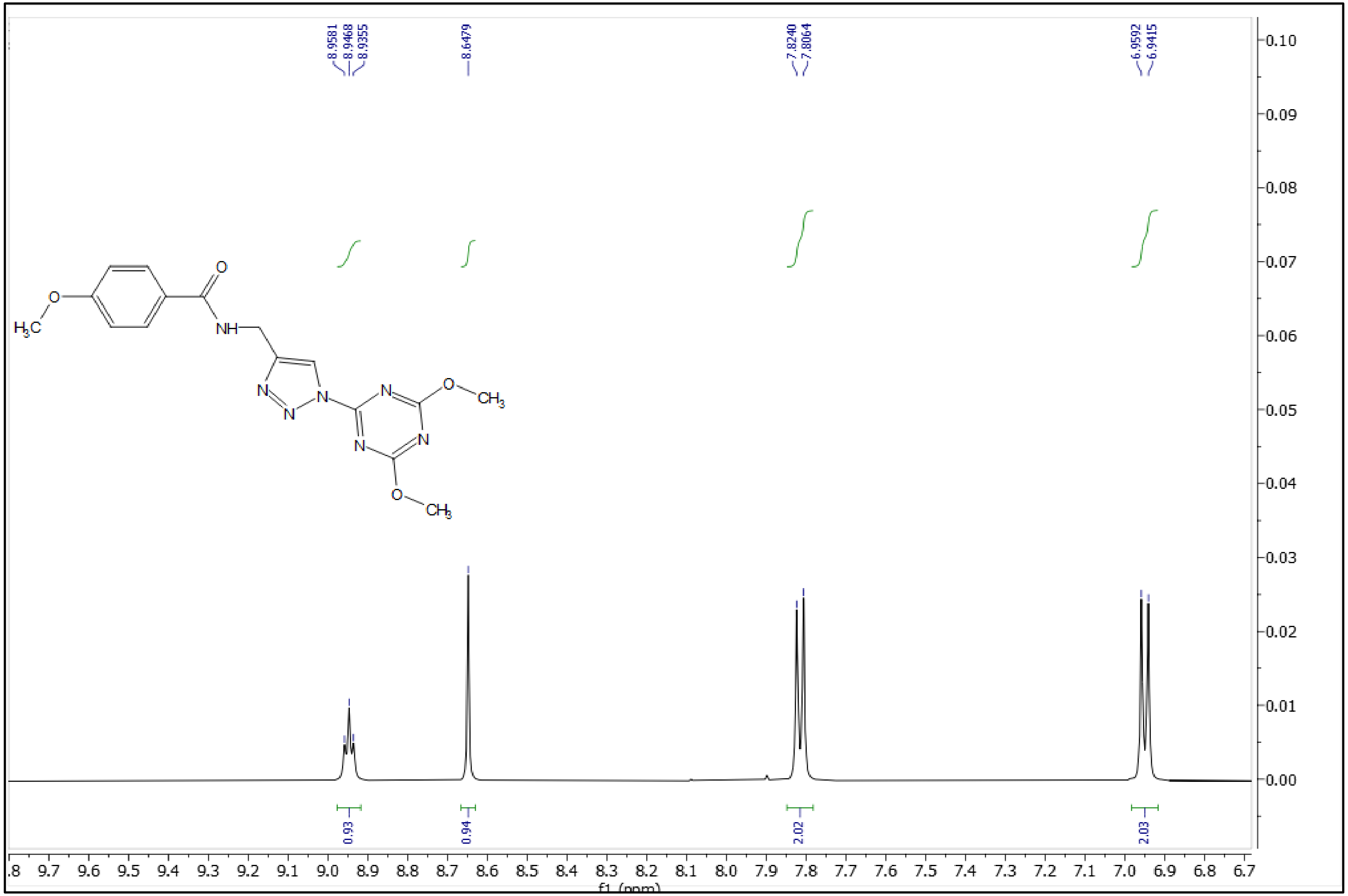

Figure S36: ^{1}H-NMR (500 MHz, DMSO-d_6) spectrum of *N*-((1-(4,6-dimethoxy-1,3,5-triazin-2-yl)-1*H*-1,2,3-triazol-4-yl) methyl)-4-methoxybenzamide **(3e)**

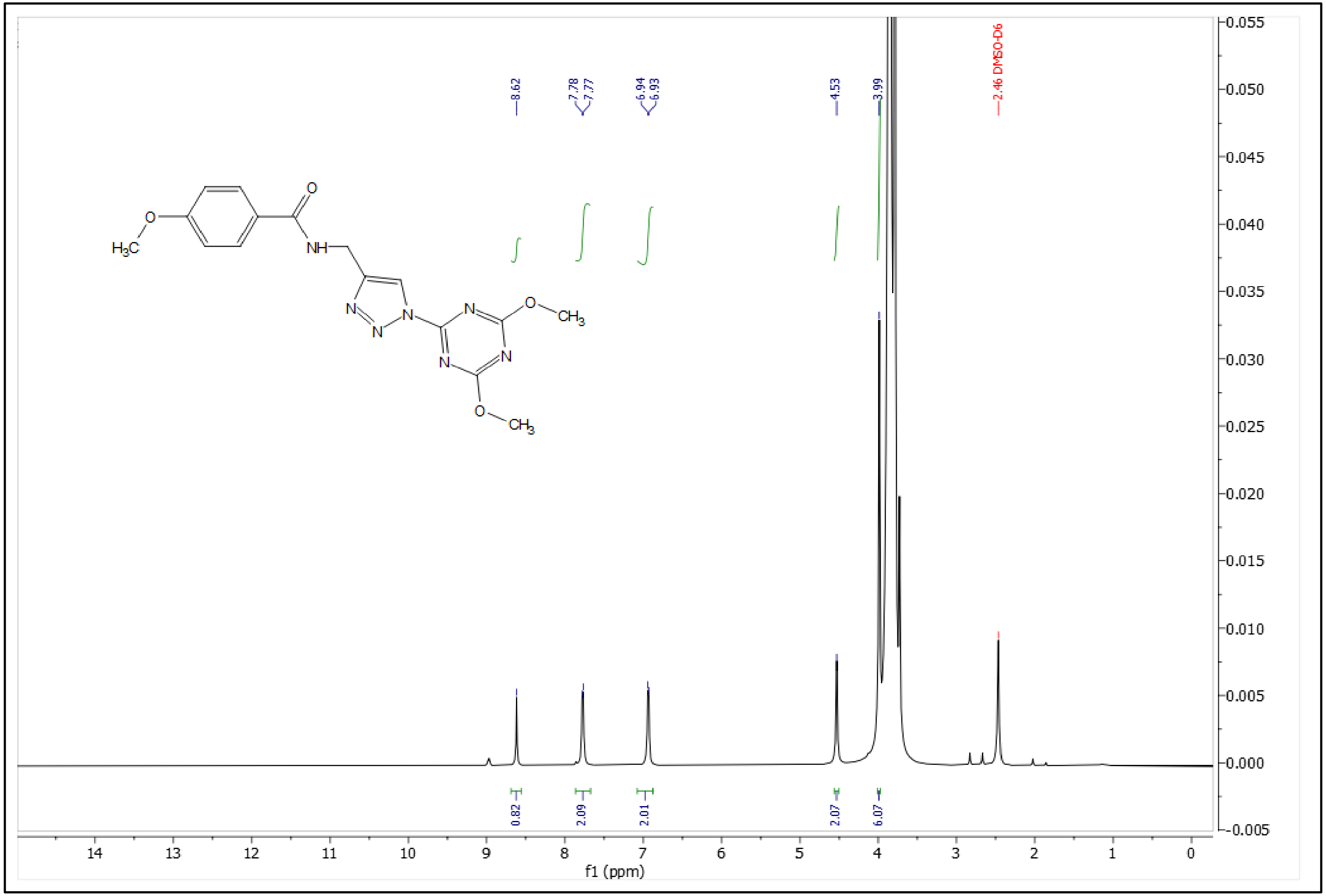

Figure S37: ^{1}H-NMR (500 MHz, DMSO-d_6, D_2O) spectrum of *N*-((1-(4,6-dimethoxy-1,3,5-triazin-2-yl)-1*H*-1,2,3-triazol-4-yl) methyl)-4-methoxybenzamide **(3e)**

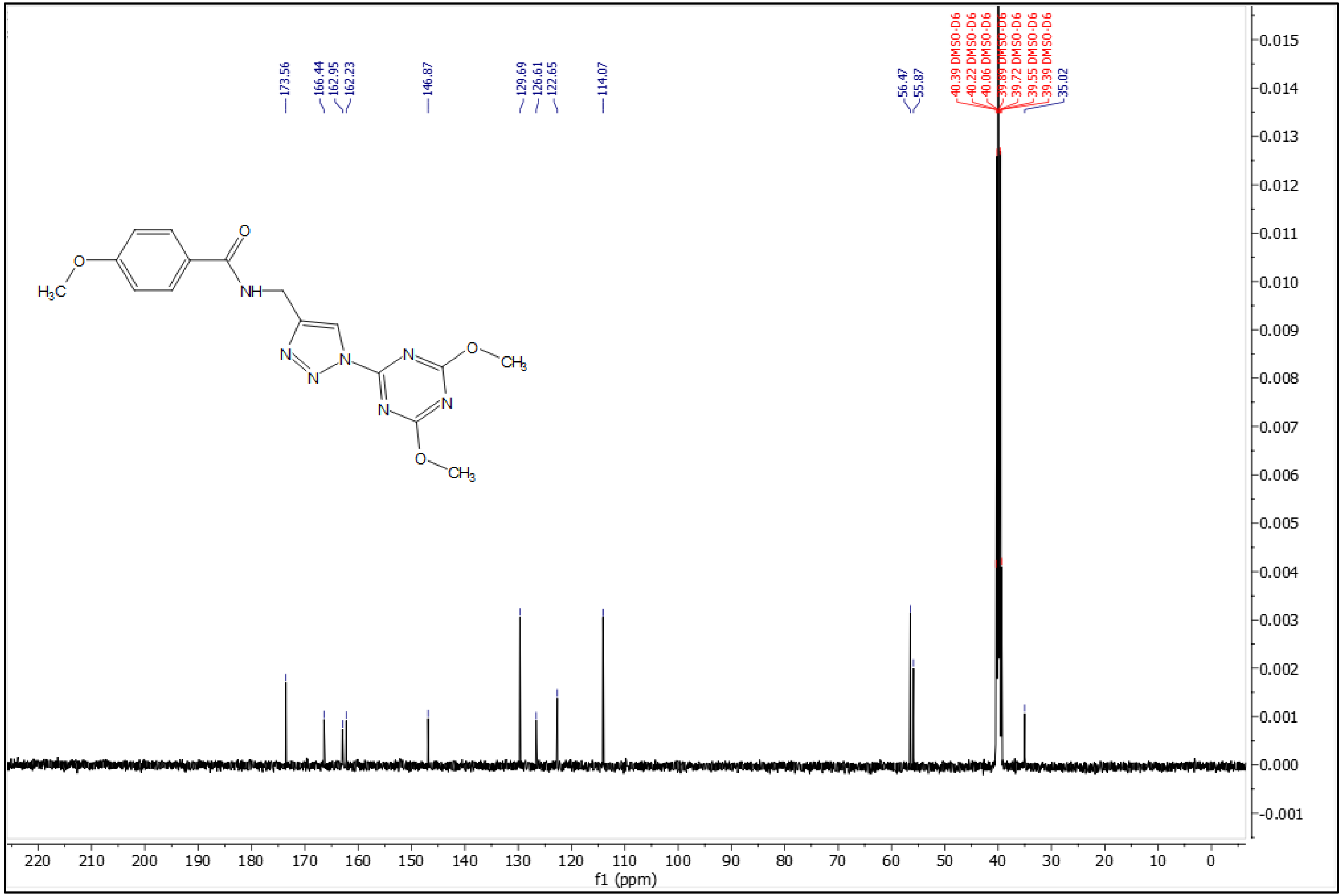

Figure S38: ^{13}C-NMR (DMSO-d_6) spectrum of *N*-((1-(4,6-dimethoxy-1,3,5-triazin-2-yl)-1*H*-1,2,3-triazol-4-yl) methyl)-4-methoxybenzamide **(3e)**

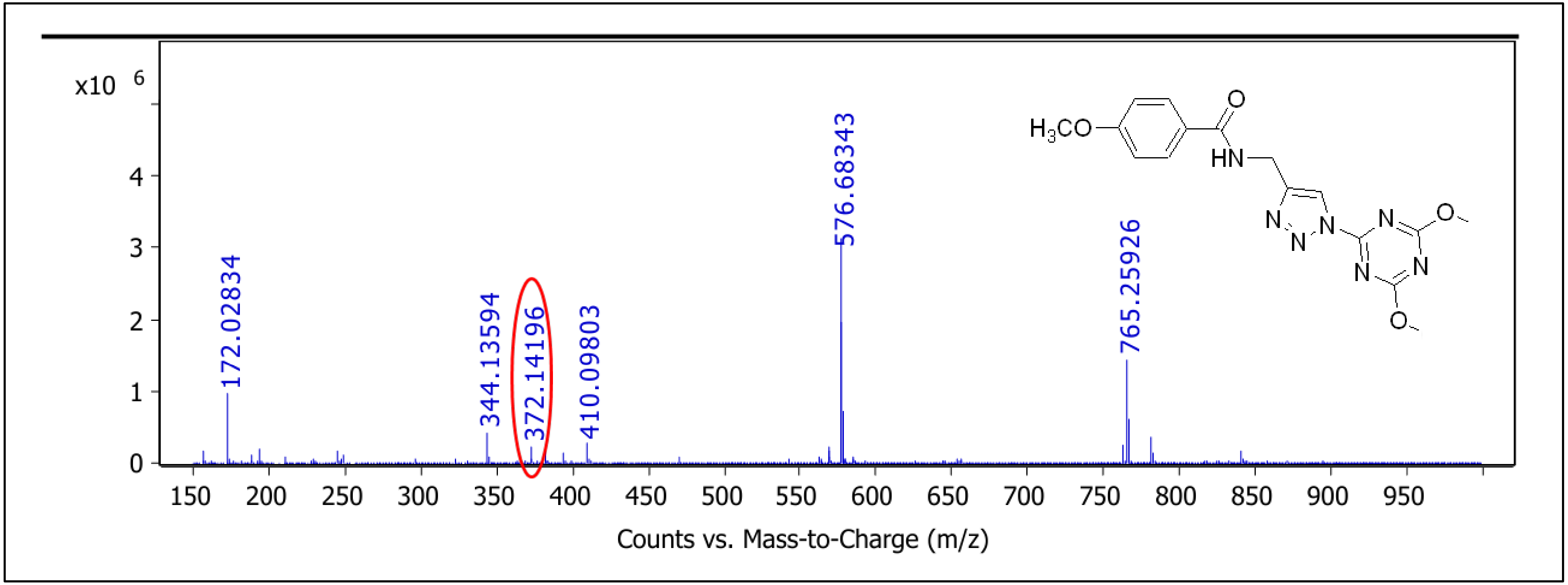

Figure S39: HRMS of *N*-((1-(4,6-dimethoxy-1,3,5-triazin-2-yl)-1*H*-1,2,3-triazol-4-yl) methyl)-4-methoxybenzamide **(3e)**

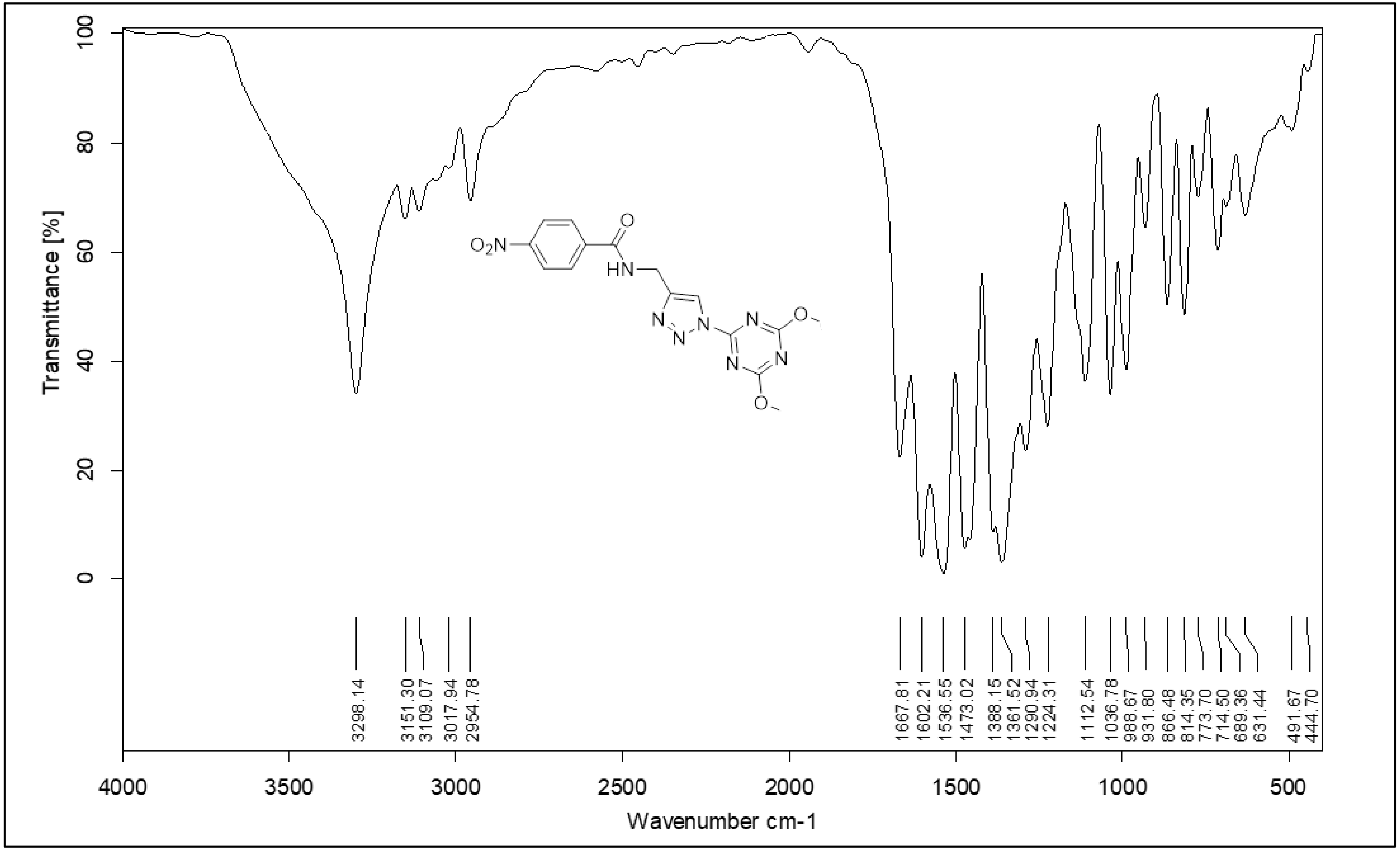

Figure S40: IR (KBr) spectrum of *N*-((1-(4,6-dimethoxy-1,3,5-triazin-2-yl)-1*H*-1,2,3-triazol-4-yl) methyl)-4-nitrobenzamide **(3f)**

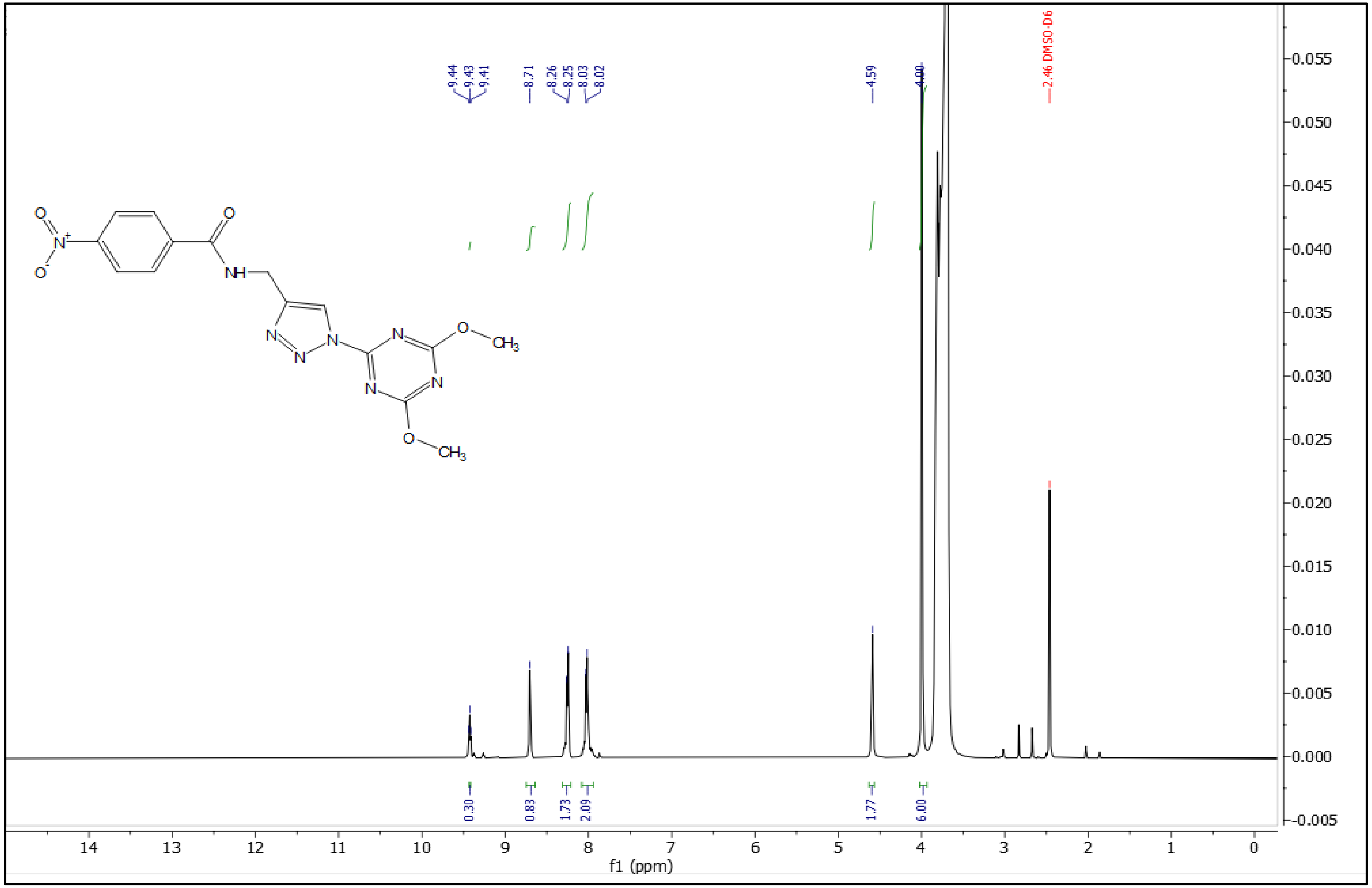

Figure S41: ^{1}H-NMR (500 MHz, DMSO-d_6) spectrum of *N*-((1-(4,6-dimethoxy-1,3,5-triazin-2-yl)-1*H*-1,2,3-triazol-4-yl) methyl)-4-nitrobenzamide **(3f)**

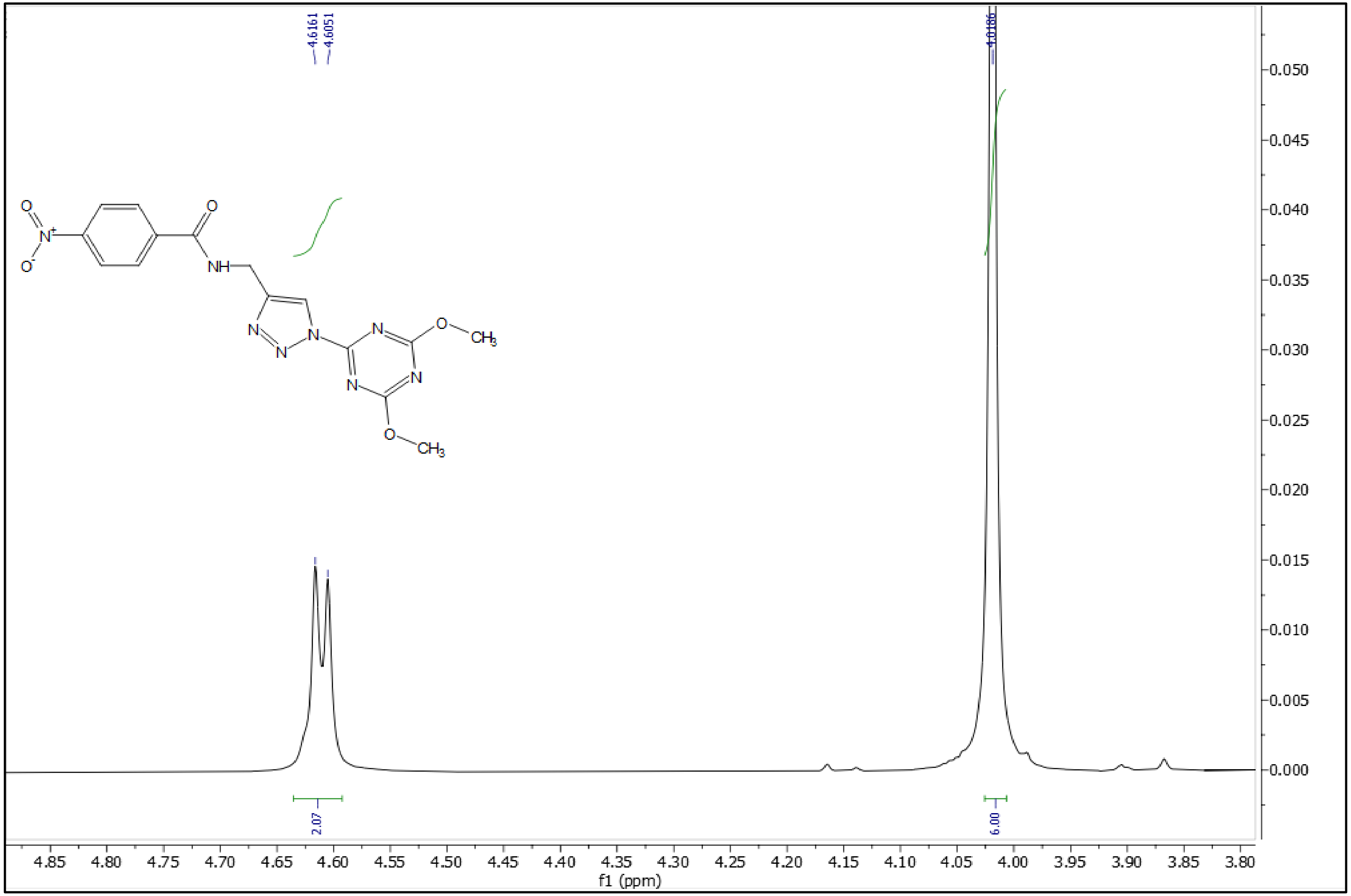

Figure S42: ^{1}H-NMR (500 MHz, DMSO-d_6) spectrum of *N*-((1-(4,6-dimethoxy-1,3,5-triazin-2-yl)-1*H*-1,2,3-triazol-4-yl) methyl)-4-nitrobenzamide **(3f)**

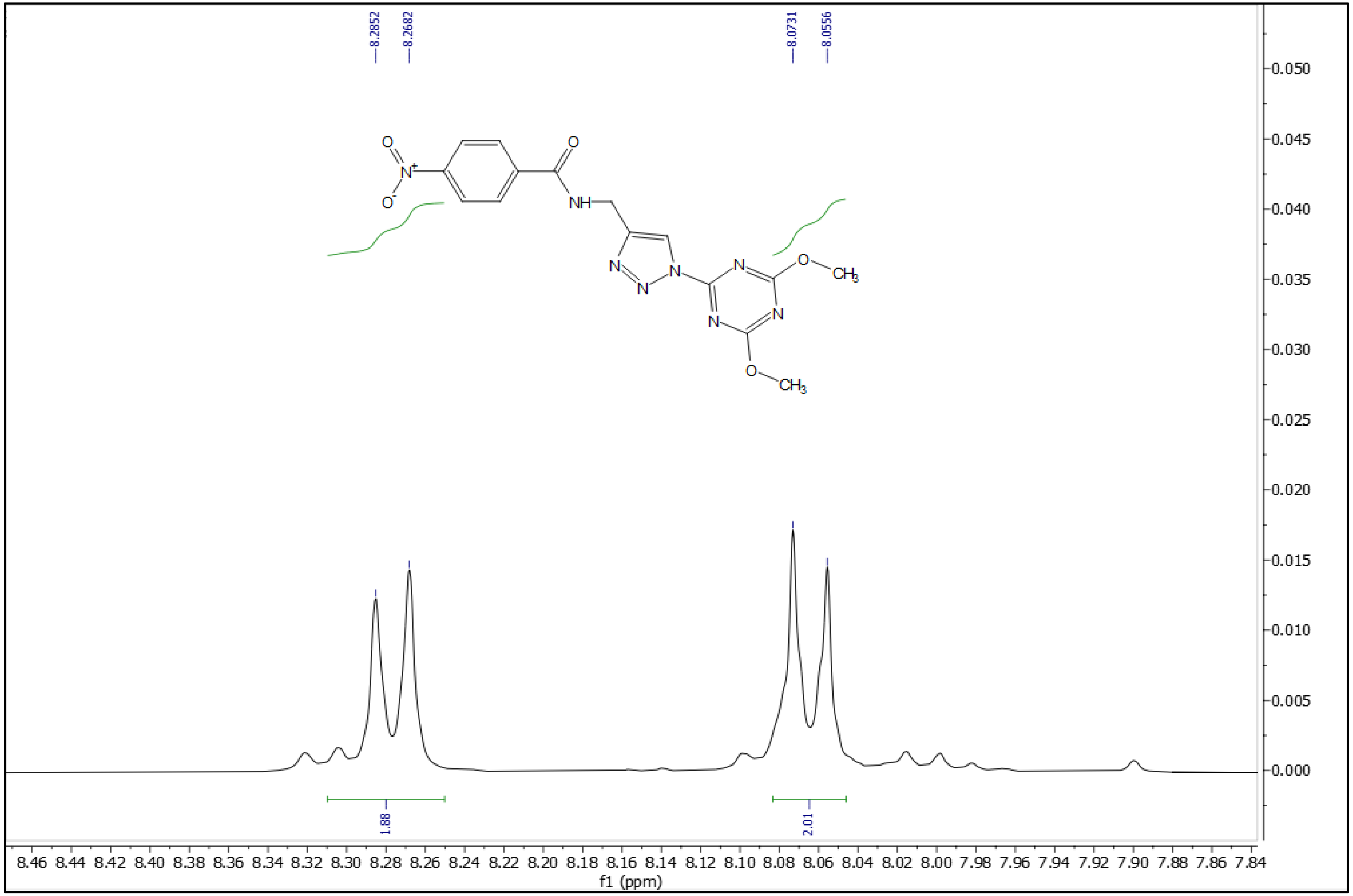

Figure S43: ^{1}H-NMR (500 MHz, DMSO-d_6) spectrum of *N*-((1-(4,6-dimethoxy-1,3,5-triazin-2-yl)-1*H*-1,2,3-triazol-4-yl) methyl)-4-nitrobenzamide **(3f)**

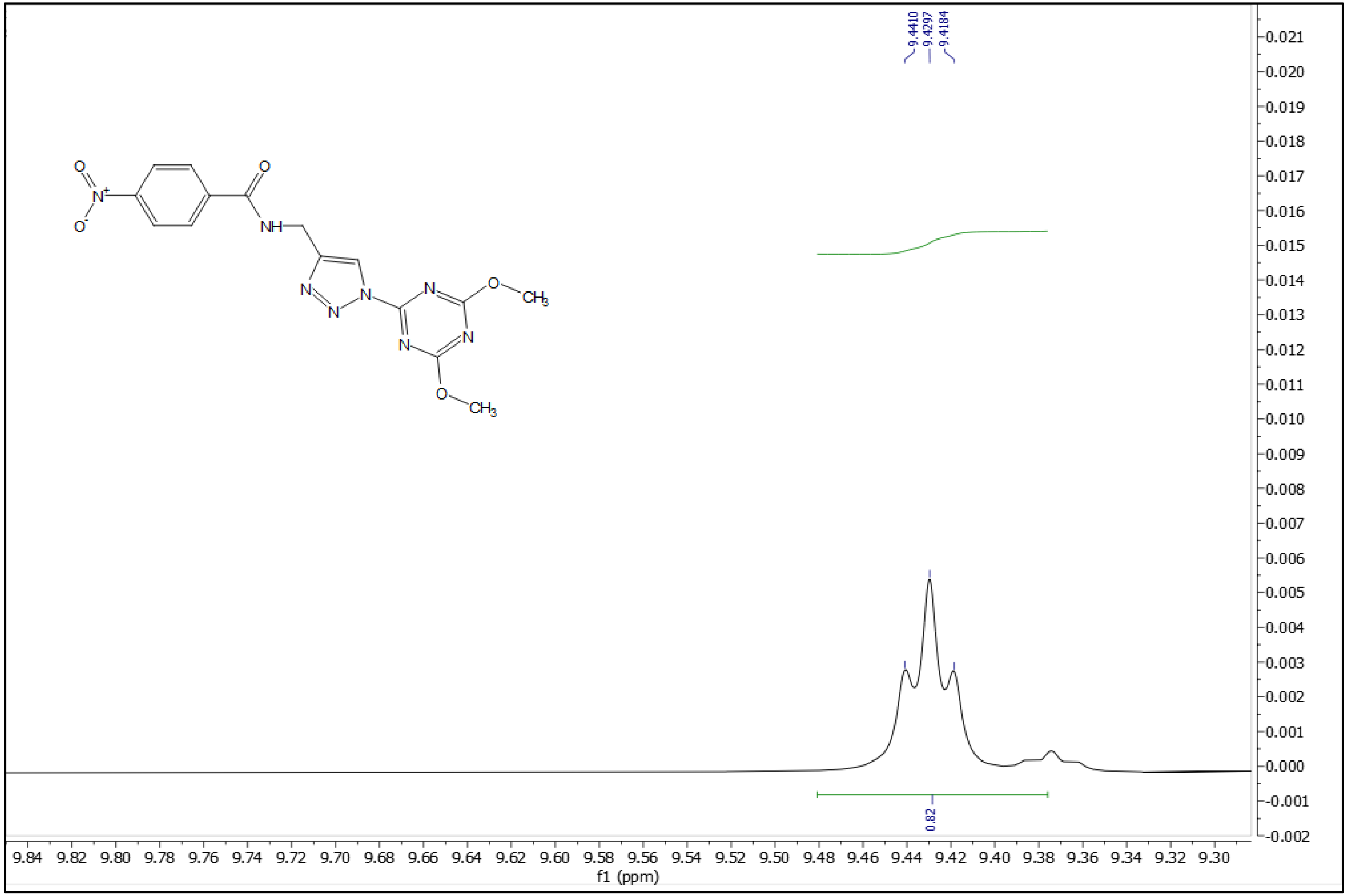

Figure S44: ^{1}H-NMR (500 MHz, DMSO-d_6) spectrum of *N*-((1-(4,6-dimethoxy-1,3,5-triazin-2-yl)-1*H*-1,2,3-triazol-4-yl) methyl)-4-nitrobenzamide

(3f)

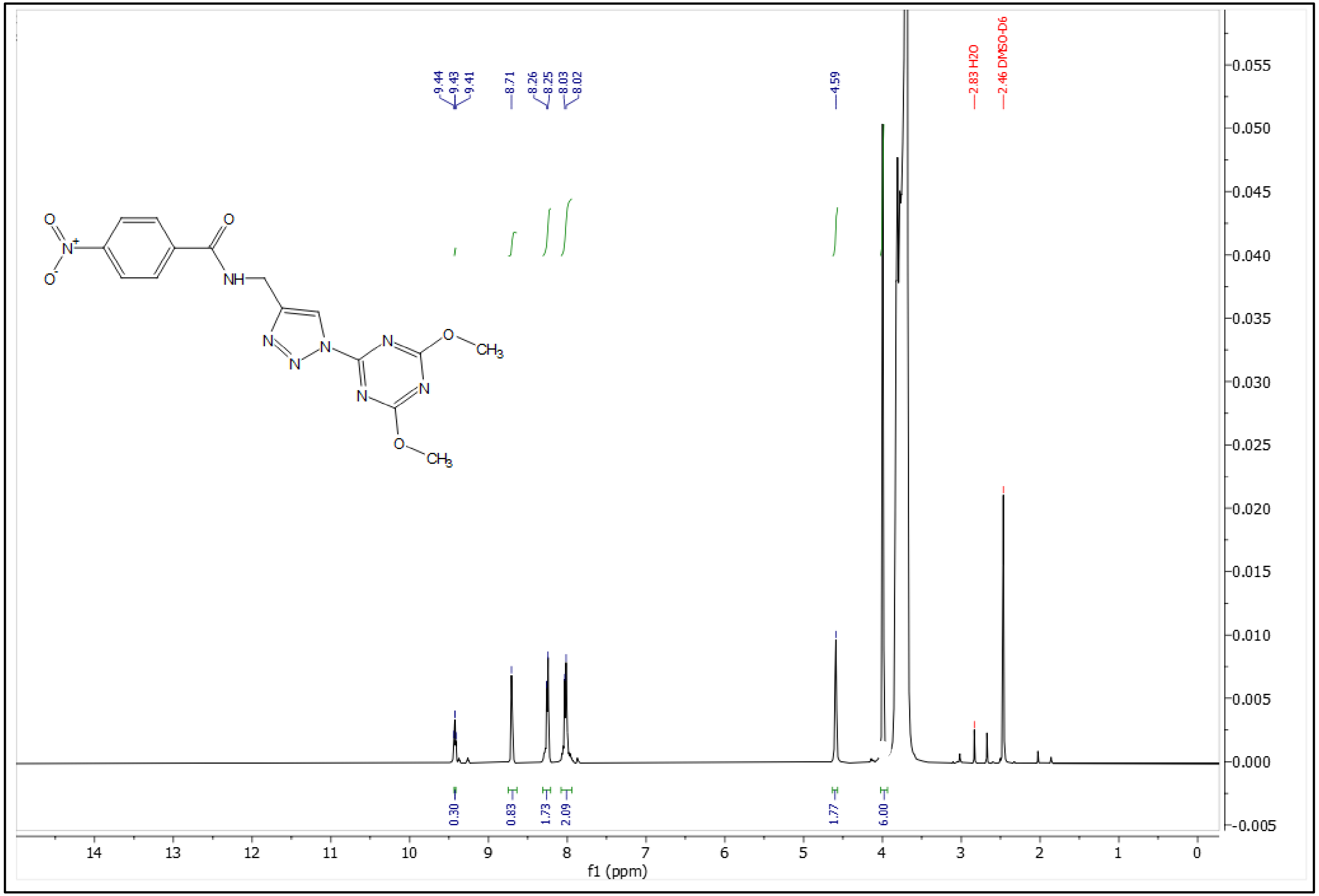

Figure S45: ^{1}H-NMR (500 MHz, DMSO-d_6, D_2O) spectrum of *N*-((1-(4,6-dimethoxy-1,3,5-triazin-2-yl)-1*H*-1,2,3-triazol-4-yl) methyl)-4-nitrobenzamide **(3f).**

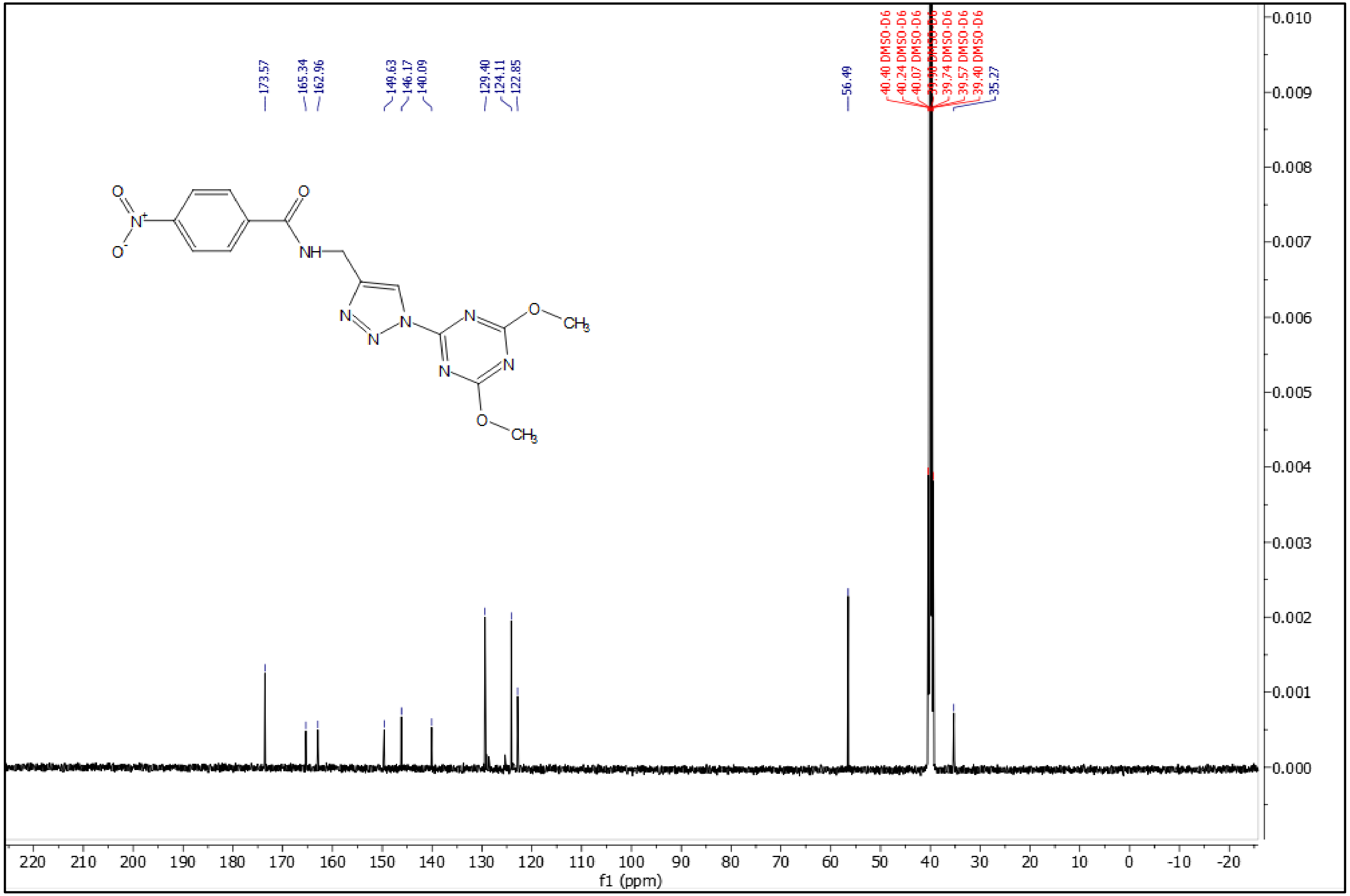

Figure S46: ^{13}C-NMR (DMSO-d_6) spectrum of *N*-((1-(4,6-dimethoxy-1,3,5-triazin-2-yl)-1*H*-1,2,3-triazol-4-yl) methyl)-4-nitrobenzamide **(3f)**

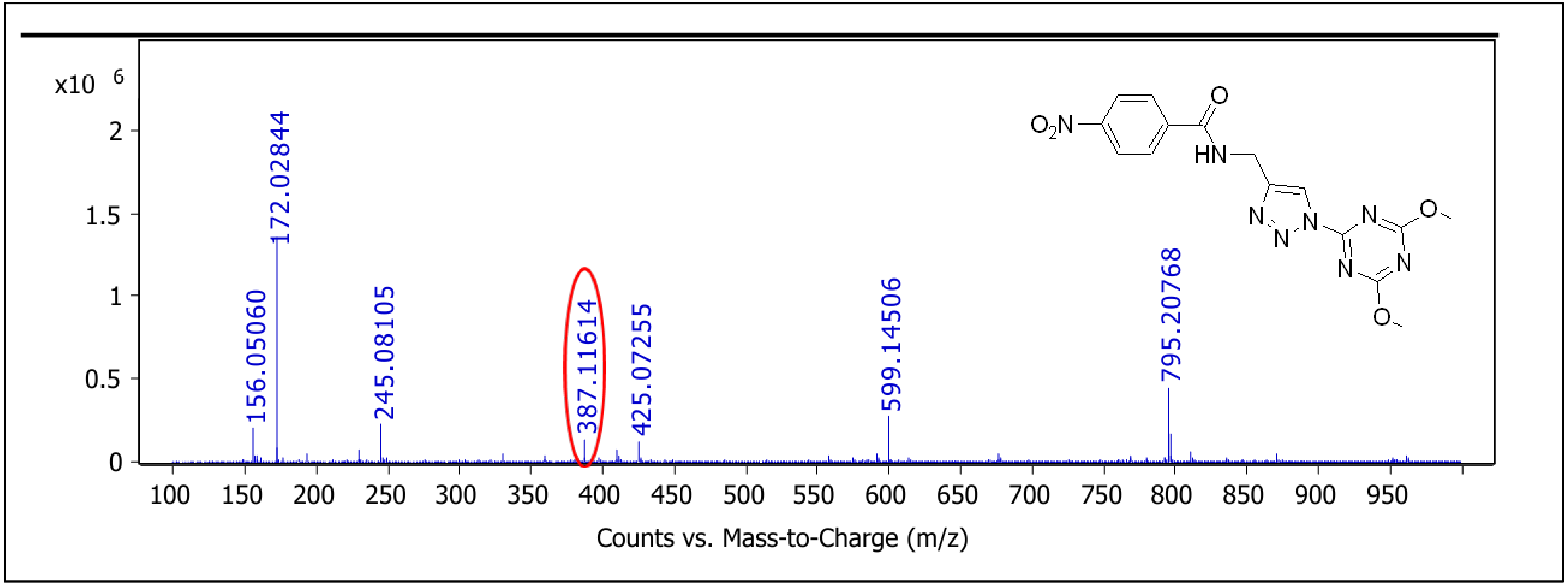

Figure S47: HRMS of *N*-((1-(4,6-dimethoxy-1,3,5-triazin-2-yl)-1*H*-1,2,3-triazol-4-yl) methyl)-4-nitrobenzamide **(3f)**

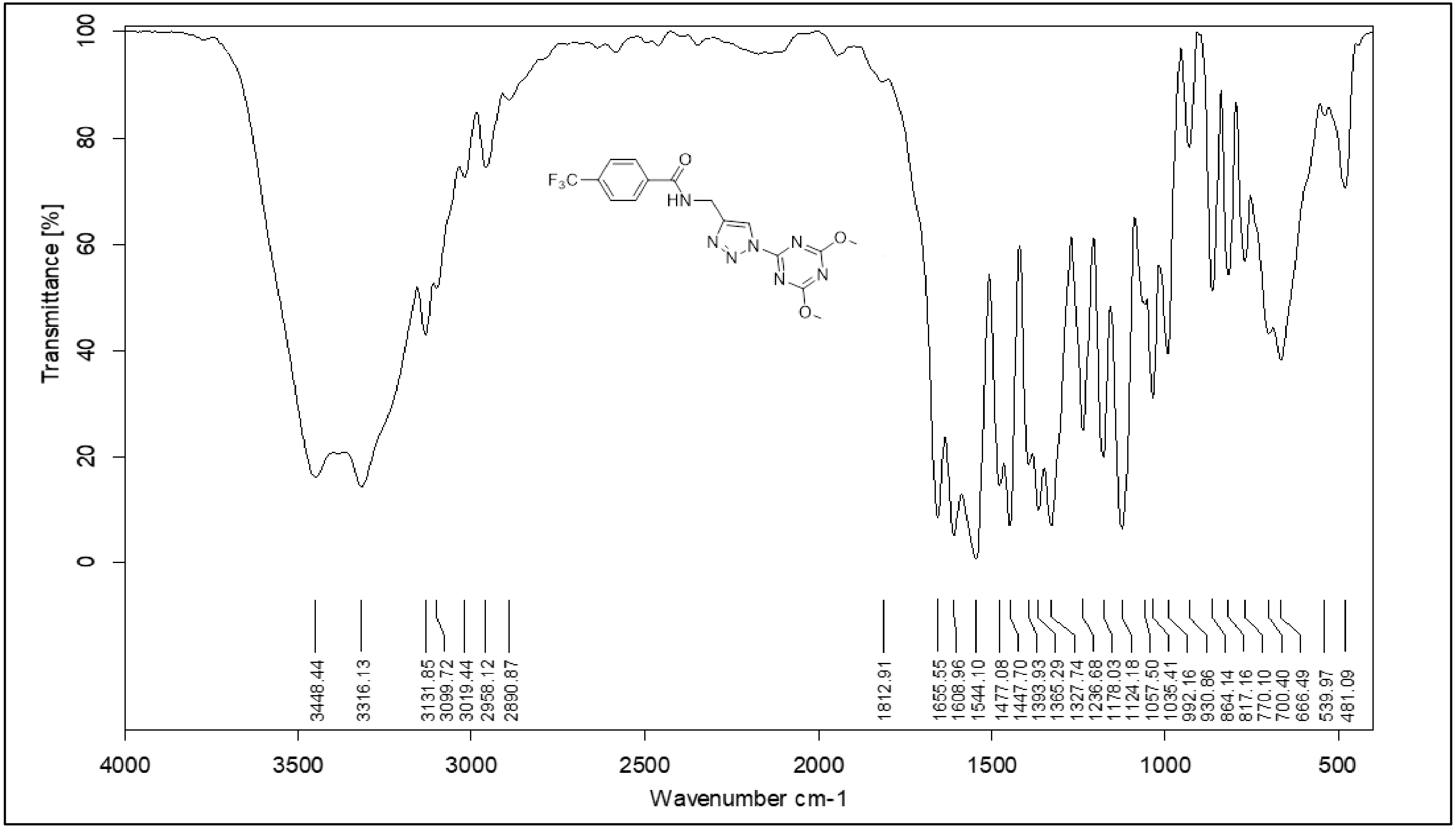

Figure S48: IR (KBr) spectrum of *N*-((1-(4,6-dimethoxy-1,3,5-triazin-2-yl)-1*H*-1,2,3-triazol-4-yl) methyl)-4-(trifluoromethyl) benzamide **(3g)**

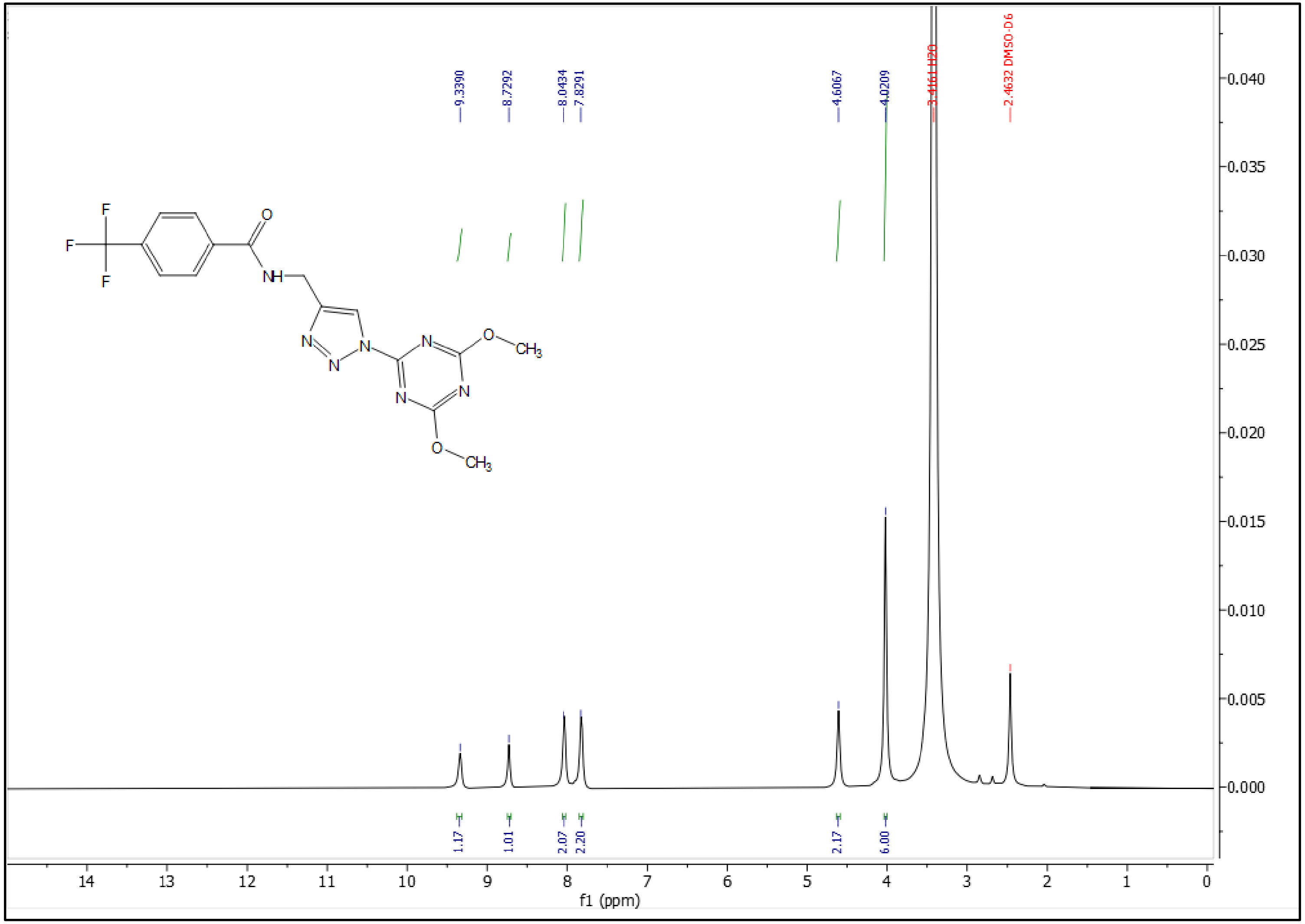

Figure S49: ^{1}H-NMR (500 MHz, DMSO-d_6) spectrum of *N*-((1-(4,6-dimethoxy-1,3,5-triazin-2-yl)-1*H*-1,2,3-triazol-4-yl) methyl)-4-(trifluoromethyl) benzamide **(3g)**

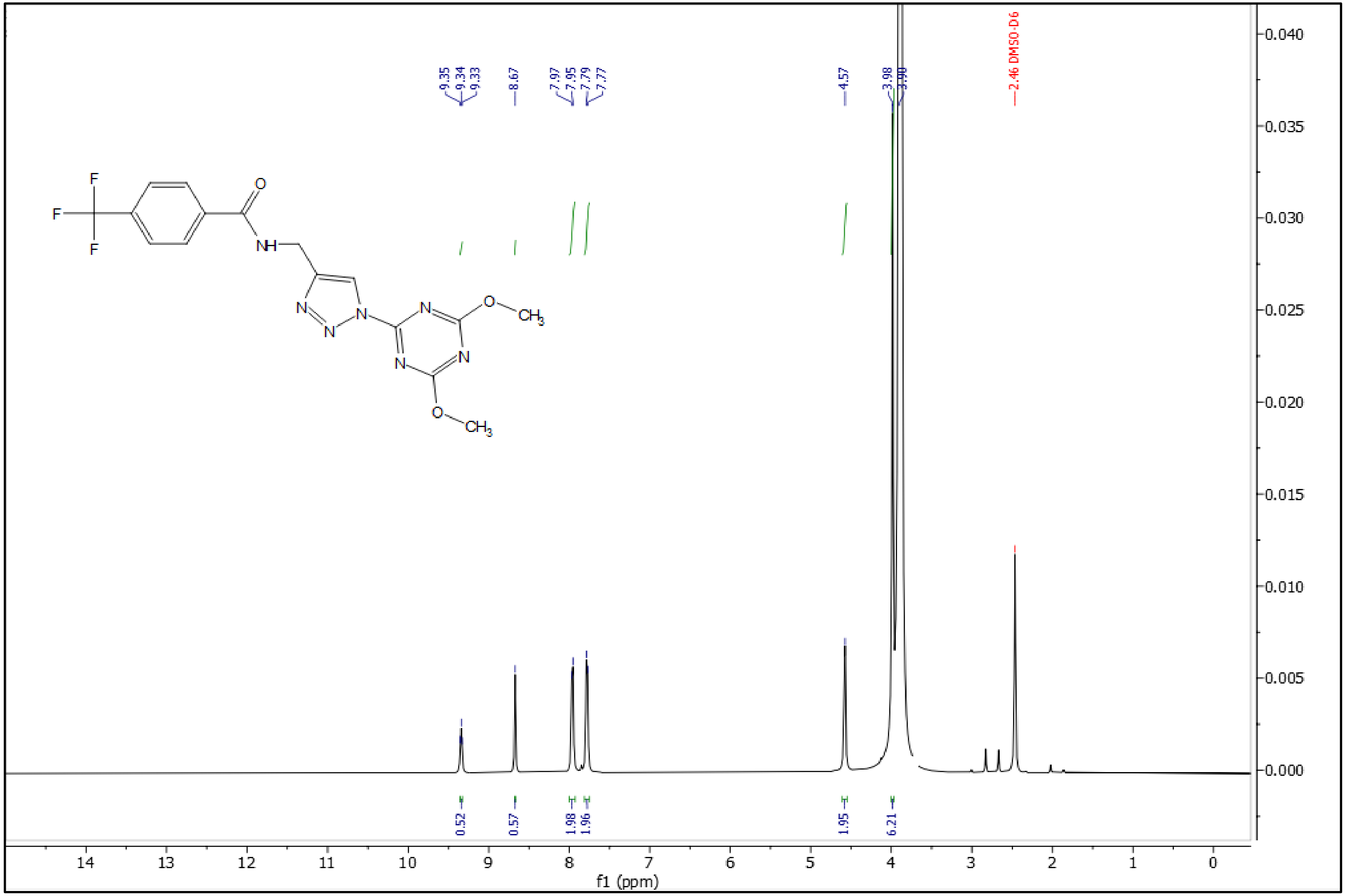

Figure S50: ^{1}H-NMR (500 MHz, DMSO-d_6, D_2O) spectrum of *N*-((1-(4,6-dimethoxy-1,3,5-triazin-2-yl)-1*H*-1,2,3-triazol-4-yl) methyl)-4-(trifluoromethyl) benzamide **(3g)**

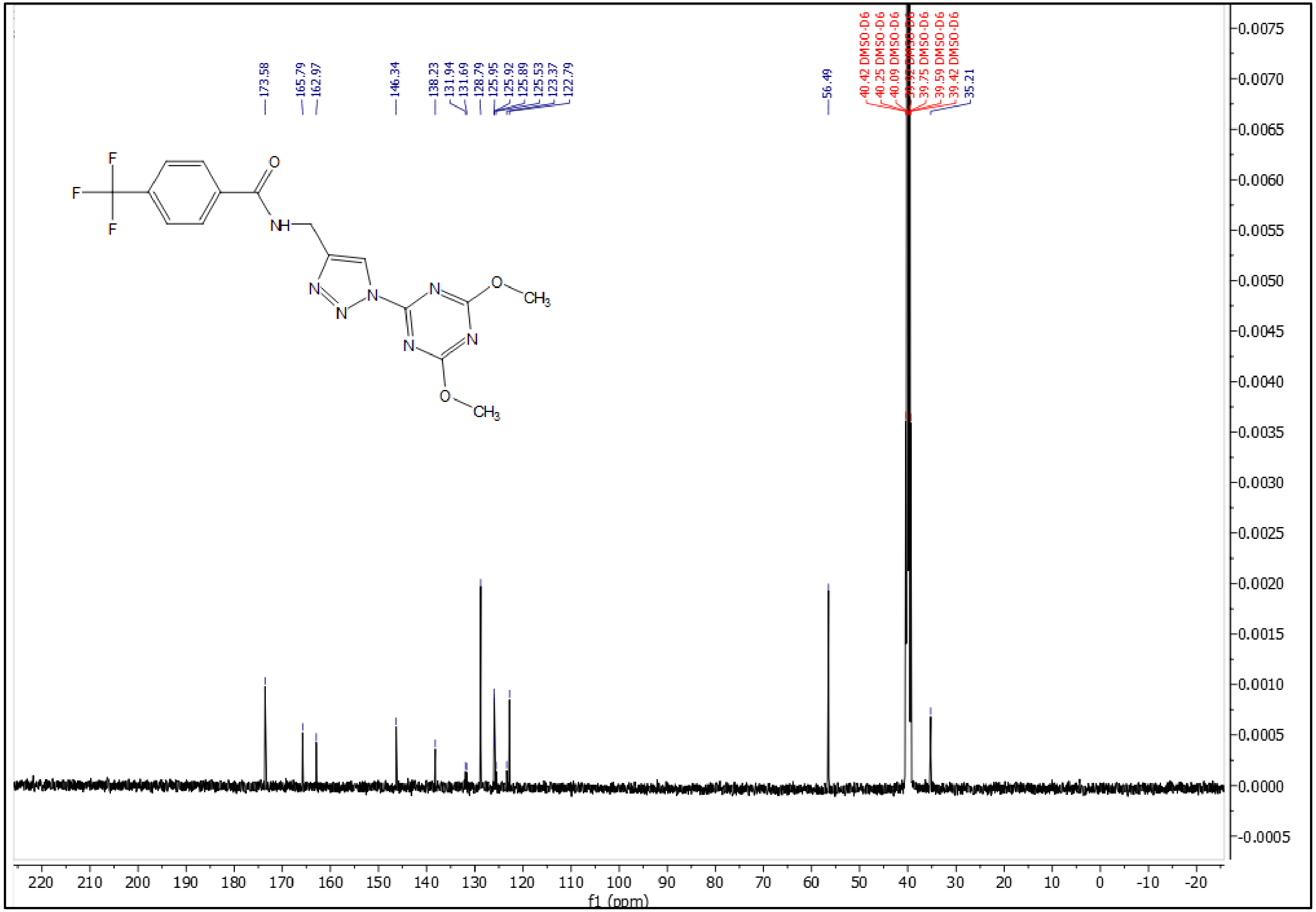

Figure S51: ^{13}C-NMR (DMSO-d_6) spectrum of *N*-((1-(4,6-dimethoxy-1,3,5-triazin-2-yl)-1*H*-1,2,3-triazol-4-yl) methyl)-4-(trifluoromethyl) benzamide **(3g)**

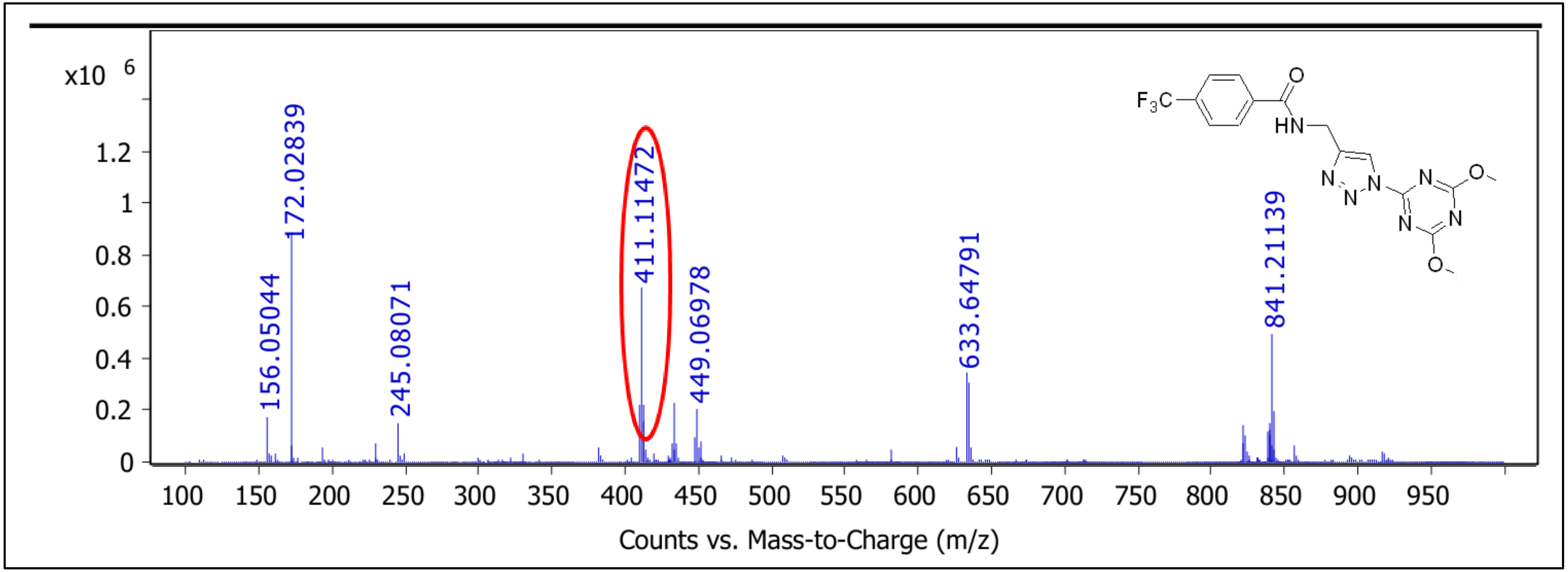

Figure S52: HRMS of *N*-((1-(4,6-dimethoxy-1,3,5-triazin-2-yl)-1*H*-1,2,3-triazol-4-yl) methyl)-4-(trifluoromethyl) benzamide **(3g)**

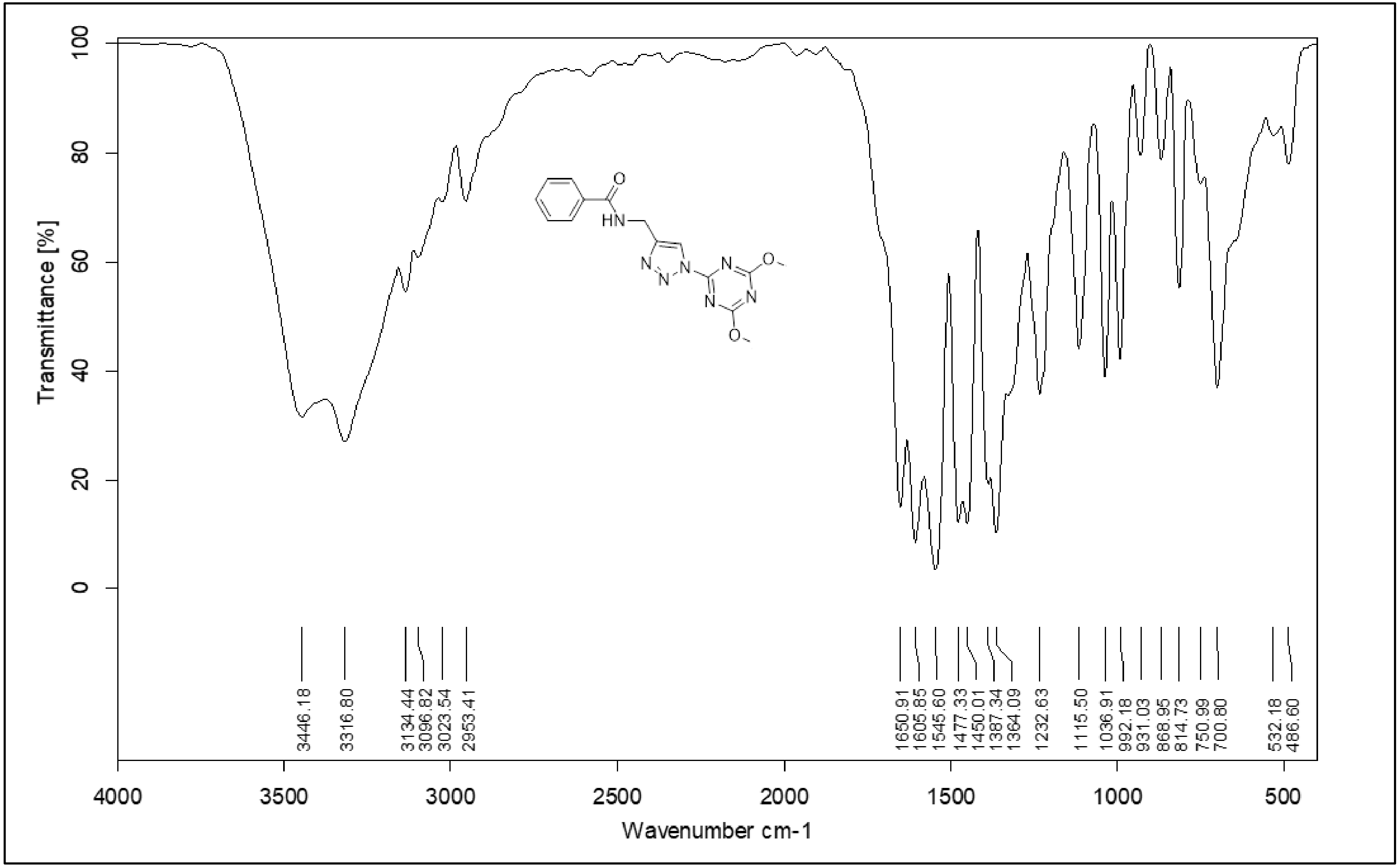

Figure S53: IR (KBr) spectrum of *N*-((1-(4,6-dimethoxy-1,3,5-triazin-2-yl)-1*H*-1,2,3-triazol-4-yl) methyl) benzamide **(3h)**

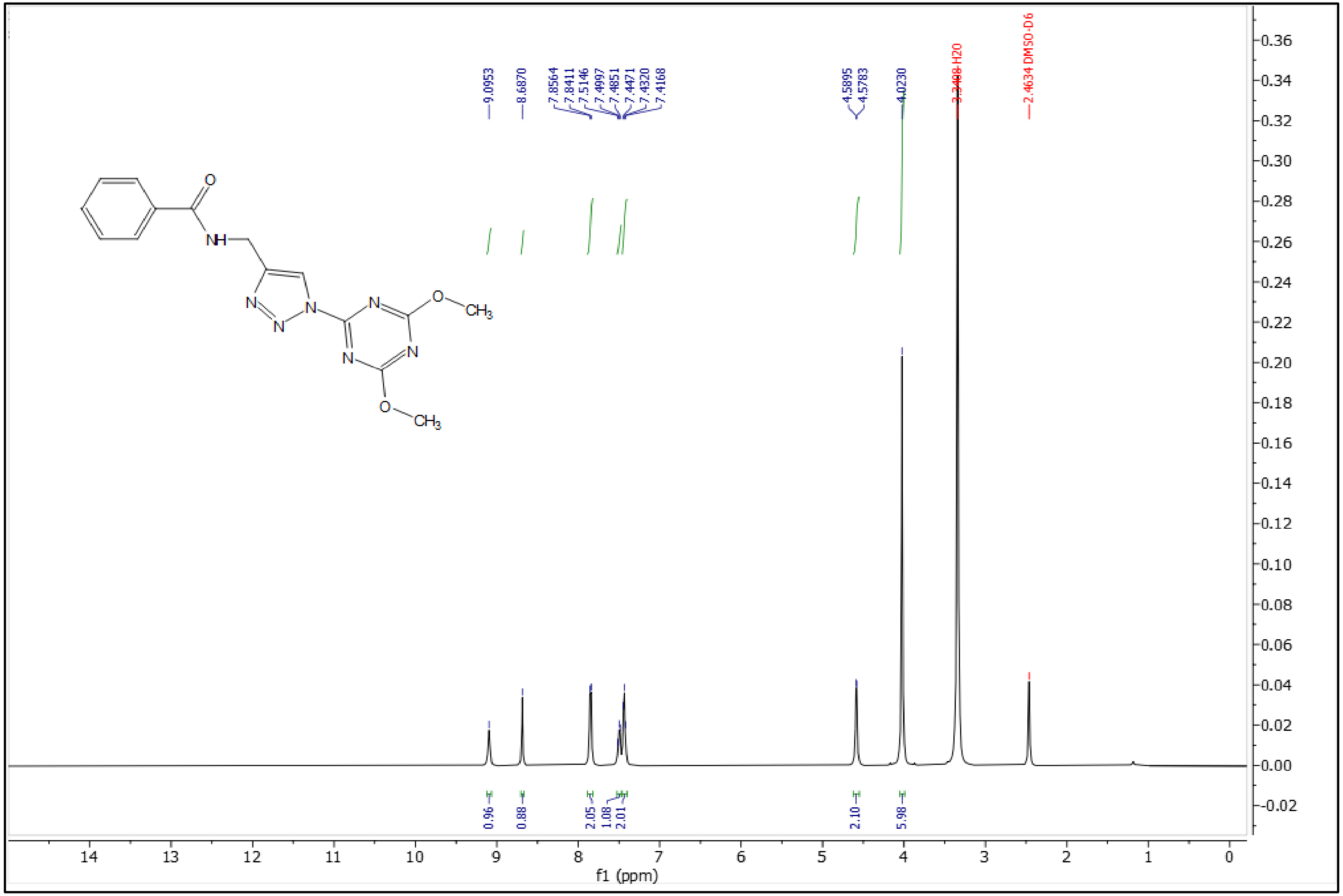

Figure S54: ^{1}H-NMR (500 MHz, DMSO-d_6) spectrum of *N*-((1-(4,6-dimethoxy-1,3,5-triazin-2-yl)-1*H*-1,2,3-triazol-4-yl) methyl) benzamide **(3h)**

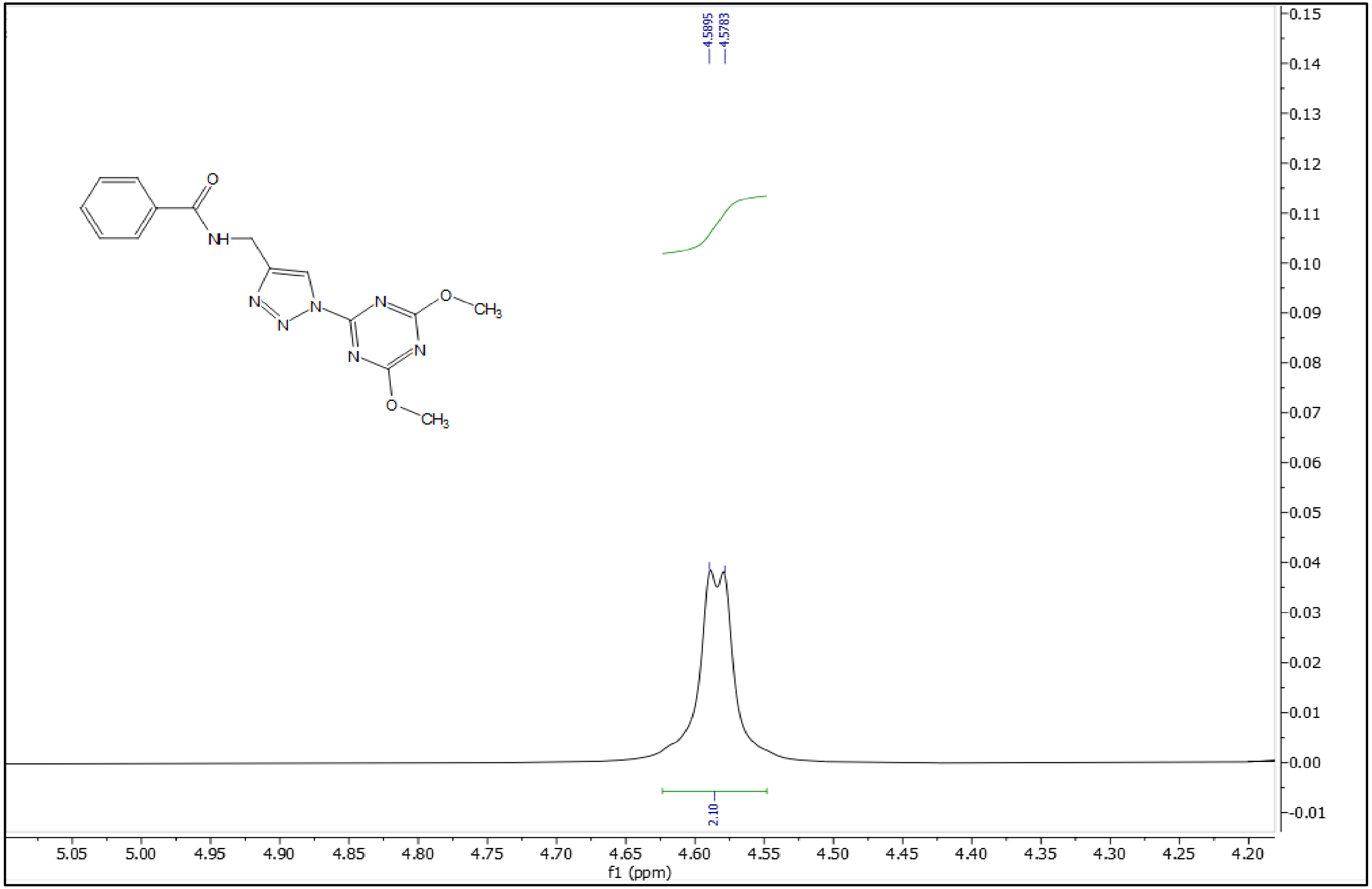

Figure S55: ^{1}H-NMR (500 MHz, DMSO-d_6) spectrum of *N*-((1-(4,6-dimethoxy-1,3,5-triazin-2-yl)-1*H*-1,2,3-triazol-4-yl) methyl) benzamide **(3h)**

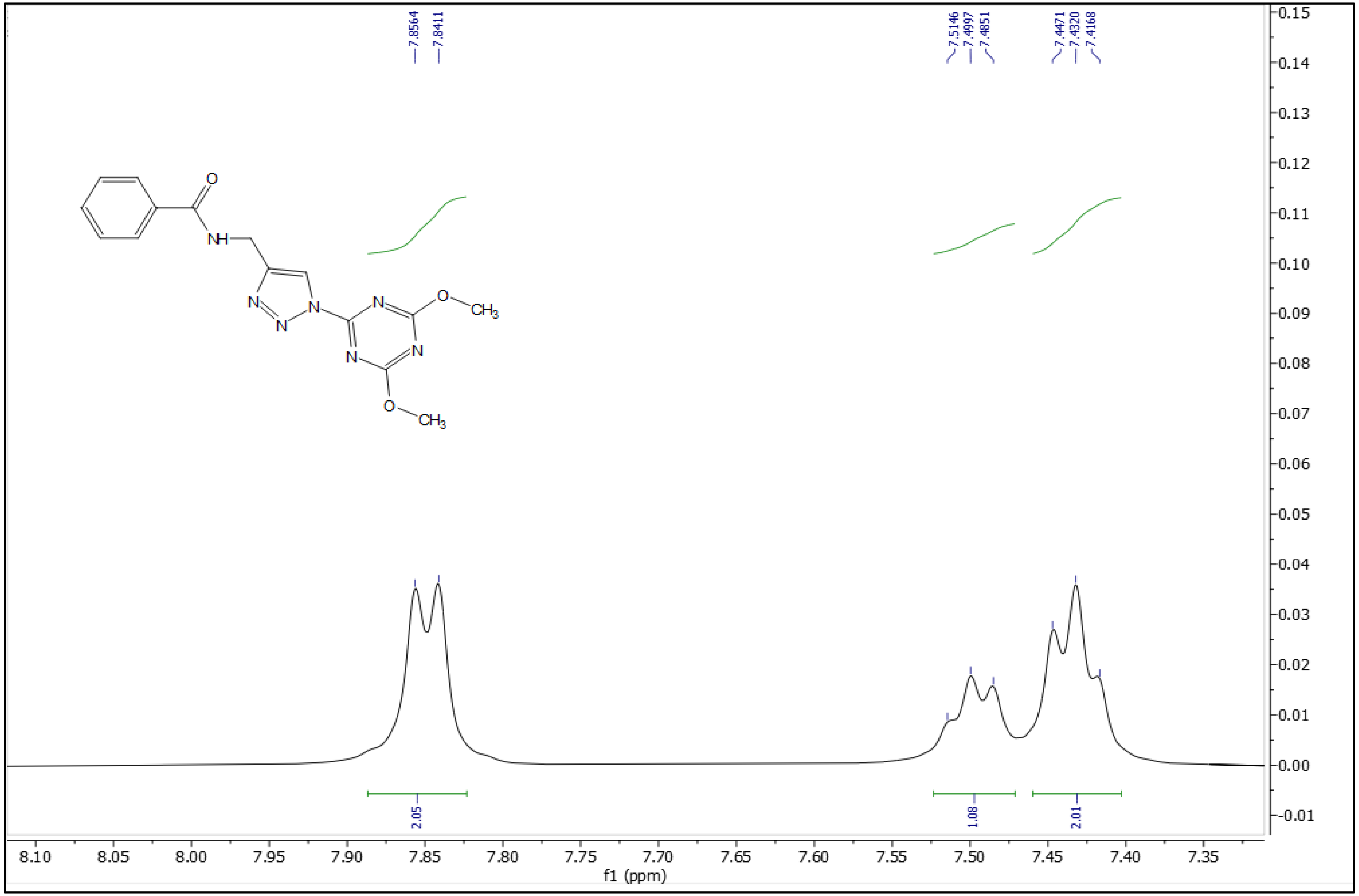

Figure S56: ^{1}H-NMR (500 MHz, DMSO-d_6) spectrum of *N*-((1-(4,6-dimethoxy-1,3,5-triazin-2-yl)-1*H*-1,2,3-triazol-4-yl) methyl) benzamide **(3h)**

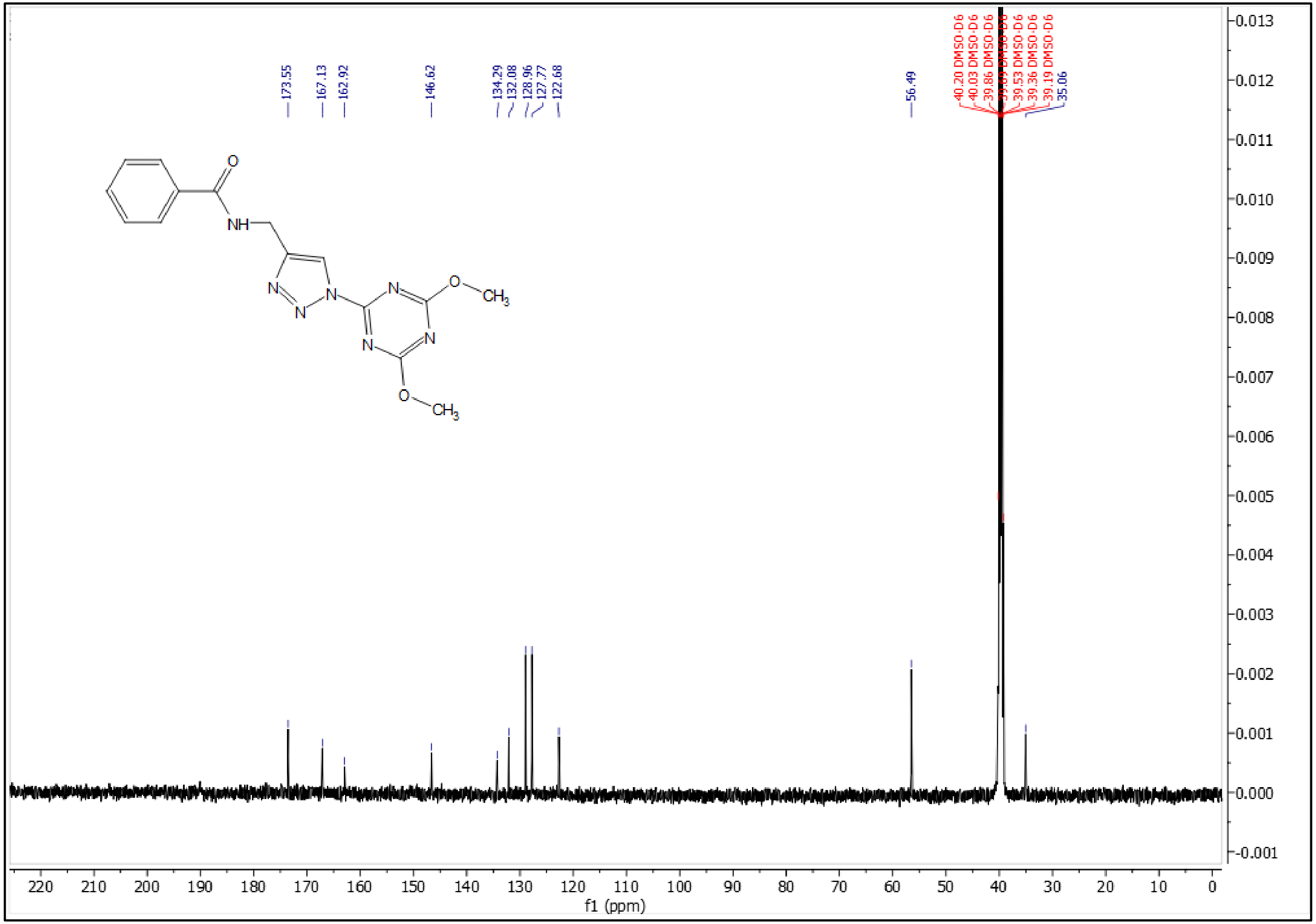

Figure S57: ^{13}C-NMR (DMSO-d_6) spectrum of *N*-((1-(4,6-dimethoxy-1,3,5-triazin-2-yl)-1*H*-1,2,3-triazol-4-yl) methyl) benzamide **(3h)**

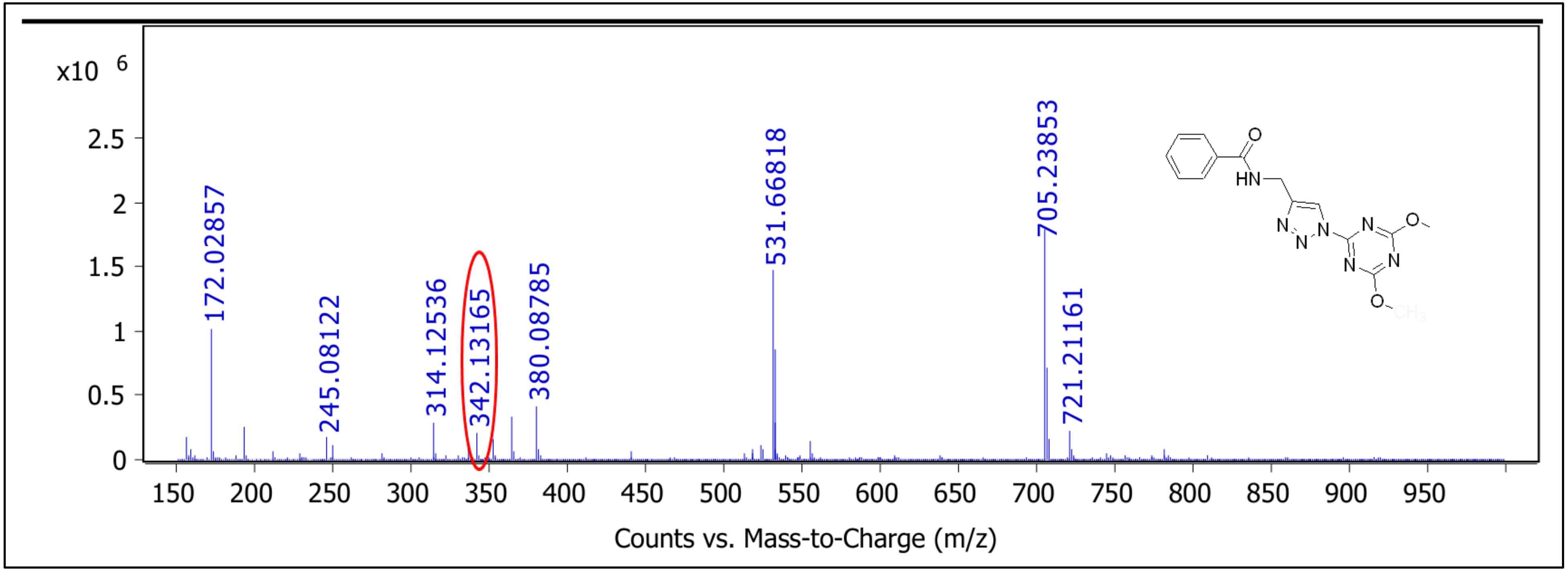

Figure S58: HRMS of *N*-((1-(4,6-dimethoxy-1,3,5-triazin-2-yl)-1*H*-1,2,3-triazol-4-yl) methyl) benzamide **(3h)**

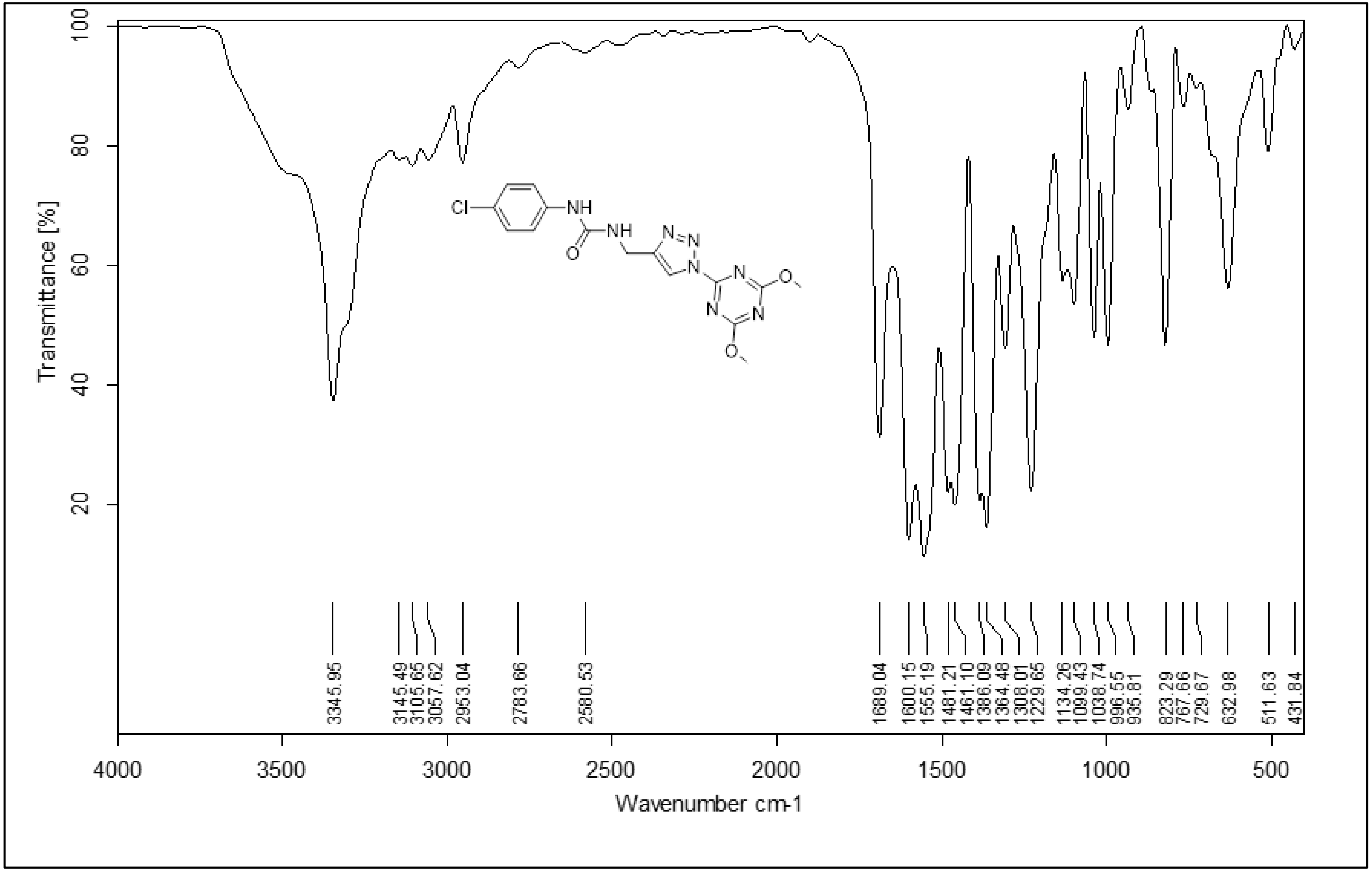

Figure S59: IR (KBr) spectrum of 1-(4-chlorophenyl)-3-((1-(4,6-dimethoxy-1,3,5-triazin-2-yl)-1*H*-1,2,3-triazol-4-yl) methyl) urea **(6a)**

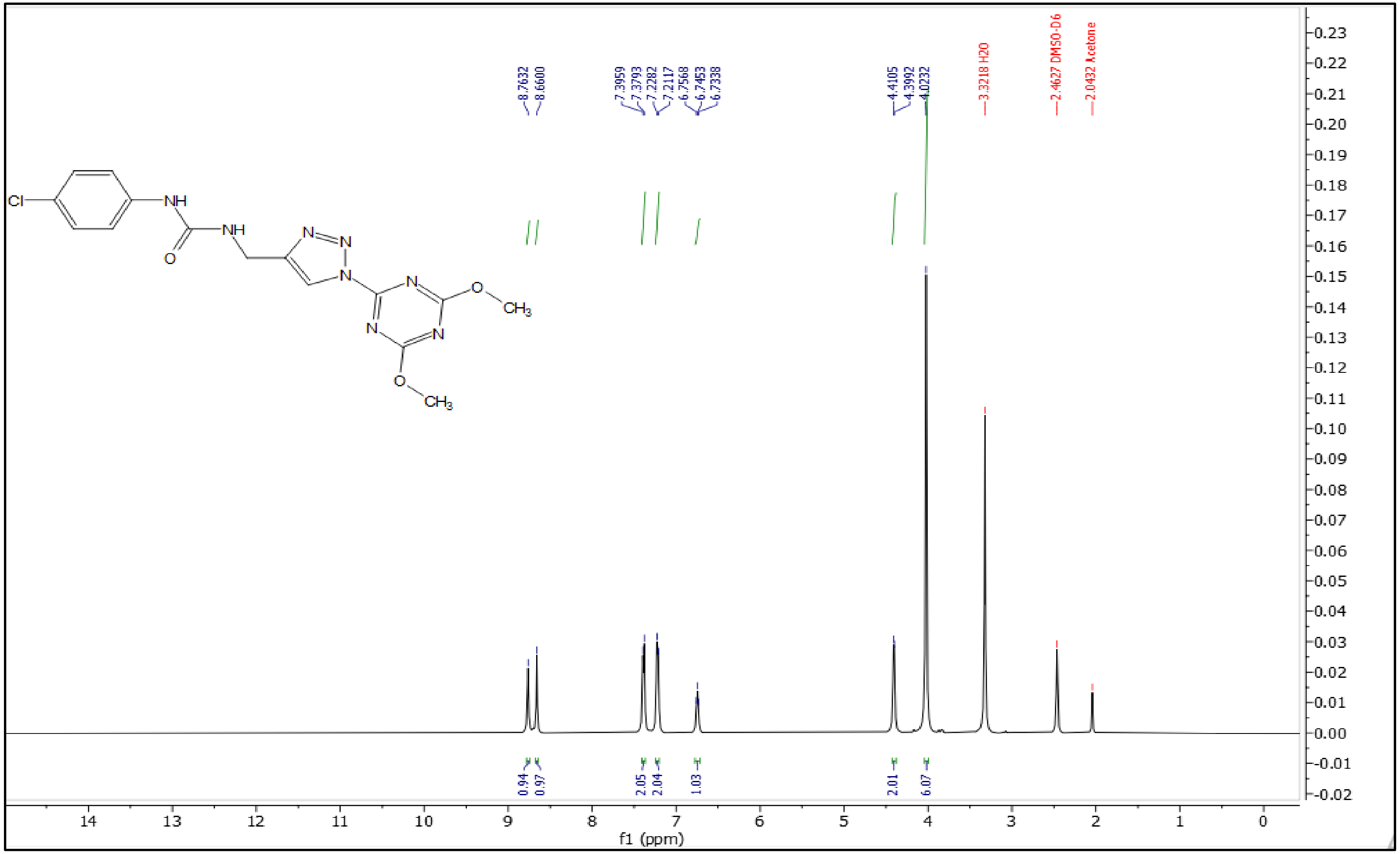

Figure S60: ^{1}H-NMR (500 MHz, DMSO-d_6) spectrum of 1-(4-chlorophenyl)-3-((1-(4,6-dimethoxy-1,3,5-triazin-2-yl)-1*H*-1,2,3-triazol-4-yl) methyl) urea **(6a)**

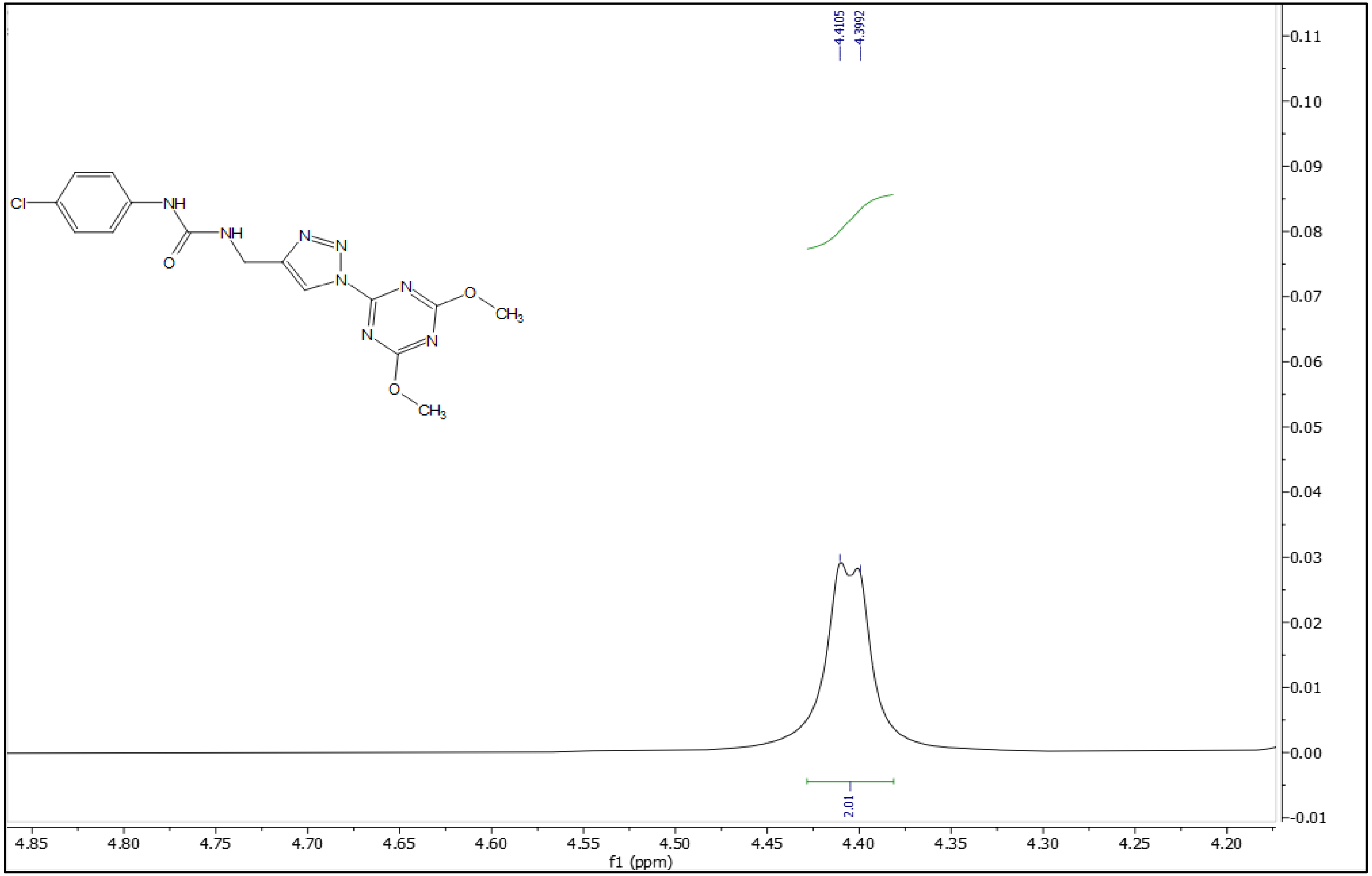

Figure S61: ^{1}H-NMR (500 MHz, DMSO-d_6) spectrum of 1-(4-chlorophenyl)-3-((1-(4,6-dimethoxy-1,3,5-triazin-2-yl)-1*H*-1,2,3-triazol-4-yl) methyl) urea **(6a)**

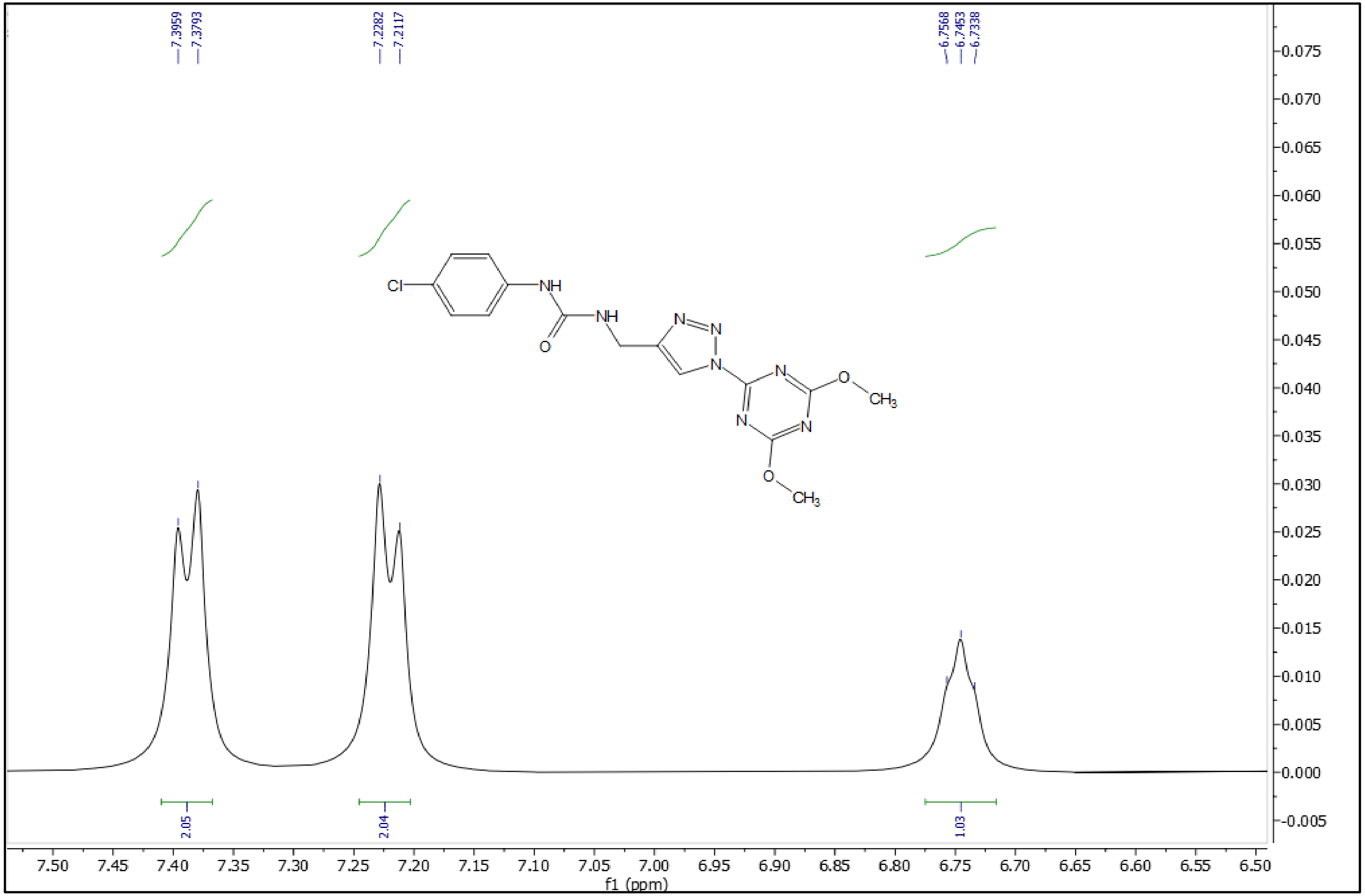

Figure S62: ^{1}H-NMR (500 MHz, DMSO-d_6) spectrum of 1-(4-chlorophenyl)-3-((1-(4,6-dimethoxy-1,3,5-triazin-2-yl)-1*H*-1,2,3-triazol-4-yl) methyl) urea **(6a)**

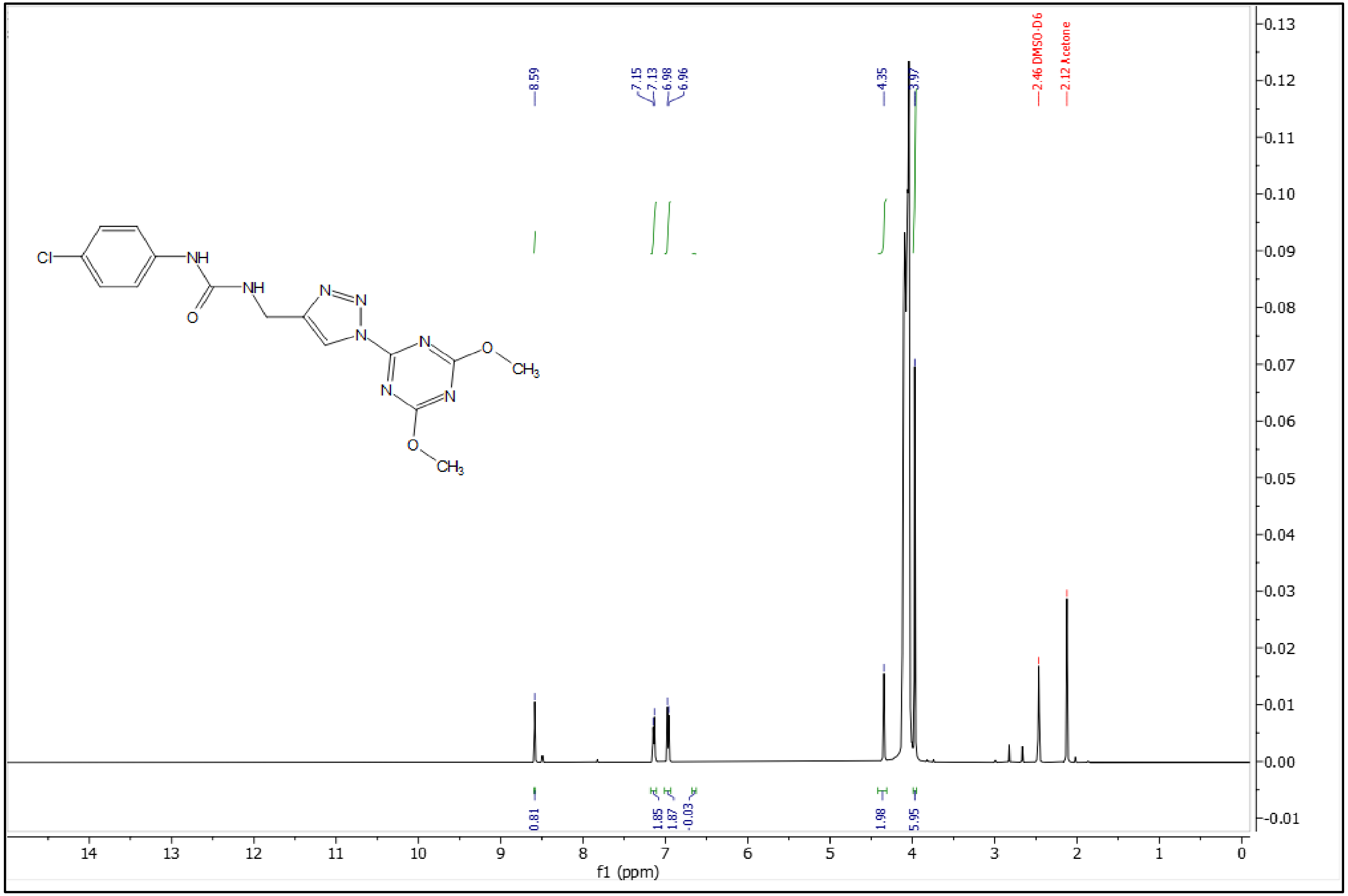

Figure S63: ^{1}H-NMR (500 MHz, DMSO-*d*$_6$, D_2O) spectrum of 1-(4-chlorophenyl)-3-((1-(4,6-dimethoxy-1,3,5-triazin-2-yl)-1*H*-1,2,3-triazol-4-yl) methyl) urea **(6a)**

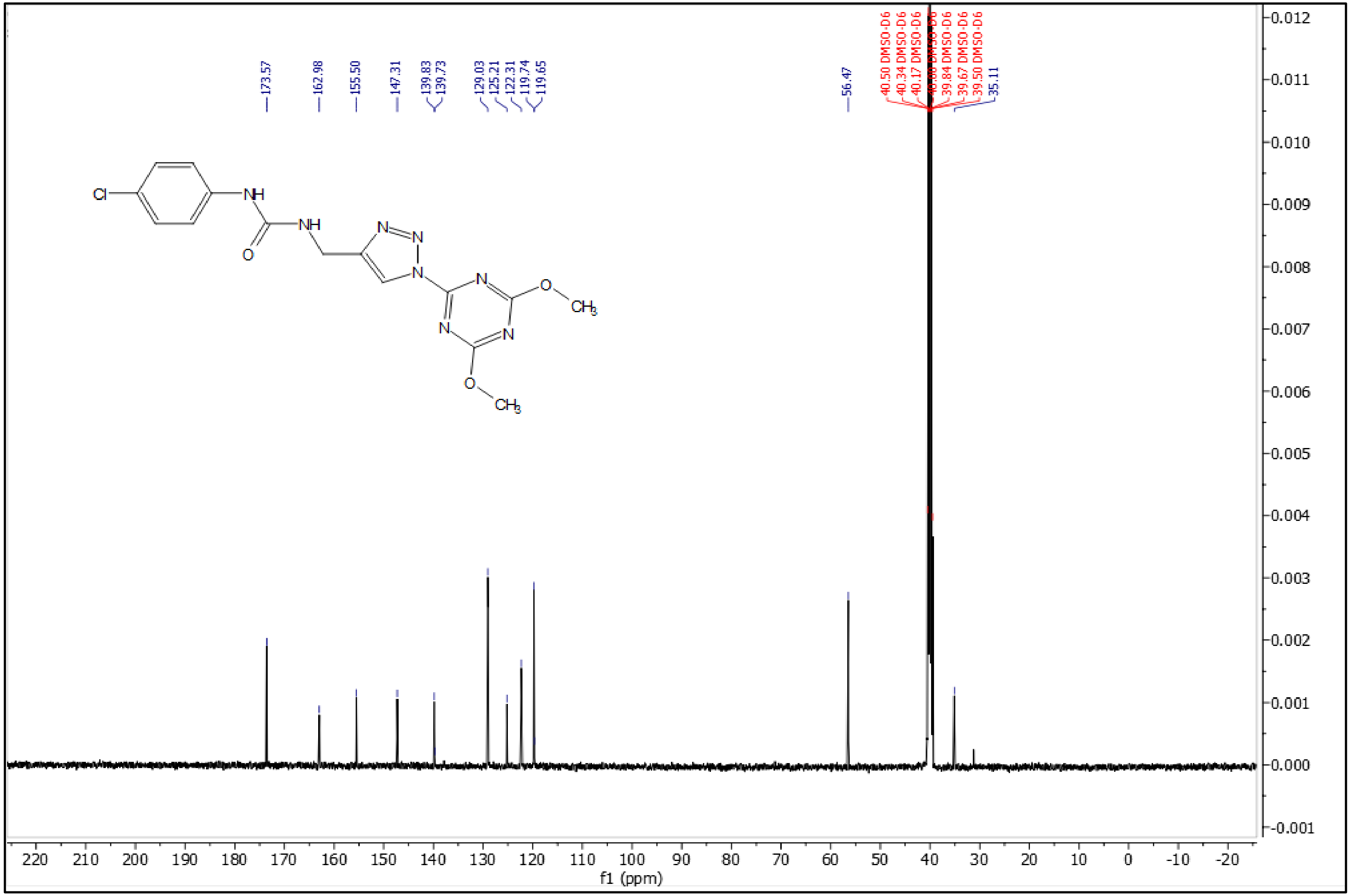

Figure S64: ^{13}C-NMR (DMSO-d_6) spectrum of 1-(4-chlorophenyl)-3-((1-(4,6-dimethoxy-1,3,5-triazin-2-yl)-1*H*-1,2,3-triazol-4-yl) methyl) urea **(6a)**

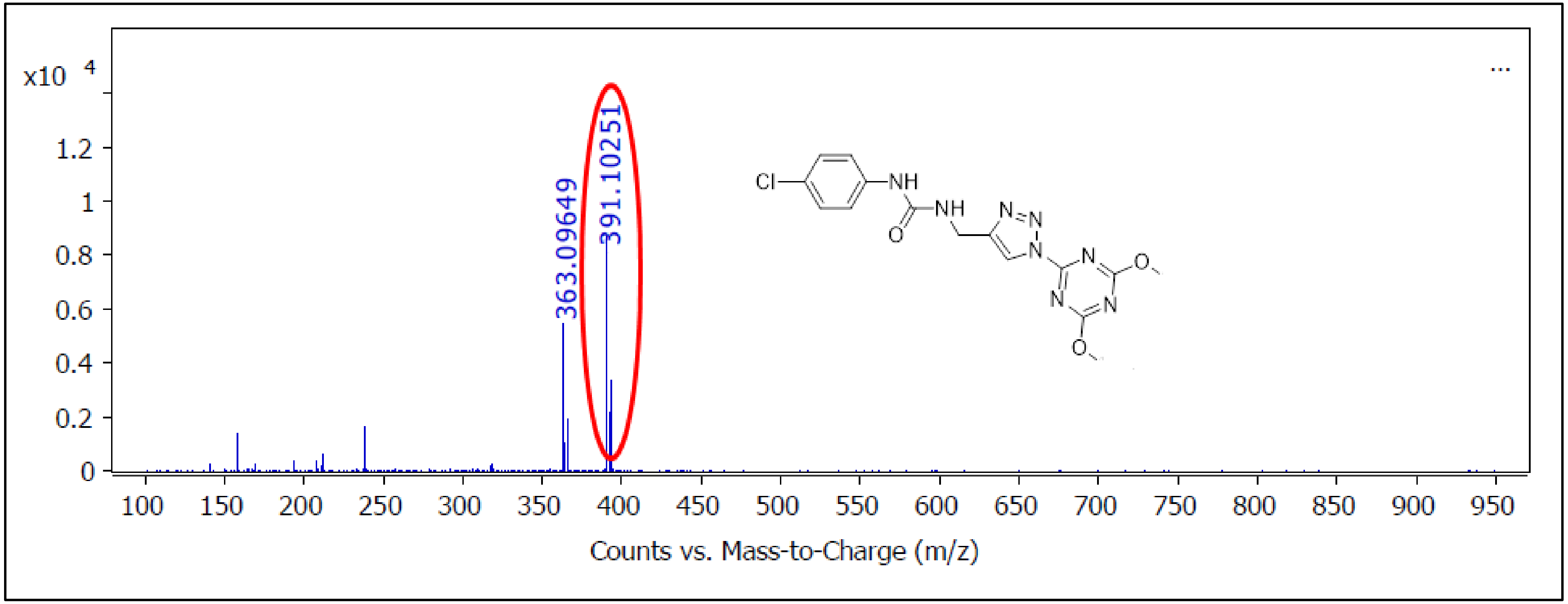

Figure S65: HRMS of 1-(4-chlorophenyl)-3-((1-(4,6-dimethoxy-1,3,5-triazin-2-yl)-1*H*-1,2,3-triazol-4-yl) methyl) urea **(6a)**

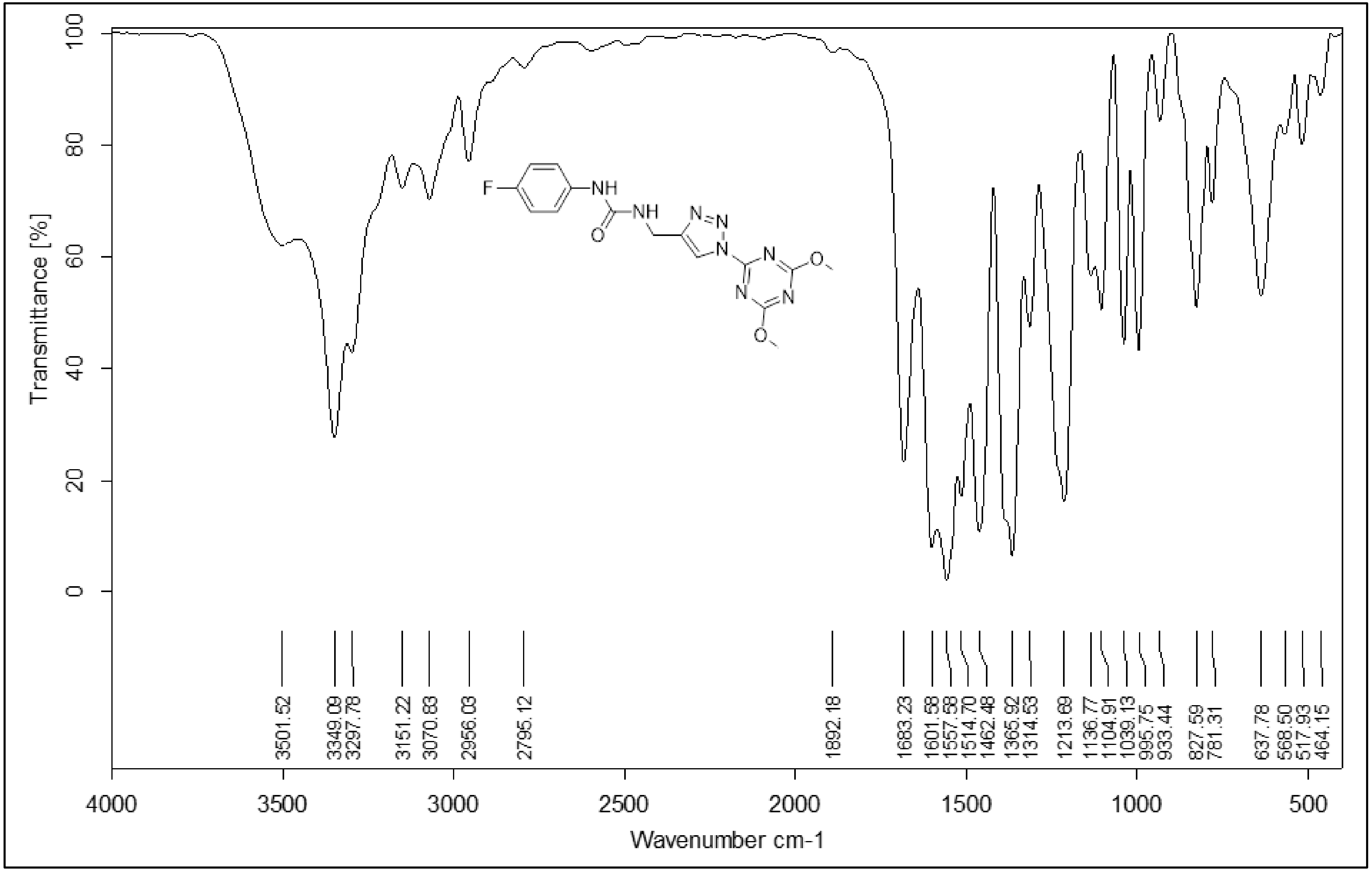

Figure S66: IR (KBr) spectrum of 1-((1-(4,6-dimethoxy-1,3,5-triazin-2-yl)-1*H*-1,2,3-triazol-4-yl) methyl)-3-(4-fluorophenyl) urea **(6b)**

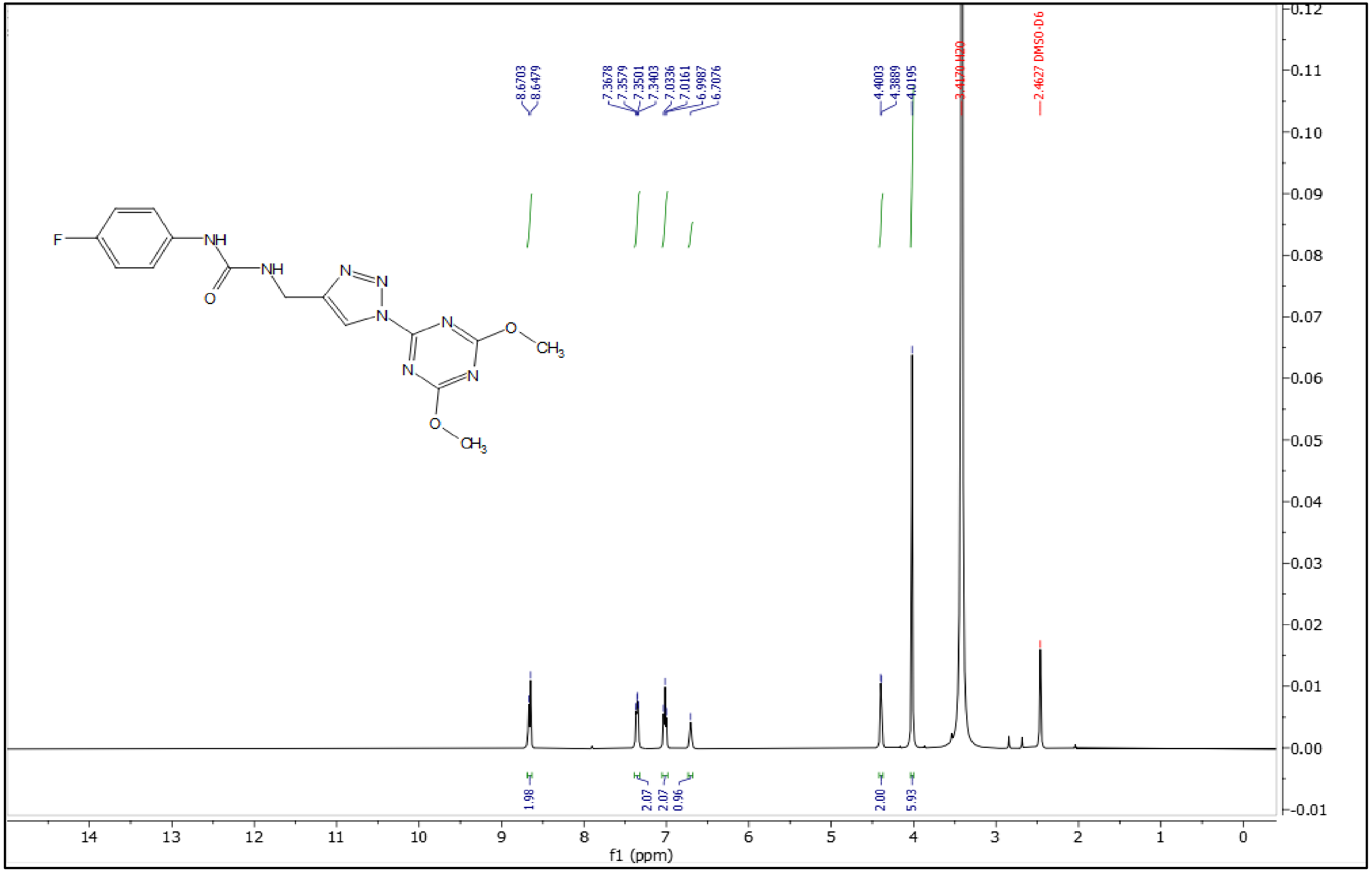

Figure S67: ^{1}H-NMR (500 MHz, DMSO-d_6) spectrum of 1-((1-(4,6-dimethoxy-1,3,5-triazin-2-yl)-1*H*-1,2,3-triazol-4-yl) methyl)-3-(4-fluorophenyl) urea **(6b)**

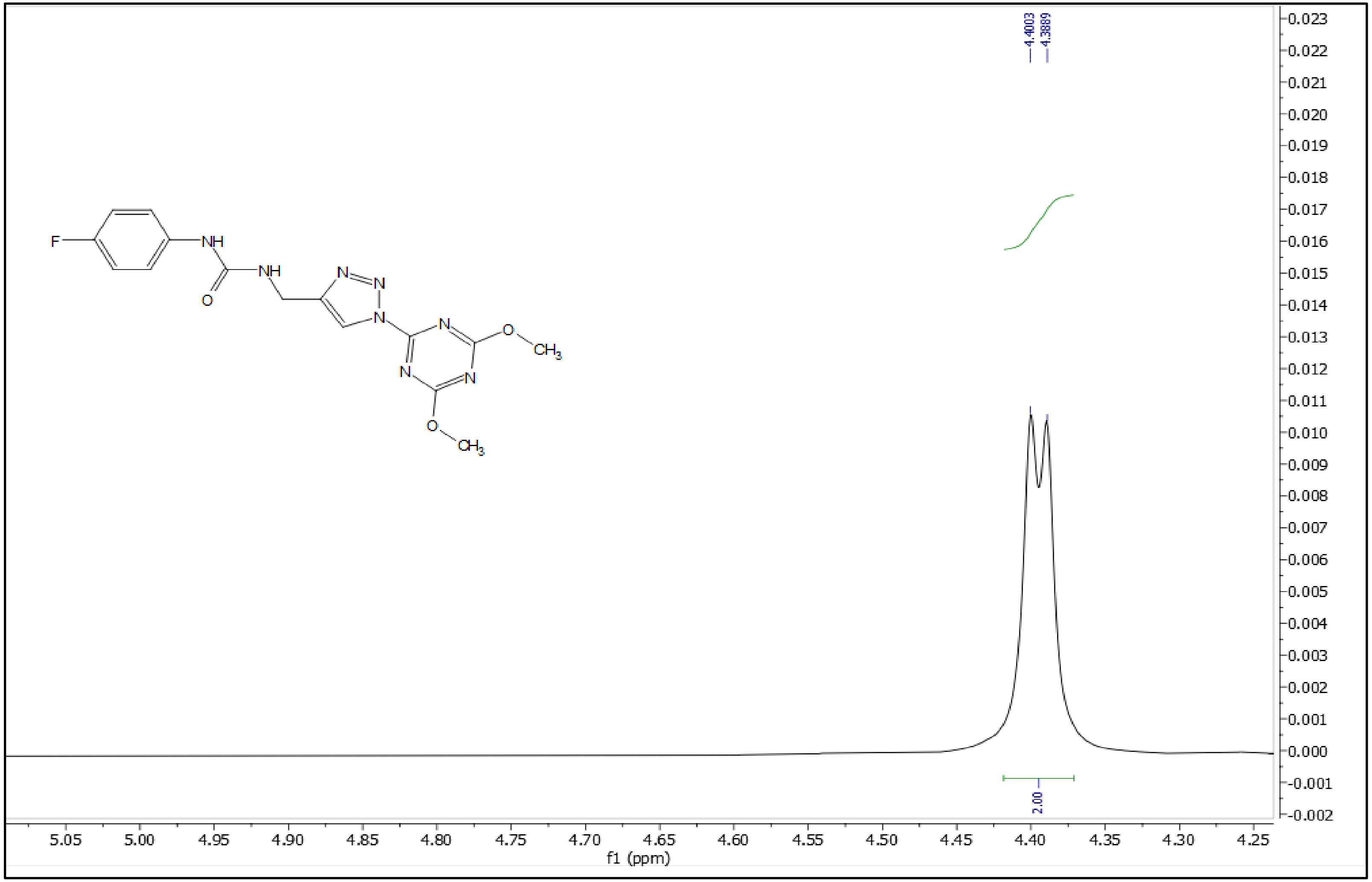

Figure S68: ^{1}H-NMR (500 MHz, DMSO-d_6) spectrum of 1-((1-(4,6-dimethoxy-1,3,5-triazin-2-yl)-1*H*-1,2,3-triazol-4-yl) methyl)-3-(4-fluorophenyl) urea **(6b)**

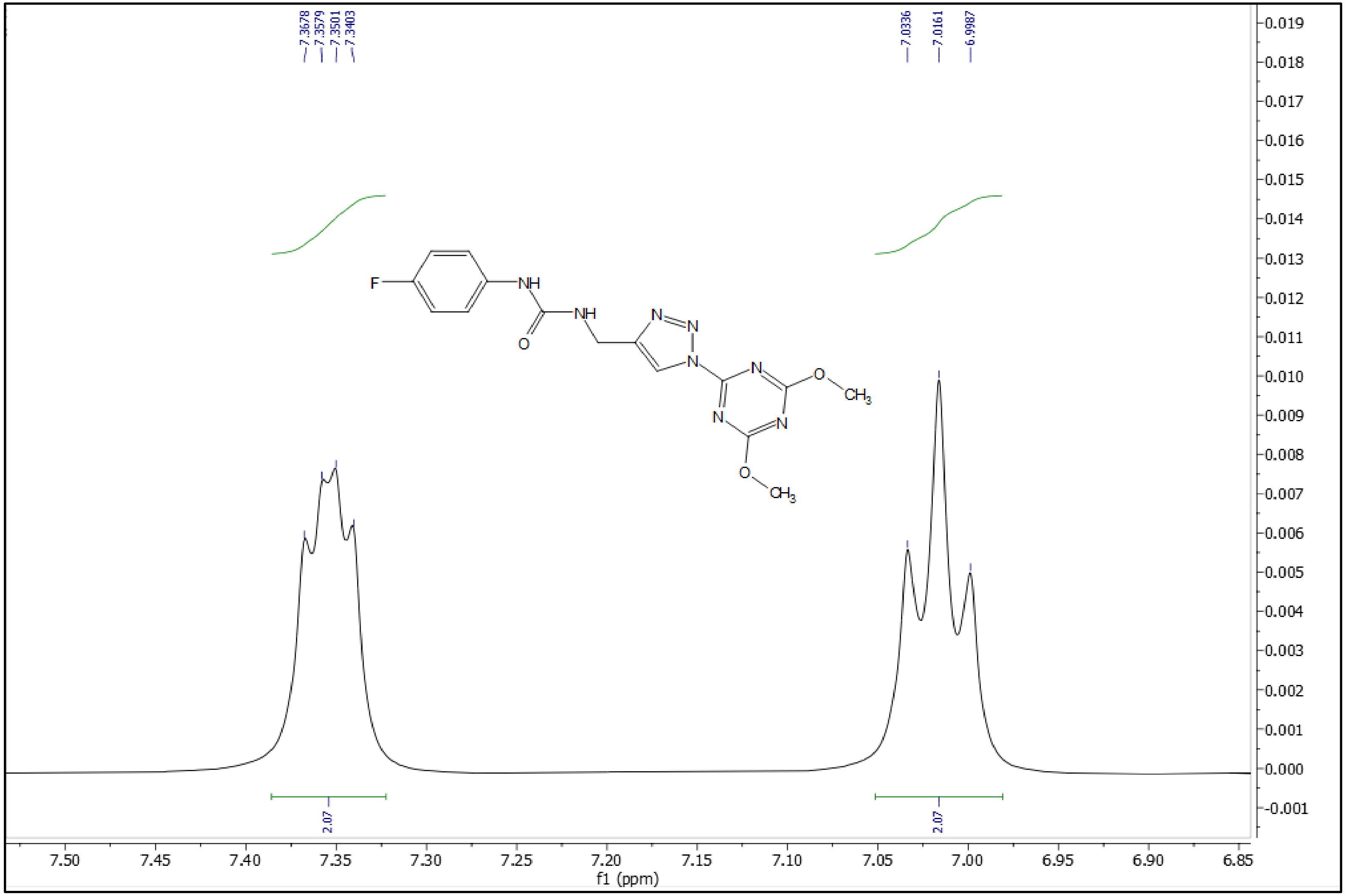

Figure S69: ^{1}H-NMR (500 MHz, DMSO-d_6) spectrum of 1-((1-(4,6-dimethoxy-1,3,5-triazin-2-yl)-1*H*-1,2,3-triazol-4-yl) methyl)-3-(4-fluorophenyl) urea **(6b)**

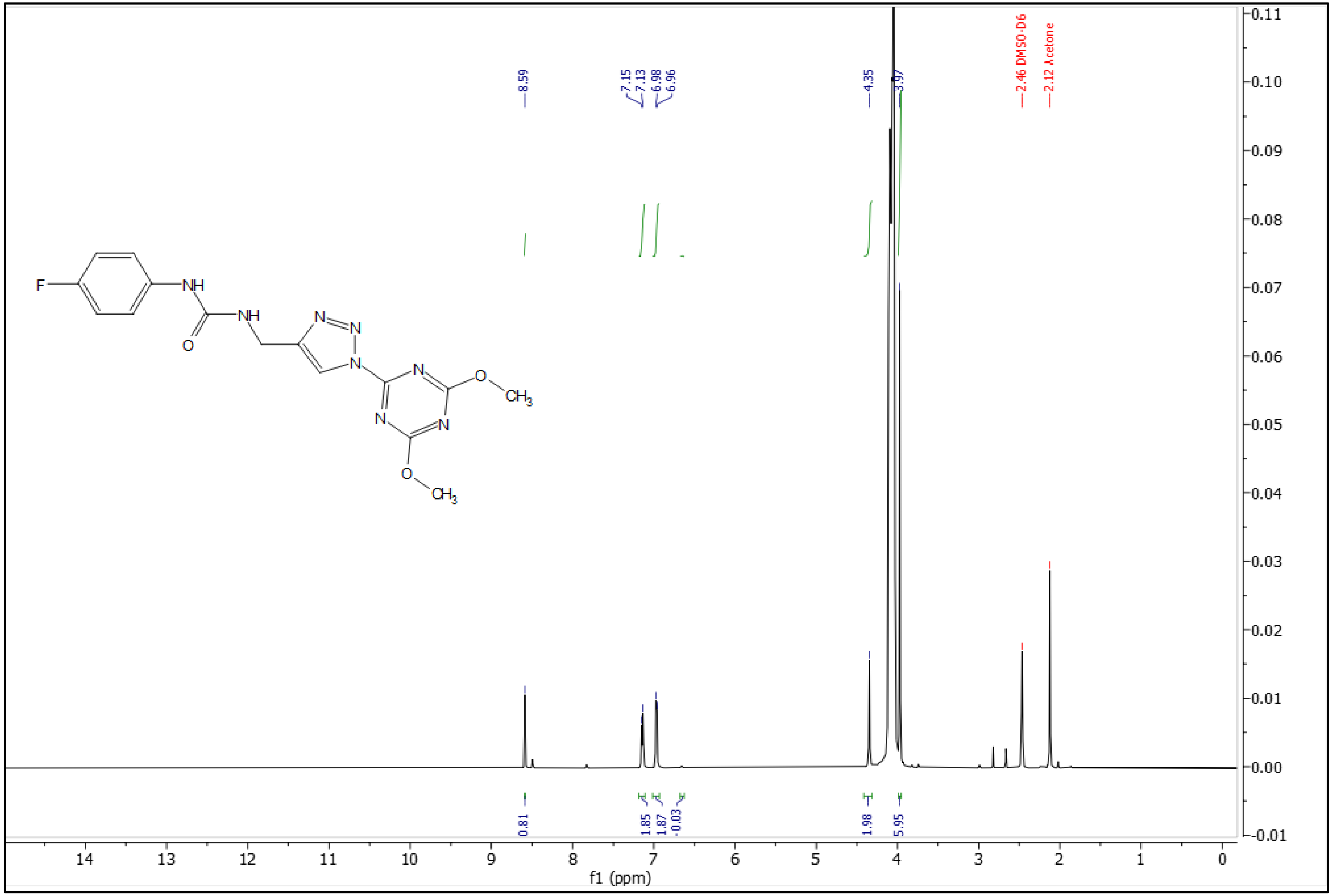

Figure S70: ^{1}H-NMR (500 MHz, DMSO-d_6, D_2O) spectrum of 1-((1-(4,6-dimethoxy-1,3,5-triazin-2-yl)-1*H*-1,2,3-triazol-4-yl) methyl)-3-(4-fluoro-phenyl) urea **(6b)**

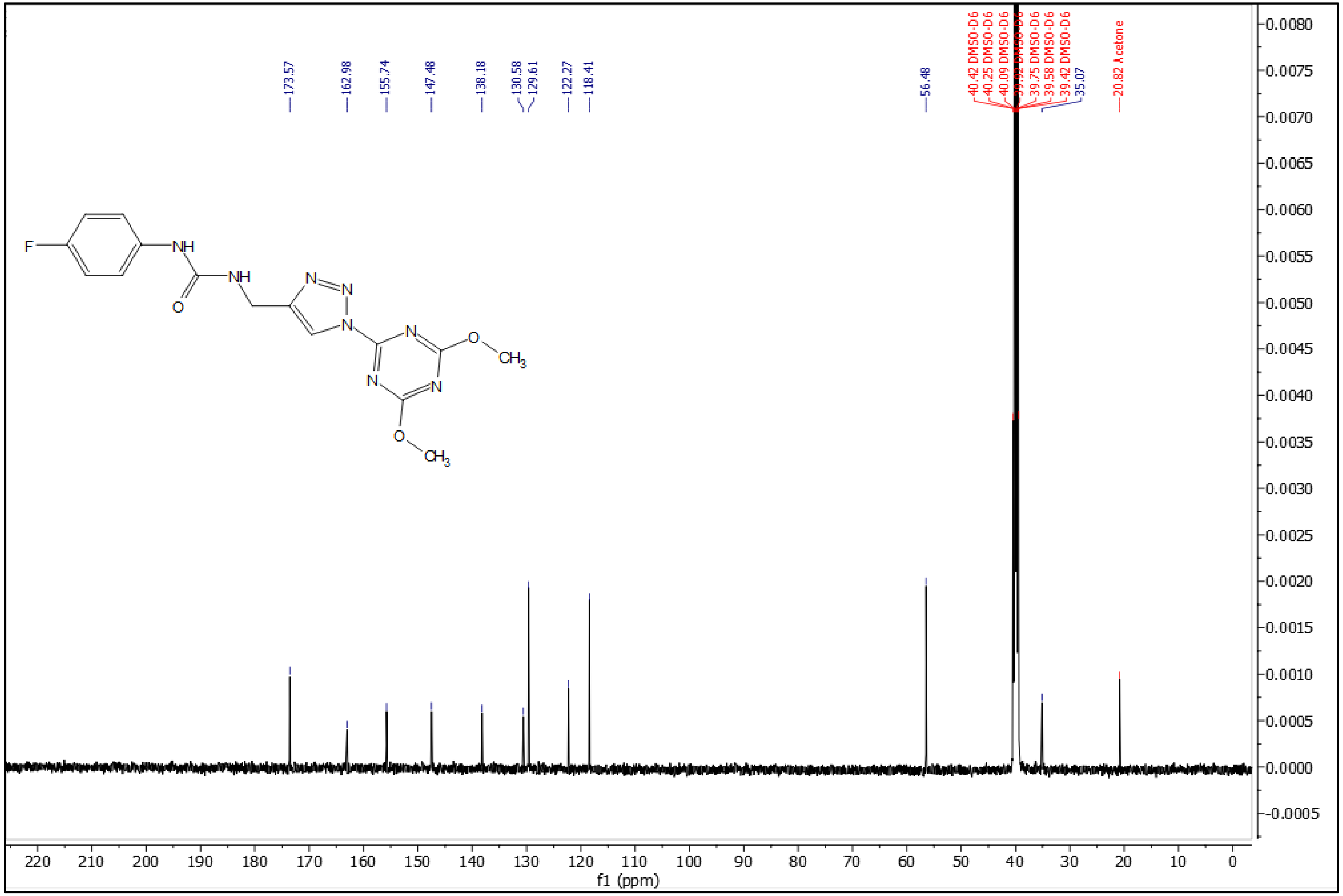

Figure S71: ^{13}C-NMR (DMSO-d_6) spectrum of 1-((1-(4,6-dimethoxy-1,3,5-triazin-2-yl)-1*H*-1,2,3-triazol-4-yl) methyl)-3-(4-fluorophenyl) urea **(6b)**

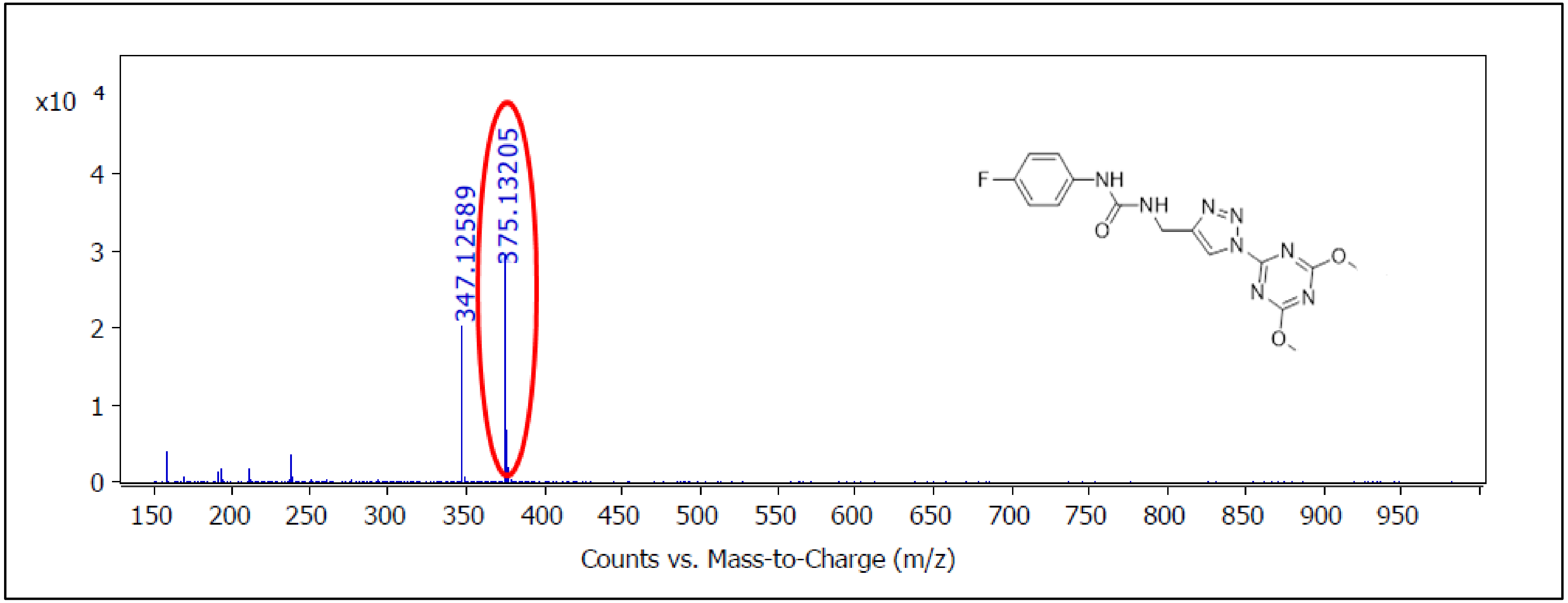

Figure S72: HRMS of 1-((1-(4,6-dimethoxy-1,3,5-triazin-2-yl)-1*H*-1,2,3-triazol-4-yl) methyl)-3-(4-fluorophenyl) urea **(6b)**

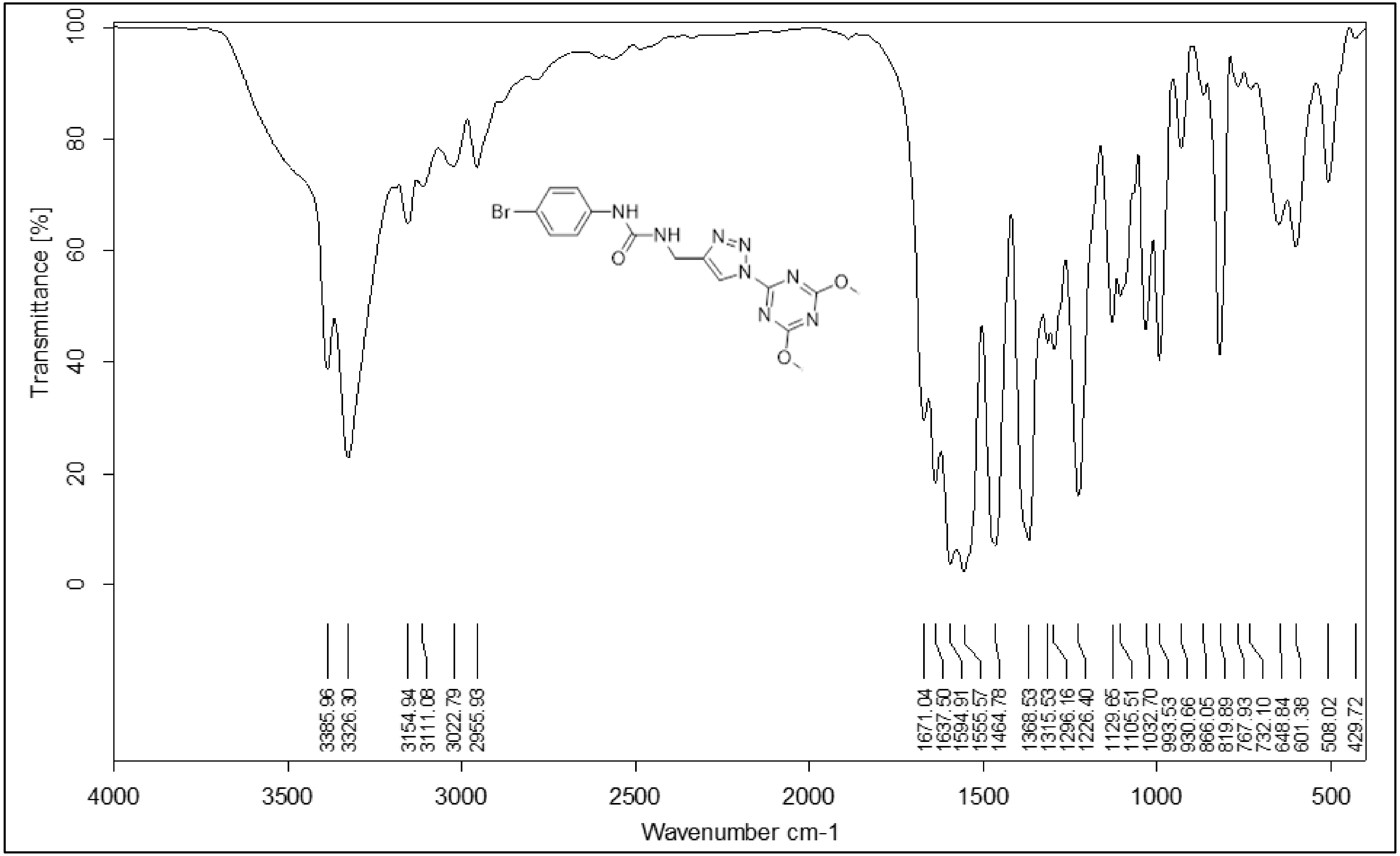

Figure S73: IR (KBr) spectrum of 1-(4-bromophenyl)-3-((1-(4,6-dimethoxy-1,3,5-triazin-2-yl)-1*H*-1,2,3-triazol-4-yl) methyl) urea **(6c)**

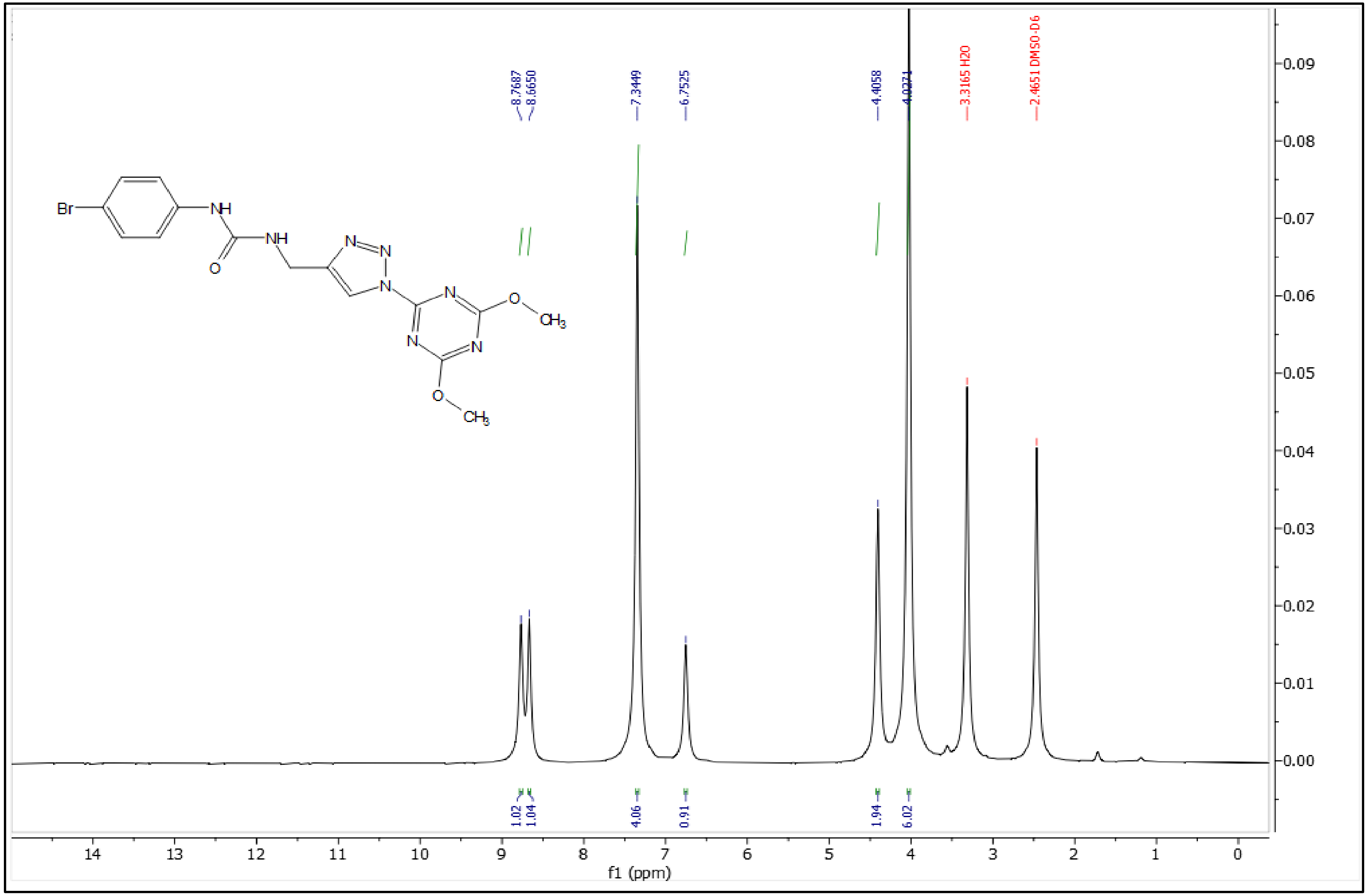

Figure S74: ^{1}H-NMR (500 MHz, DMSO-d_6) spectrum of 1-(4-bromophenyl)-3-((1-(4,6-dimethoxy-1,3,5-triazin-2-yl)-1*H*-1,2,3-triazol-4-yl) methyl) urea **(6c)**

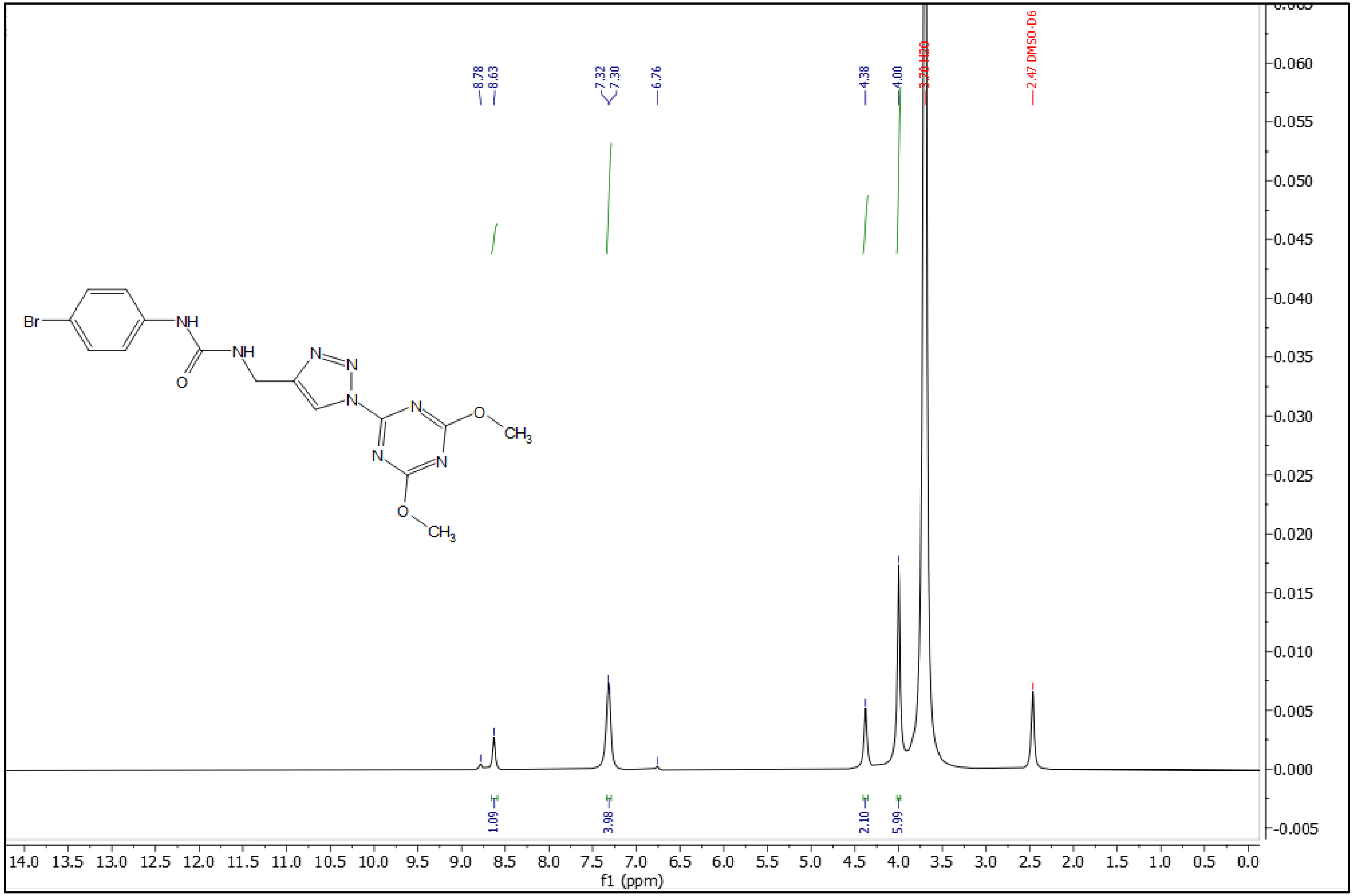

Figure S75: ^{1}H-NMR (500 MHz, DMSO-d_6, D_2O) spectrum of 1-(4-bromophenyl)-3-((1-(4,6-dimethoxy-1,3,5-triazin-2-yl)-1*H*-1,2,3-triazol-4-yl) methyl) urea **(6c)**

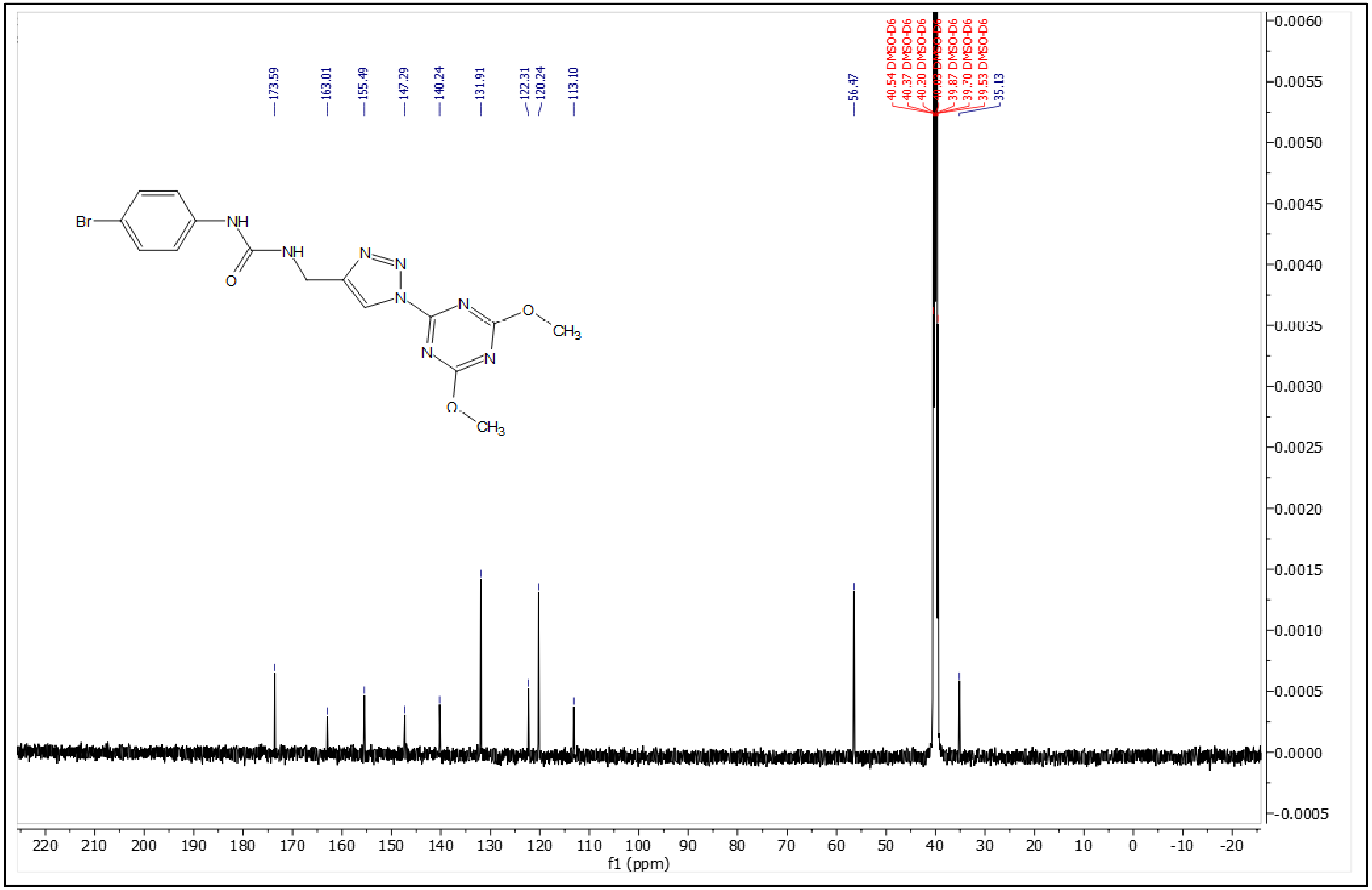

Figure S76: ^{13}C-NMR (DMSO-*d*$_6$) spectrum of 1-(4-bromophenyl)-3-((1-(4,6-dimethoxy-1,3,5-triazin-2-yl)-1*H*-1,2,3-triazol-4-yl) methyl) urea **(6c)**

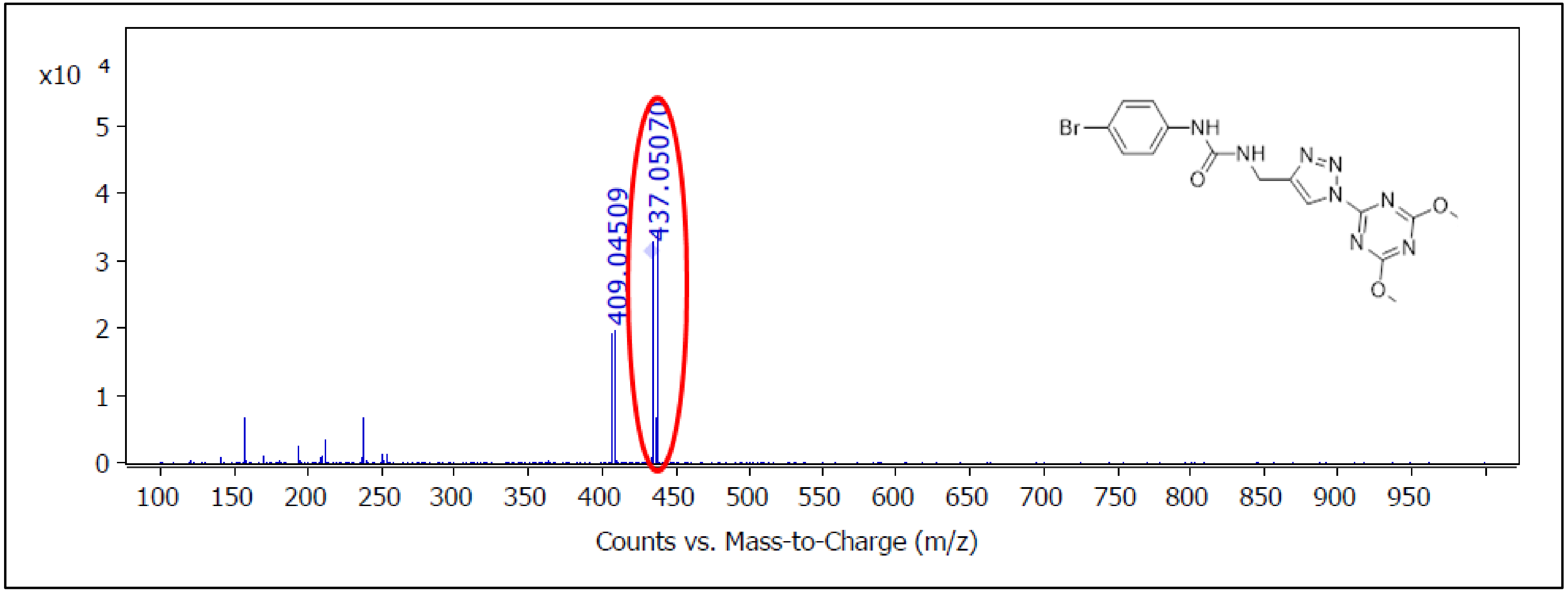

Figure S77: HRMS of 1-(4-bromophenyl)-3-((1-(4,6-dimethoxy-1,3,5-triazin-2-yl)-1*H*-1,2,3-triazol-4-yl) methyl) urea **(6c)**

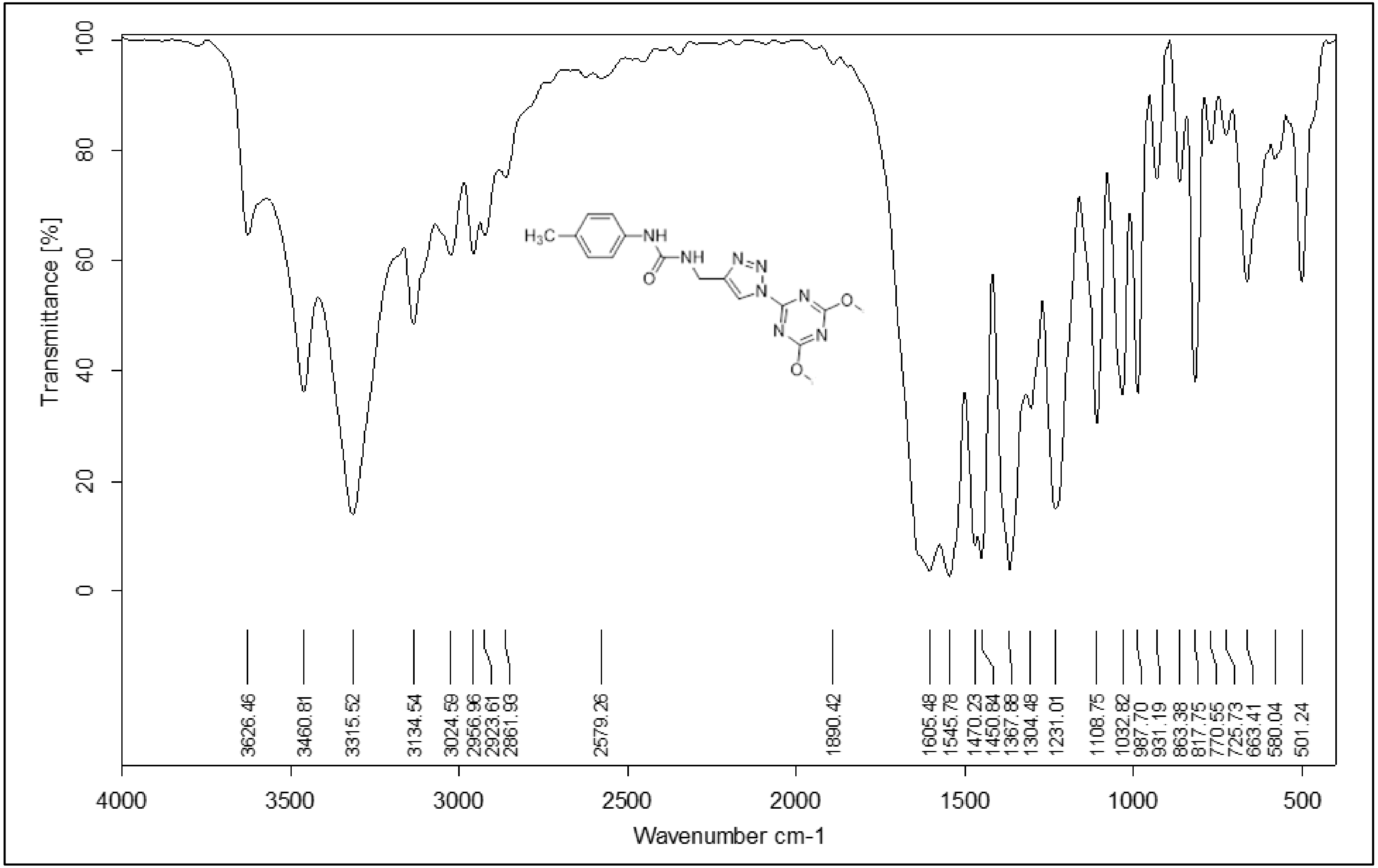

Figure S78: IR (KBr) spectrum of 1-((1-(4,6-dimethoxy-1,3,5-triazin-2-yl)-1*H*-1,2,3-triazol-4-yl) methyl)-3-(p-tolyl) urea **(6d)**

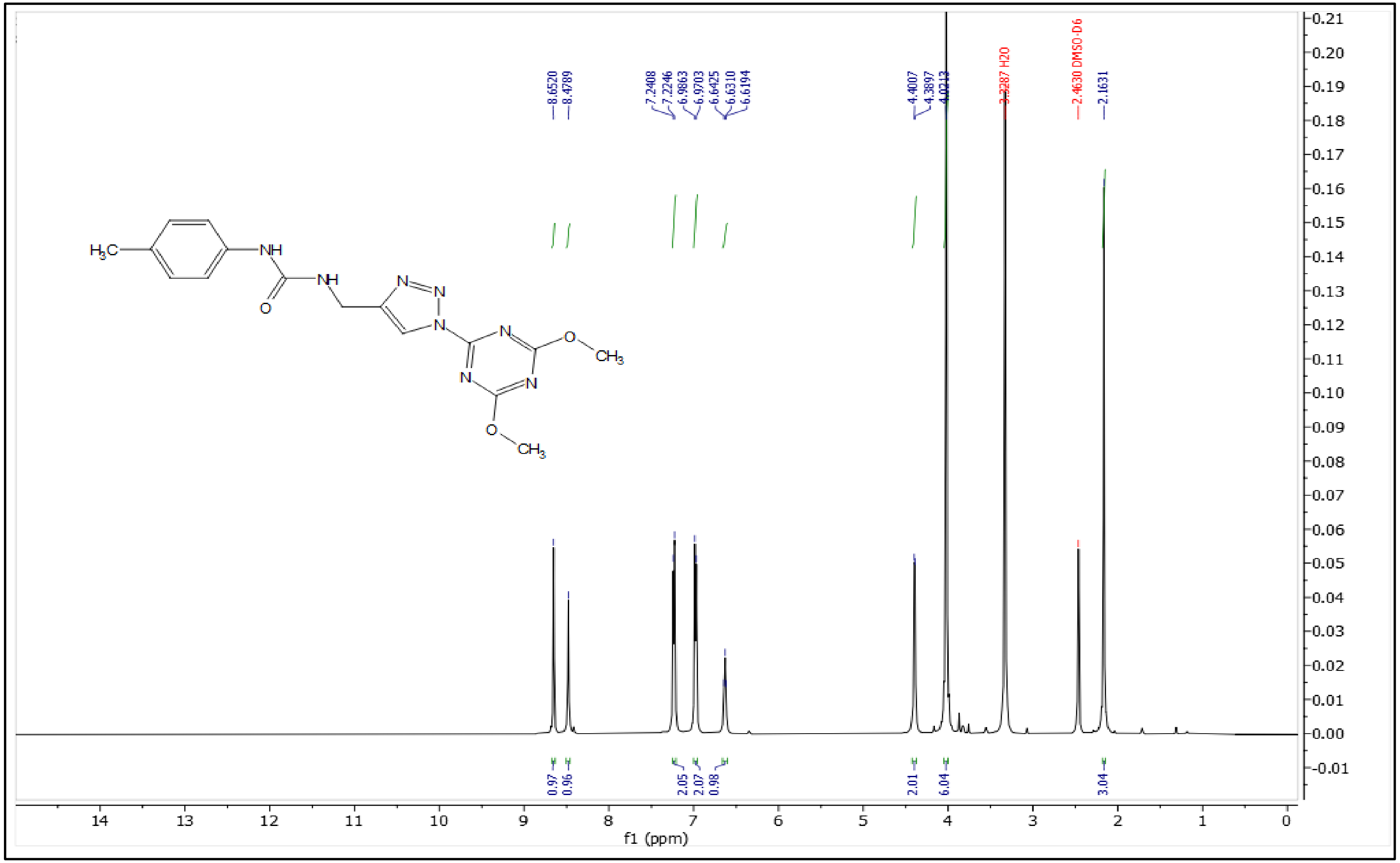

Figure S79: ^{1}H-NMR (500 MHz, DMSO-*d*$_6$) spectrum of 1-((1-(4,6-dimethoxy-1,3,5-triazin-2-yl)-1*H*-1,2,3-triazol-4-yl) methyl)-3-(p-tolyl) urea **(6d)**

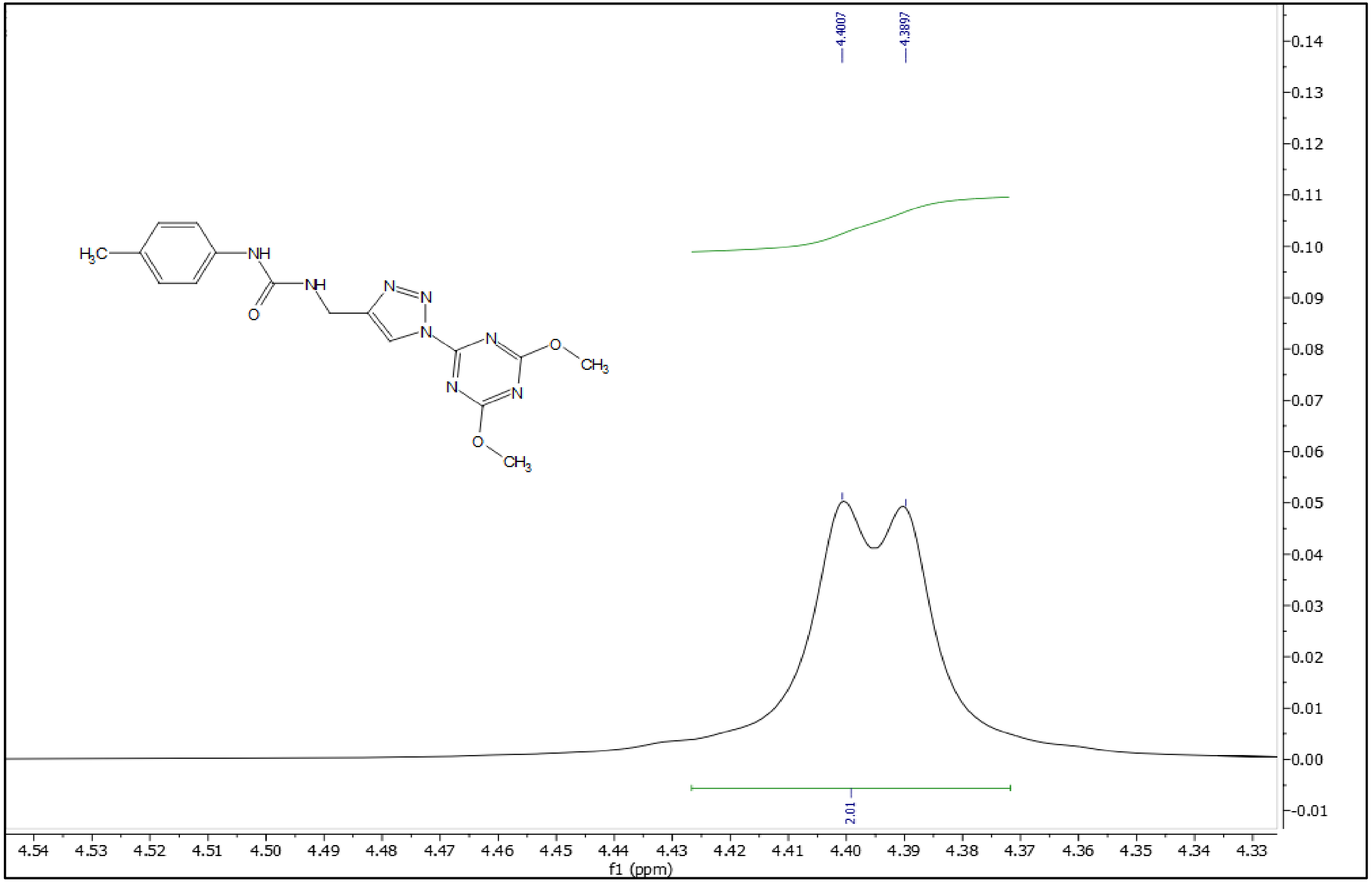

Figure S80: ^{1}H-NMR (500 MHz, DMSO-d_6) spectrum of 1-((1-(4,6-dimethoxy-1,3,5-triazin-2-yl)-1*H*-1,2,3-triazol-4-yl) methyl)-3-(p-tolyl) urea **(6d)**

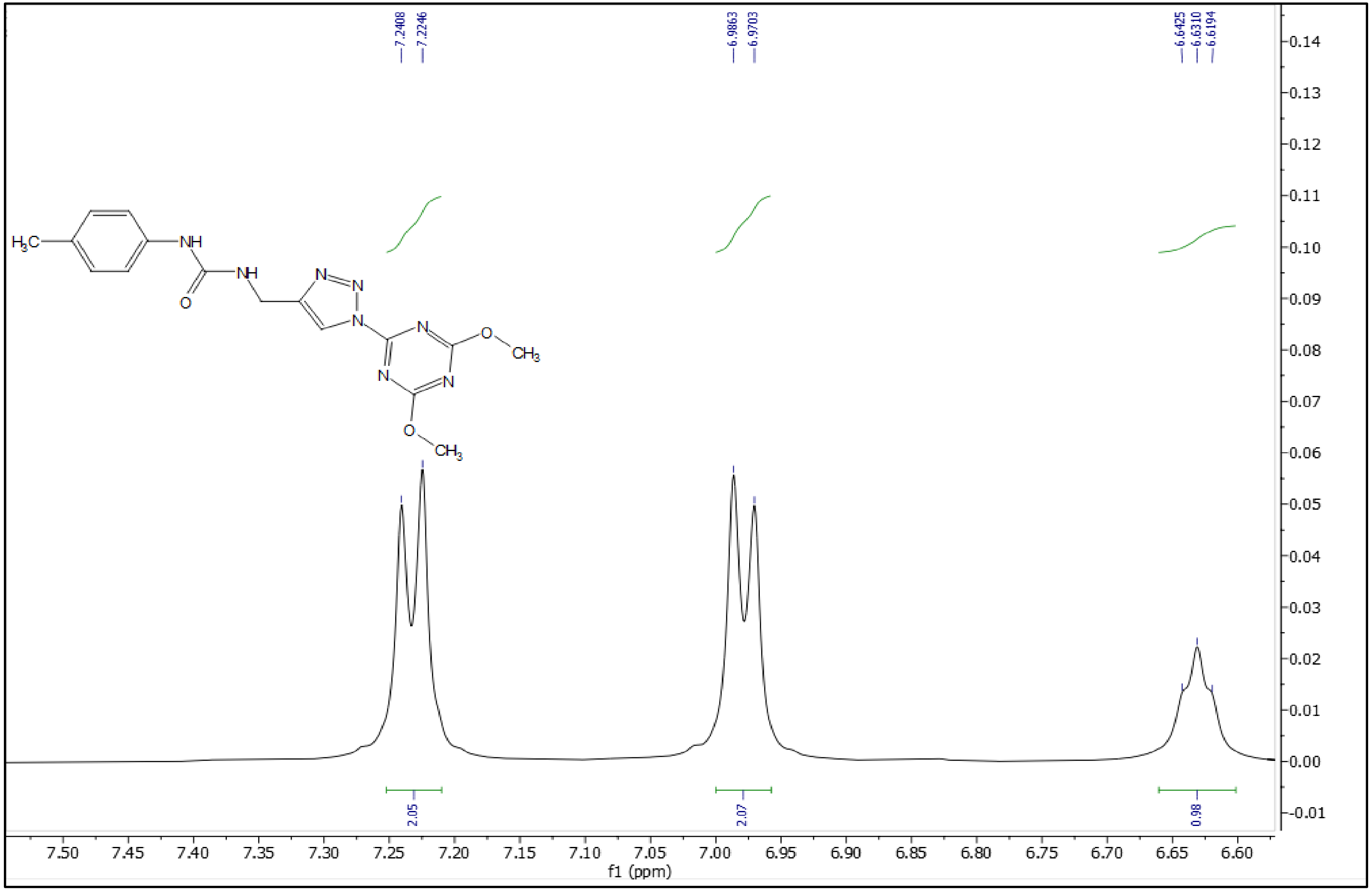

Figure S81: ^{1}H-NMR (500 MHz, DMSO-d_6) spectrum of 1-((1-(4,6-dimethoxy-1,3,5-triazin-2-yl)-1*H*-1,2,3-triazol-4-yl) methyl)-3-(p-tolyl) urea **(6d)**

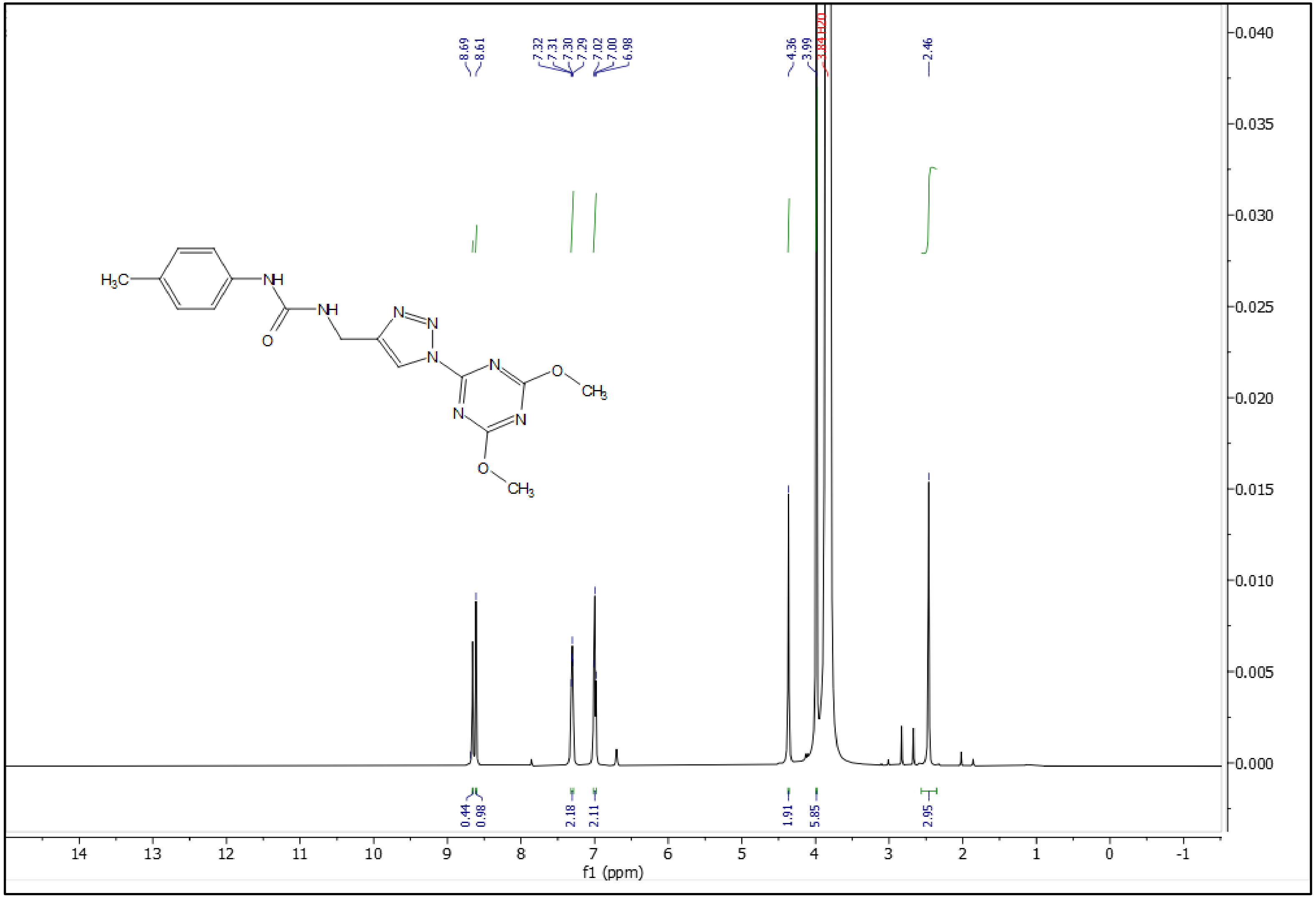

Figure S82: ^{1}H-NMR (500 MHz, DMSO-d_6, D_2O) spectrum of 1-((1-(4,6-dimethoxy-1,3,5-triazin-2-yl)-1*H*-1,2,3-triazol-4-yl) methyl)-3-(p-tolyl) urea **(6d)**

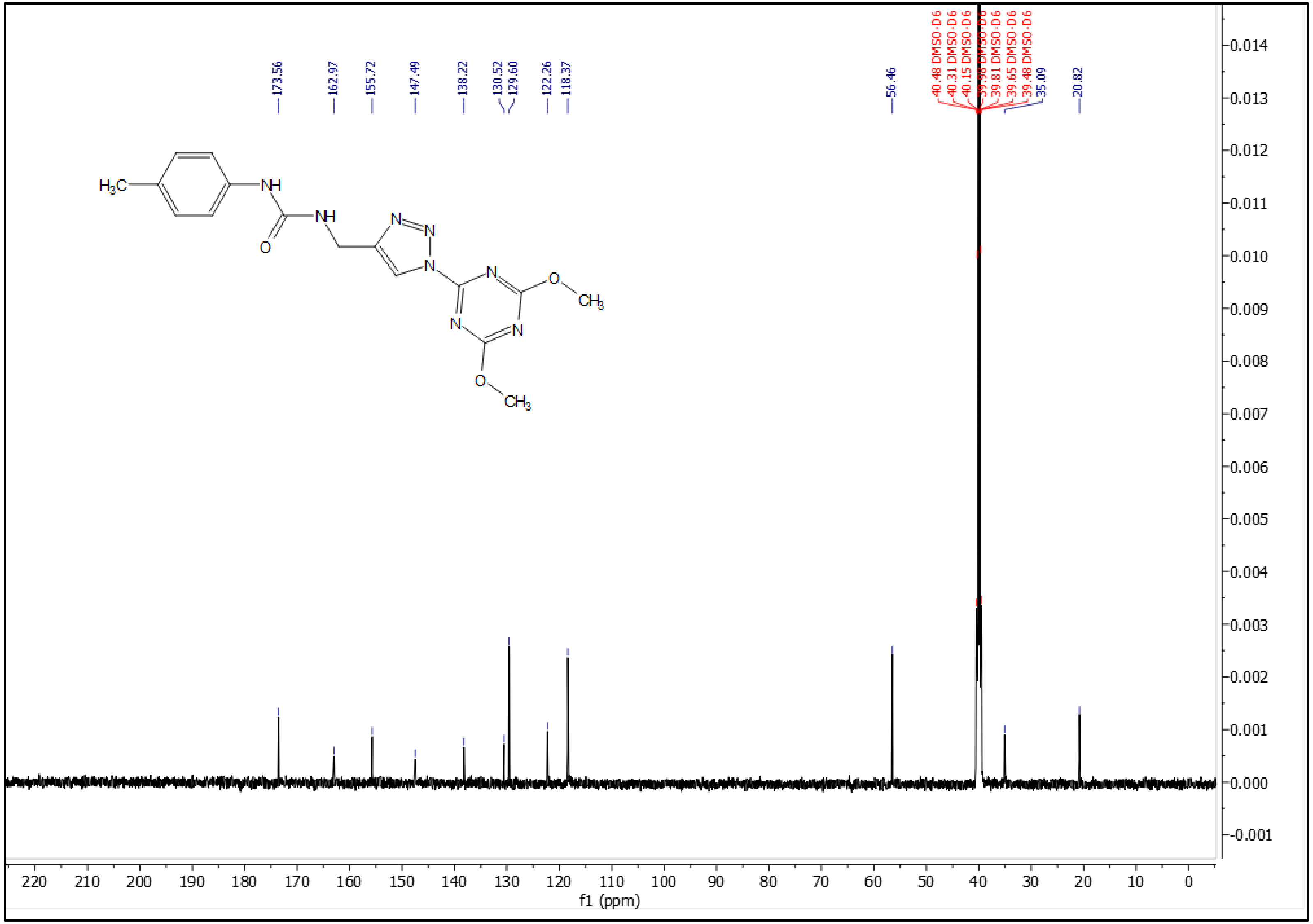

Figure S83: ^{13}C-NMR (DMSO-d_6) spectrum of 1-((1-(4,6-dimethoxy-1,3,5-triazin-2-yl)-1*H*-1,2,3-triazol-4-yl) methyl)-3-(p-tolyl) urea **(6d)**

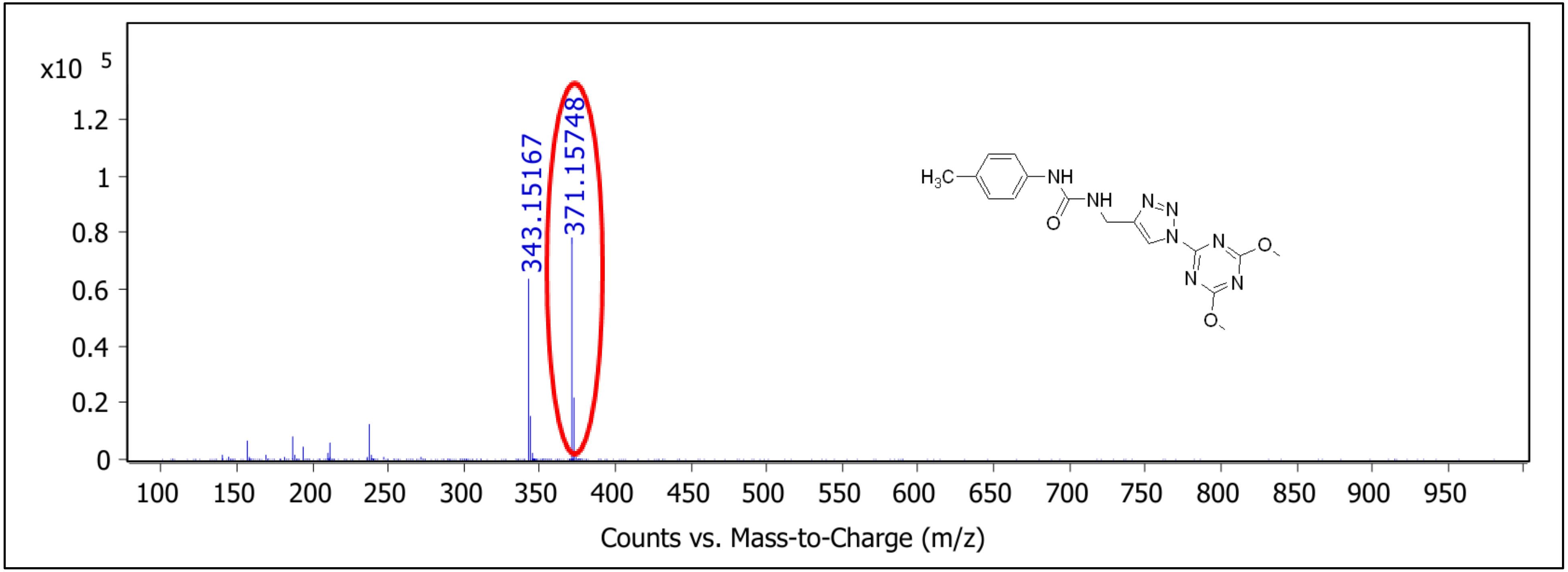

Figure S84: HRMS of 1-((1-(4,6-dimethoxy-1,3,5-triazin-2-yl)-1*H*-1,2,3-triazol-4-yl) methyl)-3-(p-tolyl) urea **(6d)**

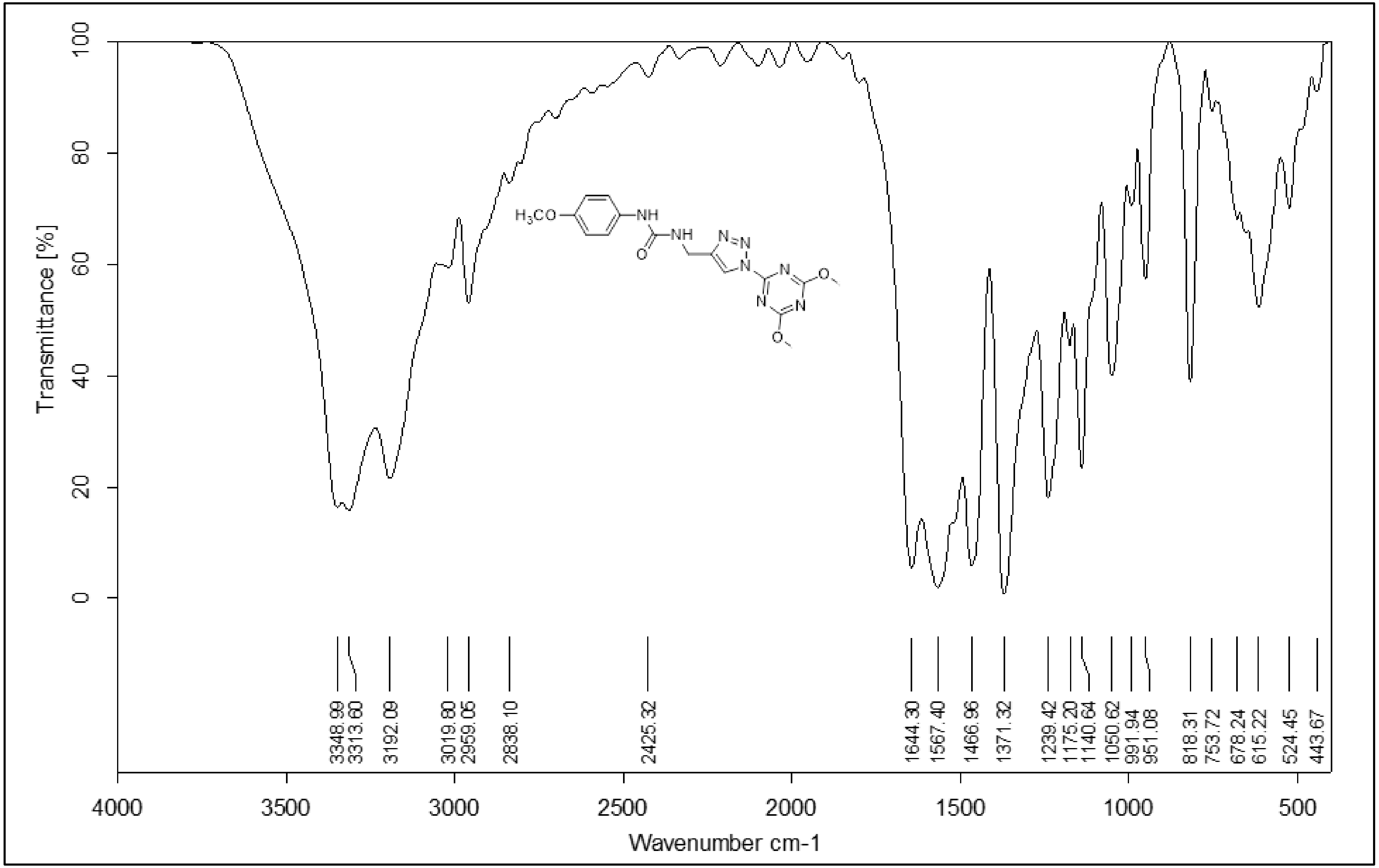

Figure S85: IR (KBr) spectrum of 1-((1-(4,6-dimethoxy-1,3,5-triazin-2-yl)-1*H*-1,2,3-triazol-4-yl) methyl)-3-(4-methoxyphenyl) urea **(6e)**

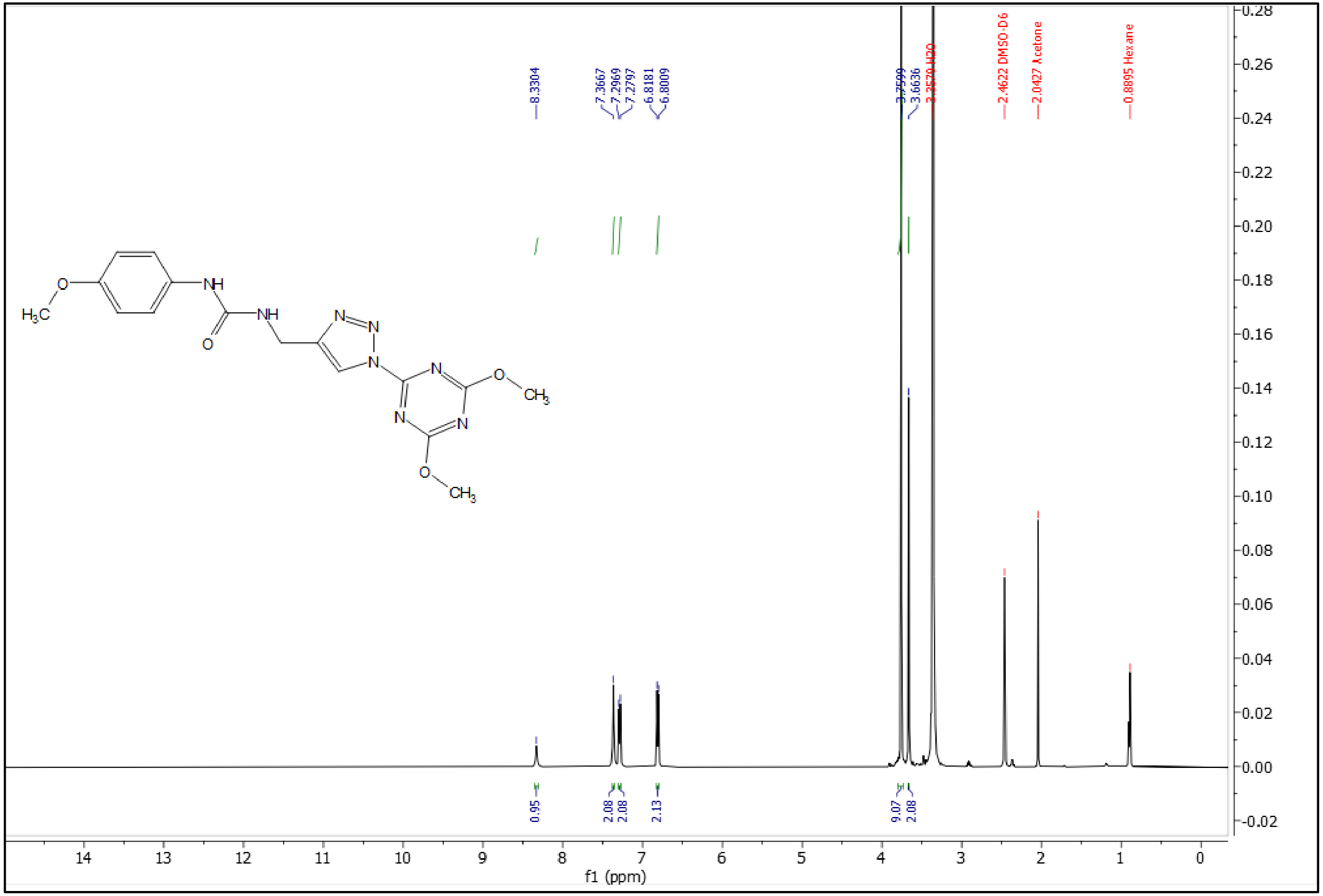

Figure S86: ^{1}H-NMR (500 MHz, DMSO-d_6) spectrum of 1-((1-(4,6-dimethoxy-1,3,5-triazin-2-yl)-1*H*-1,2,3-triazol-4-yl) methyl)-3-(4-methoxy-phenyl) urea **(6e).**

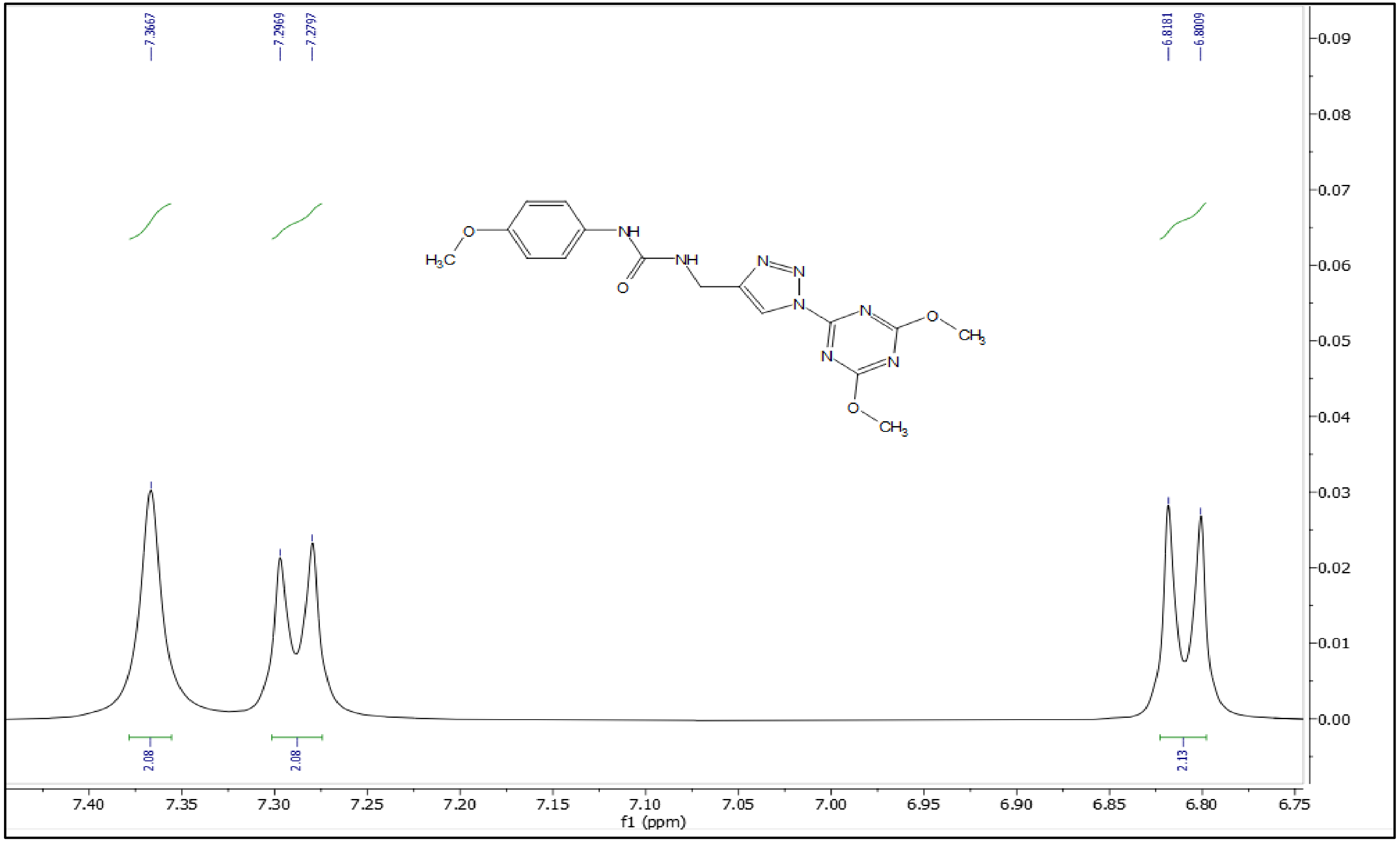

Figure S87: ^{1}H-NMR (500 MHz, DMSO-d_6) spectrum of 1-((1-(4,6-dimethoxy-1,3,5-triazin-2-yl)-1*H*-1,2,3-triazol-4-yl) methyl)-3-(4-methoxyphenyl) urea **(6e)**

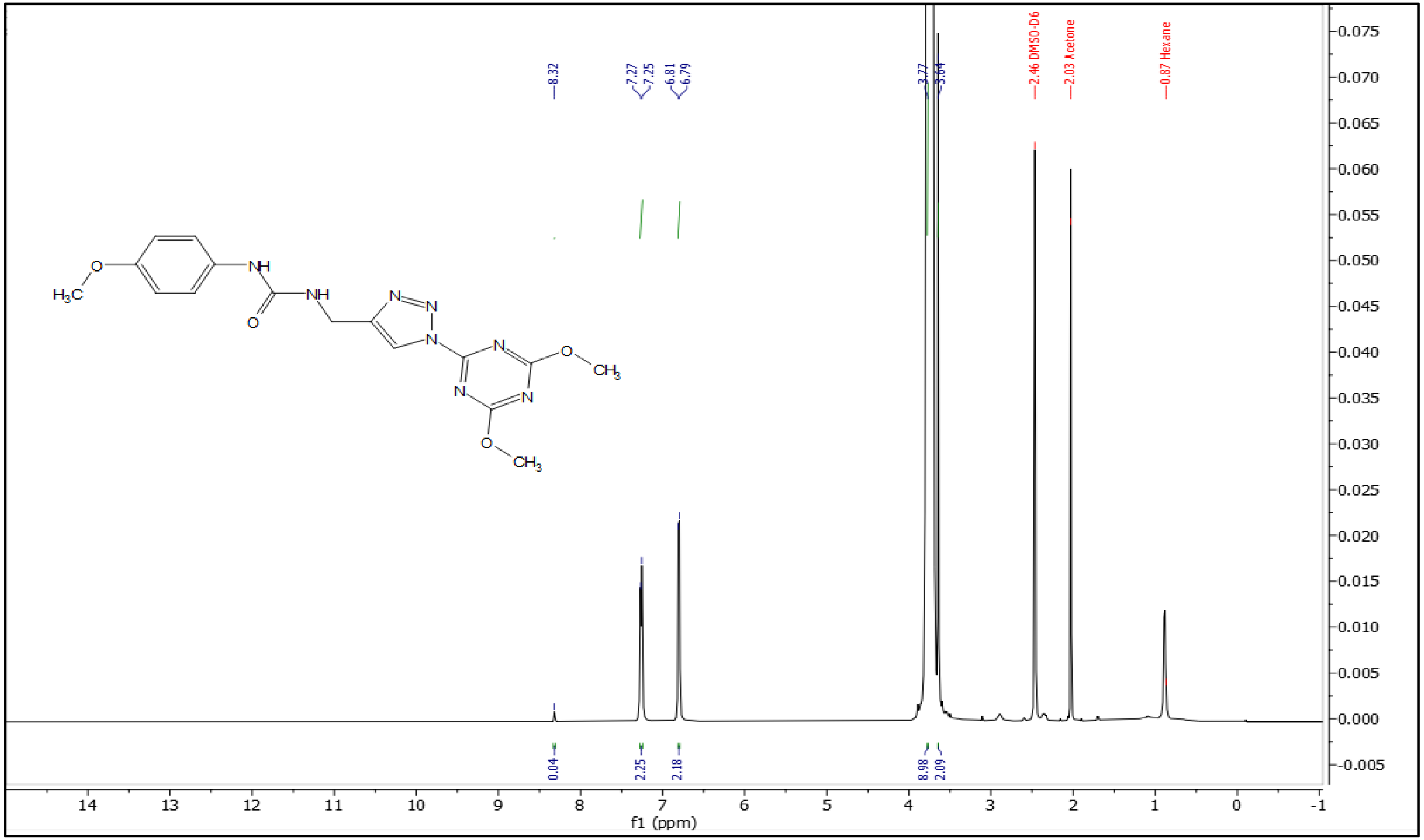

Figure S88: ^{1}H-NMR (500 MHz, DMSO-d_6, D_2O) spectrum of 1-((1-(4,6-dimethoxy-1,3,5-triazin-2-yl)-1*H*-1,2,3-triazol-4-yl) methyl)-3-(4-methoxy-phenyl) urea **(6e)**

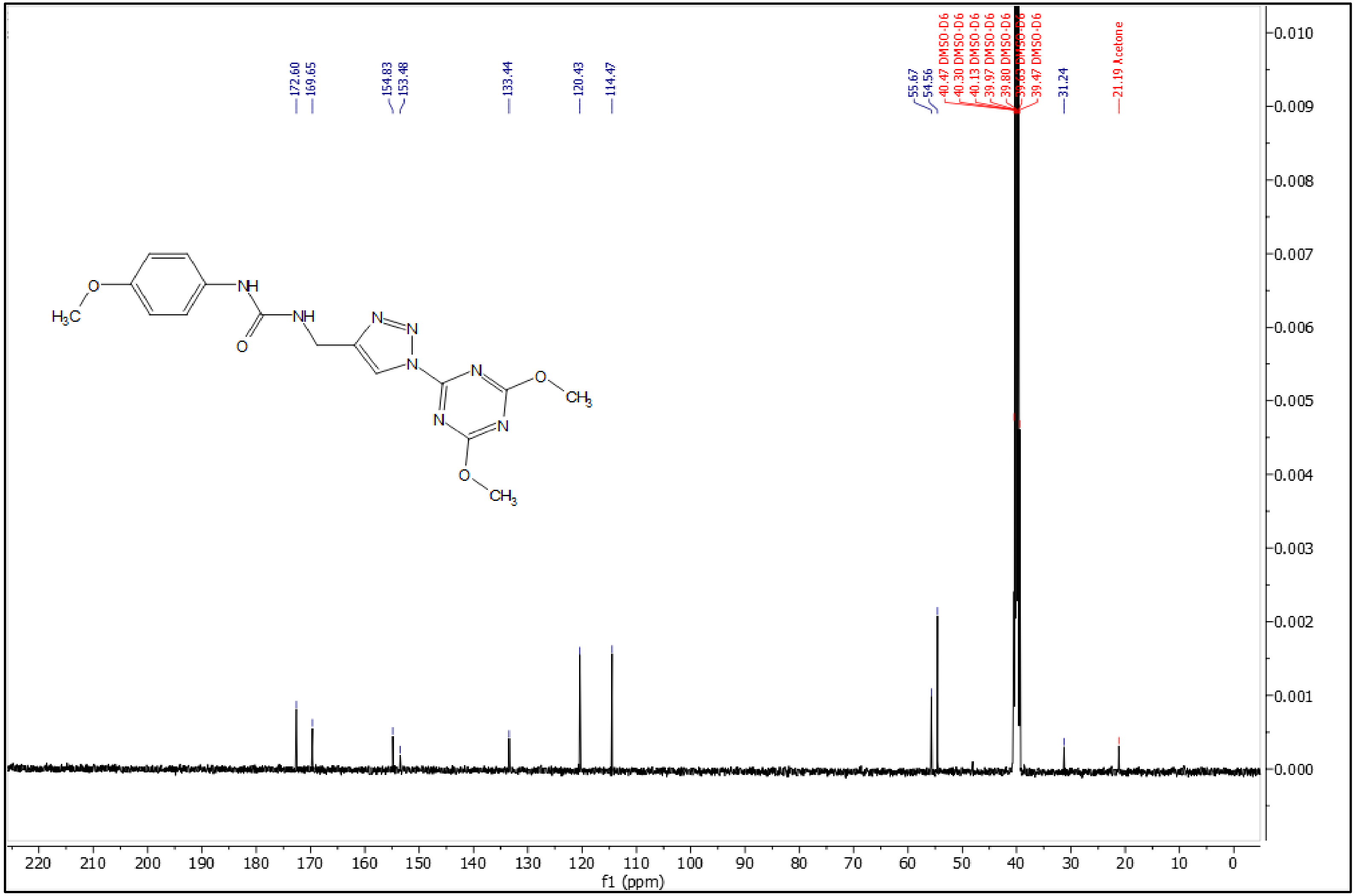

Figure S89: ^{13}C-NMR (DMSO-d_6) spectrum of 1-((1-(4,6-dimethoxy-1,3,5-triazin-2-yl)-1*H*-1,2,3-triazol-4-yl) methyl)-3-(4-methoxyphenyl) urea **(6e)**

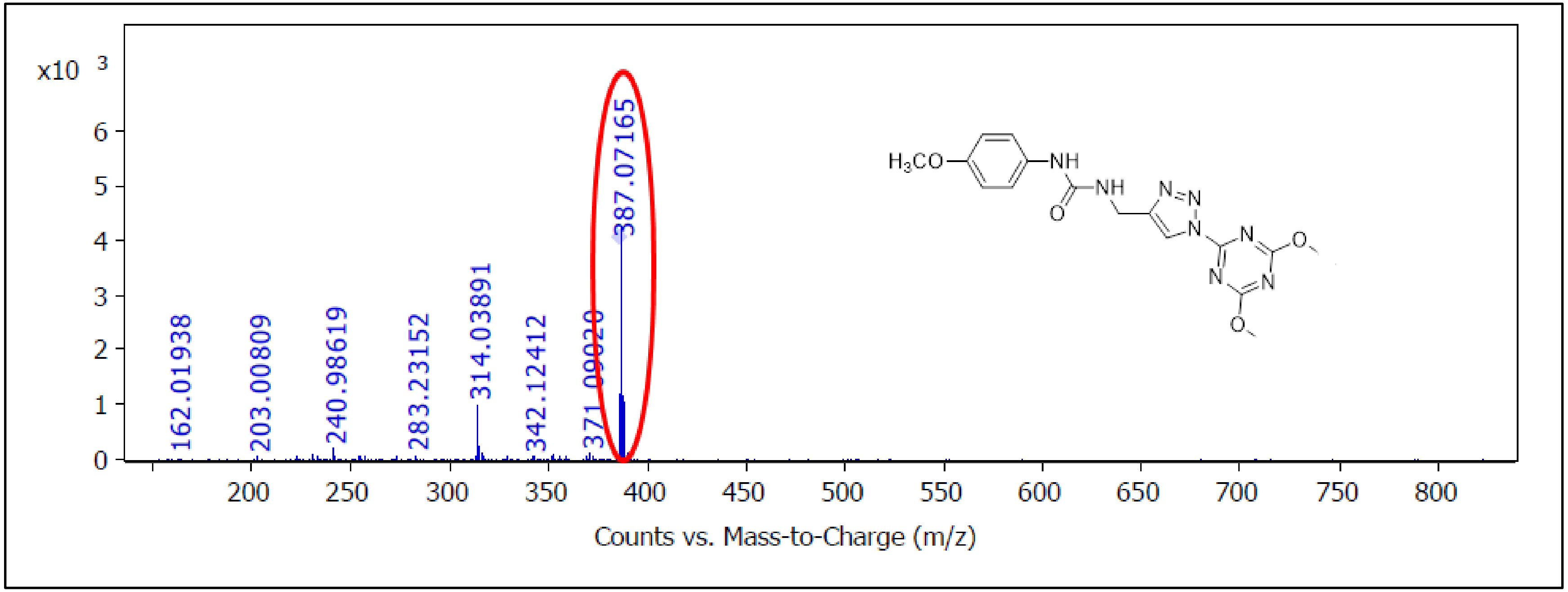

Figure S90: HRMS of 1-((1-(4,6-dimethoxy-1,3,5-triazin-2-yl)-1*H*-1,2,3-triazol-4-yl) methyl)-3-(4-methoxyphenyl) urea **(6e)**

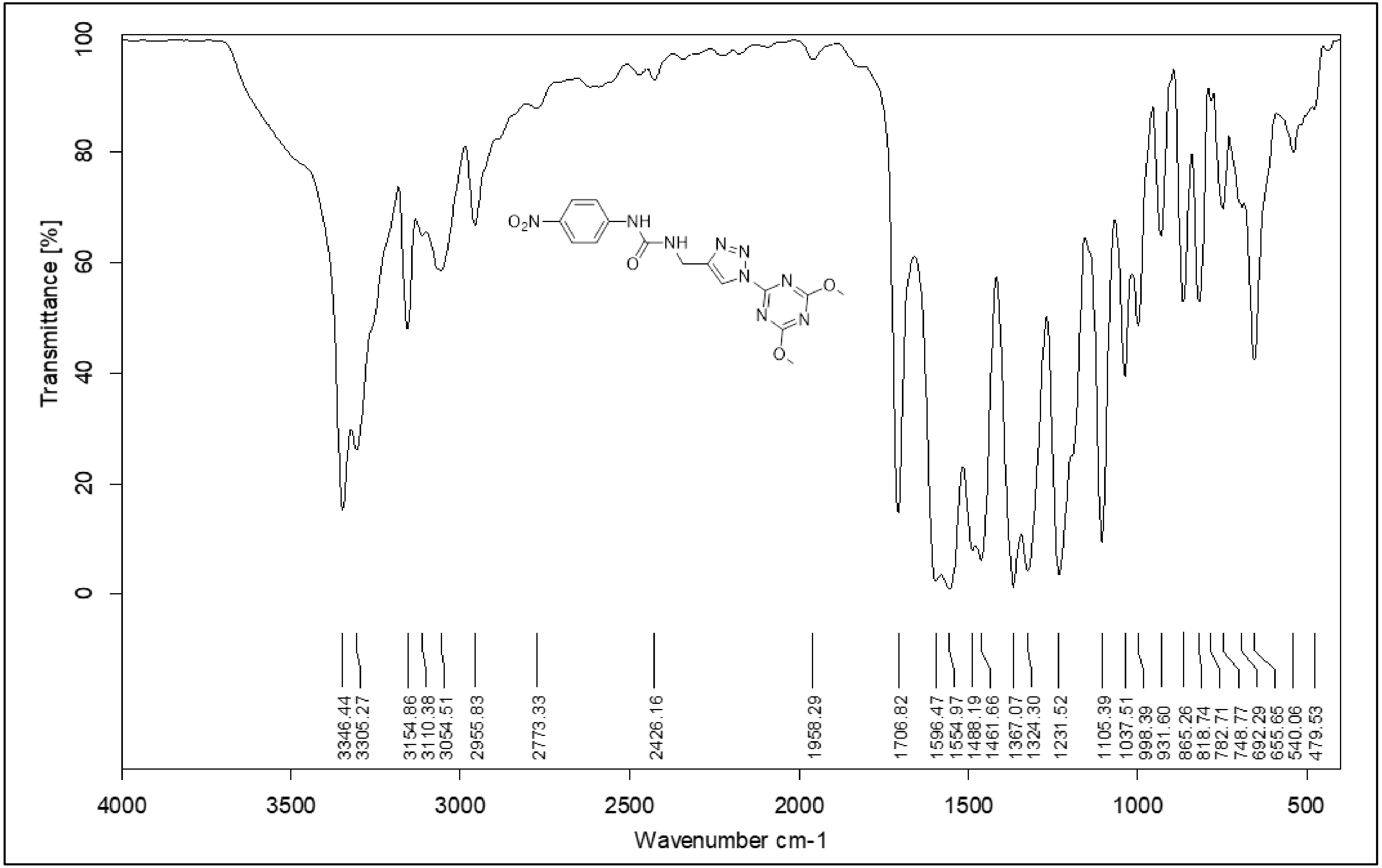

Figure S91: IR (KBr) spectrum of 1-((1-(4,6-dimethoxy-1,3,5-triazin-2-yl)-1*H*-1,2,3-triazol-4-yl) methyl)-3-(4-nitrophenyl) urea **(6f)**

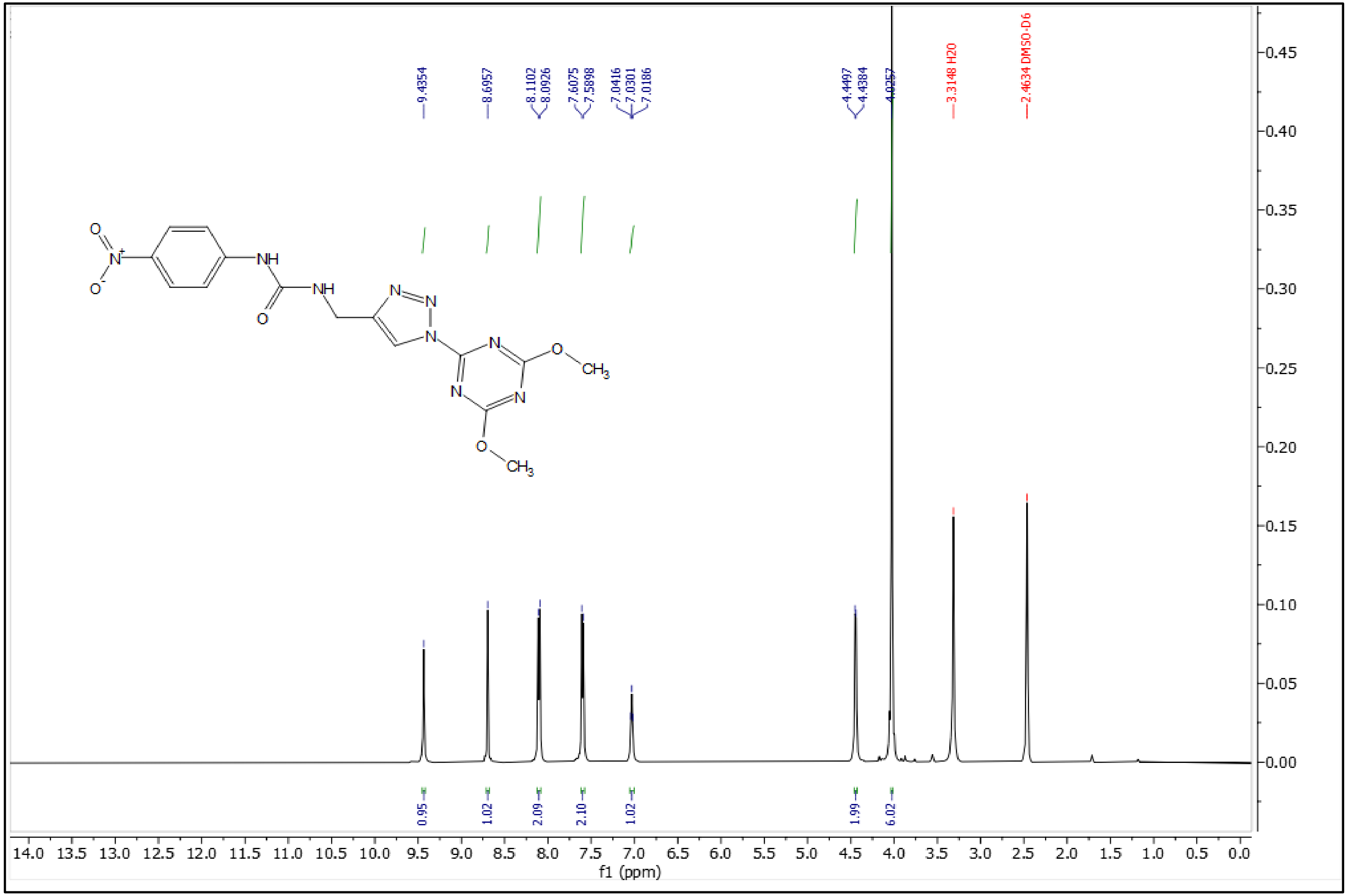

Figure S92: ^{1}H-NMR (500 MHz, DMSO-d_6) spectrum of 1-((1-(4,6-dimethoxy-1,3,5-triazin-2-yl)-1*H*-1,2,3-triazol-4-yl) methyl)-3-(4-nitrophenyl) urea **(6f)**

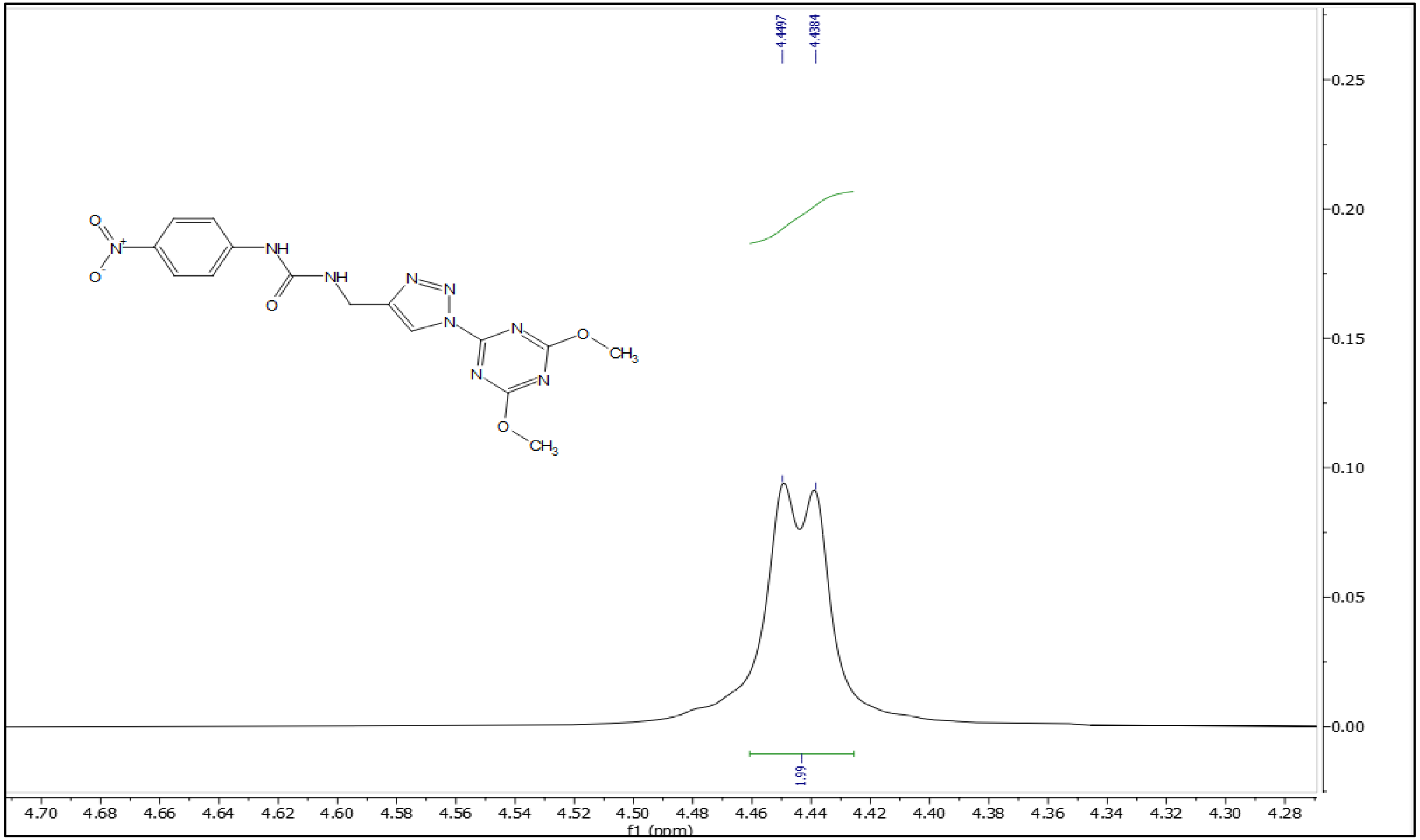

Figure S93: ^{1}H-NMR (500 MHz, DMSO-d_6) spectrum of 1-((1-(4,6-dimethoxy-1,3,5-triazin-2-yl)-1*H*-1,2,3-triazol-4-yl) methyl)-3-(4-nitrophenyl) urea **(6f)**

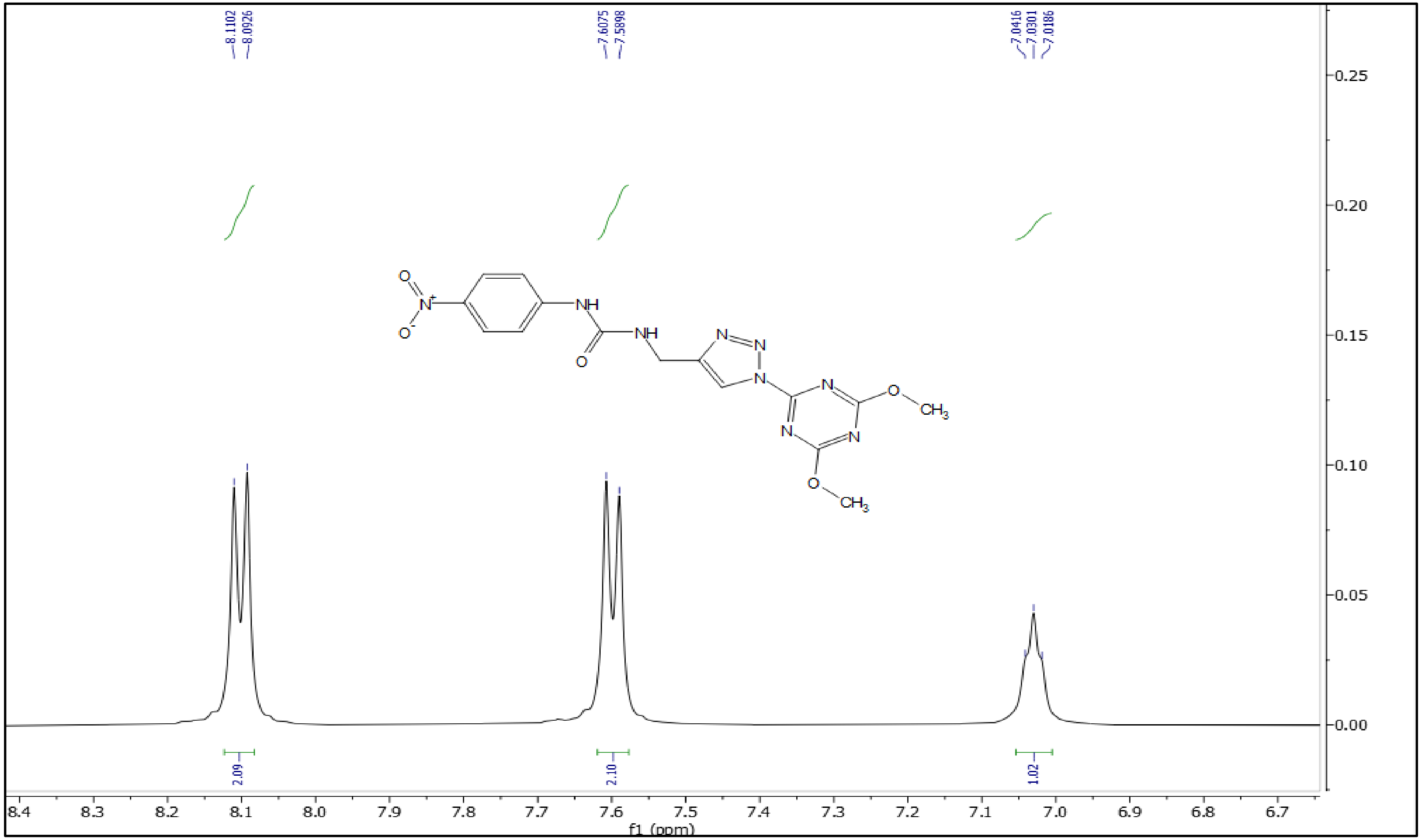

Figure S94: ^{1}H-NMR (500 MHz, DMSO-d_6) spectrum of 1-((1-(4,6-dimethoxy-1,3,5-triazin-2-yl)-1*H*-1,2,3-triazol-4-yl) methyl)-3-(4-nitrophenyl) urea **(6f)**

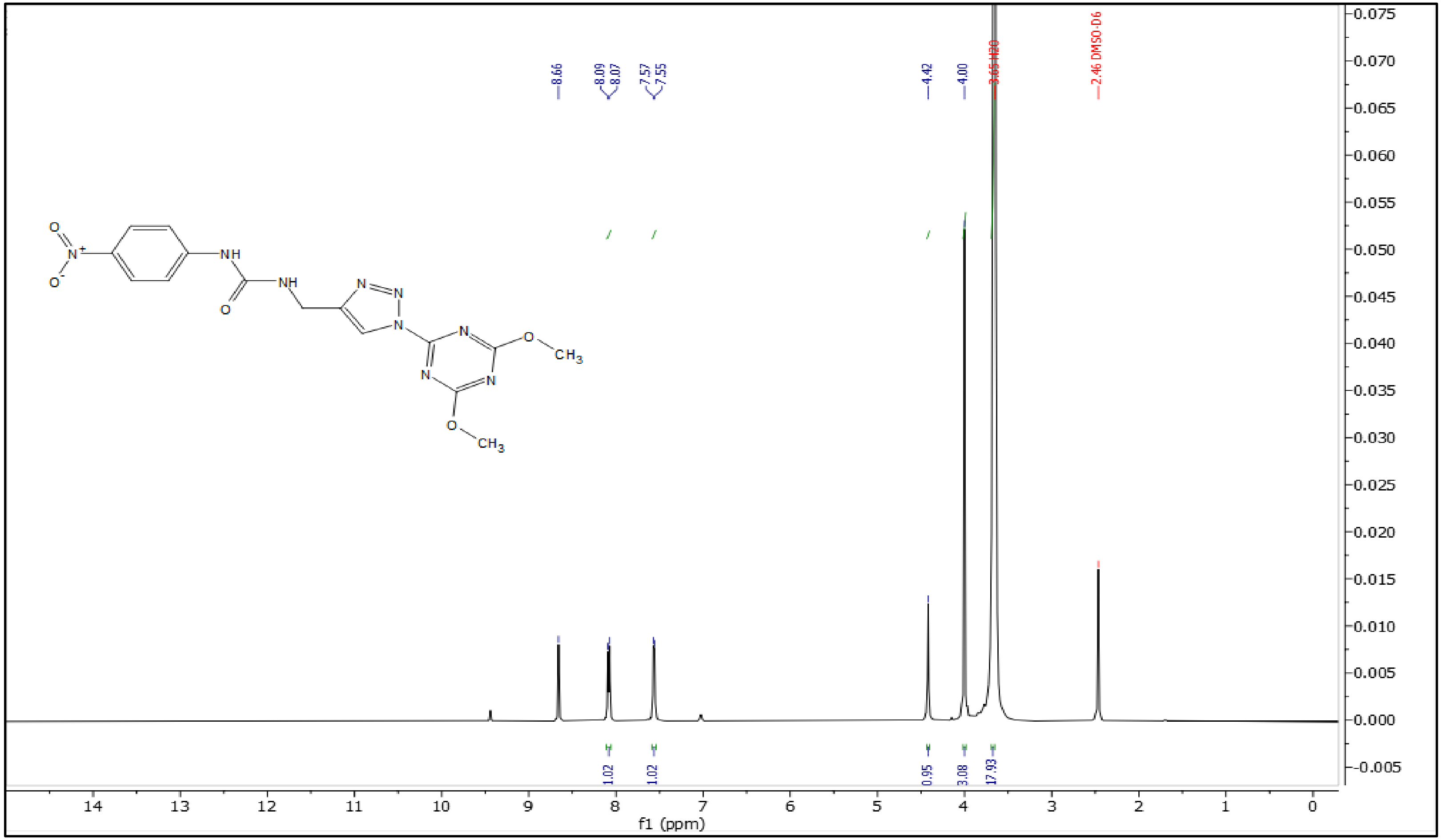

Figure S95: ^{1}H-NMR (500 MHz, DMSO-d_6, D_2O) spectrum of 1-((1-(4,6-dimethoxy-1,3,5-triazin-2-yl)-1*H*-1,2,3-triazol-4-yl) methyl)-3-(4-nitro-phenyl) urea **(6f)**

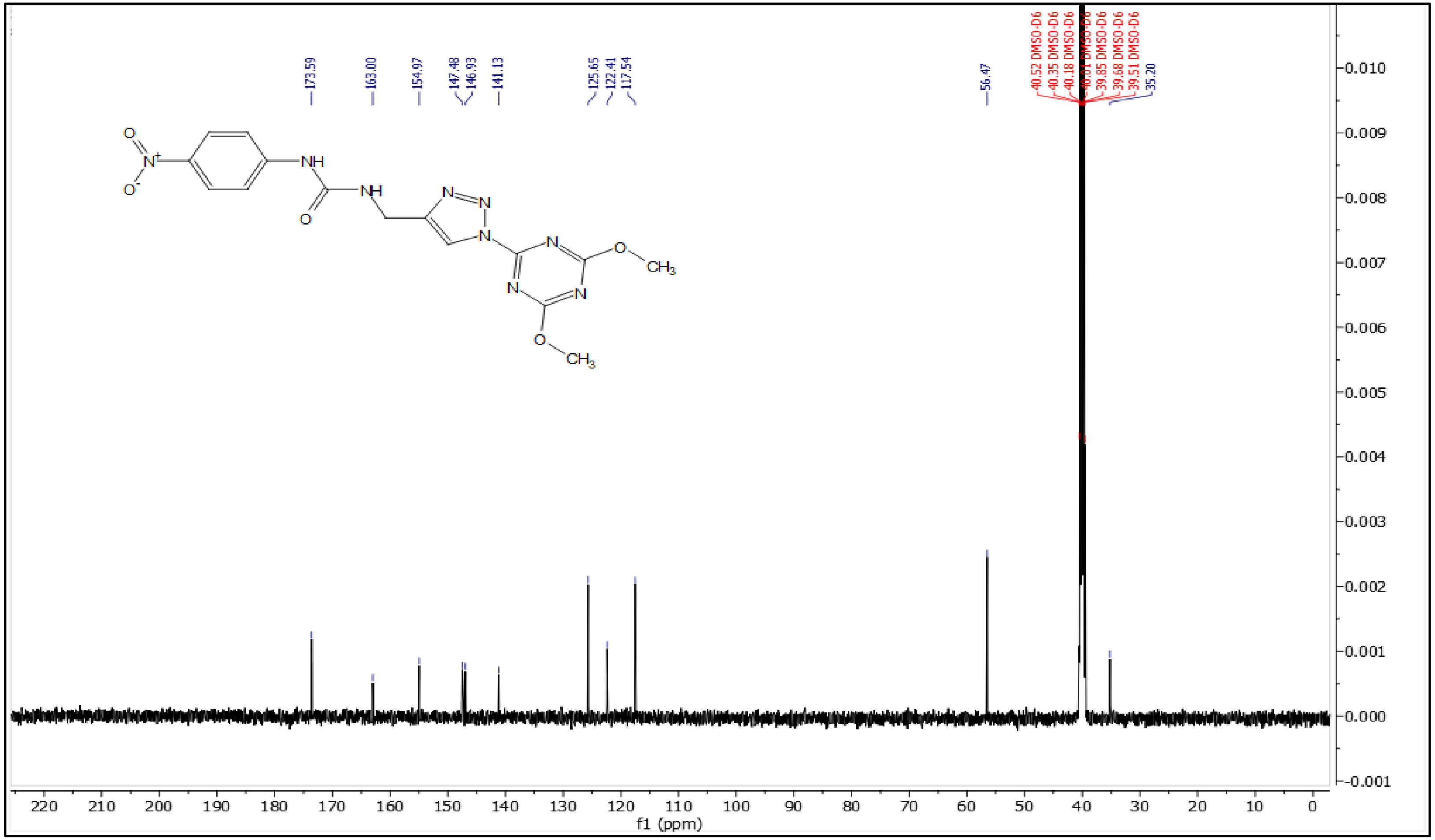

Figure S96: ^{13}C-NMR (DMSO-d_6) spectrum of 1-((1-(4,6-dimethoxy-1,3,5-triazin-2-yl)-1*H*-1,2,3-triazol-4-yl) methyl)-3-(4-nitrophenyl) urea **(6f)**

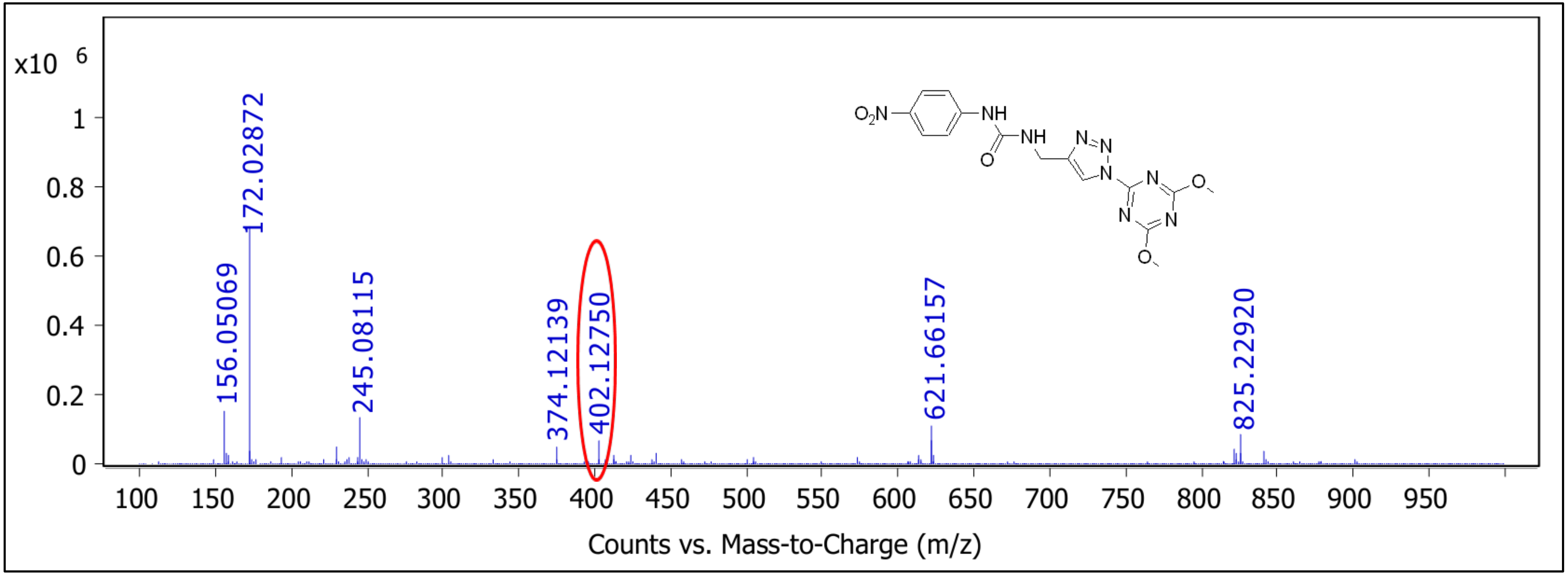

Figure S97: HRMS of 1-((1-(4,6-dimethoxy-1,3,5-triazin-2-yl)-1*H*-1,2,3-triazol-4-yl) methyl)-3-(4-nitrophenyl) urea **(6f)**

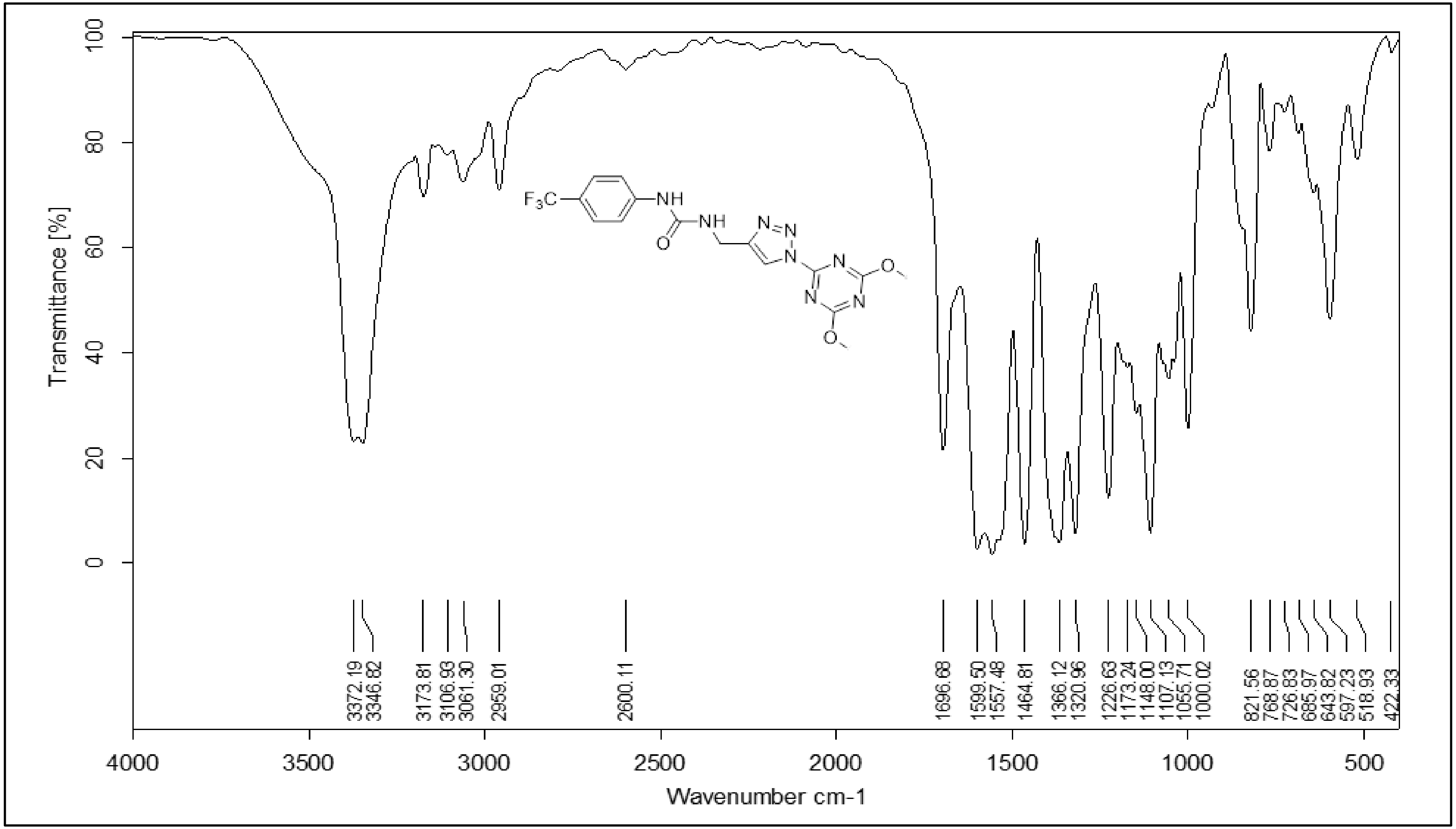

Figure S98: IR (KBr) spectrum of 1-((1-(4,6-dimethoxy-1,3,5-triazin-2-yl)-1*H*-1,2,3-triazol-4-yl) methyl)-3-(4-(trifluoromethyl) phenyl) urea **(6g)**

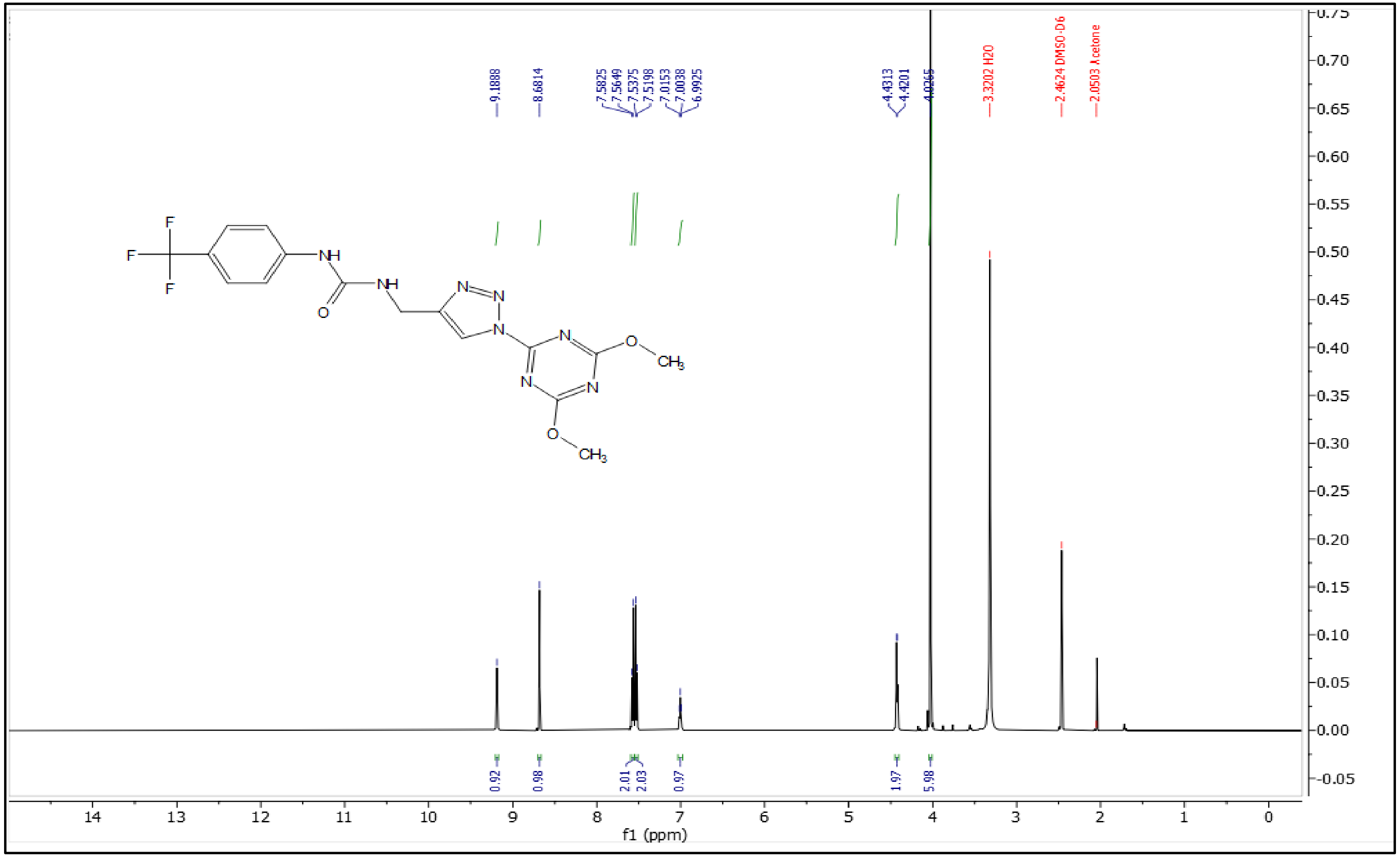

Figure S99: ^{1}H-NMR (500 MHz, DMSO-d_6) spectrum of 1-((1-(4,6-dimethoxy-1,3,5-triazin-2-yl)-1*H*-1,2,3-triazol-4-yl) methyl)-3-(4-(trifluoromethyl) phenyl) urea **(6g)**

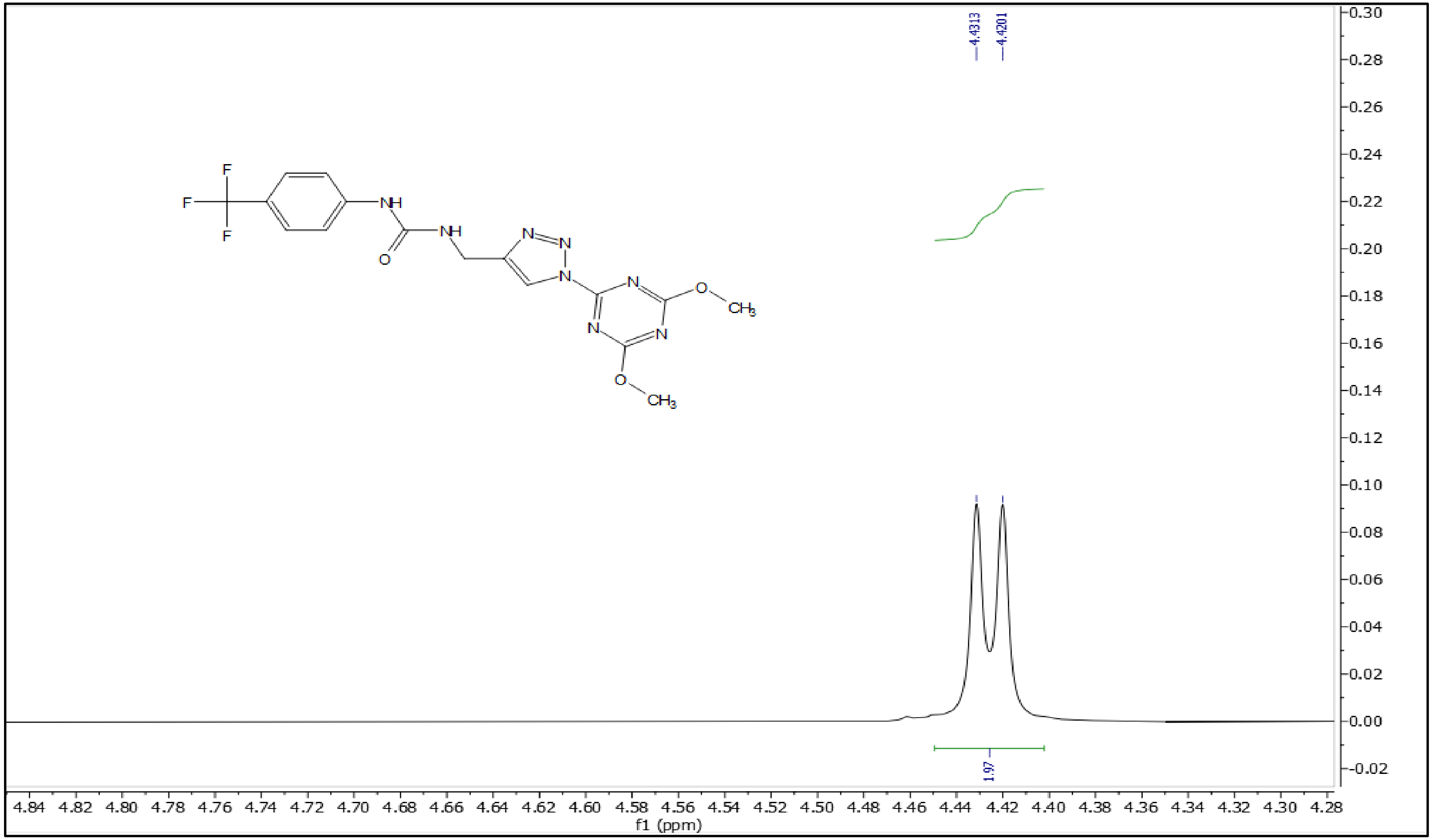

Figure S100: ^{1}H-NMR (500 MHz, DMSO-d_6) spectrum of 1-((1-(4,6-dimethoxy-1,3,5-triazin-2-yl)-1*H*-1,2,3-triazol-4-yl) methyl)-3-(4-(trifluoromethyl) phenyl) urea **(6g)**

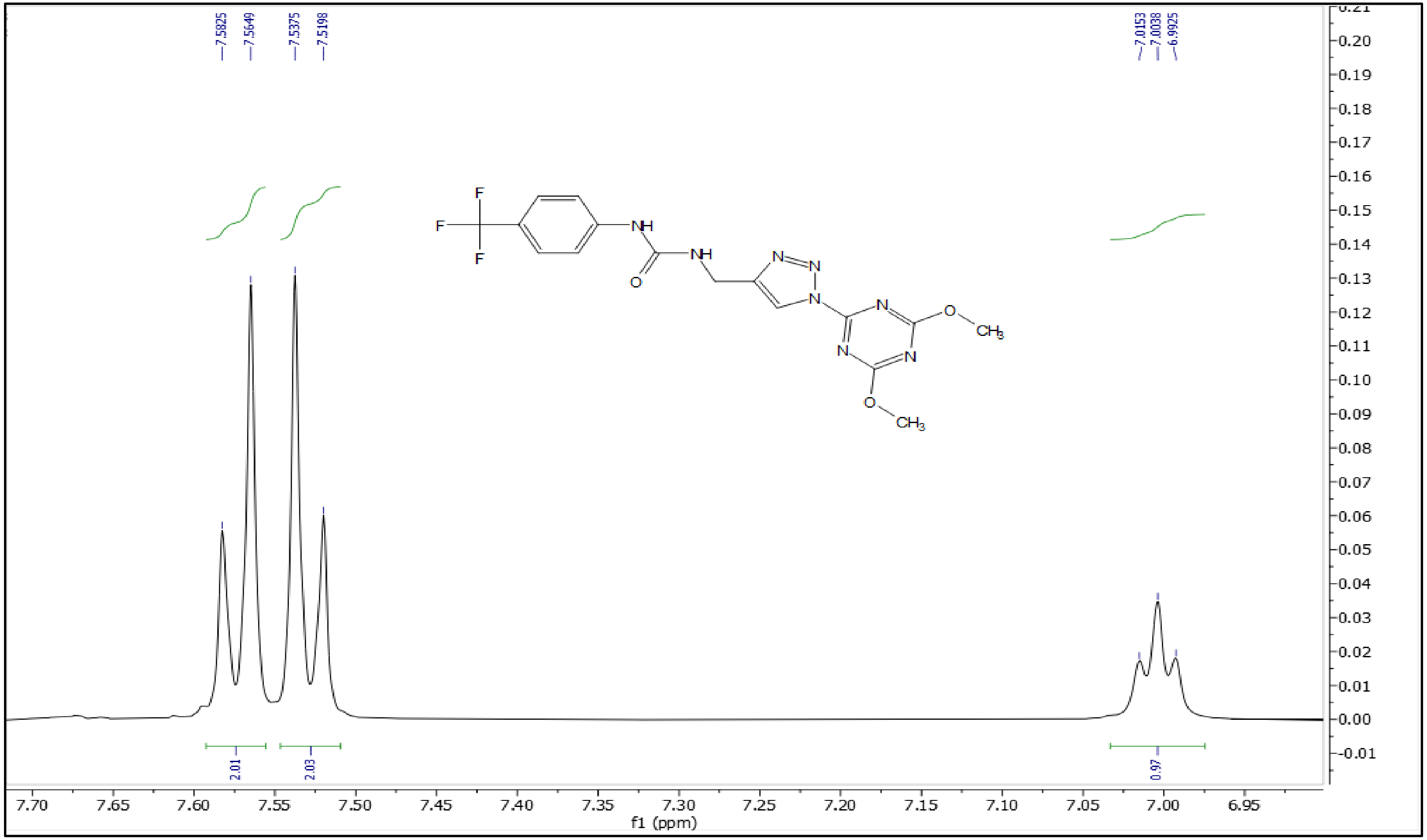

Figure S101: ^{1}H-NMR (500 MHz, DMSO-d_6) spectrum of 1-((1-(4,6-dimethoxy-1,3,5-triazin-2-yl)-1*H*-1,2,3-triazol-4-yl) methyl)-3-(4-(trifluoromethyl) phenyl) urea **(6g)**

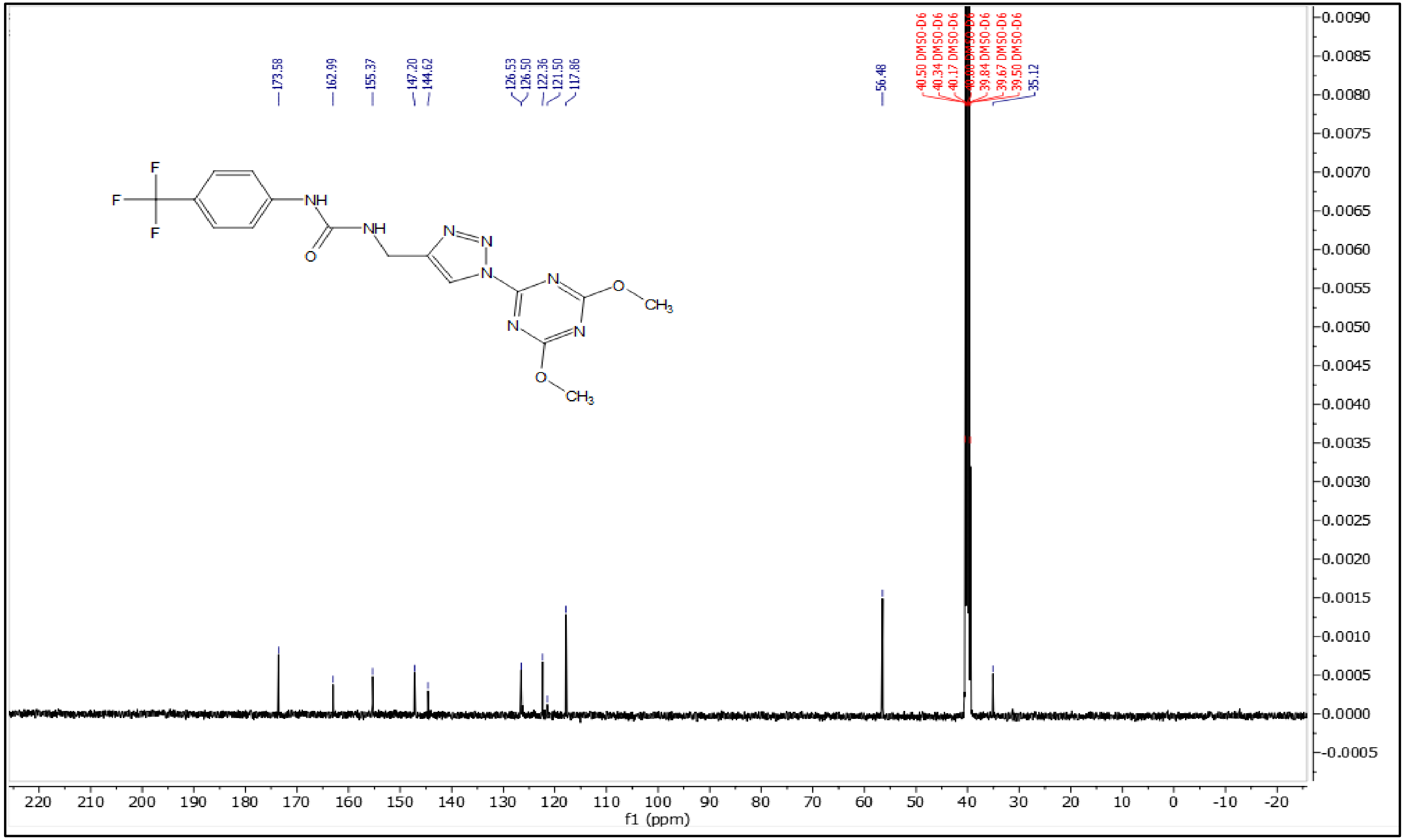

Figure S102: ^{13}C-NMR (DMSO-*d*$_6$) spectrum of 1-((1-(4,6-dimethoxy-1,3,5-triazin-2-yl)-1*H*-1,2,3-triazol-4-yl) methyl)-3-(4-(trifluoromethyl) phenyl) urea **(6g)**

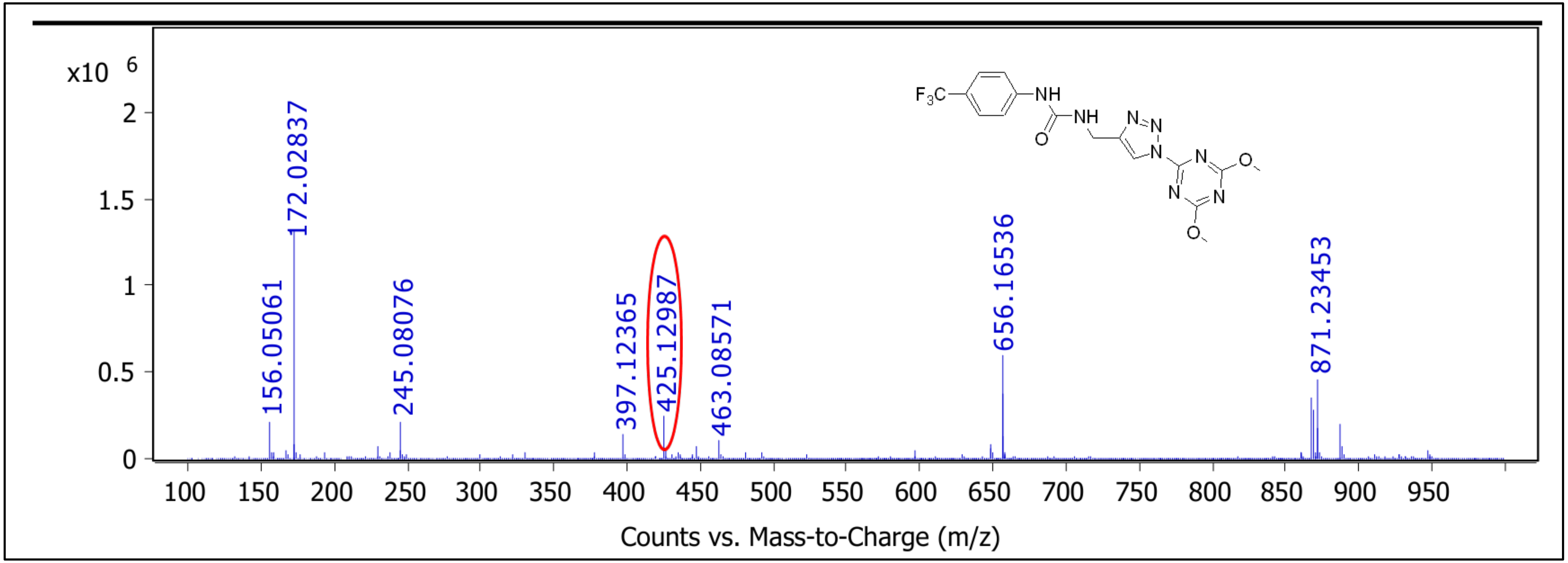

Figure S103: HRMS of 1-((1-(4,6-dimethoxy-1,3,5-triazin-2-yl)-1*H*-1,2,3-triazol-4-yl) methyl)-3-(4-(trifluoromethyl) phenyl) urea **(6g)**

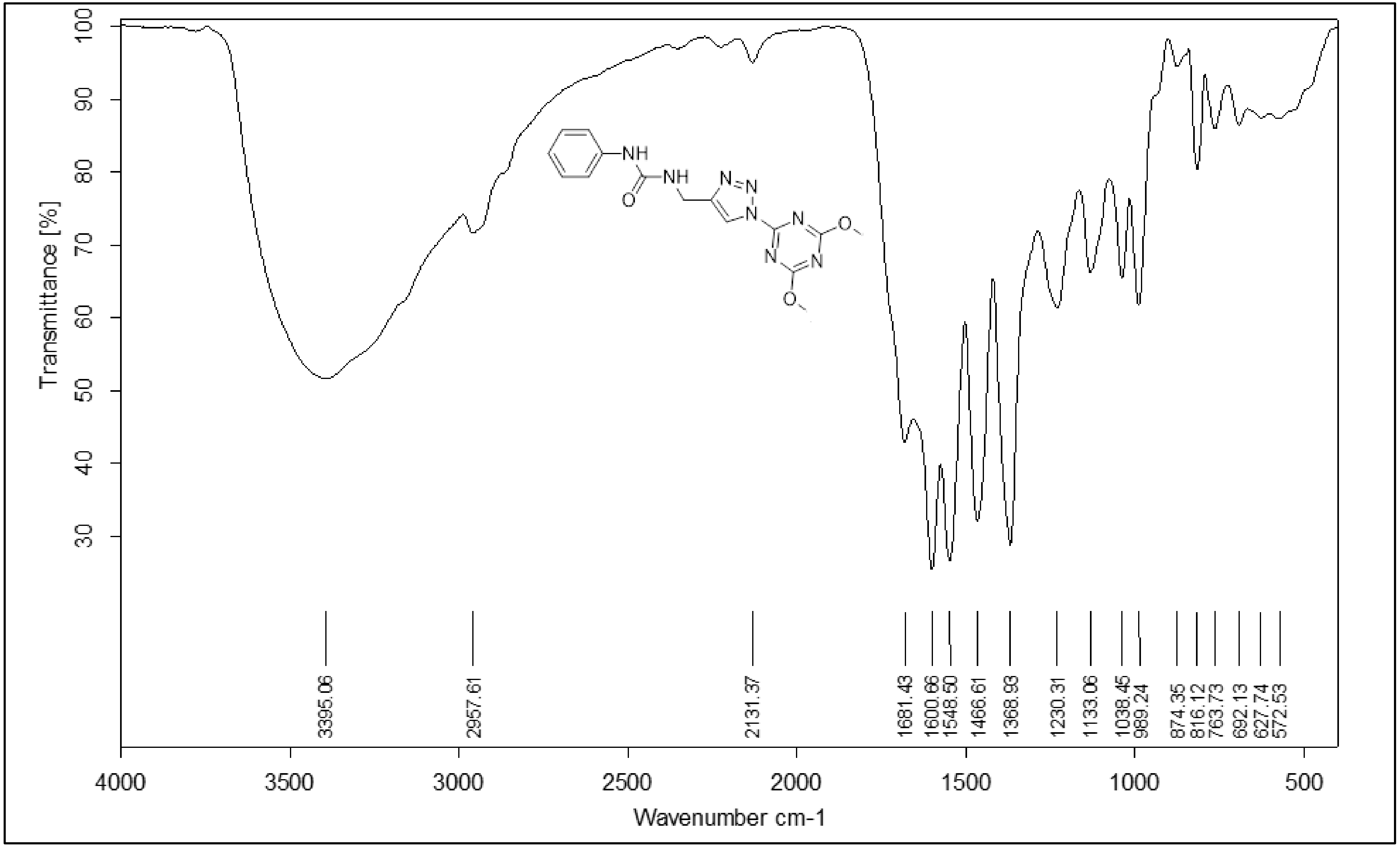

Figure S104: IR (KBr) spectrum of 1-((1-(4,6-dimethoxy-1,3,5-triazin-2-yl)-1*H*-1,2,3-triazol-4-yl) methyl)-3-phenyl urea **(6h)**

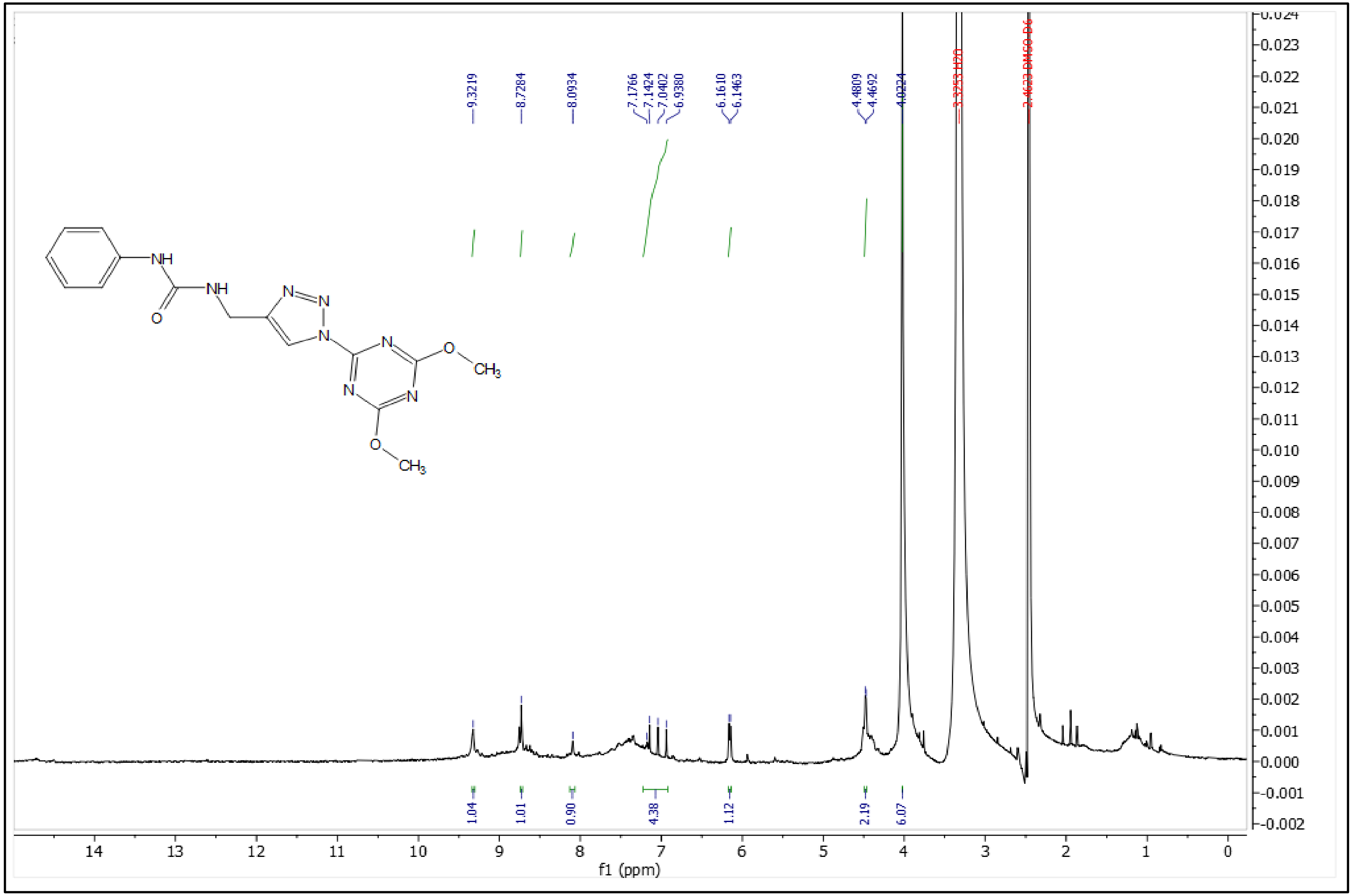

Figure S105: ^{1}H-NMR (500 MHz, DMSO-d_6) spectrum of 1-((1-(4,6-dimethoxy-1,3,5-triazin-2-yl)-1*H*-1,2,3-triazol-4-yl) methyl)-3-phenyl urea **(6h)** **(with solvent peaks)**

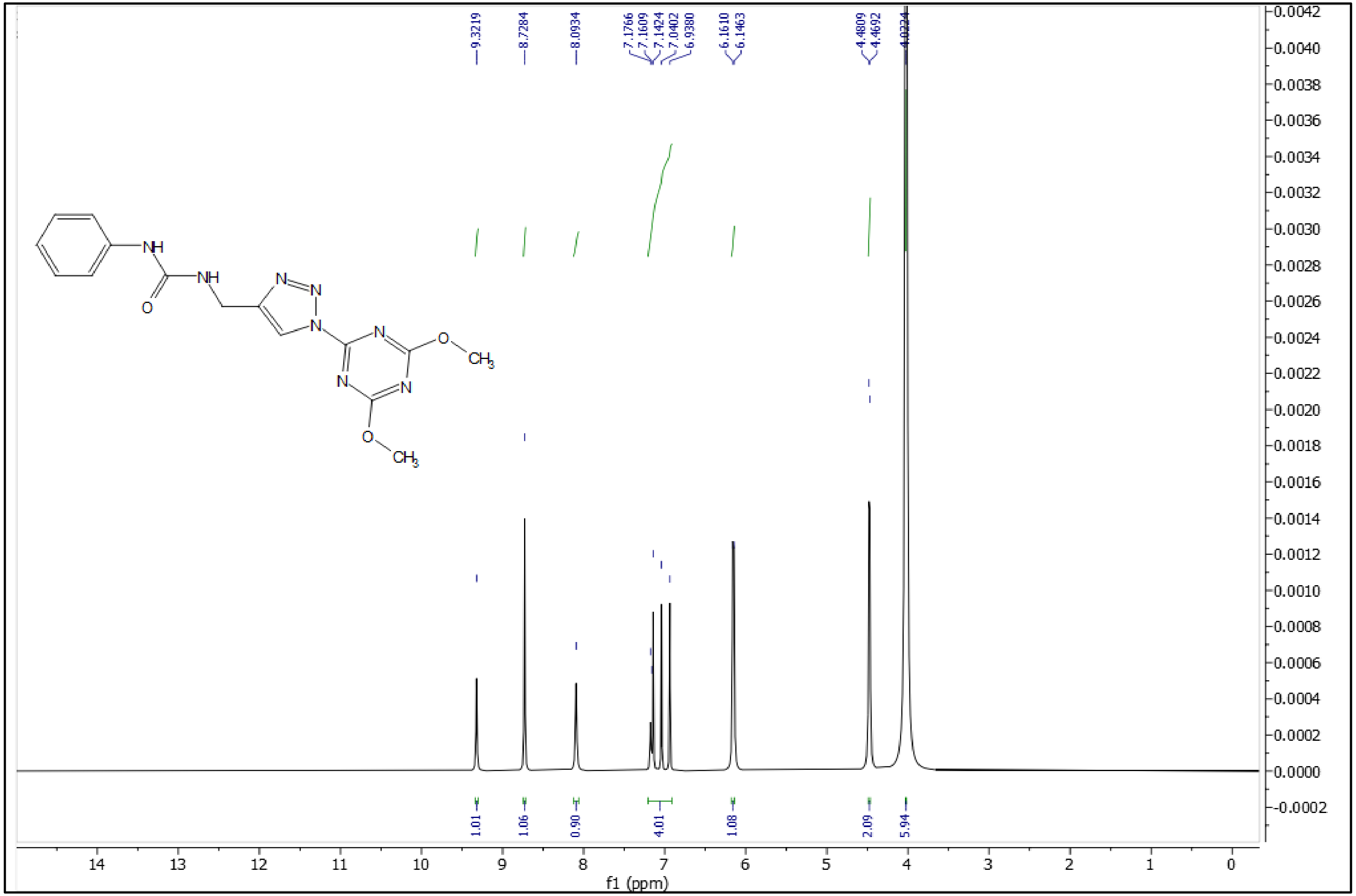

Figure S106: ^{1}H-NMR (500 MHz, DMSO-d_6) spectrum of 1-((1-(4,6-dimethoxy-1,3,5-triazin-2-yl)-1*H*-1,2,3-triazol-4-yl) methyl)-3-phenyl urea **(6h)** **(without solvent peaks)**

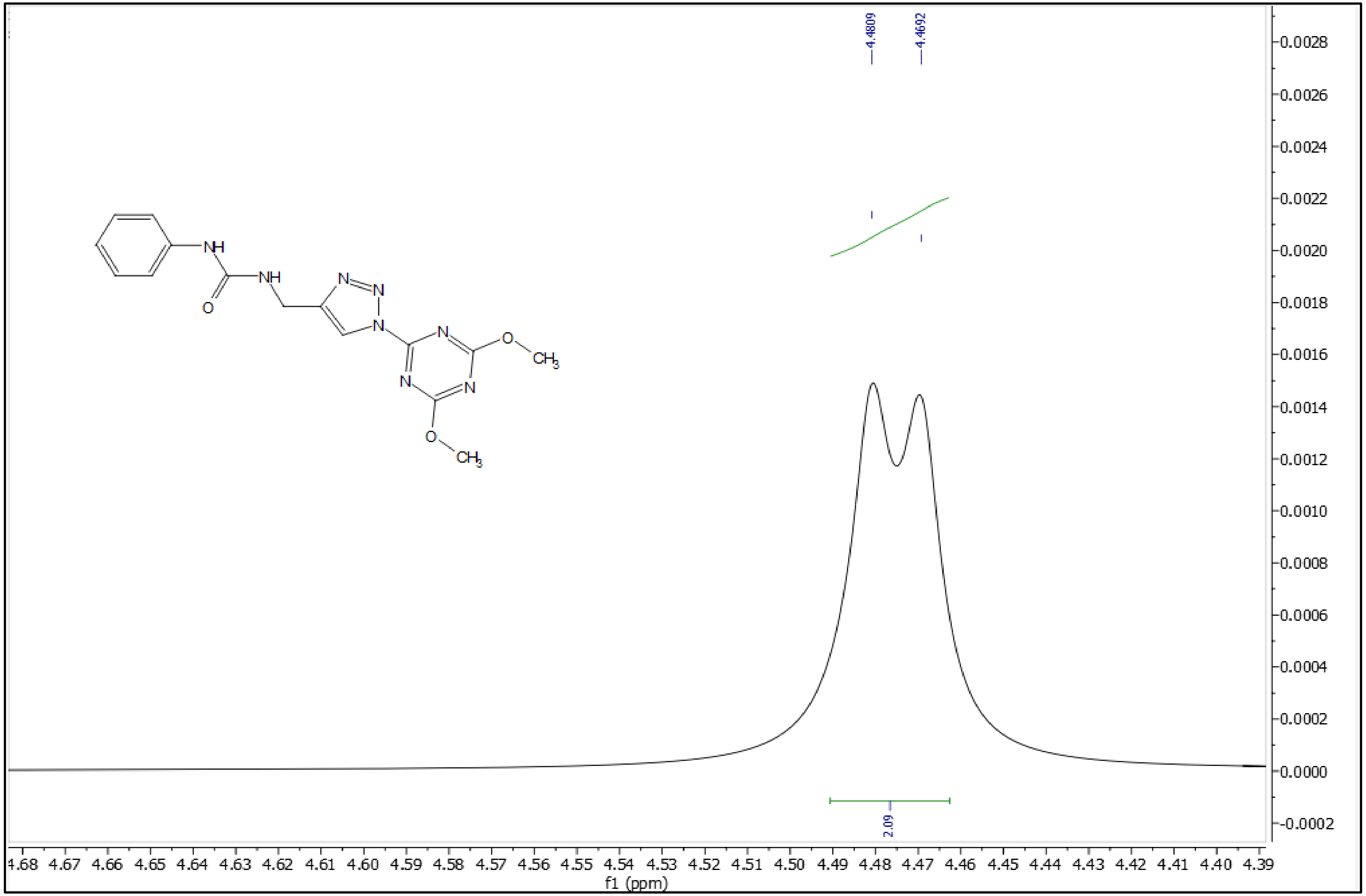

Figure S107: ^{1}H-NMR (500 MHz, DMSO-d_6) spectrum of 1-((1-(4,6-dimethoxy-1,3,5-triazin-2-yl)-1*H*-1,2,3-triazol-4-yl) methyl)-3-phenyl urea **(6h)**

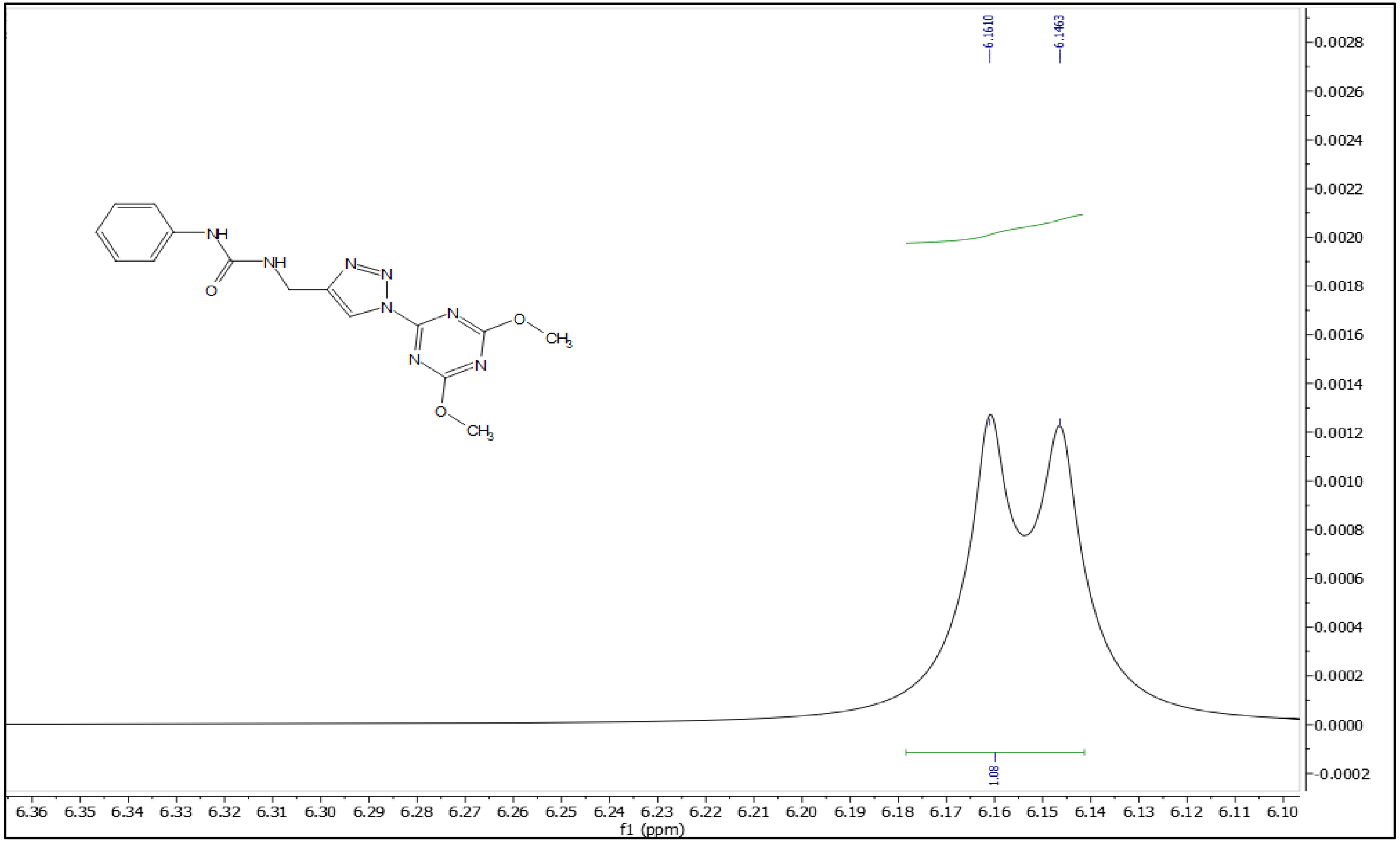

Figure S108: ^{1}H-NMR (500 MHz, DMSO-d_6) spectrum of 1-((1-(4,6-dimethoxy-1,3,5-triazin-2-yl)-1*H*-1,2,3-triazol-4-yl) methyl)-3-phenyl urea **(6h)**

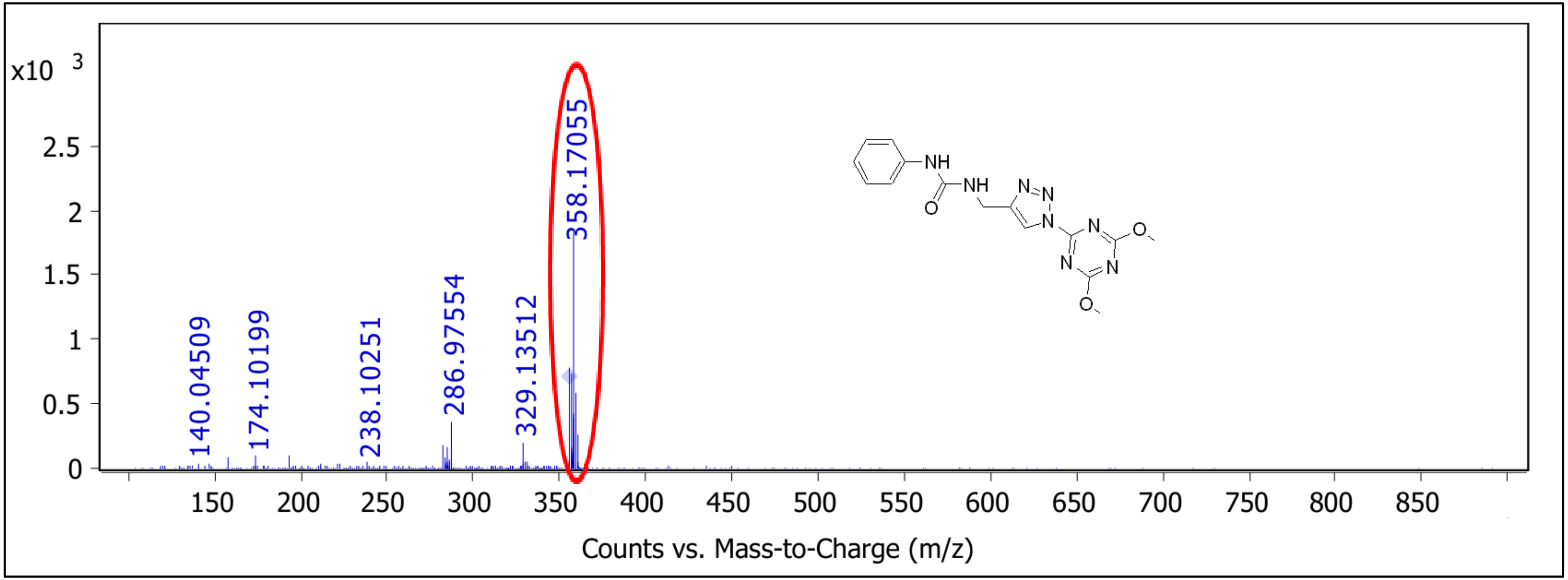

Figure S109: HRMS of 1-((1-(4,6-dimethoxy-1,3,5-triazin-2-yl)-1*H*-1,2,3-triazol-4-yl) methyl)-3-phenyl urea **(6h)**

For high resolution spectra, kindly access this link: https://drive.google.com/drive/folders/1EVncZxf_dYPtpCqXyC0X7jtCEzuzosjk?usp=sharing
